# QTL mapping for nine drought-responsive agronomic traits in bread wheat under irrigated and rain-fed environments

**DOI:** 10.1371/journal.pone.0182857

**Published:** 2017-08-09

**Authors:** Vijay Gahlaut, Vandana Jaiswal, Bhudeva S. Tyagi, Gyanendra Singh, Sindhu Sareen, Harindra S. Balyan, Pushpendra Kumar Gupta

**Affiliations:** 1 Department of Genetics and Plant Breeding, Ch. Charan Singh University, Meerut, India; 2 ICAR-Indian Institute of Wheat and Barley Research, Karnal, India; Institute of Genetics and Developmental Biology Chinese Academy of Sciences, CHINA

## Abstract

In bread wheat, QTL interval mapping was conducted for nine important drought responsive agronomic traits. For this purpose, a doubled haploid (DH) mapping population derived from Kukri/Excalibur was grown over three years at four separate locations in India, both under irrigated and rain-fed environments. Single locus analysis using composite interval mapping (CIM) allowed detection of 98 QTL, which included 66 QTL for nine individual agronomic traits and 32 QTL, which affected drought sensitivity index (DSI) for the same nine traits. Two-locus analysis allowed detection of 19 main effect QTL (M-QTL) for four traits (days to anthesis, days to maturity, grain filling duration and thousand grain weight) and 19 pairs of epistatic QTL (E-QTL) for two traits (days to anthesis and thousand grain weight). Eight QTL were common in single locus analysis and two locus analysis. These QTL (identified both in single- and two-locus analysis) were distributed on 20 different chromosomes (except 4D). Important genomic regions on chromosomes 5A and 7A were also identified (5A carried QTL for seven traits and 7A carried QTL for six traits). Marker-assisted recurrent selection (MARS) involving pyramiding of important QTL reported in the present study, together with important QTL reported earlier, may be used for improvement of drought tolerance in wheat. In future, more closely linked markers for the QTL reported here may be developed through fine mapping, and the candidate genes may be identified and used for developing a better understanding of the genetic basis of drought tolerance in wheat.

## Introduction

Wheat (*Triticum aestivum* L.) is an important staple crop worldwide, contributing ~20% of the total caloric intake in humans [1]. In order to feed the growing human population with increasing per capita income and consumption, global wheat production need to increase by at least 50% by the year 2030 [2]. This target needs to be achieved despite reduced land area, reduced water for irrigation and the predicted climate change. It appears to be difficult in view of the fact that the rate of annual growth in wheat production has shown a decline from 3% to less than 1% in recent years [3]. It has been shown that the major constraint for average global wheat productivity is due to water/drought stress [4–7]. Currently 70% of the cultivated wheat area experiences water stress globally [8], which may further increase due to future climate changes. Even the irrigated wheat growing areas are expected to experience water scarcity [9], making the development of water-use efficient and/or drought-resilient wheat varieties a priority research area for wheat breeders. Therefore, the development of strategies to increase wheat productivity under water stress (along with other biotic and abiotic stresses) is currently receiving world-wide attention. A global Wheat Yield Consortium (WYC) has also been constituted to address the problem of wheat productivity under abiotic stresses like drought and heat [2,10].

In recent years, major efforts have been made to develop drought-tolerant and water use efficient crop cultivars by traditional breeding, with only limited success [9,11,12]. Low heritability and large “genotype × environment” interactions for drought-responsive traits under drought are considered to be the major cause for this limited success [13–15]. It is thus obvious that further knowledge of genetic architecture of important agronomic traits under drought stress could facilitate wheat breeding for drought tolerance.

In the past, QTL analysis (including both linkage-based QTL interval mapping and LD-based association mapping) has been used to identify wheat genomic regions associated with drought-related complex traits, such as yield under drought and/or heat-stress [16–20]. As a result, QTL as well as meta-QTL have been identified for grain yield and yield components in wheat under drought [6,11,18–25]. However, only a few QTL studies have been conducted under drought/rain-fed conditions that are experienced by the crop in Indian sub-continent [26,27]. Consequently, there is an urgent need to identify QTL for grain yield and related traits under conditions of water stress in India. This will facilitate deployment of marker-assisted recurrent selection (MARS) in breeding programmes aimed at producing drought tolerant wheat genotypes for India.

In view of the above, during the present study, single-locus and two-locus QTL analyses for nine drought responsive agronomic traits were conducted using a DH population derived from the cross Kukri (drought sensitive)/Excalibur (drought tolerant); the population was evaluated under 22 environments in India, which included both irrigated and rain-fed conditions. Drought sensitivity index (DSI) for each trait were also calculated and QTL that affected DSI were also identified. The QTL identified during the present study along with those reported earlier may prove useful for developing drought tolerant wheat cultivars for water stress conditions; deployment of marker assisted recurrent selection (MARS) is recommended for this purpose.

## Material and methods

### Plant material (mapping population)

The mapping population used in the present study consisted of 192 doubled haploid (DH) lines; the mapping population was produced at the University of Adelaide in Australia from a cross between Excalibur and Kukri. Excalibur is a drought tolerant cultivar having the following pedigree: RAC177/‘Monoculm’//RAC311S and was released in 1999. Similarly, Kukri is a drought sensitive cultivar having the following pedigree: 76ECN44/76ECN36// MADDEN/6*RAC177 and was released in 1991. The strategy used for the development of the DH populations is described elsewhere [28].

### Field experiments and phenotypic evaluation

The 192 DH lines and their parents were grown at four locations under irrigated (IR) and rain-fed (RF) conditions over three crop seasons (2010–11 to 2012–13). At Hisar, the material was evaluated over two crop seasons only (2011–12 to 2012–13), so that the total number of environments were only 22 instead of the possible 24. The details of locations, crop seasons and other related information are presented in Table 1. In IR environments, four irrigations [1^st^, 21 days after sowing (DAS); 2^nd^, 40 DAS; 3^rd^, 60 DAS; 4^th^, 80 DAS] were given. In the RF environments, single irrigation was given at 21 DAS to allow the crop to establish and to avoid complete crop failure (more details in S1 Table). To avoid the possible adverse effect of high temperature and heat stress at the end of the season, the IR and RF trials were planted at the normal date of sowing in three crop-seasons. Harvesting was done in late March or early April in each crop-season, to avoid experience of heat stress in late April. The details of daily minimum and maximum temperatures during three crop seasons (2010–11 to 2012–13) at four locations in India are presented as supplementary data (S1 Fig). Augmented experimental design was used and comprised 12 blocks, each block with 16 DH lines and three check genotypes (NI5439, PBW175, and WH147). Each line in a block consisted of a plot of three rows, each row of 1.5 m length, with row-to-row distance of 25 cm. In each experiment, the seed rate was 10 g seed/m^2^ for each genotype. Standard agronomic practices were followed for conducting the experiments.

**Table 1:** Details of 22 environments used for phenotyping of the Kukri/Excalibur DH population.

Crop-season	Environment codes	Location	Water condition	Coordinates	Altitude (M)	ME*	Rainfall (mm)**
2010–11	E01	Kanpur	Irrigated	26° 27' N 80° 14' E	126	ME1	72.7
	E02	Kanpur	Rain-fed	26° 27' N 80° 14' E	126	ME1	72.7
	E03	Karnal	Irrigated	29.68°N 76.98°E	227	ME1	101.4
	E04	Karnal	Rain-fed	29.68°N 76.98°E	227	ME1	101.4
	E05	Pune	Irrigated	18° 31' N 73° 52'E	560	ME5	208.4
	E06	Pune	Rain-fed	18° 31' N 73° 52'E	560	ME5	208.4
2011–12	E07	Kanpur	Irrigated	26° 27' N 80° 14' E	126	ME1	74.2
	E08	Kanpur	Rain-fed	26° 27' N 80° 14' E	126	ME1	74.2
	E09	Karnal	Irrigated	29.68°N 76.98°E	227	ME1	17.6
	E10	Karnal	Rain-fed	29.68°N 76.98°E	227	ME1	17.6
	E11	Hisar	Irrigated	29.15° N, 75.70° E	215	ME1, 4	17.7
	E12	Hisar	Rain-fed	29.15° N, 75.70° E	215	ME1, 4	17.7
	E13	Pune	Irrigated	18° 31' N 73° 52'E	560	ME5	130.8
	E14	Pune	Rain-fed	18° 31' N 73° 52'E	560	ME5	130.8
2012–13	E15	Kanpur	Irrigated	26° 27' N 80° 14' E	126	ME1	123.1
	E16	Kanpur	Rain-fed	26° 27' N 80° 14' E	126	ME1	123.1
	E17	Karnal	Irrigated	29.68°N 76.98°E	227	ME1	299.5
	E18	Karnal	Rain-fed	29.68°N 76.98°E	227	ME1	299.5
	E19	Hisar	Irrigated	29.15° N, 75.70° E	215	ME1, 4	152.1
	E20	Hisar	Rain-fed	29.15° N, 75.70° E	215	ME1, 4	152.1
	E21	Pune	Irrigated	18° 31' N 73° 52'E	560	ME5	125.8
	E22	Pune	Rain-fed	18° 31' N 73° 52'E	560	ME5	125.8

* ME, Mega environments

**Total rain-fall during the crop-season

The data on the nine traits were recorded (S2 Table) as follows. (i) Germination percentage (GP): the emergence of radical/plumule from soil was taken as an indication of germination; the germination per cent in each plot was recorded daily up to 10 days after sowing; (ii) days to anthesis (DTA): calculated as days from date of sowing to extrusion of anthers in 75% ears; (iii) days to maturity (DTM): calculated as days from the date of sowing to maturity (maturity means physiological maturity, i.e. yellowing of at least 75% spikes): (iv) grain filling duration (GFD): calculated as the number of days from DTA to DTM; (v) plant height (PH): measured at the time of physiological maturity in cm, as the distance between the ground and the tip of the spike, excluding awns (the average of five measurements/plot); (vi) grain weight/ear (GWPE): calculated as the mean of grain weights of five ears per plot (in g); (vii) productive tillers/m^2^ (PTPM): recorded as number of productive tillers (ear bearing tillers)/m^2^; (viii) 1000-grain weight (TGW): estimated as weight of 1000-grains in g; (ix) grain yield per plot (GYPP): estimated as weight of harvested grains per plot in g. Drought sensitivity index (DSI) for each trait (T) was calculated according to Fischer and Maurer [29] as follows: *DSI*=[(1−*TDS*/*TC*)/*D*], where TDS represents trait values under drought stress (DS) and TC represents values under irrigated or control (C = well-watered) environments; D is drought intensity, calculated as follows: *D*=1−(*XDS*/*XC*), where XDS and XC are the mean values of the trait in all DH lines under DS and C environments, respectively.

### Statistical analysis

Descriptive statistics like mean, standard error (SE), range, coefficient of variation (CV%), Analysis of Variance (ANOVA) and heritability for each agronomic trait, and correlations among pairs of traits were calculated using the SPSS17.0 software (http://www.spss.com). Best linear unbiased prediction (BLUP) value (i.e. values pooled over multi-location and multi-year data) for each trait was calculated according to Merk et al. [30] using the R package nlme (http://www.r-project.org/). Following model was used:

$$Y_{ijk}=m+G_{i}+L_{j}+Y_{k}+R(Y_{k}\times L_{j})+GL_{ij}+GY_{ik}+e_{ijk}$$

where Y_ijk_ is the trait measured, m is the overall mean, G_i_ is the effect resulting from the ith genotype, L_j_ is the effect resulting from the j^th^ location, Y_k_ is the effect resulting from the k^th^ year, *R*(*Y*_*k*_×*L*_*j*_) is the effect resulting from replicate within year × location, GL_ij_ is the effect resulting from genotype × location interaction, GY_ik_ is the effect resulting from genotype × year interaction, and e_ijk_ is the residual error (effect resulting from experimental error). All effects were treated as random.

### Construction of linkage map

The molecular linkage map of the Kukri/Excalibur population was originally constructed at the Australian Centre for Plant Functional Genomics (ACPFG) as described by Edwards [31]. The genetic markers used for construction of the linkage map comprised 392 polymorphic markers including 222 DArT (Diversity Arrays Technology) markers, 169 SSR (simple sequence repeats) markers, and a gene-based marker for *Vrn-A1*. The markers were placed in linkage groups using the program MAPMAKER/EXP v3.0b [32]. A LOD score of 3.0 was set as the minimum threshold to indicate linkage between markers. Kosambi mapping function was used to convert recombination frequencies in cM values [33]. The final map was drawn using the MapChart program, v.2.1 [34].

### QTL analysis

Composite interval mapping (CIM) was performed using QTL Cartographer v2.5 [35]. The parameter settings for CIM were as follow: model 6 of Z-mapqtl, forward and backward stepwise regression with a threshold of P < 0.05 to select cofactors, window size 10 cM, and 2 cM walking speed along each chromosome. Multi-trait analysis involving multi-trait composite interval mapping (MCIM) was also conducted using the module JZmapqtl available in QTL Cartographer with the objective to detect pleiotropic/tightly-linked QTL. LOD scores and values for coefficients of determination (R^2^ = PVE) for each QTL were available through CIM. QTL having LOD scores greater than the empirical genome-wide and experiment-wise threshold LOD value (calculated from 1000 permutations for P< 0.01) were declared as significant.

Two-locus QTL analysis was conducted using the software QTLNetwork version 2.1 [36] to identify epistatic interactions (QQ), QTL x environment interactions (QE and QQE) and QTL effects. A ‘2D genome scan’ option was used to map epistatic QTL with or without single-locus effects. Using the ‘‘permutation” option, critical F values were calculated to control the type I error rate. QTL designations were assigned using standard nomenclature available in the catalogue of wheat gene symbols [37].

## Results

### Phenotypes of DH mapping population

**Fig 1:**
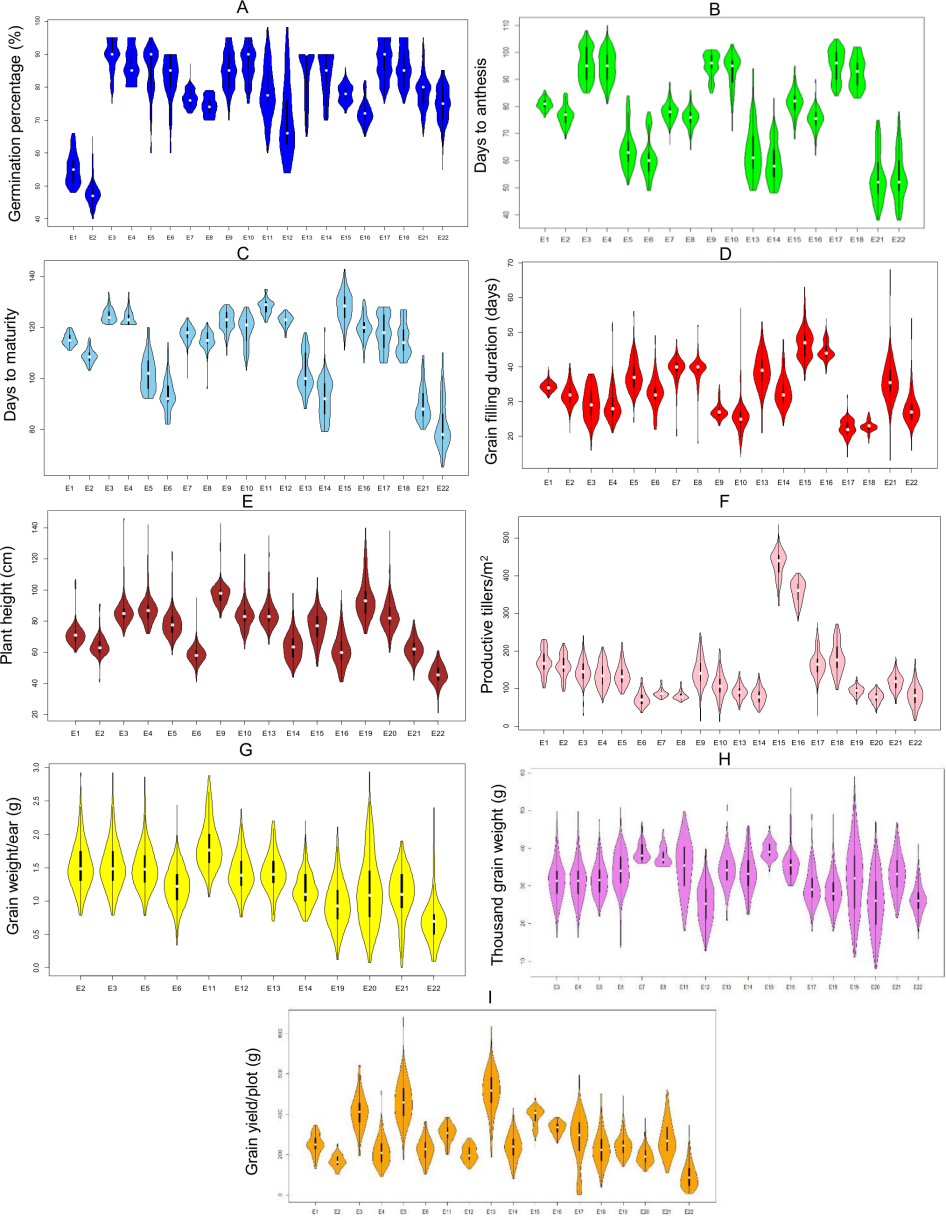
Violin plots for nine agronomic traits (A-I) measured on the Kukri/Excalibur DH mapping population in irrigated (IR) and rain-fed (RF) environments (for environment codes, refer Table 1).

Descriptive statistics including values of mean, SE, range, coefficient of variation (CV %), ANOVA, estimates of heritability and violin plots showing distribution for all the nine agronomic traits in DH population in 22 environments are summarized in S3 & S4 Tables and Fig 1. Similar data for DSI of the nine agronomic traits under 12 different environments are presented in S5 Table. The phenotypic values for each trait exhibited wide range, with the CV ranging from 1.93% (DTM) to 35.66% (GWPE) in IR environments and 1.96% (DTM) to 35.76% (PTPM) in RF environments. As expected, in almost all cases, the mean trait values in IR environments were higher than those in the respective RF environments. The estimates of heritability for the nine (9) traits in different environments varied from low (>20%) to moderate (20% < H^2^ > 50%) and high (>50%; S3 Table). A minimum of 10% heritability was observed for DTM (in E07), PH (in E01), and PTPM (in E03), and the maximum of 99% heritability was observed for DTA (in E03). For each trait, transgressive segregation was also observed both under the IR and RF environments. In summary, the extent of available variation and heritability for different traits were suitable for QTL analysis.

### Correlations among traits

Values of correlation coefficients among nine agronomic traits based on data pooled over IR and RF environments are presented in Table 2. Both in the IR and RF environments, nearly half of the trait pairs, which involved almost all the traits, exhibited significant positive or negative correlations. The magnitudes of correlations were higher under IR environment relative to those in the RF environment for most of the trait pairs. Two traits (GWPE and TGW) in IR environment and five traits (GP, PH, PTPM, GWPE and TGW) in RF environment also had significant positive correlations with GYPP.

**Table 2:** Pearson’s correlation coefficients (r values) among different agronomic traits based on pooled data of Kukri/Excalibur DH mapping population in IR and RF environments.

Trait	GP	DTA	PH	DTM	PTPM	GWPE	GFD	TGW	GYPP
**GP**	1	0.140	0.090	0.150*	0.110	-0.320**	-0.140	-0.20**	0.130
**DTA**	0.03	1	0.130	0.854**	0.354**	-0.181*	-0.621**	-0.294**	-0.071
**PH**	-0.189**	0.254**	1	0.239**	0.090	0.156*	0.126	0.191**	0.112
**DTM**	0.094	0.900**	0.258**	1	0.286**	-0.162*	-0.177*	-0.211**	-0.060
**PTPM**	0.125	0.229**	0.042	0.195**	1	-0.188*	-0.259**	-0.264**	-0.058
**GWPE**	-0.117	-0.153*	0.408**	-0.109	-0.109	1	0.120	0.548**	0.344**
**GFD**	0.156*	-0.330**	-0.012	0.040	-0.095	0.072	1	0.220**	-0.008
**TGW**	0.049	-0.225**	0.386**	-0.199**	0.099	0.472**	0.017	1	0.280**
**GYPP**	0.224**	-0.050	0.246**	0.029	0.157*	0.285*	0.106	0.392**	1

Values above the diagonal indicate r values using pooled data of IR environment; values below the diagonal indicate r values using pooled data of RF environments

*, ** Significant at P≤0.05 and P≤0.0, respectively

GP, germination percentage; DTA, days to anthesis; DTM, days to maturity; GFD, grain filling duration; PH, plant height; GWPE, grain weight/ear; PTPM, productive tillers/m^2^; TGW, 1000 grain weight; GYPP, grain yield /plot.

### Linkage map of DH population

The Kukri/Excalibur genetic linkage map had a length of 1598.7 cM and consisted of 21 linkage groups, with a total of 392 marker loci. The A sub-genome had the highest coverage, with 168 marker loci, while the D sub-genome had the lowest coverage with 70 markers; the B sub-genome had 154 markers (S6 Table). It is apparent that A and B sub-genomes had each more than double the number of markers mapped on the D sub-genome, although the genetic length in cM did not differ markedly among the three sub-genomes. The smallest linkage group belonged to chromosome 4D (4 markers; 20.2 cM) and the longest linkage group belonged to 7D (13 markers; 107.9 cM). Apparently, there was no relationship between the number of markers and the length of individual linkage groups, as evident from the fact that the longest linkage group (107.9 cM) on 7D had only 13 markers (S6 Table), while another long linkage group on 7A (105.2 cM) had the highest number of markers (38).

### Single-locus analysis

Single-locus single trait (SLST) included identification of (*i*) QTL for nine individual traits, (*ii*) independent QTL affecting DSI for these nine traits, and (*iii*) QTL identified through multi-trait analysis involving correlated traits.

#### QTL for nine individual traits

**Fig 2:**
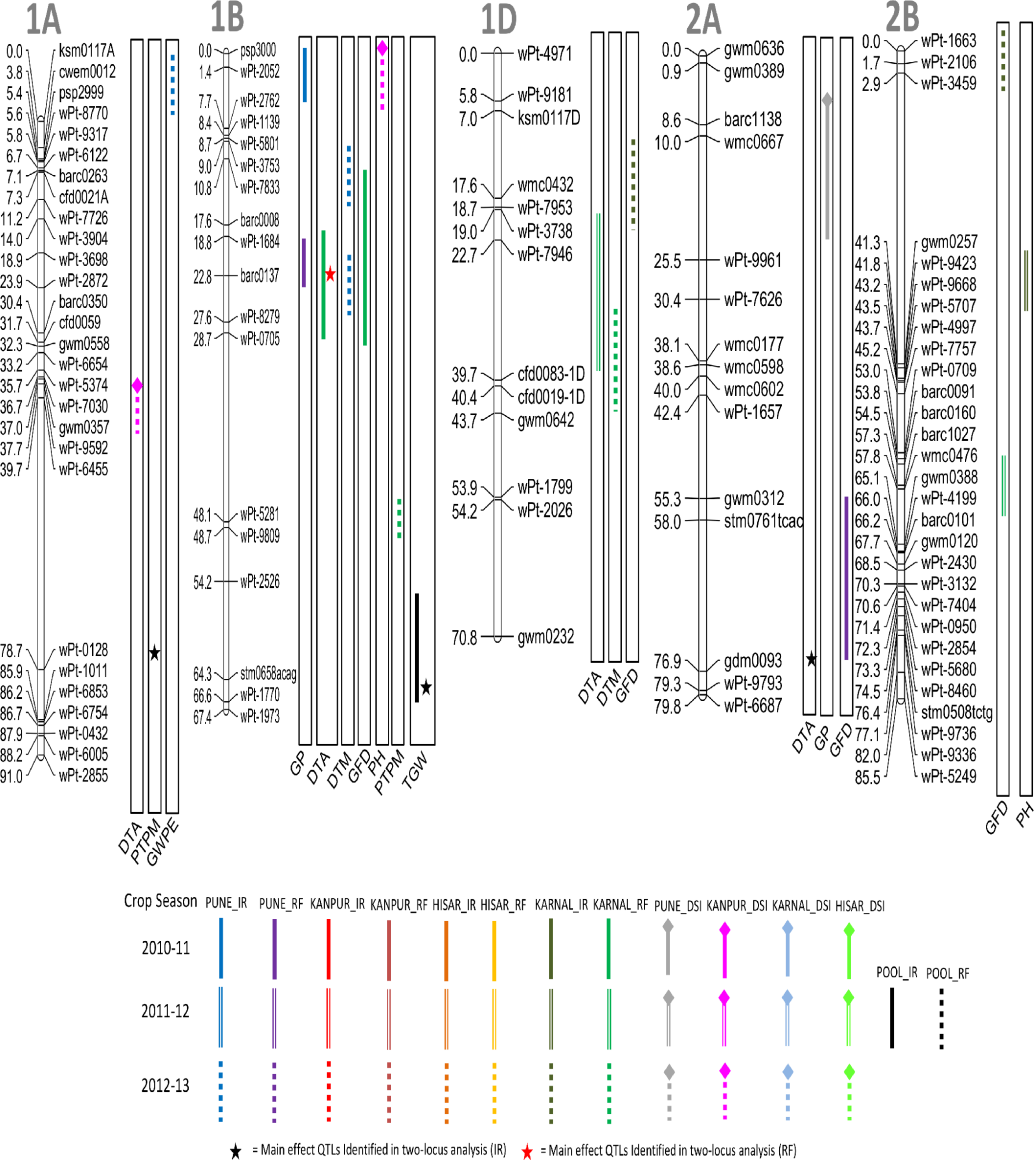
Linkage maps of chromosomes 1A, 1B, 1D, 2A, and 2B showing QTL on the right side and centimorgan (cM) distance on the left. A coloured bar represents the CI (confidence interval) of QTL identified through single-locus analysis. Asterisks represent closest marker of main effect QTL identified through QTL Network. Different colors and styles of bars represent different environments. For details of abbreviations of traits, refer footnote of Table 2.

**Fig 3:**
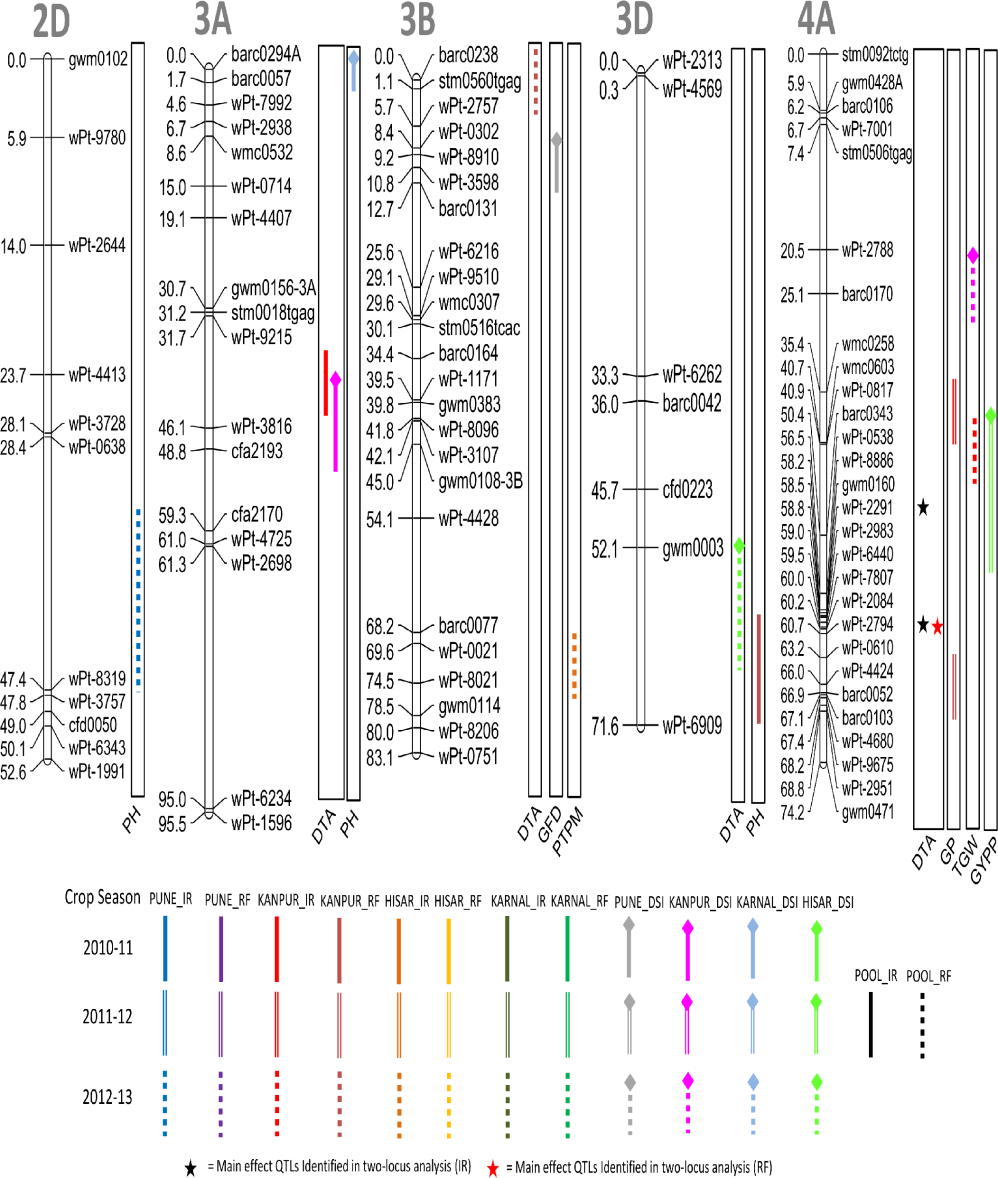
Linkage maps of chromosomes 2D, 3A, 3B, 3D and 4A showing QTL on the right side and centimorgan (cM) distance on the left. A coloured bar represents the CI (confidence interval) of QTL identified through single-locus analysis. Asterisks represent closest marker of main effect QTL identified through QTL Network. Different colors and styles of bars represent different environments. For details of abbreviations of traits, refer footnote of Table 2.

**Fig 4:**
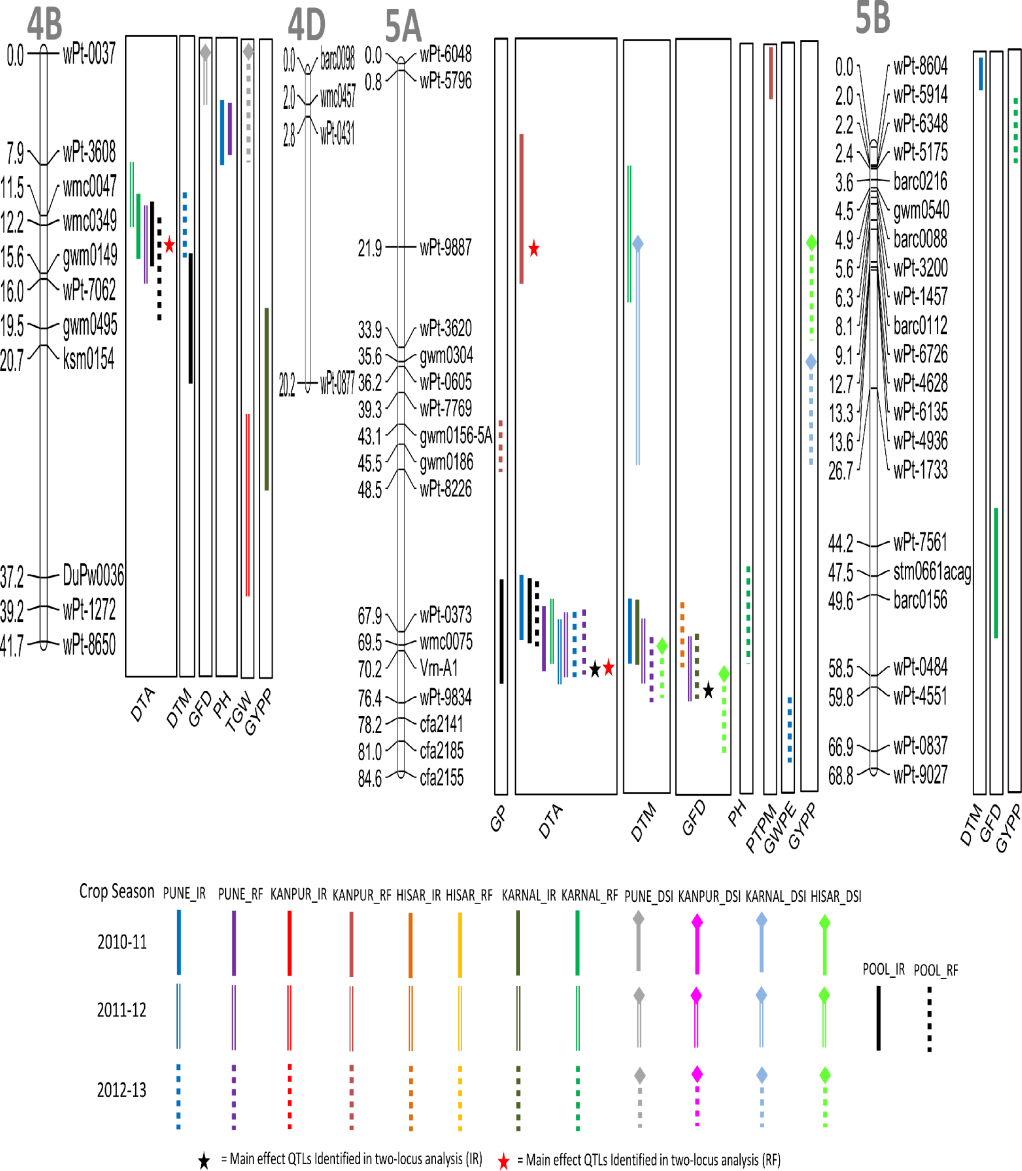
Linkage maps of chromosomes 4B, 4D, 5A and 5B showing QTL on the right side and centimorgan (cM) distance on the left. A coloured bar represents the CI (confidence interval) of QTL identified through single-locus analysis. Asterisks represent closest marker of main effect QTL identified through QTL Network. Different colors and styles of bars represent different environments. For details of abbreviations of traits, refer footnote of Table 2.

**Fig 5:**
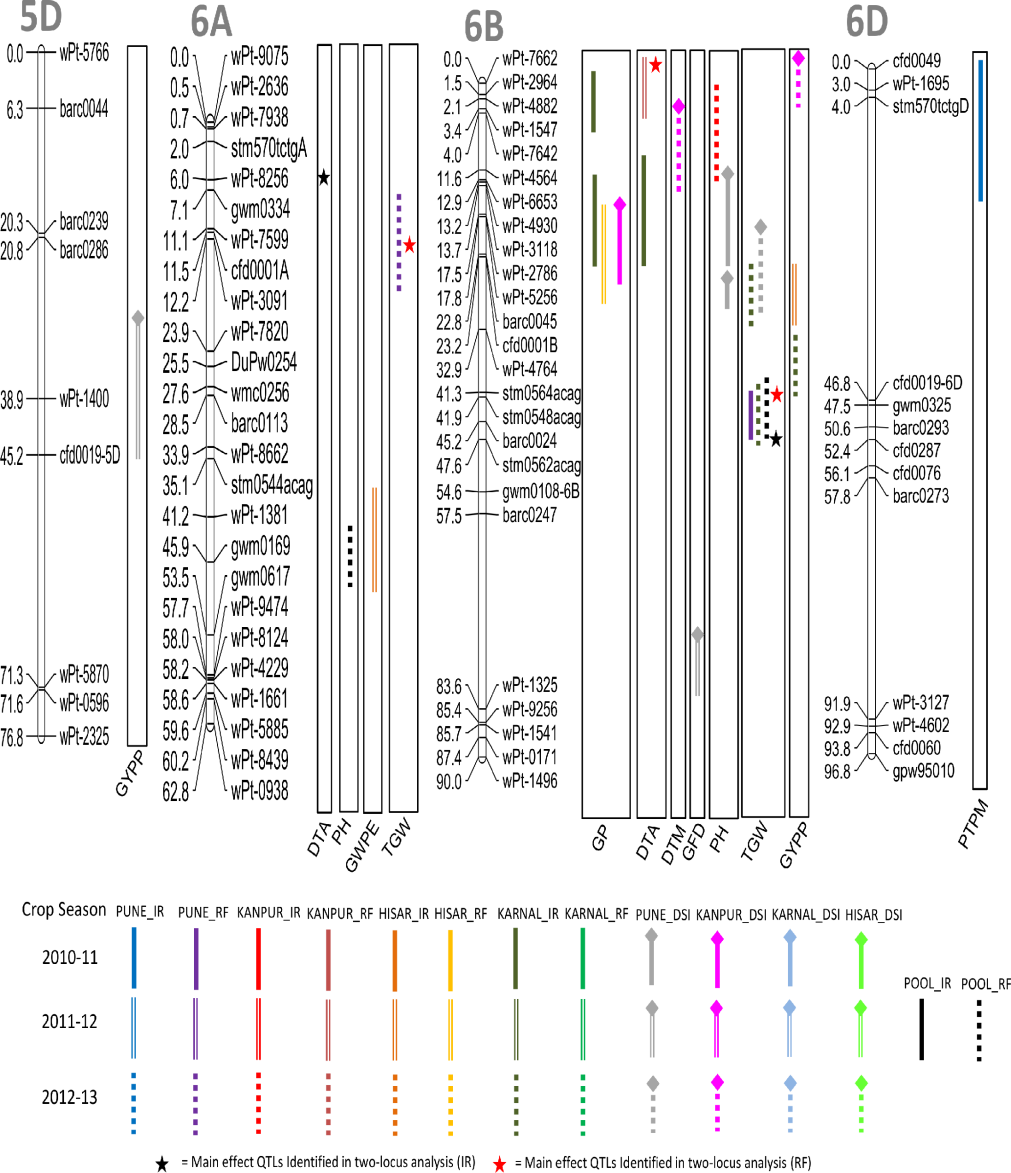
Linkage maps of chromosomes 5D, 6A, 6B and 6D showing QTL on the right side and centimorgan (cM) distance on the left. A coloured bar represents the CI (confidence interval) of QTL identified through single-locus analysis. Asterisks represent closest marker of main effect QTL identified through QTL Network. Different colors and styles of bars represent different environments. For details of abbreviations of traits, refer footnote of Table 2.

**Fig 6:**
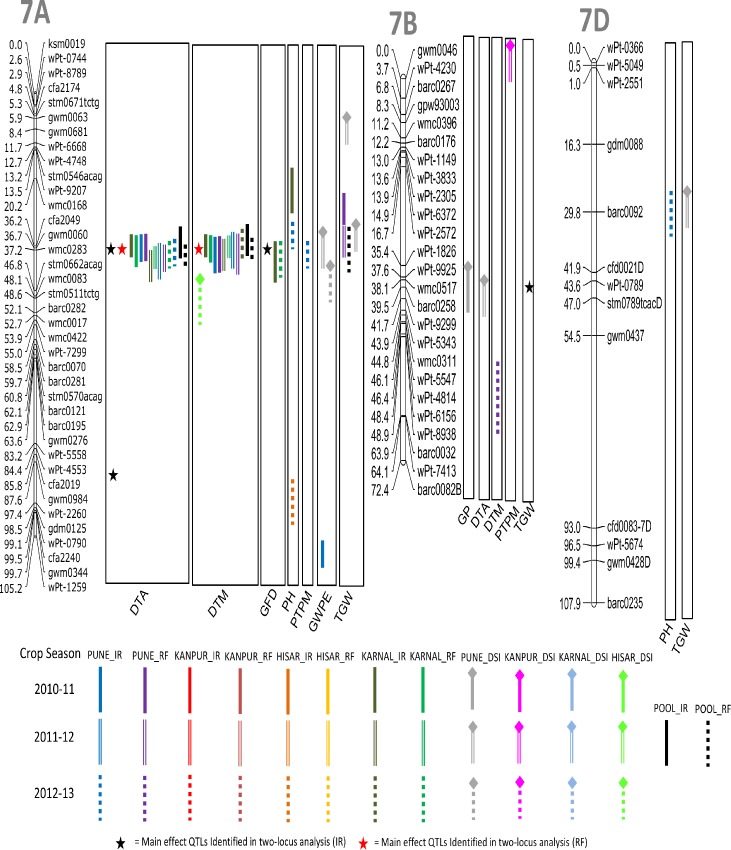
Linkage maps of chromosomes 7A, 7B and 7D showing QTL on the right side and centimorgan (cM) distance on the left. A coloured bar represents the CI (confidence interval) of QTL identified through single-locus analysis. Asterisks represent closest marker of main effect QTL identified through QTL Network. Different colors and styles of bars represent different environments. For details of abbreviations of traits, refer footnote of Table 2.

A total of 66 QTL were detected for nine different agronomic traits using CIM (Table 3; Figs 2–6); these were located on 19 different chromosomes (except 4D and 5D). Relative to other chromosomes, 5A and 7A carried many more QTL for a number of traits within short distances. A minimum of 4 QTL were available each for GWPE and GYPP, and the maximum of 11 QTL were available for PH. Many QTL were identified both in IR (34 QTL) and in RF (23 QTL); only 9 QTL were identified in both; five of these 9 QTL were also identified in the pooled data (3 for DTA, 1 for DTM, and 1 for TGW). LOD scores for individual QTL ranged from 1.80 to 10.50 and PVE ranged from 3.85% to 20.43%. Of the above 66 QTL, 12 QTL were major because each had >10% PVE (for details of these QTL, see Table 3).

**Table 3:** QTL for different agronomic traits identified following composite interval mapping (CIM) using Kukri/Excalibur DH population. The details of phenotypic variation explained (R^2^) and additive effect are also included.

Trait and QTL name	Peak position (CI) in (cM)	Marker nearest peak LOD	Environments	LOD	R^2^ (%)	Additive effect^a^
**1. Germination percentage**		** **		** **	** **	** **
*QGp*.*ccsu-1B*.*1*	0 (00.0–05.0)	*psp3000*	E05	4.05	8.55	1.95
*QGp*.*ccsu-1B*.*2*	19.8 (18.8–22.8)	*wPt-1684*	E06	2.97	7.80	-2.55
*QGp*.*ccsu-4A*.*1*	51.4 (46.1–56.8)	*barc0343*	E07	3.60	8.04	0.10
*QGp*.*ccsu-4A*.*2*	66.9 (63.3–67.4)	*barc0052*	E08	3.35	6.70	-0.09
*QGp*.*ccsu-5A*.*1*	45.5 (43.1–48.3)	*gwm0186*	E16	3.01	6.50	0.10
*QGp*.*ccsu-5A*.*2*	67.9 (59.1–73.3)	*wPt-0373*	P01	3.18	6.32	0.04
*QGp*.*ccsu-6B*.*1*	3.4 (01.6–08.7)	*wPt-1547*	E03	2.83	6.49	1.19
*QGp*.*ccsu-6B*.*2*	13.7 (11.6–17.7)	*wPt-3118*	E01; E12	3.22–3.87	6.89–8.59	0.52
**2. Days to anthesis **						
*QDa*.*ccsu-1B*.*1*	22.8 (18.1–27.2)	*barc0137*	E04	3.23	5.93	1.41
*QDa*.*ccsu-1D*.*2*	39.7 (31.8–40.9)	*cfd0083-1D*	E07	3.17	6.47	0.93
*QDa*.*ccsu-3A*.*3*	45.7 (36.7–49.0)	*wPt3816*	E02	3.60	7.59	0.98
*QDa*.*ccsu-3B*.*1*	1 (00.0–04.6)	*stm0560tgag*	E16	3.07	6.24	0.06
*QDa*.*ccsu-4B*	9.9 (07.2–12.2)	*wmc0349*	E04; E10; E14; P01; P02	3.10–5.10	5.8–8.94	-1.35 to -2.8
*QDa*.*ccsu-5A*.*1*	17.8 (09.4–25.8)	*wPt9887*	E02	3.86	10.59	1.15
*QDa*.*ccsu-5A*.*2*	67.9 (59.9–74.4)	*Vrn-A1*	E05; E06; E10; E13; E14; E21; E22; P01; P02	3.54–8.58	6.49–16.13	1.03 to 3.63
*QDa*.*ccsu-6B*.*1*	2.1 (00.0–04.4)	*wPt-4882*	E08	3.80	5.43	-0.98
*QDa*.*ccsu-6B*.*2*	13.7 (09.5–16.7)	*wPt-3118*	E03	3.58	6.52	0.96 to 1.50
*QDa*.*ccsu-7A*	37.2 (32.5–45.9)	*wmc0283*	E03; E04; E05; E06; E09; E10; E13; E14; E18; E21; P01; P02	3.03–10.5	4.56–20.43	-0.06 to -3.35
**3. Days to maturity**						
*QDm*.*ccsu-1B*.*1*	12.8 (10.4–17.1)	*wPt-7833*	E21	3.94	8.59	2.09
*QDm*.*ccsu-1B*.*2*	23.8 (22.3–29.7)	*barc0137*	E21	3.88	7.81	1.97
*QDm*.*ccsu-1D*	39.7 (31.3–41.9)	*cfd0083-1D*	E18	3.43	7.12	1.70
*QDm*.*ccsu-4B*	12.2 (11.0–15.1)	*wmc0349*	E21; P01	3.28–3.44	6.51–6.59	-0.94 to -2.47
*QDm*.*ccsu-5A*.*1*	21.9 (10.5–31.1)	*wPt-9887*	E10	2.66	5.77	-2.27
*QDm*.*ccsu-5A*.*2*	67.9 (58.0–71.9)	*Vrn-A1*	E05; E09; E14; E21	3.20–6.39	6.11–12.10	1.02–3.20
*QDm*.*ccsu-5B*	0 (00.0–01.5)	*wPt8604*	E05	3.97	7.44	0.75 to 1.94
*QDm*.*ccsu-7A*	37.2 (29.4–46.4)	*wmc0283*	E03; E04; E06; E05; E10; E09; E13; E14; E17; P02; P01	3.16–7.09	8.00–16.6	-0.76 to -2.82
*QDm*.*ccsu-7B*	52.9 (47.4–63.1)	*wPt-8938*	E22	3.13	6.39	-2.36
**4. Grain filling duration**						
*QGfd*.*ccsu-1B*	22.8 (10.7–29.9)	*barc0137*	E04	2.70	5.69	-1.09
*QGfd*.*ccsu-1D*	18.6 (10.4–21.8)	*wPt-7953*	E15	3.22	6.24	1.39
*QGfd*.*ccsu-2A*	70 (57.8–76.9)	*stm0761tcac*	E06	3.06	7.68	-1.17
*QGfd*.*ccsu-2B*.*1*	1.7 (00.0–02.8)	*wPt-2106*	E15	3.51	7.20	1.49
*QGfd*.*ccsu-2B*.*2*	65.1 (61.0–70.3)	*gwm0388*	E08	2.95	6.012	1.01
*QGfd*.*ccsu-5A*	69.5 (64.2–70.3)	*wmc0075*	E13; E17; E19	2.70–5.20	5.6–10.69	-0.9441 to -2.22
*QGfd*.*ccsu-5B*	47.5 (40.6–53.4)	*stm0661acag*	E02	3.86	7.98	0.84
*QGfd*.*ccsu-7A*	37.2 (31.8–47.9)	*wmc0283*	E03; E18	2.67–8.70	5.72–17.24	0.68–1.87
**5. Plant height **						
*QHt*.*ccsu-2B*	52.2 (49.5–52.5)	*wPt-9423*	E09	3.74	10.94	-55.52
*QHt*.*ccsu-2D*	40.4 (31.0–47.8)	*wPt-0638*	E21	3.17	8.52	-2.51
*QHt*.*ccsu-3D*	66.1 (55.8–70.1)	*wPt6909*	E02	2.86	8.54	-1.84
*QHt*.*ccsu-4B*.*1*	7 (03.0–10.8)	*wPt3608*	E05; E06	3.28–4.83	7.17–9.12	1.75 to 2.91
*QHt*.*ccsu-5A*	68.9 (62.8–69.5)	*wPt-0373*	E16	5.93	12.40	6.68
*QHt*.*ccsu-6A*	45.2 (42.5–53.3)	*wPt-1381*	P02	3.38	7.46	-0.07
*QHt*.*ccsu-6B*	7 (01.7–11.6)	*wPt-7642*	E15	3.33	7.62	3.95
*QHt*.*ccsu-7A*.*1*	20.2 (19.3–26.0)	*Wmc168*	E03	3.34	7.35	-3.10
*QHt*.*ccsu-7A*.*2*	36.7 (31.1–37.1)	*gwm0060*	E21	3.19	6.27	-2.52
*QHt*.*ccsu-7A*.*3*	98.5 (94.1–103.5)	*gdm0125*	E19	3.10	6.51	4.74
*QHt*.*ccsu-7D*	31.8 (25.5–38.4)	*barc0092*	E21	4.44	10.04	2.82
**6. Productive tillers/m**^**2**^						
*QPtm*.*ccsu-1B*	48.1 (47.6–50.8)	*wPt-5281*	E16	3.20	7.38	-10.89
*QPtm*.*ccsu-3B*	73.6 (69.4–76.8)	*wPt-0021*	E19	4.20	9.45	-5.47
*QPtm*.*ccsu-5A*	0 (00.0–04.2)	*wPt6048*	E02	4.18	8.82	-8.67
*QPtm*.*ccsu-6D*	3 (00.0–23.8)	*wPt1695*	E05	3.37	6.99	7.41
*QPtm*.*ccsu-7A*	40.2 (35.8–44.9)	*wmc0283*	E21	4.24	10.42	-8.73
**7. Grain weight/ear**						
*Qgwe*.*ccsu-1A*	0 (00.0–03.6)	*ksm0117A*	E21	3.87	7.97	-0.13
*Qgwe*.*ccsu-5A*	81 (79.3–82.9)	*cfa2185*	E21	3.55	7.30	-0.12
*Qgwe*.*ccsu-6A*	45.2 (40.6–54.0)	*wPt-1381*	E11	3.48	7.96	0.14
*Qgwe*.*ccsu-7A*	102.7 (94.9–103.8)	*gwm0344*	E05	3.18	7.30	0.08
**8. Thousand grain weight**						
*QTgw*.*ccsu-1B*	61.2 (54.8–66.3)	*wPt-2526*	P01	3.26	8.01	0.49
*QTgw*.*ccsu-4A*	56.5 (55.1–58.8)	*wPt-0538*	E15	3.27	6.88	-0.82
*QTgw*.*ccsu-4B*	29.7 (23.9–38.7)	*DuPw0036*	E09	3.38	9.74	1.19
*QTgw*.*ccsu-6A*	11.5 (08.6–14.5)	*cfd0001A*	E22	3.47	7.13	-1.19
*QTgw*.*ccsu-6B*.*1*	23.2 (19.7–27.1)	*cfd0001B*	E17	3.94	7.90	-1.74
*QTgw*.*ccsu-6B*.*2*	45.2 (38.5–47.3)	*barc24*	E06; E17; P02	2.95–4.45	5.96–9.37	0.37–2.18
*QTgw*.*ccsu-7A*	36.2 (30.7–36.6)	*wmc028*	E06; E14; P02	3.02–8.57	6.35–18.4	-0.39 to—1.87
**9. Grain yield per plot**						
*QGypp*.*ccsu-4B*	19.5 (18.7–27.0)	*gwm04595*	E03	3.19	6.62	27.74
*QGypp*.*ccsu-5B*	2.2 (01.9–02.7)	*wPt-6348*	E18	4.22	8.70	-34.11
*QGypp*.*ccsu-6B*.*1*	17.8 (14.8–21.2)	*wPt-5256*	E11	4.53	9.79	18.33
*QGypp*.*ccsu-6B*.*2*	36.9 (32.6–41.5)	*wPt-4764*	E17	3.55	8.60	39.62

For location codes refer Table 1; P01, BLUP data of all irrigated environments; P02, BULP data of all rain-fed environments.

^a^ A positive value of the additive main effects (a) indicates that Kukri contributes allele to increase the phenotype value, and a negative value means that Excalibur provides allele to increase the phenotype value.

#### QTL affecting drought sensitivity index (DSI)

As many as 32 QTL affecting DSI for nine traits were identified on the following 14 different chromosomes: 1A, 1B, 2A, 3A, 3B, 3D, 4A 4B, 5A, 5D, 6B, 7A, 7B and 7D (Table 4). Each of the 32 QTL was identified in one environment only (one location for one year) except one QTL (*QDSIGwe*.*ccsu-7A*) which was identified at two locations (LY8; LY12). The number of DSI QTL for individual traits ranged from 2 (each for PTPM and GWPE) to 6 (TGW). The LOD score for these QTL ranged from 2.13 to 7.19 and PVE values ranged from 3.58 to 16.90%.

**Table 4:** QTL for DSI for different agronomic traits identified following composite interval mapping (CIM) using Kukri/Excalibur DH population. Values of phenotypic variation explained (R^2^) and additive effect are also included.

Trait/QTL Name	Peak position (CI) in (cM)	Marker nearest peak LOD	Env.	LOD	R^2^ (%)	Additive effect^a^
**1. Germination percentage**						
*QDSIGp*.*ccsu-2A*	16 (03.5–24.5)	*wmc0667*	LY4	3.26	8.22	7.19
*QDSIGp*.*ccsu-6B*	17.5 (13.4–20.7)	*wPt-2786*	LY1	3.91	8.56	-0.14
*QDSIGp*.*ccsu-7B*	39.5 (36.4–42.1)	*barc0258*	LY4	3.19	6.38	6.31
**2. Days to anthesis**						
*QDSIDa*.*ccsu-3A*	47.1 (36.7–52.6)	*wPt-3816*	LY1	3.31	7.07	-0.19
*QDSIDa*.*ccsu-3D*	68.1 (57.8–70.1)	*gwm0003*	LY11	3.12	8.48	7.84
*QDSIDa*.*ccsu-7B*	39.5 (37.5–41.0)	*barc0258*	LY8	4.41	9.05	-0.34
**3. Days to maturity**						
*QDSIDm*.*ccsu-5A*.*1*	36.2 (22.8–47.6)	*wPt-0605*	LY6	2.17	3.58	0.43
*QDSIDm*.*ccsu-5A*.*2*	74.2 (70.1–79.3)	*Vrn-A1*	LY11	3.38	7.26	-0.22
*QDSIDm*.*ccsu-6B*	9.0 (03.0–12.9)	*wPt-7642*	LY9	4.29	10.85	-0.15
*QDSIDm*.*ccsu-7A*	48.6 (40.0–53.4)	*stm0511tctg*	LY11	3.46	6.68	0.21
**4. Grain filling duration**						
*QDSIGfd*.*ccsu-3B*	9.2 (08.1–10.1)	*wPt-8910*	LY4	3.23	6.6	0.25
*QDSIGfd*.*ccsu-4B*	0.0 (00.0–03.3)	*wPt-0037*	LY8	3.05	6.26	0.31
*QDSIGfd*.*ccsu-5A*	78.2 (71.4–82.9)	*cfa2141*	LY11	2.76	4.26	-0.23
*QDSIGfd*.*ccsu-6B*	83.5 (72.7–84.9)	*wPt-1325*	LY8	4.3	9.45	-0.31
**5. Plant height**						
*QDSIHt*.*ccsu-1B*	1.4 (00.0–07.4)	*wPt-2052*	LY9	3.08	6.62	-0.14
*QDSIHt*.*ccsu-3A*	0.0 (00.0–03.9)	*barc0294A*	LY2	3.04	6.51	1.21
*QDSIHt*.*ccsu-6B*.*1*	11.6 (10.8–13.7)	*wPt-4564*	LY4	5.41	12.01	0.12
*QDSIHt*.*ccsu-6B*.*2*	17.8 (17.4–19.9)	*wPt-5256*	LY4	5.16	11.7	0.1
**6. Productive tillers/m**^**2**^						
*QDSIPt*.*ccsu-1A*	36.7 (35.3–37.1)	*wPt-7030*	LY9	3.51	7.66	0.19
*QDSIPt*.*ccsu-7B*	0.0 (00.0–03.3)	*gwm0046*	LY5	2.99	6.57	0.17
**7. Grain weight/ear**						
*QDSIGwe*.*ccsu-7A*.*1*	38.2 (31.1–43.8)	*wmc0283*	LY8; LY12	2.46–3.91	5.43–7.95	-0.44 TO -1.07
**8. Thousand grain weight**						
*QDSITgw*.*ccsu-4A*	25.1 (22.6–33.1)	*barc0170*	LY9	3.31	7.26	-0.13
*QDSITgw*.*ccsu-4B*	3.0 (00.0–07.5)	*wPt-0037*	LY12	3.5	8.53	-0.81
*QDSITgw*.*ccsu-6B*	17.8 (13.6–21.9)	*wPt-5256*	LY12	2.13	4.69	0.5
*QDSITgw*.*ccsu-7A*.*1*	8.4 (05.2–12.8)	*gwm0681*	LY8	3.02	5.9	2.15
*QDSITgw*.*ccsu-7A*.*2*	38.2 (37.0–41.4)	*wmc0283*	LY8	7.19	16.9	-3.51
*QDSITgw*.*ccsu-7D*	29.8 (19.1–38.9)	*barc0092*	LY8	3.13	6.19	2.05
**9. Grain yield per plot**						
*QDSIGyp*.*ccsu-4A*	57.5 (54.8–60.1)	*wPt-0538*	LY7	4.56	9.93	-0.15
*QDSIGyp*.*ccsu-5A*.*1*	39.3 (35.5–46.2)	*wPt-7769*	LY10	2.72	5.79	2.77
*QDSIGyp*.*ccsu-5A*.*2*	29.9 (21.8–38.7)	*wPt-9887*	LY11	3.15	6.28	-0.15
*QDSIGyp*.*ccsu-5D*	38.9 (29.5–45.1)	*wPt-1400*	LY8	3.13	7.35	0.1
*QDSIGyp*.*ccsu-6B*	0.0 (00.0–03.3)	*wPt-0037*	LY9	4.12	9.03	-0.19

Location codes; LY1, Kanpur in year 2010–11; LY4, Pune in year 2010–11; LY5, Kanpur in year 2011–12; LY6, Karnal in year 2011–12; LY8, Pune in year 2011–12; LY9, Kanpur in year 2012–13; LY11, Hisar in year 2012–13; LY12, Pune in year 2012–13. Env.: Environments.

^a^ A positive value of the additive main effects (a) indicates that Kukri contributes allele to increase the phenotype value, and a negative value means that Excalibur provides allele to increase the phenotype value.

#### Multi-trait analysis (co-located QTL)

**Fig 7:**
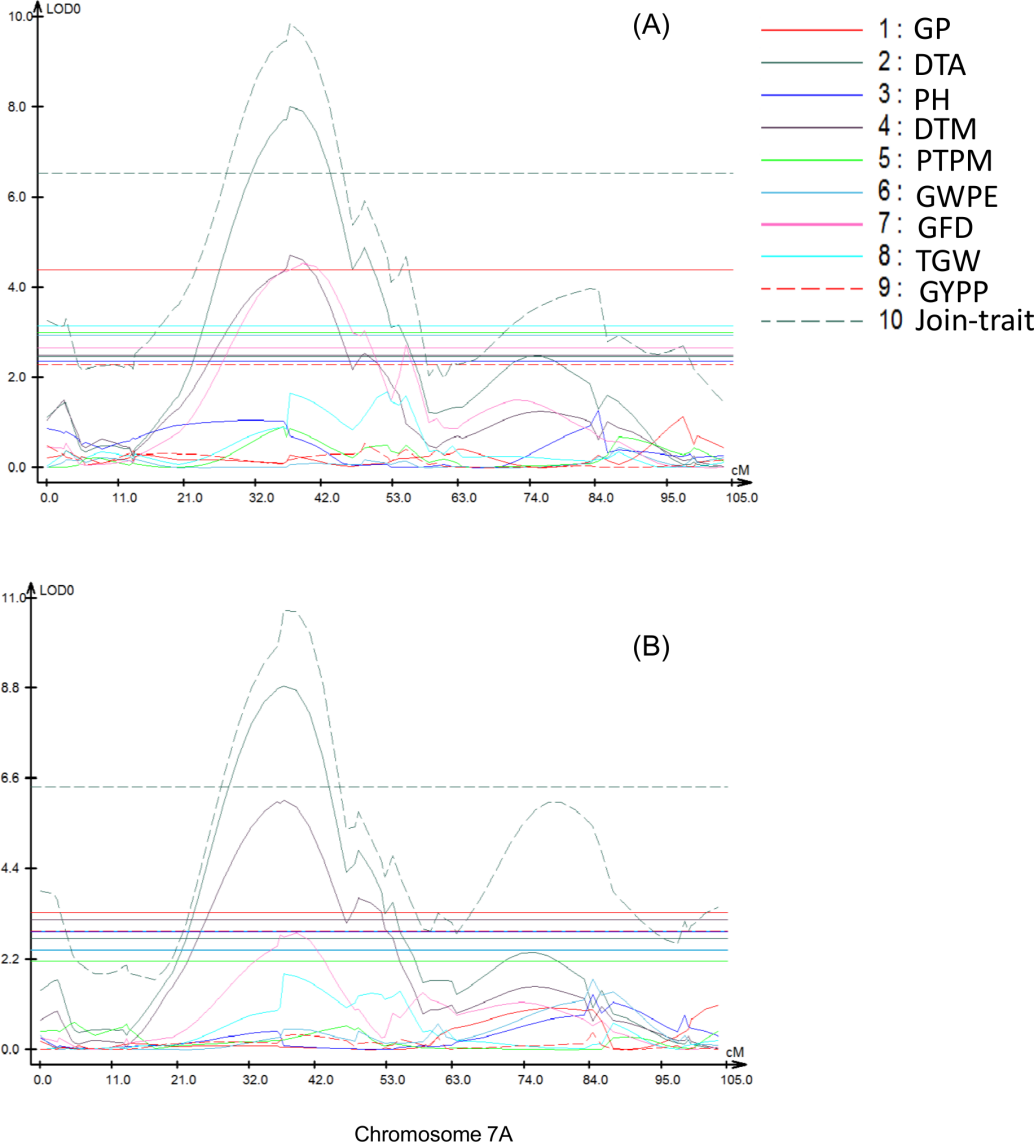
QTL cartographer plots showing a multi-trait QTL detected on chromosome 7A by multi-trait composite interval mapping (MCIM) using data pooled over IR and RF environments. (A) IR environment; (B) RF environment. GP, germination percentage; DTA, days to anthesis; DTM, days to maturity; GFD, grain filling duration; PH, plant height; PTPM, productive tillers/m^2^; GWPE, grain weight/ear; TGW, 1000 grain weight; GYPP, grain yield per plot.

For correlated traits, a solitary QTL on 7A was identified in multi-trait composite interval mapping (MCIM). Under IR conditions, this QTL was associated with three traits including DTA, DTM and GFD, while in RF conditions, it was associated with DTA and DTM (Fig 7).

### Two-locus analysis

Two-locus analysis was carried out to separate the components of genetic variation for each of the nine agronomic traits in terms of main-effect QTL (M-QTL), QTL × QTL (QQ) epistatic interactions and QTL × environment (QE) interactions.

#### Main effect QTL (M-QTL)

Using two-locus analysis, a total of 19 M-QTL were identified for only four traits (Table 5). These four traits included DTA (10 QTL), DTM (one QTL), GFD (two QTL) and TGW (six QTL). These QTL largely included QTL identified only in IR or RF conditions, and only four QTL were identified in both IR and RF conditions (3 QTL for DTA; one QTL for TGW). PVE values for all these QTL were rather low (0.33% to 5.29%). Out of the above 19 M-QTL, 8 M-QTL (5 for DTA, 1 each for DTM, GFD and TGW) were also identified in CIM analysis, making them more reliable QTL (Table 5).

**Table 5:** Main effect QTL detected by two-locus analysis with additive effects and additive × environments interactions (Q_A_×E) for DTA, DTM, and GFD in a Kukri/Excalibur DH mapping population.

QTL name		Marker (Position in cM)	A^a^	Q_A_ × E interaction (AE)^b^/h^2^ (%)				R^2^ (%)
				AE1	AE2	AE3	AE4	
**1. Days to anthesis**								
(i)	*QDa*.*ccsu-1A*	*wPt-0128 (78*.*7)*	-0.510	—	—	—	—	0.65
(ii)	*QDa*.*ccsu-1B*.1*	*barc0137 (22*.*8)*	0.719	—	—	—	—	1.50
(iii)	*QDa*.*ccsu-2A*.*1*	*gdm0093 (76*.*9)*	0.752	-0.70/1.23	—	—	0.69/1.15	1.41
(iv)	*QDa*.*ccsu-4A*.*2*	*wPt-2794 (60*.*7)*	1.374	-0.50/0.60	—	0.48/0.50	0.65/1.05	4.72
	***QDa*.*ccsu-4A*.*2***	*wPt-2794 (60*.*7)*	0.773	-0.41/0.48	—	—	0.73/1.53	1.74
(v)	*QDa*.*ccsu-4B*.2*	*wmc0349 (12*.*2)*	-0.868	0.80/1.87	—	-0.66/1.26	0.75/1.62	2.19
(vi)	*QDa*.*ccsu-5A*.1*	*wPt-9887 (21*.*9)*	-0.662	0.50/0.74	—	—	-0.95/2.60	1.28
(vii)	*QDa*.*ccsu-5A*.4*	*Vrn-A1 (70*.*2)*	1.116	-0.95/2.25	—	1.05/2.76	2.12/1.12	3.11
	*QDa*.*ccsu-5A*.4*	*Vrn-A1 (70*.*2)*	0.782	-0.89/2.30	—	0.93/2.53	1.93/0.79	1.78
(viii)	*QDa*.*ccsu-6A*	*wPt-8256 (6)*	-0.390	—	—	—	—	0.38
(ix)	***QDa*.*ccsu-6B*.*1***	*wPt-7662 (0)*	0.492	—	—	—	—	0.70
(x)	*QDa*.*ccsu-7A*.1*	*wmc0283 (37*.*2)*	-1.455	1.14/3.27	-0.58/0.83	-1.21/3.68	-1.35/4.53	5.29
	*QDa*.*ccsu-7A*.1*	*wmc0283 (37*.*2)*	-1.052	0.97/2.71	—	-1.00/2.89	-1.15/3.83	3.22
**2. Days to maturity**								
(i)	*QDm*.ccsu-7A*	*wmc0283* (37.2)	-1.176	0.95/0.86	—	—	—	1.32
**3. Grain filling duration**								
(i)	*QGfd*.*ccsu-5A*.*2*	*wPt-9834* (76.4)	-0.506	—	—	—	-0.88/2.25	0.84
(ii)	*QGfd*.ccsu-7A*	*wmc0283* (37.2)	0.613	—	—	—	—	1.23
**4. Thousand grain weight**								
(i)	*QTgw*.*ccsu-1B*.*2*	*wPt-1770* (66.6)	0.448	—	—	—	—	0.66
(ii)	***QTgw*.*ccsu-6A*.*1***	*wPt-7599* (11.1)	-0.384	—	—	—	—	0.47
(iii)	***QTgw*.*ccsu-6B*.*2***	*stm0564acag* (41.3)	0.508	—	—	—	—	0.83
(iv)	*QTgw*.*ccsu-6B*.*3*	*barc0024* (45.2)	0.333	—	0.50/0.81	—	—	0.36
(v)	*QTgw*.*ccsu-7A*.3*	*wmc0283* (37.2)	0.589	—	—	0.48/0.76	—	1.14
	*QTgw*.*ccsu-7A*.3*	*wmc0283* (37.2)	0.321	—	—	0.98/3.09	—	0.33
(vi)	*QTgw*.*ccsu-7B*.*2*	*wmc0517* (38.1)	0.454	—	—	—	—	0.68

A^a^, significant additive effects contributed by QTL mapped in the environments

^b^AE1, AE2, AE3, AE4, represent the additive effects of significant QTL× environment interactions in 4 locations (E1, Kanpur location pooled over years, E2, Karnal location pooled over years, E3, Hisar location pooled over years, E4 Pune location pooled over years)

*QTL were also identified in CIM analysis; QTL names in **bold font** indicates that the QTL was identified under rain-fed environment

#### Epistatic QTL (E-QTL)

As many as 38 E-QTL were involved in epistatic interactions involving only two traits (DTA and TGW). Out of these 38 E-QTL, 4 QTL also had main effects; the remaining 34 E-QTL did not have any main effects (Table 6). These 38 QTL were involved in 19 QQ epistatic interactions (Table 6). The PVE due to individual QQ interactions was rather low (0.24% to 1.36%).

**Table 6:** Epistatic QTL (QQ) and their interaction with environment (QQE) detected by two-locus analysis using Kukri/Excalibur DH mapping population.

QTL_i		QTL_j		AAij^a^	Q×Q×E interactions (AAEij)^b^/h^2^ (%)				h^2^ (AA) (%)
QTL name	Marker (position, cM)	QTL name	Marker (position, cM)		AAE1	AAE2	AAE3	AAE4	
**1. Days to anthesis**									
*QDa*.*ccsu-1A*.*1*	*gwm0558* (32.3)	*QDa*.*ccsu-5A*.*2*	*wPt-8226* (48.5)	0.32	—	—	—	—	0.30
*QDa*.*ccsu-1A*.*2*	*wPt-6455* (39.7)	*QDa*.*ccsu-3B*.*2*	*gwm0383* (39.8)	-0.37	0.42/0.51	0.57/0.96	-0.65/1.21	-1.30/4.94	0.40
*QDa*.*ccsu-1B*.*2*	*wPt-2526* (54.2)	*QDa*.*ccsu-1D*.*1*	*ksm0117D* (7.0)	0.68	-0.49/0.69	—	0.50/0.73	—	1.36
*QDa*.*ccsu-2A*.*2*	*barc1138* (8.6)	*QDa*.*ccsu-7A*.*2*	*wPt-7299* (55.0)	0.45	-0.77/1.73	—	0.62/1.11	0.99/2.62	0.60
*QDa*.*ccsu-3A*.*1*	*wPt-7992* (4.6)	*QDa*.*ccsu-7B*	*wPt-2305* (13.9)	-0.33	0.58/0.96	-0.52/0.80	—	—	0.31
*QDa*.*ccsu-3A*.*2*	*stm0018tgag* (31.2)	*QDa*.*ccsu-3B*.*3*	*gwm0108-3B* (45.0)	0.63	-0.58/0.96	—	0.72/1.52	—	1.15
*QDa*.*ccsu-4A*.*1*	*wPt-7001* (6.7)	*QDa*.*ccsu-5A*.*4*	*Vrn-A1* (70.2)	0.59	-0.37/0.40	—	—	—	1.01
*QDa*.*ccsu-4D*	*wmc0457* (2)	*QDa*.*ccsu-5B*	*barc0112* (8.1)	0.63	—	—	—	—	1.15
*QDa*.*ccsu-7D*	*wPt-5049 (0*.*5)*	*QDa*.*ccsu-7D*.*2*	*cfd0021D (41*.*9)*	0.54	-0.37/0.40	—	—	—	0.84
**2. Thousand grain weight**									
*QTgw*.*ccsu-2B*.*1*	*wPt-4997* (43.7)	*QTgw*.*ccsu-2D*	*gwm0102* (0)	-0.45	—	—	—	—	0.65
*QTgw*.*ccsu-2B*.*2*	*wPt-7404* (70.6)	*QTgw*.*ccsu-7A*.*4*	*gwm0276* (63.6)	0.27	—	—	0.60/1.18	—	0.24
*QTgw*.*ccsu-3A*	*cfa2170* (59.3)	*QTgw*.*ccsu-3B*.*1*	*wPt-0302* (8.4)	0.50	—	—	—	—	0.82
***QTgw*.*ccsu-3A***	*cfa2170* (59.3)	*QTgw*.*ccsu-3B*.*1*	*wPt-0302* (8.4)	0.46	—	—	—	—	0.67
*QTgw*.*ccsu-3B*.*2*	*wPt-3107* (42.1)	*QTgw*.*ccsu-7A*.*1*	*wPt-0744* (2.6)	0.48	—	—	—	—	0.75
*QTgw*.*ccsu-3D*	*cfd0223* (45.7)	*QTgw*.*ccsu-7B*.*1*	*gwm0046* (0.0)	-0.50	—	—	—	—	0.81
***QTgw*.*ccsu-3D***	*cfd0223* (45.7)	*QTgw*.*ccsu-7B*.*1*	*gwm0046* (0.0)	-0.43	—	—	-0.47/0.71	—	0.59
*QTgw*.*ccsu-4A*.*3*	*wPt-0610* (63.2)	*QTgw*.*ccsu-6B*.*3*	*barc0024* (45.2)	—	—	—	0.58/1.12	-0.70/1.59	
*QTgw*.*ccsu-4A*.*4*	*wPt-9675* (68.2)	*QTgw*.*ccsu-7D*.*1*	*wPt-0789* (43.6)	-0.26	0.62/1.25	-0.59/1.15	-0.62/1.24	—	0.22
***QTgw*.*ccsu-4A*.*2***	*wPt-2983* (59.0)	*QTgw*.*ccsu-7D*.*2*	*stm0789tcacD* (47)	*-0*.*51*	—	-0.59/1.13	-0.76/1.84	—	0.83
***QTgw*.*ccsu-4A*.*5***	*wPt-2951* (68.8)	*QTgw*.*ccsu-6B*.*2*	*stm0564acag* (41.3)	*0*.*47*	—	—	—	—	0.72
*QTgw*.*ccsu-7A*.*3*	*wmc0283* (37.2)	*QTgw*.*ccsu-7B*.*3*	*wPt-7413* (64.1)	*0*.*51*	—	—	0.671.49	—	0.85

^a^AAij, additive effects of QxQ interaction.

^b^AAE1, AAE2, AAE3, AAE4, represent the additive effects of QxQxE1, QxQxE2, QxQxE3, and QxQxE4, respectively; where, E1, Kanpur location pooled over years, E2, Karnal location pooled over years, E3, Hisar location pooled over years, E4 Pune location pooled over years. QTL_i and QTL_j are a pair of QTL involved in epistasis.

QTL names in **bold font** indicates that the QTL was identified under rain-fed environment

### QTL× environmental interactions (QE and QQE)

As many as 10 QTL exhibited QE interactions in 1 to 4 environments (Table 5). These QTL involved only four traits (6 QTL for DTA, 1 QTL each for DTM and GFD, and 2 QTL for TGW). As many as 13 pairs of QTL (7 for DTA and 6 for TGW) also exhibited significant QQE interactions in 1 to 4 environments (Table 6).

## Discussion

The development of new drought tolerant wheat varieties and improvement of the existing high yielding drought-sensitive elite wheat varieties for drought tolerance is a priority area of research [38]. During the last two decades, QTL analysis has been extensively used to identify QTL associated with a number of complex traits that are known to be associated with drought tolerance in wheat; these traits also include yield under drought and/or heat-stress [16,17,20,21]. However, only few QTL have been utilized in molecular breeding and none cloned so far [6,11,38,39].

### QTL for specific environments (IR and RF)

It may be recalled that each of the 34 QTL detected in IR environments only, and the 23 QTL detected in RF environments only, figured in only one or two of the 22 environments used in the present study. These QTL also include QTL that were detected using pooled data. Similar results were reported in two earlier studies [20, 26]. In one of these two earlier studies, RIL mapping population derived from Seri M82/Babax was used and QTL analysis was conducted for 13 traits using data recorded in six environments (irrigated and restricted irrigation for inducing terminal drought stress); QTL for 7 of the 13 traits were reported in only one or two environments [20]. The results reported in the second study conducted by Shukla et al. [26] were no different. They used an RIL mapping population derived from WL711/C306, and reported QTL for 11 traits, each in one or two of the six environments (irrigated and limited irrigation). These results of two earlier studies are similar to those obtained in the present study and suggest a significant role of genotype x environment interaction in expression of QTL in varying environments, even though the magnitude of Q x E interactions for individual QTL may be low. Identification of fewer QTL in the RF environments may also be attributed to poor heritability of some of the traits in these environments, because the power of QTL detection is known to depend partly on the level of heritability of the trait as demonstrated in two earlier studies on GWAS in wheat [40,41].

### Major QTL under IR conditions

Of the above 34 QTL for IR environment, following four major QTL, each had >10% PVE and therefore deserve special attention: *QHt*.*ccsu*.*2B*, *QGfd*.*ccsu-5A*, *QPtm*.*ccsu-7A* and *QHt*.*ccsu*.*7D*. Of these 4 QTL, a major QTL (*QGfd*.*ccsu-5A* for GFD) was located on chromosome 5A and associated with *VERNALIZATION-A1* (*Vrn-A1*) gene. However, the DH population used for discovering the above QTL was developed by crossing two spring wheat genotypes, so that the *Vrn-A1* gene as such may not have any major effect on phenology, although marker associated with *Vrn-A1* gene may still be utilized in MAS for selection of the associated QTL. This QTL has a confidence interval (CI) of ~10 cM and overlaps the CIs for four other QTL for four different traits (see Table 3 and Fig 4). Therefore, this region on chromosome 5A seems to be important for developing wheat varieties for the IR environment.

From the remaining three of the above four major QTL for IR environment, two QTL (*QHt*.*ccsu*.*2B* and *QHt*.*ccsu*.*7D*) for PH were located on chromosomes, on which QTL for PH were also reported earlier [20,42–48]. The QTL for PH on 7D reported in the present study seems to be novel, since it is located in the middle of the chromosome, while that reported in the earlier study was located in the terminal position of the same chromosome [47]. The other QTL for PH on 2B reported in the present study could be the same as the QTL reported in the earlier study [49], since both were located in a genomic region in the middle of chromosome 2B (at 52.2 cM and 50 cM respectively). Due to unavailability of common markers between the two studies, the QTL in two studies can not be compared with precision. The remaining one major QTL (*QPtm*.*ccsu-7A*) for PTPM that was associated with SSR marker *wmc0283* also seems to be novel, since in earlier studies, no QTL for this trait was ever reported on 7A. These three QTL on 2B, 7A and 7D may be useful for MARS (for QTL on 7A, also see later).

### Major QTL under RF conditions

It may be recalled that 23 QTL were detected under RF conditions only; of these, three QTL were major QTL, one each for DTA (*QDa*.*ccsu-5A*.*1*), PH (*QHt*.*ccsu-5A*) and TGW (*QTgw*.*ccsu-7A*). The QTL for DTA and PH on 5A may both be novel, since QTL for DTA in all earlier studies were located on chromosomes other than 5A (2D, 3B and 7A; [26,27,50], while QTL for PH in two studies are located on the opposite ends of the same chromosome ([49]; for more details see review by Gupta et al. [6]). The third major QTL (*QTgw*.*ccsu-7A*) for TGW that was associated with SSR marker *wmc0283* could, however, be declared to be the same as *QTgw-7A*, which is one of the two important QTL (*QTgw*.*aww*.*7A* and *QTgw-7A*) for TGW reported earlier on the same chromosome [24,28]. Since both are associated with the same marker, these can be declared to be the same with higher level of confidence.

In several earlier studies also, 14 major QTL, for several drought related traits (data recorded under water stress conditions) were reported; these traits included grain yield (4A, 3B, 7A), TGW (3B, 7D), DTH (2A, 7D), DTM (7D), stem reserve mobilisation i.e. SRM (2D, 5D, 7D), water soluble carbohydrates i.e. WSC (3A) and chlorophyll content on 3B (S7 Table). Two of the above QTL (QTL on 4A associated with marker *Xwmc 420* and QTL *Qyld*.*csdh*.*7AL* associated with marker *wmc322*) for grain yield also mapped with meta-QTL for drought and heat stress [21]. However, such QTL that are specific to RF condition should be validated and tested for their robustness and PVE contribution in other genotypes and then used for MAS or MARS in breeding programs for developing wheat varieties suitable for rain-fed conditions.

Taken together, only three major QTL with PVE of >10% for three traits (PH, DTA and TGW) were available, which presumably expressed only under drought stress. In earlier QTL studies involving drought tolerance, ~700 QTL have so far been reported, but only 14 of these QTL for agronomic and physiological traits were consistent with a PVE of >20% (for details see Gupta et al. [6]). Therefore, we propose that while breeding for drought tolerance, the QTL specific for drought stress, reported during the present study and those reported in earlier studies may be pyramided following marker-assisted recurrent selection (MARS). Alternatively, with the progress in next-generations sequencing (giving large number of SNP markers) and the statistical resources, genomic selection (GS) can be another option for developing drought tolerant wheat cultivars.

### Major and stable QTL with wider adaptation (detected in both IR + RF)

A number of QTL were detected in IR as well as RF conditions; five such QTL (*QDa*.*ccsu-5A*.*2*, *QDm*.*ccsu-5A*.*2*, *QDa*.*ccsu-7A*, *QDm*.*ccsu-7A* and *QGfd*.*ccsu-7A*) were major QTL (PVE ~20%); four of these QTL (except *QGfd*.*ccsu-7A*) were identified in multiple environments ranging in number from 4 to 10. These QTL were considered to be relatively stable QTL and were therefore important (for details of these QTL with linked markers, see Table 7). The QTL for DTA (*QDa*.*ccsu-5A*.*2*) was also co-located with QTL for DTM and GFD linked with a gene specific marker *Vrn-A1*. The other three QTL (*QDa*.*ccsu-7A* and *QDm*.*ccsu-7A* and *QGfd*.*ccsu-7A*) on chromosome 7A were co-located with QTL for PTPM linked with a SSR marker *wmc0283*. The PTMP also had positive association with GYPP in the RF environment. Therefore, these four QTL can be used for breeding for wide adaptation and high yield under environments with variable soil moisture including the RF environments (see later).

**Table 7:** Details of major and stable QTL for different traits identified during the present study using Kukri/Excalibur DH mapping population.

QTL (PVE >10%)	Linked marker	Favourable allele
**(a) Identified in IR environments only**		
1. *QHt*.*ccsu*.*2B*	*wPt-9423*	Excalibur
2. *QGfd*.*ccsu-5A*	*wmc0075*	Excalibur
3. *QPtm*.*ccsu-7A*	*wmc0283*	Excalibur
4. *QHt*.*ccsu*.*7D*	*barc0092*	Kukri
**(b) Identified in RF environments only**		
1. *QHt*.*ccsu-5A*	*wPt-0373*	Kukri
2. *QDa*.*ccsu-5A*.*1*	*wPt-9887*	Kukri
3.*QTgw*.*ccsu7A*	*wmc0283*	Excalibur
**(c) Identified in both (IR and RF) environments**		
1. *QDa*.*ccsu-5A*.2*	*Vrn-A1*	Kukri
2. *QDm*.*ccsu-5A*.2*	*Vrn-A1*	Kukri
3. *QDm*.ccsu-7A*	*wmc0283*	Excalibur
4. *QDa*.ccsu-7A*	*wmc0283*	Excalibur
5. *QGfd*.*ccsu-7A*	*wmc0283*	Excalibur

*Stable QTL (identified in a minimum of 4 and a maximum of 10 environments)

### Important QTL on chromosomes 5A and 7A

Chromosomes 5A and 7A carry important QTL for some important traits and therefore deserve special attention. For instance, chromosome 5A carries *QDm*.*ccsu-5A*.*2* for DTM and *QDa*.*ccsu-5A*.*2* for DTA, both associated with *Vrn-A1* gene. QTL affecting DSI for DTM (*QDSIDm*.*ccsu-5A*.*2*) was also identified in the same region. A meta-QTL (M-QTL4) for yield and related traits was also reported in the proximity of *Vrn-A1* [51], suggesting the importance of this particular genomic region for traits like DTM, grain yield and other related traits. Beside the above genomic region, other genomic regions harbouring QTL for GP, PH, GFD, PTPM and GWPE on 5A were also identified during the present study (Table 3).

QTL on chromosome 5A for a number of traits including DTA and DTM were also reported in earlier studies, although these QTL reported earlier did not map in the same region, where the above two QTL identified in the present study are located. These earlier reported QTL included QTL for yield, plant height, tiller number, ear compactness, spike length, DTA, DTM and canopy temperature (CT) at vegetative stage (for details of references and the information therein, see review by Gupta et al. [6]). This suggested that more than one regions on 5A may be important for drought tolerance, so that their functional analysis will provide important information on the genetic architecture of the above traits.

Chromosome 7A also carries QTL for the following seven traits: DTA, DTM, GFD, PH, PTPM, GWPE and TGW (Table 3). Three co-localized QTL, one each for DTA (*QDa*.*ccsu-7A*), DTM (*QDm*.*ccsu-7A*) and TGW (*QTgw*.*ccsu-7A*) were linked with the SSR marker *wmc0283*. These QTL explained 16.6%- 20.43% PV, which are the highest values of PVE in an individual environment. In earlier studies also, a QTL for grain yield (*Qyld*.*csdh*.*7AS*.*2*) under drought was reported in the same genomic region [50,52]. These QTL on chromosome 7A also coincided with a meta-QTL (M-QTL) for drought/heat stress [21]. The position of this M-QTL coincides with the position of QTL for the following eight physiological and agronomic traits contributing to drought adaptation: (i) biomass, (ii) canopy temperature, (iii) kernel number, (iv) days to maturity, (v) stay-green, (vi) TGW, (vii) WSC and (viii) yield. Interestingly, the gene *TaTEF-7A*, a member of the transcript elongation factor gene family, was also mapped at a distance of 11.9 cM from *wmc0283* linked to the above QTL [53]. This gene has the highest expression in both, the young spikes and developing seeds and is associated with grain number per spike, grain yield and other yield-related traits [53].

The importance of chromosome 7A was also evident from multi-trait CIM (MCIM), which identified regions on 7A with QTL for several important traits including GP, DTA, DTM, GFD, PH, PTPM, GWPE and TGW. Of these traits, GFD deserves special attention since grain-filling period is known to be positively related with grain yield in wheat [54]. Significant genetic variation for GFD has also been reported in wheat, making GFD a suitable target for breeding under drought stress [5,55,56]. The above genomic regions have also been implicated in controlling spike architecture, grains per spike, grain yield, yield-related traits and hormone metabolism in other studies [20,43,47,50,52]. A QTL affecting DSI for GWPE (*QDSIGwe*.*ccsu-7A*) was also located on 7A. Thus, one or more specific regions of chromosome 7A may prove useful for MARS, while breeding wheat for adaptation, so that the improved cultivars may be suitable for cultivation under both environments (IR and RF).

### Use of QTL for breeding

As discussed above, the major QTL identified during the present study and those identified in earlier studies can be used for breeding varieties, which would be tolerant to water-stress conditions, and also those which can be grown under both IR and RF conditions. The three major QTL identified only under RF condition, and the four major QTL identified only under IR conditions were unfortunately not consistent. Therefore, their utility may be limited. More important QTL, however, are those five major QTL, which were located on chromosomes 5A and 7A and were detected both under IR and RF conditions; these QTL were also consistent (detected in up to 4–5 RF and 2–5 IR conditions).

The desirable alleles for 5A QTL belonged to the parent Kukri, which is drought sensitive, and the desirable alleles of 7A QTL (associated with SSR marker *wmc0283*) belonged to Excalibur, which is drought tolerant. Therefore, both the cultivars i.e. Kukri and Excalibur along with other drought tolerant genotypes (to be identified) may be used as donors for mobilization and pyramiding of desirable QTL alleles into one or more drought sensitive elite cultivars.

Interestingly, positive and significant correlations of GYPP were observed with five traits (GP, PH, PTPM, GWPE and TGW) in RF environment and with two traits (GWPE and TGW) in IR environment. Such correlations may be either due to pleiotropic QTL or due to close proximity of QTL in the same genomic regions [20,26,47,49]. However, during the present study, the genomic regions harbouring QTL for the above correlated traits did not carry QTL for GYPP, which is in agreement with the findings of some earlier studies [19,56]. This observation can be attributed to significant but poor correlation of GYPP with the above mentioned traits both in the IR (r = 0.157 to 0.392) and RF (r = 0.282 to 0.344) environments, Unfortunately, therefore, the major QTL for the above mentioned correlated traits may not be useful in MAS for indirect improvement in GYPP.

Some useful QTL have also been reported in earlier studies (e.g., *Qyld*.*csdh*.*7AS*.*2*). In addition to these, 14 other useful QTL are known from earlier studies and may be used for developing drought resilient cultivars (S7 Table; also see Gupta et al. [6]). It is thus obvious that a number of desirable QTL for drought tolerance are now available, so that a breeding programme involving MARS for deployment of about a dozen QTL can now be implemented for developing drought resilient wheat cultivars.

### Two locus analysis

QQ (epistasis) and QQE (epistasis × environment) interactions sometimes make an important component of the genetics of stress adaptive responses [57]. In several earlier QTL mapping studies in wheat, however, these interactions (QQ, QE, and QQE) were not examined [19,20,24,42,46,52,58–63]. During the present study, when epistatic interactions were examined, surprisingly E-QTL for only two traits (DTA and TGW) were available; also, PVE due to these E-QTL was rather low (0.24% to 1.36%; Table 6). Similar results were also reported in two earlier studies for response to drought stress [26,27]. In one of these studies involving 10 traits, Kadam et al. [27] reported only three epistatic (QQ) interactions for two traits (one QTL for shoot biomass and two QTL for days to flowering) only; the PVE was also rather low (0.56% to 1.06%). In the other study involving 11 traits, Shukla et al. [26] reported only three epistatic (QQ) interactions for three traits (one each for grain yield, TGW and days to flowering); the value of PVE was low in this study also (0.27% to 0.64%). Similarly, Kumar et al. [64] found that epistasis was not statistically important compared to the main additive effects. Relatively low contribution of epistasis in the total phenotypic variation may also be attributed to low power of the statistical tests [65,66].

## Summary and conclusions

Although several QTL for different drought related agronomic traits were identified in IR and RF environments, these included only few major QTL. It was also observed that specific genomic regions of chromosomes 5A and 7A were important for wheat improvement for drought tolerance through MARS. In particular, the following QTL on 5A can be exploited for developing drought tolerant cultivars: (*i*) Two QTL for DTA, one each linked with *wPt-9887* and *Vrn-A1*; (*ii*) a QTL for PH linked with *wPt-0373*; (*iii*) a QTL for DSI for DTM associated with the *Vrn-A1* and co-located with the QTL for DTA. A QTL hotspot on chromosome 7A was also considered to be important, since it contains QTL for DTA, DTM, GFD, PTPM and TGW together with a QTL affecting DSI for GWPE linked with *wmc0283*. The information generated during the present study also represents a rich resource for further investigations and for annotation of relevant genomic regions/genes using the available wheat genomic resources. This will allow development of additional markers for MARS and will also facilitate development of a better understanding of the genetic architecture that controls drought tolerance in wheat.

## Supporting information

S1 Fig Minimum and maximum temperature during the crop growth period for three crop seasons (2010–11 to 2012–13) at four locations in India. (A) Kanpur, (B) Karnal, (C) Pune, and (D) Hisar.
(PDF)

S1 Table Total water applied through irrigation to the Kukri/Excalibur DH mapping population during the 2010–2013 crop cycles in different environments. (PDF)

S2 Table Raw phenotype data for nine agronomic traits measured on the Kukri/Excalibur DH mapping population in 22 different environments. (PDF)

S3 Table Descriptive statistics (mean, SE, range, and CV %) for nine (9) important agronomic traits measured on the Kukri/Excalibur DH mapping population in 22 different environments. (PDF)

S4 Table Analysis of variance (ANOVA) for nine agronomic traits measured on the Kukri/Excalibur DH mapping population in 22 different environments. (PDF)

S5 Table Descriptive statistics (mean, SE, and range) for DSI of nine (9) important agronomic traits measured on the Kukri/Excalibur DH mapping population in 12 different environments. (PDF)

S6 Table Number of markers per chromosome and the length of individual linkage groups of Kukri × Excalibur DH mapping population. (PDF)

S7 Table List of major QTL (PVE >20%) for seven drought-responsive traits identified in earlier studies along with their linked markers, PVE, mapping population type, and parental genotypes. (PDF)

## References

[1] Shiferaw B, Smale M, Braun H-J, Duveiller E, Reynolds M, Muricho G. Crops that feed the world 10. Past successes and future challenges to the role played by wheat in global food security. Food Secur. 2013;5: 291–317. 10.1007/s12571-013-0263-y

[2] Parry MAJ, Reynolds M, Salvucci ME, Raines C, Andralojc PJ, Zhu X-G, et al Raising yield potential of wheat. II. Increasing photosynthetic capacity and efficiency. J Exp Bot. 2011;62: 453–467. 10.1093/jxb/erq304 10.1093/jxb/erq304 21030385

[3] Ray DK, Ramankutty N, Mueller ND, West PC, Foley JA. Recent patterns of crop yield growth and stagnation. Nat Commun. Nature Publishing Group, a division of Macmillan Publishers Limited. All Rights Reserved.; 2012;3: 1293 Available: 10.1038/ncomms2296 23250423

[4] Araus JL, Slafer GA, Royo C, Serret MD. Breeding for Yield Potential and Stress Adaptation in Cereals. CRC Crit Rev Plant Sci. 2008;27: 377–412. 10.1080/07352680802467736

[5] Farooq M, Hussain M, Siddique KHM. Drought Stress in Wheat during Flowering and Grain-filling Periods. CRC Crit Rev Plant Sci. Taylor & Francis; 2014;33: 331–349. 10.1080/07352689.2014.875291

[6] Gupta P, Balyan H, Gahlaut V. QTL Analysis for Drought Tolerance in Wheat: Present Status and Future Possibilities. Agronomy. 2017;7: 5. 10.3390/agronomy7010005

[7] Turner NC, Blum A, Cakir M, Steduto P, Tuberosa R, Young N. Strategies to increase the yield and yield stability of crops under drought–are we making progress? Funct Plant Biol. 2014;41: 1199–1206. Available: 10.1071/FP14057 32481069

[8] Portmann FT, Siebert S, Döll P. MIRCA2000—Global monthly irrigated and rainfed crop areas around the year 2000: A new high-resolution data set for agricultural and hydrological modeling. Global Biogeochem Cycles. 2010;24: n/a-n/a. 10.1029/2008GB003435

[9] Hadiarto T, Tran L-SP. Progress studies of drought-responsive genes in rice. Plant Cell Rep. 2011;30: 297–310. 10.1007/s00299-010-0956-z 10.1007/s00299-010-0956-z 21132431

[10] Reynolds M, Bonnett D, Chapman SC, Furbank RT, Manès Y, Mather DE, et al Raising yield potential of wheat. I. Overview of a consortium approach and breeding strategies. J Exp Bot. 2011;62: 439–452. Available: 10.1093/jxb/erq311 20952629

[11] Gupta PK, Balyan HS, Gahlaut V, Kulwal PL. Phenotyping, Genetic Dissection, and Breeding for Drought and Heat Tolerance in Common Wheat: Status and Prospects. Plant Breeding Reviews. John Wiley & Sons, Inc.; 2012 pp. 85–168. 10.1002/9781118358566.ch2

[12] Jogaiah S, Govind SR, Tran L-SP. Systems biology-based approaches toward understanding drought tolerance in food crops. Crit Rev Biotechnol. Taylor & Francis; 2013;33: 23–39. 10.3109/07388551.2012.659174 10.3109/07388551.2012.659174 22364373

[13] Blum A. Plant breeding for stress environments [Internet]. Boca Raton, FL: CRC Press, Inc.; 1988 Available: https://www.cabdirect.org/cabdirect/abstract/19901610549

[14] Langridge P, Reynolds MP. Genomic tools to assist breeding for drought tolerance. Curr Opin Biotechnol. 2015;32: 130–135. 10.1016/j.copbio.2014.11.027 25531270

[15] Passioura JB. Phenotyping for drought tolerance in grain crops: when is it useful to breeders? Funct Plant Biol. 2012;39: 851–859. Available: 10.1071/FP12079 32480835

[16] Ain Q, Rasheed A, Anwar A, Mahmood T, Imtiaz M, Mahmood T, et al Genome-wide association for grain yield under rainfed conditions in historical wheat cultivars from Pakistan. Front Plant Sci. Frontiers Media S.A.; 2015;6: 743 10.3389/fpls.2015.00743 10.3389/fpls.2015.00743 26442056 PMC4585131

[17] Edae E a., Byrne PF, Manmathan H, Haley SD, Moragues M, Lopes MS, et al Association Mapping and Nucleotide Sequence Variation in Five Drought Tolerance Candidate Genes in Spring Wheat. Plant Gen. 2013;6. 10.3835/plantgenome2013.04.0010

[18] Mason RE, Mondal S, Beecher FW, Pacheco A, Jampala B, Ibrahim AMH, et al QTL associated with heat susceptibility index in wheat (Triticum aestivum L.) under short-term reproductive stage heat stress. Euphytica. 2010;174: 423–436. 10.1007/s10681-010-0151-x

[19] McIntyre CL, Mathews KL, Rattey A, Chapman SC, Drenth J, Ghaderi M, et al Molecular detection of genomic regions associated with grain yield and yield-related components in an elite bread wheat cross evaluated under irrigated and rainfed conditions. Theor Appl Genet. 2010;120: 527–541. 10.1007/s00122-009-1173-4 10.1007/s00122-009-1173-4 19865806

[20] Pinto RS, Reynolds MP, Mathews KL, McIntyre CL, Olivares-Villegas J-J, Chapman SC. Heat and drought adaptive QTL in a wheat population designed to minimize confounding agronomic effects. Theor Appl Genet. 2010;121: 1001–1021. 10.1007/s00122-010-1351-4 10.1007/s00122-010-1351-4 20523964 PMC2938441

[21] Acuna-Galindo MA, Mason RE, Subramanian NK, Hays DB. Meta-analysis of wheat QTL regions associated with adaptation to drought and heat stress. Crop Sci. 2015;55: 477–492. 10.2135/cropsci2013.11.0793

[22] Alexander LM, Kirigwi FM, Fritz AK, Fellers JP. Mapping and Quantitative Trait Loci Analysis of Drought Tolerance in a Spring Wheat Population Using Amplified Fragment Length Polymorphism and Diversity Array Technology Markers. Crop Sci. Madison, WI: The Crop Science Society of America, Inc.; 2012;52: 253–261. 10.2135/cropsci2011.05.0267

[23] Bennett D, Izanloo A, Edwards J, Kuchel H, Chalmers K, Tester M, et al Identification of novel quantitative trait loci for days to ear emergence and flag leaf glaucousness in a bread wheat (Triticum aestivum L.) population adapted to southern Australian conditions. Theor Appl Genet. 2012;124: 697–711. 10.1007/s00122-011-1740-3 10.1007/s00122-011-1740-3 22045047

[24] Bennett D, Reynolds M, Mullan D, Izanloo A, Kuchel H, Langridge P, et al Detection of two major grain yield QTL in bread wheat (Triticum aestivum L.) under heat, drought and high yield potential environments. Theor Appl Genet. 2012;125: 1473–1485. 10.1007/s00122-012-1927-2 10.1007/s00122-012-1927-2 22772727

[25] Kuchel H, Williams KJ, Langridge P, Eagles HA, Jefferies SP. Genetic dissection of grain yield in bread wheat. I. QTL analysis. Theor Appl Genet. 2007;115: 1029–1041. 10.1007/s00122-007-0629-7 10.1007/s00122-007-0629-7 17713755

[26] Shukla S, Singh K, Patil R V, Kadam S, Bharti S, Prasad P, et al Genomic regions associated with grain yield under drought stress in wheat (Triticum aestivum L.). Euphytica. 2015;203: 449–467. 10.1007/s10681-014-1314-y

[27] Kadam S, Singh K, Shukla S, Goel S, Vikram P, Pawar V. Genomic associations for drought tolerance on the short arm of wheat chromosome 4B. Funct Integr Genomics. 2012;12. 10.1007/s10142-012-0276-1 10.1007/s10142-012-0276-1 22476619

[28] Hill CB, Taylor JD, Edwards J, Mather D, Bacic A, Langridge P, et al Whole-genome mapping of agronomic and metabolic traits to identify novel quantitative trait Loci in bread wheat grown in a water-limited environment. Plant Physiol. 2013;162: 1266–1281. 10.1104/pp.113.217851 10.1104/pp.113.217851 23660834 PMC3707548

[29] Fischer RA, Maurer R. Drought resistance in spring wheat cultivars. I. Grain yield responses. Aust J Agric Res. 1978;29: 897–912.

[30] Merk HL, Yarnes SC, Van Deynze A, Tong N, Menda N, Mueller LA, et al Trait Diversity and Potential for Selection Indices Based on Variation Among Regionally Adapted Processing Tomato Germplasm. J Am Soc Hortic Sci. 2012;137: 427–437. Available: http://journal.ashspublications.org/content/137/6/427.abstract

[31] Edwards J. A genetic analysis of drought related traits in hexaploid wheat. University of Adelaide 2012.

[32] Lander ES, Green P, Abrahamson J, Barlow A, Daly MJ, Lincoln SE, et al MAPMAKER: An interactive computer package for constructing primary genetic linkage maps of experimental and natural populations. Genomics. 1987;1: 174–181. 10.1016/0888-7543(87)90010-3 3692487

[33] Kosambi DD. The estimation of map distances from recombination values. Ann Eugen. Blackwell Publishing Ltd; 1943;12: 172–175. 10.1111/j.1469-1809.1943.tb02321.x

[34] Voorrips RE. MapChart: Software for the Graphical Presentation of Linkage Maps and QTLs. J Hered. 2002;93: 77–78. Available: 10.1093/jhered/93.1.77 12011185

[35] Wang S, Basten CJ, Zeng ZB. Windows QTL Cartographer 2.5. In: Department of Statistics, North Carolina State University, Raleigh, NC [Internet]. 2012 Available: http://statgen.ncsu.edu/qtlcart/WQTLCart.htm

[36] Yang J, Hu C, Hu H, Yu R, Xia Z, Ye X, et al QTLNetwork: mapping and visualizing genetic architecture of complex traits in experimental populations. Bioinformatics. 2008;24: 721–723. Available: 10.1093/bioinformatics/btm494 18202029

[37] McIntosh RA, Yamazaki Y, Dubcovsky J, Rogers J, Morris C, Appels R, et al. Catalogue of Gene Symbols for Wheat. 12th International Wheat Genetics Symposium. Yokohama, Japan; 2013.

[38] Fleury D, Jefferies S, Kuchel H, Langridge P. Genetic and genomic tools to improve drought tolerance in wheat. J Exp Bot. 2010;61: 3211–3222. Available: 10.1093/jxb/erq152 20525798

[39] Reynolds M, Tuberosa R. Translational research impacting on crop productivity in drought-prone environments. Curr Opin Plant Biol. 2008;11: 171–179. 10.1016/j.pbi.2008.02.005 18329330

[40] Mwadzingeni L, Shimelis H, Rees DJG, Tsilo TJ. Genome-wide association analysis of agronomic traits in wheat under drought-stressed and non-stressed conditions. PLoS One. Public Library of Science; 2017;12: e0171692 Available: http://dx.doi.org/10.1371%2Fjournal.pone.0171692 28234945 10.1371/journal.pone.0171692 PMC5325217

[41] Laidò G, Marone D, Russo MA, Colecchia SA, Mastrangelo AM, De Vita P, et al Linkage Disequilibrium and Genome-Wide Association Mapping in Tetraploid Wheat (Triticum turgidum L.). PLoS One. Public Library of Science; 2014;9: e95211 Available: http://dx.doi.org/10.1371%2Fjournal.pone.0095211 24759998 10.1371/journal.pone.0095211 PMC3997356

[42] Marza F, Bai G-H, Carver BF, Zhou W-C. Quantitative trait loci for yield and related traits in the wheat population Ning7840 × Clark. Theor Appl Genet. 2006;112: 688–698. 10.1007/s00122-005-0172-3 10.1007/s00122-005-0172-3 16369760

[43] Mathews KL, Malosetti M, Chapman S, McIntyre L, Reynolds M, Shorter R, et al Multi-environment QTL mixed models for drought stress adaptation in wheat. Theor Appl Genet. 2008;117: 1077–1091. 10.1007/s00122-008-0846-8 10.1007/s00122-008-0846-8 18696042

[44] Maccaferri M, Sanguineti MC, Corneti S, Ortega JLA, Salem M Ben, Bort J, et al Quantitative trait loci for grain yield and adaptation of durum wheat (Triticum durum Desf.) across a wide range of water availability. Genetics. 2008;178: 489–511. 10.1534/genetics.107.077297 10.1534/genetics.107.077297 18202390 PMC2206097

[45] Kumar N, Kulwal PL, Gaur A, Tyagi AK, Khurana JP, Khurana P, et al QTL analysis for grain weight in common wheat. Euphytica. 2006;151: 135–144. 10.1007/s10681-006-9133-4

[46] Huang XQ, Kempf H, Ganal MW, Röder MS. Advanced backcross QTL analysis in progenies derived from a cross between a German elite winter wheat variety and a synthetic wheat (Triticum aestivumL.). Theor Appl Genet. 2004;109: 933–943. 10.1007/s00122-004-1708-7 10.1007/s00122-004-1708-7 15243706

[47] Lopes MS, Reynolds MP, McIntyre CL, Mathews KL, Jalal Kamali MR, Mossad M, et al QTL for yield and associated traits in the Seri/Babax population grown across several environments in Mexico, in the West Asia, North Africa, and South Asia regions. Theor Appl Genet. 2013;126: 971–984. 10.1007/s00122-012-2030-4 10.1007/s00122-012-2030-4 23269228

[48] Wang RX, Hai L, Zhang XY, You GX, Yan CS, Xiao SH. QTL mapping for grain filling rate and yield-related traits in RILs of the Chinese winter wheat population Heshangmai x Yu8679. Theor Appl Genet. 2009;118: 313–325. 10.1007/s00122-008-0901-5 10.1007/s00122-008-0901-5 18853131

[49] Gao F, Wen W, Liu J, Rasheed A, Yin G, Xia X, et al Genome-Wide Linkage Mapping of QTL for Yield Components, Plant Height and Yield-Related Physiological Traits in the Chinese Wheat Cross Zhou 8425B/Chinese Spring. Front Plant Sci. 2015;6: 1099 10.3389/fpls.2015.01099 10.3389/fpls.2015.01099 26734019 PMC4683206

[50] Quarrie SA, Pekic Quarrie S, Radosevic R, Rancic D, Kaminska A, Barnes JD, et al Dissecting a wheat QTL for yield present in a range of environments: From the QTL to candidate genes. J Exp Bot. 2006;57: 2627–2637. 10.1093/jxb/erl026 10.1093/jxb/erl026 16831847

[51] Zhang L-Y, Liu D-C, Guo X-L, Yang W-L, Sun J-Z, Wang D-W, et al Genomic Distribution of Quantitative Trait Loci for Yield and Yield-related Traits in Common Wheat. J Integr Plant Biol. Blackwell Publishing Asia; 2010;52: 996–1007. 10.1111/j.1744-7909.2010.00967.x 10.1111/j.1744-7909.2010.00967.x 20977657

[52] Quarrie SA, Steed A, Calestani C, Semikhodskii A, Lebreton C, Chinoy C, et al A high-density genetic map of hexaploid wheat (Triticum aestivum L.) from the cross Chinese Spring x SQ1 and its use to compare QTLs for grain yield across a range of environments. Theor Appl Genet. 2005;110: 865–880. 10.1007/s00122-004-1902-7 10.1007/s00122-004-1902-7 15719212

[53] Zheng J, Liu H, Wang Y, Wang L, Chang X, Jing R, et al TEF-7A, a transcript elongation factor gene, influences yield-related traits in bread wheat (Triticum aestivum L.). J Exp Bot. 2014;65: 5351–5365. Available: 10.1093/jxb/eru306 25056774 PMC4157721

[54] Evans LT, Fischer RA. Yield Potential: Its Definition, Measurement, and Significance. Crop Sci. Madison, WI: Crop Science Society of America; 1999;39: 1544–1551. 10.2135/cropsci1999.3961544x

[55] Charmet G, Robert N, Branlard G, Linossier L, Martre P, Triboï E. Genetic analysis of dry matter and nitrogen accumulation and protein composition in wheat kernels. Theor Appl Genet. 2005;111: 540–550. 10.1007/s00122-005-2045-1 10.1007/s00122-005-2045-1 15951993

[56] Kamaluddin, Singh RM, Abdin MZ, Khan MA, Alam T, Khan S, et al Inheritance of grain filling duration in spring wheat (Triticum aestivum L. em thell). J Plant Biol. 2007;50: 504–507. 10.1007/BF03030690

[57] Yang J, Zhu J, Williams RW. Mapping the genetic architecture of complex traits in experimental populations. Bioinformatics. 2007;23: 1527–1536. Available: 10.1093/bioinformatics/btm143 17459962

[58] Araki E, Miura H, Sawada S. Identification of genetic loci affecting amylose content and agronomic traits on chromosome 4A of wheat. Theor Appl Genet. 1999;98: 977–984. 10.1007/s001220051158

[59] Kato K, Miura H, Sawada S. Mapping QTLs controlling grain yield and its components on chromosome 5A of wheat. Theor Appl Genet. 2000;101: 1114–1121. 10.1007/s001220051587

[60] Börner A, Schumann E, Fürste A, Cöster H, Leithold B, Röder M, et al Mapping of quantitative trait loci determining agronomic important characters in hexaploid wheat (Triticum aestivum L.). Theor Appl Genet. 2002;105: 921–936. 10.1007/s00122-002-0994-1 10.1007/s00122-002-0994-1 12582918

[61] Groos C, Robert N, Bervas E, Charmet G. Genetic analysis of grain protein-content, grain yield and thousand-kernel weight in bread wheat. Theor Appl Genet. 2003;106: 1032–1040. 10.1007/s00122-002-1111-1 10.1007/s00122-002-1111-1 12671751

[62] Heidari B, Sayed-Tabatabaei BE, Saeidi G, Kearsey M, Suenaga K. Mapping QTL for grain yield, yield components, and spike features in a doubled haploid population of bread wheat. Genome. NRC Research Press; 2011;54: 517–527. 10.1139/g11-017 10.1139/g11-017 21635161

[63] Jiang G-L. Molecular Markers and Marker-Assisted Breeding in Plants. In: Andersen SBBT-PB from L to F, editor. Rijeka: InTech; 2013. p. Ch. 03. 10.5772/52583

[64] Kumar N, Kulwal PL, Balyan HS, Gupta PK. QTL mapping for yield and yield contributing traits in two mapping populations of bread wheat. Mol Breed. 2007;19: 163–177. 10.1007/s11032-006-9056-8

[65] Goldringer I, Brabant P, Gallais A. Estimation of additive and epistatic genetic variances for agronomic traits in a population of doubled-haploid lines of wheat. Heredity (Edinb). 1997;79: 60–71. 10.1038/hdy.1997.123

[66] Wu X, Chang X, Jing R. Genetic insight into yield-associated traits of wheat grown in multiple rain-fed environments. PLoS One. 2012;7. 10.1371/journal.pone.0031249 10.1371/journal.pone.0031249 PMC3281929 22363596

