# Supplementary material for: QTL mapping for nine drought-responsive agronomic traits in bread wheat under irrigated and rain-fed environments
Source: PLoS One. 2017 Aug 9;12(8):e0182857. doi: 10.1371/journal.pone.0182857 (PMC5550002; doi:10.1371/journal.pone.0182857)
Supplement: S1 Fig — (A) Kanpur, (B) Karnal, (C) Pune, and (D) Hisar. (PDF) [file pone.0182857.s001.pdf]

(a)

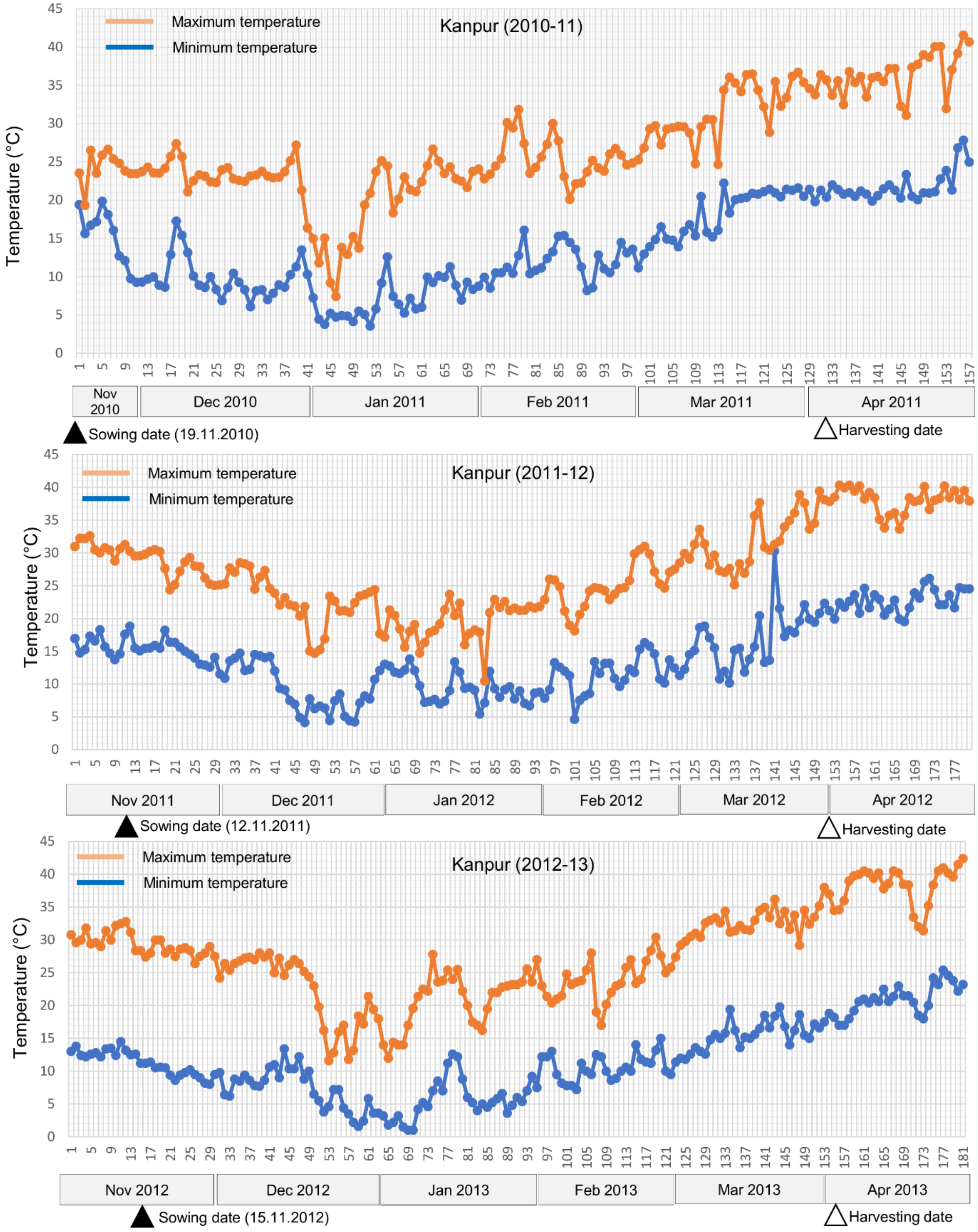
Kanpur (2010-11)
Maximum temperature
Minimum temperature
Temperature (°C)
Nov 2010
Dec 2010
Jan 2011
Feb 2011
Mar 2011
Apr 2011
Sowing date (19.11.2010)
Harvesting date
Kanpur (2011-12)
Maximum temperature
Minimum temperature
Temperature (°C)
Nov 2011
Dec 2011
Jan 2012
Feb 2012
Mar 2012
Apr 2012
Sowing date (12.11.2011)
Harvesting date
Kanpur (2012-13)
Maximum temperature
Minimum temperature
Temperature (°C)
Nov 2012
Dec 2012
Jan 2013
Feb 2013
Mar 2013
Apr 2013
Sowing date (15.11.2012)
Harvesting date

(b)

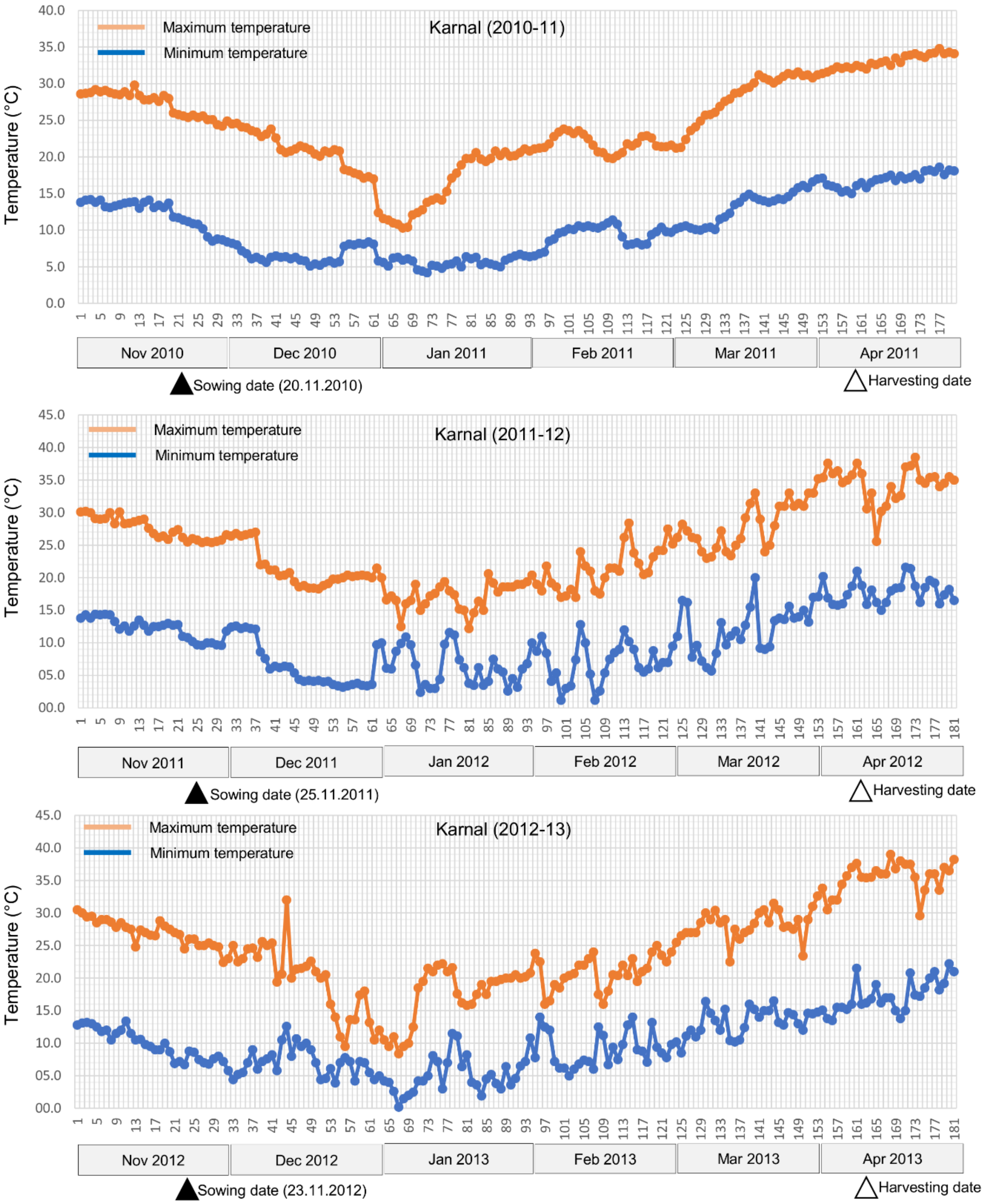
Karnal (2010-11)
Maximum temperature
Minimum temperature
Temperature (°C)
Nov 2010
Dec 2010
Jan 2011
Feb 2011
Mar 2011
Apr 2011
Sowing date (20.11.2010)
Harvesting date
Karnal (2011-12)
Maximum temperature
Minimum temperature
Temperature (°C)
Nov 2011
Dec 2011
Jan 2012
Feb 2012
Mar 2012
Apr 2012
Sowing date (25.11.2011)
Harvesting date
Karnal (2012-13)
Maximum temperature
Minimum temperature
Temperature (°C)
Nov 2012
Dec 2012
Jan 2013
Feb 2013
Mar 2013
Apr 2013
Sowing date (23.11.2012)
Harvesting date

(c)

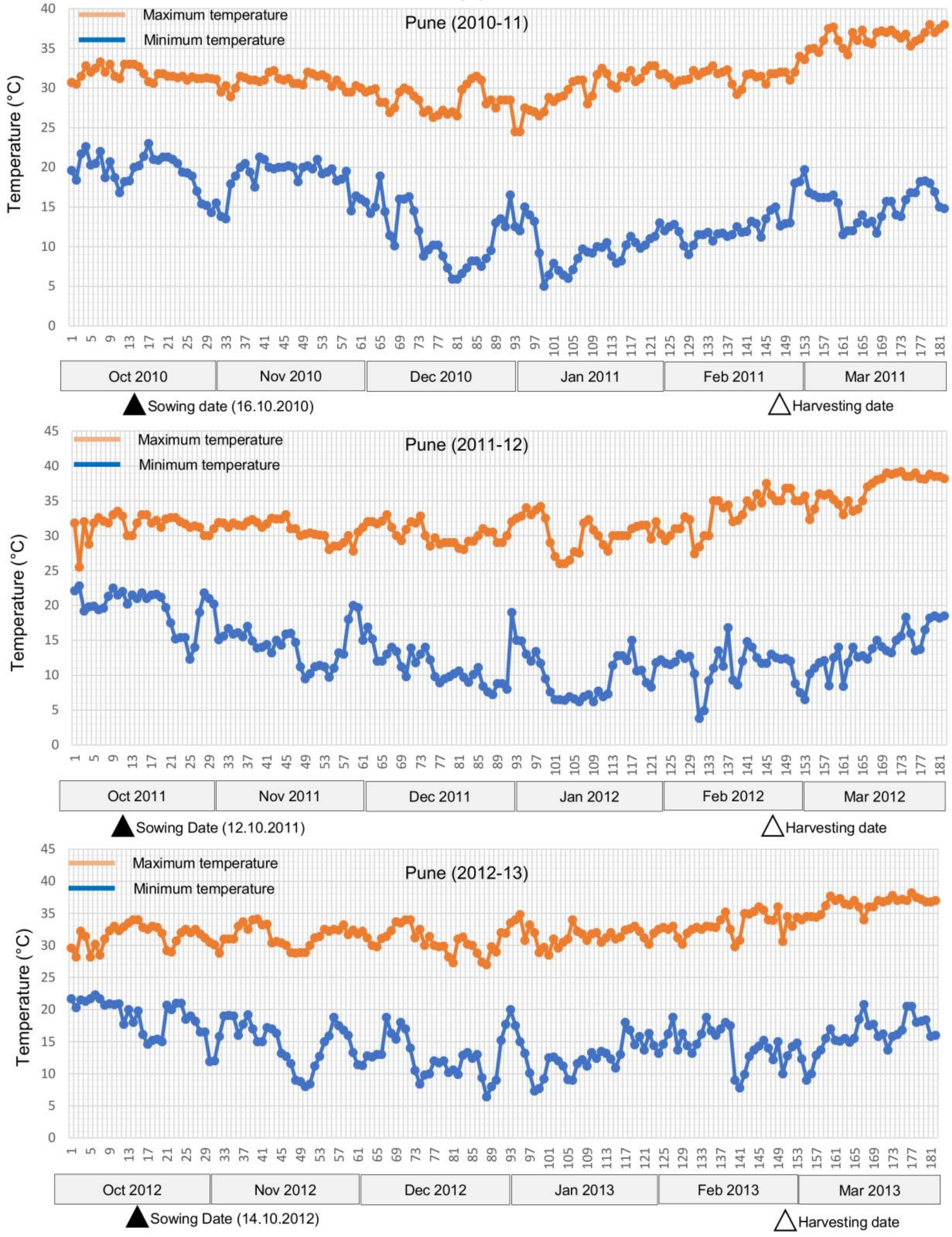

Maximum temperature
Minimum temperature
Pune (2010-11)
Temperature (°C)
Oct 2010
Nov 2010
Dec 2010
Jan 2011
Feb 2011
Mar 2011
Sowing date (16.10.2010)
Harvesting date
Maximum temperature
Minimum temperature
Pune (2011-12)
Temperature (°C)
Oct 2011
Nov 2011
Dec 2011
Jan 2012
Feb 2012
Mar 2012
Sowing Date (12.10.2011)
Harvesting date
Maximum temperature
Minimum temperature
Pune (2012-13)
Temperature (°C)
Oct 2012
Nov 2012
Dec 2012
Jan 2013
Feb 2013
Mar 2013
Sowing Date (14.10.2012)
Harvesting date

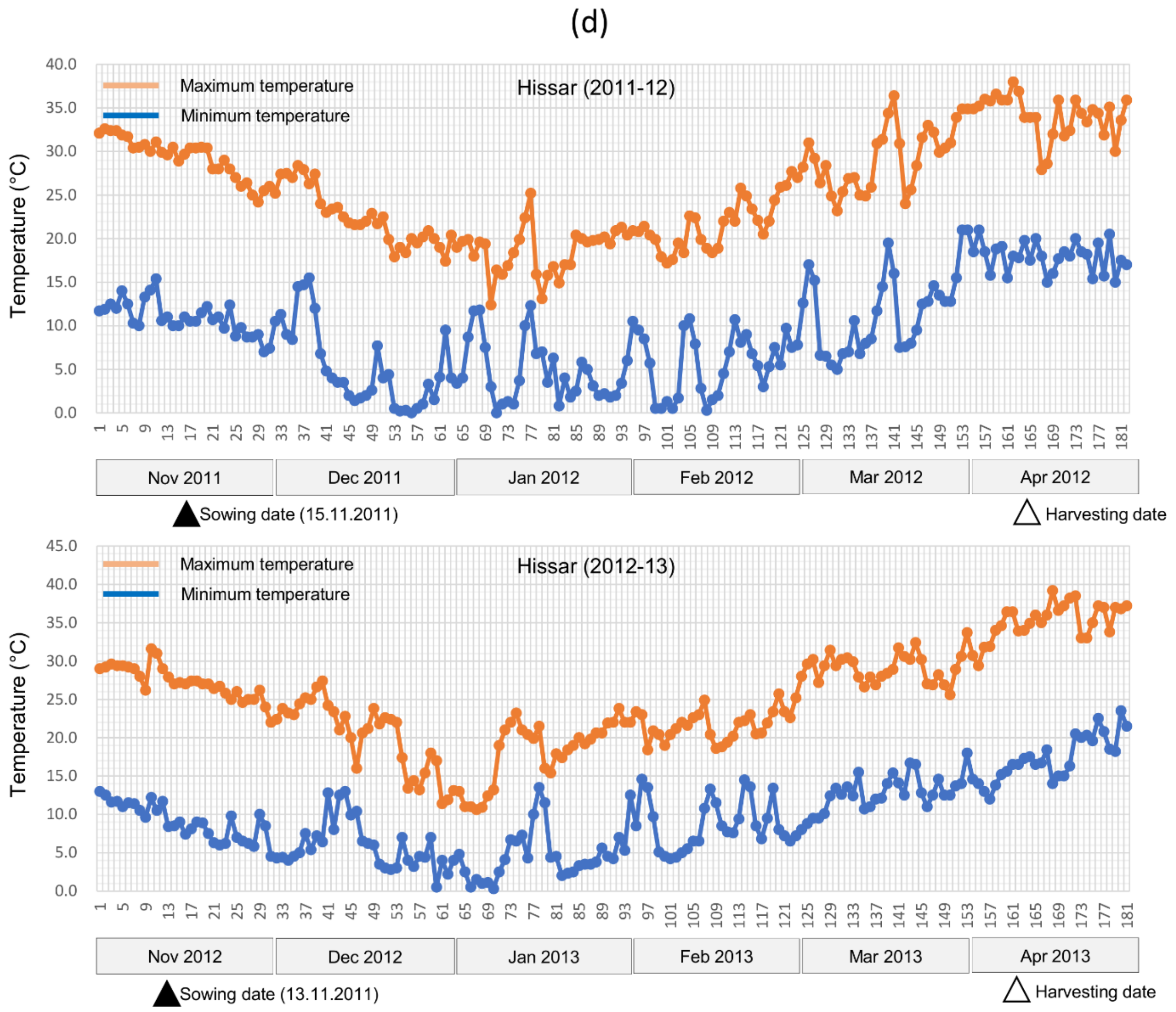


**S1 Fig.** Minimum and maximum temperatures during the crop growth period for three crop seasons (2010-11 to 2012-13) at four locations in India. (a) Kanpur (b) Karnal (c) Pune and (d) Hisar.
