# Supplementary material for: QTL mapping for nine drought-responsive agronomic traits in bread wheat under irrigated and rain-fed environments
Source: PLoS One. 2017 Aug 9;12(8):e0182857. doi: 10.1371/journal.pone.0182857 (PMC5550002; doi:10.1371/journal.pone.0182857)
Supplement: S1 Table — (PDF) [file pone.0182857.s002.pdf]

S1 Table. Total water applied through irrigation to the Kukri/Excalibur DH mapping population during the 2010–2013 crop cycles in different environments.

Locations	Irrigated/rainfed	Crop-season	No. of irrigation	Measure of irrigation (mm)	Total measure of irrigation (mm)
Kanpur	IR	2010-11	4	60	240
Kanpur	RF	2010-11	1	60	60
Kanpur	IR	2011-12	4	60	240
Kanpur	RF	2011-12	1	60	60
Kanpur	IR	2012-13	4	60	240
Kanpur	RF	2012-13	1	60	60
Hissar	IR	2010-11	No phenotypic data		
Hissar	RF	2010-11	No phenotypic data		
Hissar	IR	2011-12	4	60	240
Hissar	RF	2011-12	1	60	60
Hissar	IR	2012-13	4	60	240
Hissar	RF	2012-13	1	60	60
Pune	IR	2010-11	4	60	240
Pune	RF	2010-11	1	60	60
Pune	IR	2011-12	4	60	240
Pune	RF	2011-12	1	60	60
Pune	IR	2012-13	4	60	240
Pune	RF	2012-13	1	60	60
Karnal	IR	2010-11	4	60	240
Karnal	RF	2010-11	1	60	60
Karnal	IR	2011-12	4	60	240
Karnal	RF	2011-12	1	60	60
Karnal	IR	2012-13	4	60	240
Karnal	RF	2012-13	1	60	60

The recommended practice: four irrigations. Ist irrigation at 21 days after sowing, IInd irrigation after 40 days, IIIrd irrigation around after 60 days of sowing and IVth irrigation after 80 days (milking stage), in rainfed trails only Ist Irrigation at 21 days after sowing was given.
