# Supplementary material for: QTL mapping for nine drought-responsive agronomic traits in bread wheat under irrigated and rain-fed environments
Source: PLoS One. 2017 Aug 9;12(8):e0182857. doi: 10.1371/journal.pone.0182857 (PMC5550002; doi:10.1371/journal.pone.0182857)
Supplement: S2 Table — (PDF) [file pone.0182857.s003.pdf]

Phenotypic data on DH line (Excallibur X Kukri)

Traits name (trait abbreviation)

1. Germination percentage (GP)
2. Days to anthesis (DTA)
3. Days to maturity (DTM)
4. Grain filling duration (GFD)
5. Plant height in cm (PH)
6. Grain weight per ear in g (GWPE)
7. Productive tillers per meter (PTPM)
8. Thousand grain weight in g (TGW)
9. Grain yield per plot g (GYPP)

Locations

Kanpur, India: Year 2010-11; 2011-12; 2012-13
Karnal, India: Year 2010-11; 2011-12; 2012-13
Pune, India: Year 2010-11; 2011-12; 2012-13
Hissar, India: Year 2010-11; 2011-12; 2012-13

N/A: data were not aviable
* phenotypic data with decimal places were rounded off.

**Location**	Kanpur									
**Crop-season**	2010-11									
**Condition**	Rainfed									
**S.No.**	**DH-2 Line code**	**GP***	**DTA**	**PH***	**DTM**	**GFD**	**PTPM**	**GWPE**	**TGW**	**GYPP**
CHK1	Chk 1 (WH147)	47	73	81	108	35	170	NA	NA	173
CHK2	Chk 2 (PBW175)	55	72	96	107	35	207	NA	NA	240
CHK3	Chk 3 (NI5439)	56	71	91	106	35	201	NA	NA	220
1	DH-E001	50	75	68	107	32	118	NA	NA	140
2	DH-E003	58	73	68	105	32	185	NA	NA	200
3	DH-E005	51	73	70	108	35	165	NA	NA	165
4	DH-E006	50	72	65	105	33	157	NA	NA	150
5	DH-E007	48	76	71	108	32	161	NA	NA	175
6	DH-E008	48	75	70	106	31	128	NA	NA	135
7	DH-E009	43	74	70	105	31	117	NA	NA	127
8	DH-E010	47	74	72	105	31	97	NA	NA	108
9	DH-E014	47	75	71	110	35	103	NA	NA	118
10	DH-E015	46	72	67	104	32	158	NA	NA	140
11	DH-E018	46	72	69	107	35	168	NA	NA	160
12	DH-E019	48	72	69	106	34	128	NA	NA	137
13	DH-E020	51	71	69	107	36	196	NA	NA	190
14	DH-E021	49	72	58	109	37	192	NA	NA	200
15	DH-E022	65	73	57	109	36	190	NA	NA	200
16	DH-E023	42	73	60	108	35	170	NA	NA	180
CHK1	Chk 1 (WH147)	47	74	96	107	33	207	NA	NA	230
CHK2	Chk 2 (PBW175)	51	72	90	108	36	201	NA	NA	219
CHK3	Chk 3 (NI5439)	42	72	81	107	35	196	NA	NA	207
17	DH-E024	46	73	60	108	35	158	NA	NA	150
18	DH-E025	45	72	61	104	32	159	NA	NA	162
19	DH-E026	46	71	62	105	34	160	NA	NA	178
20	DH-E029	45	72	68	112	40	120	NA	NA	132
21	DH-E032	46	76	69	109	33	103	NA	NA	119
22	DH-E033	47	75	63	107	32	157	NA	NA	160
23	DH-E034	46	70	65	108	38	140	NA	NA	133
24	DH-E036	45	73	70	109	36	205	NA	NA	208
25	DH-E038	45	75	59	107	32	192	NA	NA	182
26	DH-E039	50	81	64	111	30	196	NA	NA	198
27	DH-E040	51	81	62	111	30	198	NA	NA	210
28	DH-E041	50	82	58	112	30	120	NA	NA	140
29	DH-E042	48	76	62	110	34	156	NA	NA	150
30	DH-E043	43	73	41	106	33	158	NA	NA	152
31	DH-E044	45	75	60	107	32	160	NA	NA	153
32	DH-E046	45	78	70	107	29	160	NA	NA	165
CHK1	Chk 1 (WH147)	56	78	81	110	32	214	NA	NA	215
CHK2	Chk 2 (PBW175)	54	77	90	107	30	196	NA	NA	207
CHK3	Chk 3 (NI5439)	53	77	72	108	31	192	NA	NA	198
33	DH-E047	43	76	61	104	28	157	NA	NA	167
34	DH-E051	42	82	61	111	29	121	NA	NA	135
35	DH-E052	48	77	63	107	30	133	NA	NA	130
36	DH-E053	48	78	72	108	30	201	NA	NA	230
37	DH-E054	43	79	71	110	31	159	NA	NA	167

38	DH-E055	47	78	60	109	31	162	NA	NA	160
39	DH-E056	55	77	65	108	31	212	NA	NA	207
40	DH-E058	51	81	64	111	30	193	NA	NA	198
41	DH-E059	47	78	69	105	27	159	NA	NA	164
42	DH-E060	43	73	61	106	33	151	NA	NA	165
43	DH-E061	47	74	63	108	34	154	NA	NA	170
44	DH-E062	47	78	64	109	31	127	NA	NA	138
45	DH-E063	42	78	68	106	28	117	NA	NA	120
46	DH-E065	47	78	65	105	27	198	NA	NA	101
47	DH-E066	47	77	66	112	35	139	NA	NA	142
48	DH-E067	48	77	66	111	34	180	NA	NA	192
CHK1	Chk 1 (WH147)	55	78	67	109	31	220	NA	NA	230
CHK2	Chk 2 (PBW175)	56	82	92	110	28	222	NA	NA	235
CHK3	Chk 3 (NI5439)	52	82	93	111	29	207	NA	NA	210
49	DH-E068	43	83	64	104	21	100	NA	NA	109
50	DH-E069	41	74	60	106	32	156	NA	NA	163
51	DH-E070	40	84	63	107	23	148	NA	NA	149
52	DH-E071	43	78	64	106	28	218	NA	NA	220
53	DH-E072	50	78	64	108	30	162	NA	NA	182
54	DH-E073	47	76	64	108	32	165	NA	NA	169
55	DH-E074	50	78	65	106	28	170	NA	NA	182
56	DH-E075	50	83	77	109	26	196	NA	NA	207
57	DH-E076	49	81	61	114	33	196	NA	NA	207
58	DH-E077	48	76	62	110	34	192	NA	NA	210
59	DH-E078	49	75	61	108	33	160	NA	NA	182
60	DH-E080	49	79	69	110	31	150	NA	NA	172
61	DH-E081	50	75	60	109	34	153	NA	NA	163
62	DH-E082	49	76	62	108	32	154	NA	NA	150
63	DH-E083	47	76	61	109	33	120	NA	NA	148
64	DH-E084	46	77	60	107	30	110	NA	NA	130
CHK1	Chk 1 (WH147)	49	77	78	109	32	166	NA	NA	187
CHK2	Chk 2 (PBW175)	55	77	90	110	33	220	NA	NA	230
CHK3	Chk 3 (NI5439)	52	76	81	112	36	207	NA	NA	220
65	DH-E086	46	75	61	109	34	96	NA	NA	107
66	DH-E087	47	85	58	114	29	142	NA	NA	150
67	DH-E088	49	84	60	113	29	180	NA	NA	178
68	DH-E090	48	79	71	113	34	138	NA	NA	148
69	DH-E091	44	80	67	110	30	196	NA	NA	199
70	DH-E092	47	76	69	111	35	160	NA	NA	167
71	DH-E093	46	78	65	111	33	120	NA	NA	140
72	DH-E094	49	79	66	112	33	196	NA	NA	200
73	DH-E095	47	81	65	110	29	194	NA	NA	198
74	DH-E096	48	78	63	108	30	192	NA	NA	197
75	DH-E097	47	76	62	106	30	168	NA	NA	182
76	DH-E098	40	77	64	107	30	152	NA	NA	160
77	DH-E099	46	76	65	109	33	156	NA	NA	150
78	DH-E100	47	78	64	108	30	160	NA	NA	167
79	DH-E102	43	84	63	115	31	110	NA	NA	120
80	DH-E103	46	83	72	114	31	140	NA	NA	138
CHK1	Chk 1 (WH147)	53	80	78	110	30	207	NA	NA	210

CHK2	Chk 2 (PBW175)	54	81	91	111	30	220	NA	NA	230
CHK3	Chk 3 (NI5439)	51	81	79	111	30	196	NA	NA	200
81	DH-E104	47	74	73	108	34	180	NA	NA	189
82	DH-E105	49	78	60	109	31	92	NA	NA	103
83	DH-E108	49	79	62	110	31	201	NA	NA	208
84	DH-E109	50	78	57	108	30	158	NA	NA	163
85	DH-E110	49	77	61	108	31	162	NA	NA	170
86	DH-E111	56	81	62	109	28	172	NA	NA	180
87	DH-E113	47	81	61	111	30	120	NA	NA	132
88	DH-E114	49	77	57	109	32	190	NA	NA	198
89	DH-E115	50	75	60	106	31	192	NA	NA	260
90	DH-E117	48	77	68	108	31	168	NA	NA	172
91	DH-E118	49	79	70	109	30	158	NA	NA	162
92	DH-E119	48	76	71	110	34	161	NA	NA	158
93	DH-E120	47	77	69	109	32	157	NA	NA	164
94	DH-E121	43	76	68	110	34	110	NA	NA	124
95	DH-E122	42	75	61	108	33	142	NA	NA	152
96	DH-E123	49	76	62	106	30	173	NA	NA	180
CHK1	Chk 1 (WH147)	56	77	90	109	32	165	NA	NA	230
CHK2	Chk 2 (PBW175)	55	78	90	108	30	212	NA	NA	225
CHK3	Chk 3 (NI5439)	52	79	80	110	31	218	NA	NA	195
97	DH-E124	47	85	65	116	31	98	NA	NA	107
98	DH-E125	43	84	66	114	30	148	NA	NA	154
99	DH-E126	45	74	66	108	34	138	NA	NA	135
100	DH-E127	59	77	67	106	29	193	NA	NA	201
101	DH-E129	43	78	64	109	31	163	NA	NA	186
102	DH-E130	47	79	65	110	31	165	NA	NA	190
103	DH-E131	48	76	63	105	29	163	NA	NA	180
104	DH-E132	53	81	70	110	29	180	NA	NA	207
105	DH-E133	45	78	64	109	31	192	NA	NA	138
106	DH-E134	59	76	68	106	30	121	NA	NA	201
107	DH-E135	50	77	78	108	31	196	NA	NA	196
108	DH-E137	50	78	70	109	31	192	NA	NA	196
109	DH-E138	51	77	72	104	27	190	NA	NA	180
110	DH-E141	52	78	68	110	32	162	NA	NA	162
111	DH-E144	50	85	67	115	30	153	NA	NA	154
112	DH-E145	48	84	70	114	30	152	NA	NA	163
CHK1	Chk 1 (WH147)	57	81	90	111	30	220	NA	NA	235
CHK2	Chk 2 (PBW175)	56	82	81	111	29	215	NA	NA	230
CHK3	Chk 3 (NI5439)	50	77	58	109	32	169	NA	NA	186
113	DH-E146	47	74	70	107	33	157	NA	NA	168
114	DH-E147	53	78	68	108	30	153	NA	NA	120
115	DH-E148	45	76	69	108	32	110	NA	NA	152
116	DH-E149	48	77	68	109	32	140	NA	NA	180
117	DH-E150	45	79	59	110	31	173	NA	NA	196
118	DH-E152	47	68	63	108	40	96	NA	NA	101
119	DH-E153	43	77	66	109	32	148	NA	NA	152
120	DH-E154	41	79	62	111	32	138	NA	NA	142
121	DH-E155	51	75	61	106	31	196	NA	NA	201
122	DH-E156	50	77	60	110	33	190	NA	NA	138

123	DH-E158	48	79	57	109	30	154	NA	NA	168
124	DH-E159	47	76	59	108	32	156	NA	NA	163
125	DH-E161	49	76	58	107	31	158	NA	NA	168
126	DH-E162	43	77	59	108	31	159	NA	NA	170
127	DH-E164	49	76	58	109	33	154	NA	NA	160
128	DH-E165	43	76	60	108	32	121	NA	NA	138
CHK1	Chk 1 (WH147)	54	81	90	110	29	210	NA	NA	225
CHK2	Chk 2 (PBW175)	53	80	79	110	30	196	NA	NA	207
CHK3	Chk 3 (NI5439)	52	80	58	109	29	192	NA	NA	208
129	DH-E166	46	77	62	109	32	142	NA	NA	153
130	DH-E167	45	85	62	112	27	180	NA	NA	192
131	DH-E168	47	84	63	111	27	96	NA	NA	101
132	DH-E169	45	74	67	106	32	150	NA	NA	162
133	DH-E170	46	77	66	108	31	138	NA	NA	130
134	DH-E171	47	78	68	106	28	201	NA	NA	218
135	DH-E172	45	79	63	109	30	160	NA	NA	168
136	DH-E175	47	77	61	109	32	159	NA	NA	164
137	DH-E176	55	79	91	108	29	220	NA	NA	230
138	DH-E177	53	76	59	106	30	190	NA	NA	200
139	DH-E178	51	77	59	108	31	107	NA	NA	196
140	DH-E179	45	78	56	109	31	160	NA	NA	121
141	DH-E180	47	77	57	104	27	158	NA	NA	172
142	DH-E181	48	78	60	110	32	157	NA	NA	162
143	DH-E182	47	78	59	109	31	158	NA	NA	150
144	DH-E183	47	85	59	115	30	159	NA	NA	155
CHK1	Chk 1 (WH147)	51	77	63	107	30	148	NA	NA	150
CHK2	Chk 2 (PBW175)	47	79	62	106	27	152	NA	NA	156
CHK3	Chk 3 (NI5439)	46	78	63	107	29	155	NA	NA	163
145	DH-E184	48	84	58	114	30	140	NA	NA	162
146	DH-E185	47	75	63	107	32	179	NA	NA	148
147	DH-E186	49	76	64	108	32	170	NA	NA	183
148	DH-E187	45	79	66	110	31	138	NA	NA	141
149	DH-E188	50	78	58	108	30	201	NA	NA	210
150	DH-E189	49	77	63	109	32	160	NA	NA	180
151	DH-E190	48	81	66	111	30	158	NA	NA	162
152	DH-E191	47	82	62	110	28	163	NA	NA	168
153	DH-E192	49	83	60	112	29	190	NA	NA	196
154	DH-E193	55	83	90	111	28	207	NA	NA	215
155	DH-E194	54	83	91	111	28	210	NA	NA	230
156	DH-E195	50	77	80	108	31	158	NA	NA	162
157	DH-E196	51	75	62	106	31	150	NA	NA	160
158	DH-E197	46	71	58	103	32	163	NA	NA	172
159	DH-E198	47	72	58	105	33	148	NA	NA	152
160	DH-E199	48	71	57	105	34	150	NA	NA	162
CHK1	Chk 1 (WH147)	56	71	90	108	37	207	NA	NA	220
CHK2	Chk 2 (PBW175)	53	72	63	106	34	180	NA	NA	192
CHK3	Chk 3 (NI5439)	55	73	62	109	36	162	NA	NA	180
161	DH-E200	49	71	58	106	35	153	NA	NA	158
162	DH-E202	47	72	59	105	33	150	NA	NA	163
163	DH-E203	48	78	63	113	35	152	NA	NA	163

164	DH-E204	48	77	65	111	34	110	NA	NA	130
165	DH-E205	45	78	65	105	27	138	NA	NA	142
166	DH-E206	46	72	66	109	37	178	NA	NA	182
167	DH-E207	47	73	65	108	35	92	NA	NA	101
168	DH-E208	46	74	58	107	33	148	NA	NA	153
169	DH-E209	48	72	63	108	36	137	NA	NA	148
170	DH-E210	45	73	66	109	36	196	NA	NA	201
171	DH-E211	49	73	62	108	35	160	NA	NA	181
172	DH-E213	48	75	62	109	34	162	NA	NA	183
173	DH-E214	43	75	60	112	37	196	NA	NA	207
174	DH-E215	48	74	61	111	37	198	NA	NA	210
175	DH-E216	55	73	90	106	33	210	NA	NA	225
176	DH-E217	48	73	63	110	37	147	NA	NA	152
CHK1	Chk 1 (WH147)	55	74	91	108	34	210	NA	NA	230
CHK2	Chk 2 (PBW175)	54	75	90	109	34	215	NA	NA	225
CHK3	Chk 3 (NI5439)	53	73	58	109	36	203	NA	NA	207
177	DH-E218	47	72	63	105	33	150	NA	NA	162
178	DH-E219	43	78	60	110	32	154	NA	NA	150
179	DH-E220	45	77	59	110	33	110	NA	NA	107
180	DH-E221	46	70	59	109	39	148	NA	NA	152
181	DH-E222	45	70	63	106	36	180	NA	NA	183
182	DH-E223	45	74	63	109	35	146	NA	NA	153
183	DH-E224	46	73	67	104	31	130	NA	NA	143
184	DH-E226	43	72	66	110	38	160	NA	NA	180
185	DH-E227	43	75	58	109	34	162	NA	NA	173
186	DH-E228	46	74	62	115	41	201	NA	NA	210
187	DH-E229	47	75	66	114	39	196	NA	NA	198
188	DH-E230	49	75	63	107	32	192	NA	NA	201
189	DH-E231	48	75	61	108	33	190	NA	NA	198
190	DH-E232	47	72	60	108	36	180	NA	NA	190
191	DH-E233	48	70	61	109	39	150	NA	NA	162
192	DH-E233	49	72	60	110	38	152	NA	NA	164
**Location**	Kanpur									
**Crop-season**	2010-11									
**Condition**	Irrigated									
S.No.	**DH-2 Line code**	**GP***	**DTA**	**PH***	**DTM**	**GFD**	**PTPM**	**GWPE**	**TGW**	**GYPP**
CHK1	Chk 1 (WH147)	52	82	88	120	38	183	NA	NA	260
CHK2	Chk 2 (PBW175)	66	81	105	117	36	231	NA	NA	344
CHK3	Chk 3 (NI5439)	67	80	97	118	38	230	NA	NA	340
1	DH-E001	55	83	76	115	32	130	NA	NA	184
2	DH-E003	63	81	71	113	32	214	NA	NA	319
3	DH-E005	56	83	73	115	32	170	NA	NA	252
4	DH-E006	64	78	71	113	35	216	NA	NA	320
5	DH-E007	51	82	75	117	35	164	NA	NA	145
6	DH-E008	50	80	73	115	35	131	NA	NA	196
7	DH-E009	49	79	76	114	35	120	NA	NA	180
8	DH-E010	48	79	77	114	35	120	NA	NA	148
9	DH-E014	52	83	75	118	35	186	NA	NA	286
10	DH-E015	51	78	71	117	39	182	NA	NA	240
11	DH-E018	51	81	75	119	38	171	NA	NA	250

12	DH-E019	56	81	73	115	34	130	NA	NA	200
13	DH-E020	66	80	73	115	35	185	NA	NA	298
14	DH-E021	64	83	62	118	35	191	NA	NA	310
15	DH-E022	60	83	60	119	36	207	NA	NA	305
16	DH-E023	57	83	65	118	35	175	NA	NA	250
CHK1	Chk 1 (WH147)	52	80	105	115	35	226	NA	NA	261
CHK2	Chk 2 (PBW175)	67	80	103	116	36	214	NA	NA	345
CHK3	Chk 3 (NI5439)	57	83	88	115	32	208	NA	NA	340
17	DH-E024	51	80	65	116	36	161	NA	NA	241
18	DH-E025	50	78	65	114	36	165	NA	NA	238
19	DH-E026	51	79	66	114	35	166	NA	NA	247
20	DH-E029	50	79	71	115	36	126	NA	NA	185
21	DH-E032	51	83	74	119	36	109	NA	NA	290
22	DH-E033	52	78	66	118	40	161	NA	NA	286
23	DH-E034	50	81	70	117	36	145	NA	NA	242
24	DH-E036	60	80	74	115	35	226	NA	NA	330
25	DH-E038	50	83	64	115	32	140	NA	NA	205
26	DH-E039	63	83	66	117	34	216	NA	NA	310
27	DH-E040	61	83	67	117	34	212	NA	NA	303
28	DH-E041	56	83	65	118	35	125	NA	NA	195
29	DH-E042	50	78	66	116	38	170	NA	NA	220
30	DH-E043	50	78	67	112	34	163	NA	NA	240
31	DH-E044	50	80	66	113	33	167	NA	NA	242
32	DH-E046	50	79	80	113	34	168	NA	NA	240
CHK1	Chk 1 (WH147)	67	81	88	116	35	214	NA	NA	307
CHK2	Chk 2 (PBW175)	64	80	105	113	33	208	NA	NA	310
CHK3	Chk 3 (NI5439)	65	80	86	114	34	209	NA	NA	303
33	DH-E047	49	80	69	112	32	163	NA	NA	248
34	DH-E051	50	84	68	117	33	130	NA	NA	190
35	DH-E052	50	80	68	113	33	146	NA	NA	230
36	DH-E053	64	81	78	114	33	230	NA	NA	320
37	DH-E054	51	82	79	116	34	170	NA	NA	250
38	DH-E055	52	81	65	115	34	174	NA	NA	250
39	DH-E056	66	80	71	114	34	230	NA	NA	328
40	DH-E058	61	83	70	117	34	207	NA	NA	287
41	DH-E059	50	78	79	111	33	161	NA	NA	218
42	DH-E060	50	78	69	112	34	165	NA	NA	220
43	DH-E061	51	81	70	114	33	164	NA	NA	220
44	DH-E062	50	82	71	116	34	132	NA	NA	190
45	DH-E063	49	80	72	114	34	121	NA	NA	178
46	DH-E065	48	80	73	113	33	105	NA	NA	148
47	DH-E066	52	84	75	118	34	151	NA	NA	230
48	DH-E067	50	83	74	117	34	195	NA	NA	270
CHK1	Chk 1 (WH147)	66	83	105	117	34	230	NA	NA	342
CHK2	Chk 2 (PBW175)	67	83	103	117	34	235	NA	NA	350
CHK3	Chk 3 (NI5439)	56	83	86	116	33	214	NA	NA	310
49	DH-E068	52	78	72	113	35	107	NA	NA	150
50	DH-E069	51	81	68	116	35	163	NA	NA	240
51	DH-E070	50	80	70	115	35	153	NA	NA	230
52	DH-E071	52	81	71	116	35	228	NA	NA	320

53	DH-E072	53	82	72	116	34	171	NA	NA	260
54	DH-E073	54	80	74	113	33	175	NA	NA	270
55	DH-E074	55	80	75	113	33	180	NA	NA	230
56	DH-E075	60	83	75	117	34	211	NA	NA	300
57	DH-E076	55	83	70	119	36	215	NA	NA	315
58	DH-E077	56	80	71	116	36	210	NA	NA	310
59	DH-E078	55	78	69	114	36	170	NA	NA	250
60	DH-E080	54	82	74	117	35	161	NA	NA	242
61	DH-E081	55	80	67	115	35	165	NA	NA	247
62	DH-E082	54	79	68	114	35	164	NA	NA	244
63	DH-E083	52	78	67	114	36	132	NA	NA	198
64	DH-E084	51	80	66	114	34	120	NA	NA	180
CHK1	Chk 1 (WH147)	53	81	85	115	34	174	NA	NA	268
CHK2	Chk 2 (PBW175)	66	80	102	116	36	234	NA	NA	352
CHK3	Chk 3 (NI5439)	60	82	91	118	36	211	NA	NA	320
65	DH-E086	50	80	68	113	33	101	NA	NA	132
66	DH-E087	54	85	65	120	35	151	NA	NA	226
67	DH-E088	58	84	66	119	35	190	NA	NA	280
68	DH-E090	58	81	70	117	36	151	NA	NA	230
69	DH-E091	54	82	73	116	34	228	NA	NA	335
70	DH-E092	53	80	75	117	37	171	NA	NA	260
71	DH-E093	50	82	71	118	36	130	NA	NA	199
72	DH-E094	60	83	72	119	36	215	NA	NA	327
73	DH-E095	58	82	70	118	36	211	NA	NA	312
74	DH-E096	57	82	71	114	32	213	NA	NA	318
75	DH-E097	54	80	70	112	32	175	NA	NA	262
76	DH-E098	56	80	72	113	33	162	NA	NA	250
77	DH-E099	57	81	73	115	34	166	NA	NA	243
78	DH-E100	57	80	72	114	34	167	NA	NA	230
79	DH-E102	56	85	70	120	35	125	NA	NA	190
80	DH-E103	58	84	78	119	35	150	NA	NA	201
CHK1	Chk 1 (WH147)	61	82	86	116	34	216	NA	NA	328
CHK2	Chk 2 (PBW175)	60	83	101	117	34	210	NA	NA	317
CHK3	Chk 3 (NI5439)	58	83	83	117	34	207	NA	NA	307
81	DH-E104	51	76	80	113	37	195	NA	NA	289
82	DH-E105	53	81	66	114	33	107	NA	NA	138
83	DH-E108	60	82	70	116	34	228	NA	NA	307
84	DH-E109	58	81	67	114	33	171	NA	NA	250
85	DH-E110	60	80	69	114	34	174	NA	NA	260
86	DH-E111	66	83	70	114	31	231	NA	NA	330
87	DH-E113	53	83	67	117	34	130	NA	NA	149
88	DH-E114	60	80	65	116	36	210	NA	NA	189
89	DH-E115	62	76	67	112	36	211	NA	NA	320
90	DH-E117	55	80	75	114	34	176	NA	NA	260
91	DH-E118	65	82	77	117	35	160	NA	NA	230
92	DH-E119	57	78	78	116	38	167	NA	NA	250
93	DH-E120	58	80	76	115	35	164	NA	NA	240
94	DH-E121	50	79	76	116	37	123	NA	NA	175
95	DH-E122	57	79	69	114	35	150	NA	NA	240
96	DH-E123	59	80	70	112	32	190	NA	NA	276

CHK1	Chk 1 (WH147)	66	81	101	115	34	228	NA	NA	338
CHK2	Chk 2 (PBW175)	65	82	104	114	32	210	NA	NA	330
CHK3	Chk 3 (NI5439)	57	82	87	117	35	190	NA	NA	280
97	DH-E124	52	85	71	120	35	107	NA	NA	163
98	DH-E125	53	84	72	119	35	161	NA	NA	250
99	DH-E126	54	78	71	112	34	146	NA	NA	230
100	DH-E127	60	80	73	113	33	203	NA	NA	310
101	DH-E129	55	82	72	115	33	171	NA	NA	260
102	DH-E130	56	82	73	116	34	170	NA	NA	262
103	DH-E131	55	80	71	113	33	176	NA	NA	263
104	DH-E132	59	83	77	118	35	207	NA	NA	290
105	DH-E133	52	82	74	115	33	130	NA	NA	198
106	DH-E134	62	79	73	112	33	217	NA	NA	307
107	DH-E135	63	80	75	114	34	215	NA	NA	309
108	DH-E137	61	81	76	115	34	210	NA	NA	300
109	DH-E138	60	80	80	111	31	175	NA	NA	260
110	DH-E141	58	81	76	116	35	160	NA	NA	251
111	DH-E144	58	86	75	120	34	167	NA	NA	265
112	DH-E145	57	85	77	119	34	166	NA	NA	260
CHK1	Chk 1 (WH147)	66	83	106	117	34	212	NA	NA	328
CHK2	Chk 2 (PBW175)	55	83	87	117	34	180	NA	NA	268
CHK3	Chk 3 (NI5439)	56	80	63	115	35	207	NA	NA	289
113	DH-E146	55	78	77	113	35	163	NA	NA	275
114	DH-E147	50	82	76	114	32	125	NA	NA	192
115	DH-E148	51	80	77	114	34	150	NA	NA	240
116	DH-E149	52	82	76	117	35	190	NA	NA	290
117	DH-E150	50	82	65	116	34	105	NA	NA	170
118	DH-E152	56	81	71	114	33	161	NA	NA	260
119	DH-E153	52	80	74	114	34	145	NA	NA	210
120	DH-E154	53	83	70	117	34	170	NA	NA	240
121	DH-E155	51	78	67	112	34	135	NA	NA	210
122	DH-E156	58	80	66	116	36	215	NA	NA	280
123	DH-E158	55	82	61	115	33	161	NA	NA	243
124	DH-E159	56	81	66	116	35	165	NA	NA	250
125	DH-E161	57	78	65	113	35	166	NA	NA	270
126	DH-E162	57	80	66	114	34	167	NA	NA	272
127	DH-E164	56	78	65	115	37	164	NA	NA	250
128	DH-E165	50	78	67	114	36	130	NA	NA	198
CHK1	Chk 1 (WH147)	65	83	105	117	34	215	NA	NA	340
CHK2	Chk 2 (PBW175)	64	82	86	115	33	208	NA	NA	310
CHK3	Chk 3 (NI5439)	64	81	63	115	34	207	NA	NA	315
129	DH-E166	56	80	71	115	35	150	NA	NA	230
130	DH-E167	50	84	70	120	36	190	NA	NA	280
131	DH-E168	51	84	71	119	35	107	NA	NA	148
132	DH-E169	52	76	75	112	36	165	NA	NA	180
133	DH-E170	51	81	74	114	33	151	NA	NA	190
134	DH-E171	65	81	66	116	35	228	NA	NA	340
135	DH-E172	56	82	70	117	35	173	NA	NA	270
136	DH-E175	59	81	69	117	36	175	NA	NA	270
137	DH-E176	66	82	103	117	35	230	NA	NA	345

138	DH-E177	63	78	67	114	36	209	NA	NA	307
139	DH-E178	63	80	63	113	33	210	NA	NA	309
140	DH-E179	50	81	61	114	33	113	NA	NA	182
141	DH-E180	58	80	64	115	35	175	NA	NA	260
142	DH-E181	58	81	66	116	35	163	NA	NA	260
143	DH-E182	57	81	65	114	33	167	NA	NA	250
144	DH-E183	57	85	66	120	35	168	NA	NA	260
CHK1	Chk 1 (WH147)	56	80	71	113	33	165	NA	NA	260
CHK2	Chk 2 (PBW175)	57	82	70	115	33	162	NA	NA	267
CHK3	Chk 3 (NI5439)	56	81	71	114	33	164	NA	NA	264
145	DH-E184	58	84	67	119	35	165	NA	NA	260
146	DH-E185	56	78	71	112	34	150	NA	NA	230
147	DH-E186	59	81	72	114	33	192	NA	NA	290
148	DH-E187	52	82	74	115	33	148	NA	NA	210
149	DH-E188	63	81	66	117	36	230	NA	NA	310
150	DH-E189	56	80	71	112	32	171	NA	NA	260
151	DH-E190	55	83	74	117	34	165	NA	NA	255
152	DH-E191	57	82	70	116	34	172	NA	NA	270
153	DH-E192	63	83	68	118	35	208	NA	NA	309
154	DH-E193	66	83	103	117	34	215	NA	NA	330
155	DH-E194	65	83	107	117	34	214	NA	NA	340
156	DH-E195	56	80	86	114	34	170	NA	NA	260
157	DH-E196	56	78	70	112	34	160	NA	NA	250
158	DH-E197	50	79	64	111	32	175	NA	NA	240
159	DH-E198	51	80	66	113	33	160	NA	NA	230
160	DH-E199	52	79	65	112	33	165	NA	NA	232
CHK1	Chk 1 (WH147)	66	79	105	112	33	220	NA	NA	338
CHK2	Chk 2 (PBW175)	65	80	71	113	33	196	NA	NA	280
CHK3	Chk 3 (NI5439)	50	81	70	114	33	175	NA	NA	248
161	DH-E200	53	79	66	112	33	167	NA	NA	235
162	DH-E202	54	80	67	114	34	166	NA	NA	230
163	DH-E203	54	86	71	120	34	163	NA	NA	238
164	DH-E204	50	85	72	119	34	120	NA	NA	182
165	DH-E205	50	76	73	112	36	150	NA	NA	220
166	DH-E206	52	80	74	113	33	192	NA	NA	270
167	DH-E207	51	81	76	114	33	107	NA	NA	148
168	DH-E208	53	82	66	115	33	160	NA	NA	232
169	DH-E209	50	80	71	113	33	145	NA	NA	210
170	DH-E210	58	81	74	115	34	229	NA	NA	309
171	DH-E211	57	81	70	115	34	171	NA	NA	250
172	DH-E213	53	82	70	117	35	175	NA	NA	242
173	DH-E214	62	83	68	118	35	207	NA	NA	309
174	DH-E215	63	82	67	117	35	210	NA	NA	308
175	DH-E216	63	81	103	114	33	228	NA	NA	340
176	DH-E217	53	80	71	115	35	160	NA	NA	238
CHK1	Chk 1 (WH147)	66	82	105	117	35	225	NA	NA	343
CHK2	Chk 2 (PBW175)	65	83	104	117	34	220	NA	NA	330
CHK3	Chk 3 (NI5439)	64	81	63	116	35	210	NA	NA	310
177	DH-E218	52	81	66	115	34	166	NA	NA	239
178	DH-E219	64	85	66	119	34	165	NA	NA	235

179	DH-E220	50	78	65	112	34	128	NA	NA	182
180	DH-E221	51	80	67	114	34	151	NA	NA	220
181	DH-E222	54	80	71	114	34	196	NA	NA	270
182	DH-E223	52	82	71	115	33	161	NA	NA	238
183	DH-E224	50	81	75	114	33	147	NA	NA	215
184	DH-E226	53	80	74	114	34	172	NA	NA	250
185	DH-E227	54	83	66	117	34	175	NA	NA	262
186	DH-E228	58	82	70	116	34	228	NA	NA	228
187	DH-E229	62	83	71	115	32	215	NA	NA	309
188	DH-E230	63	83	71	119	36	210	NA	NA	310
189	DH-E231	58	83	69	118	35	207	NA	NA	307
190	DH-E232	57	80	68	116	36	196	NA	NA	270
191	DH-E233	56	78	69	113	35	160	NA	NA	240
192	DH-E233	57	80	68	115	35	166	NA	NA	252
**Location**	Karnal									
**Crop-season**	2010-11									
**Condition**	Rainfed									
S.No.	**DH-2 Line code**	**GP***	**DTA**	**PH***	**DTM**	**GFD**	**PTPM**	**GWPE**	**TGW***	**GYPP**
CHK1	Chk 1 (WH147)	95	94	113	123	29	133	1.2	24	131
CHK2	Chk 2 (PBW175)	95	95	109	124	29	132	2.6	47	242
CHK3	Chk 3 (NI5439)	95	96	108	124	28	138	2.0	45	174
1	DH-E001	90	92	84	122	30	205	1.2	26	271
2	DH-E003	95	102	99	129	27	163	1.9	35	120
3	DH-E005	95	92	75	122	30	144	0.9	19	105
4	DH-E006	95	94	83	121	27	102	1.3	24	164
5	DH-E007	95	102	87	127	25	142	1.6	28	147
6	DH-E008	95	83	84	121	38	152	1.8	40	134
7	DH-E009	90	99	92	126	27	163	1.9	39	142
8	DH-E010	95	99	97	126	27	166	1.0	26	130
9	DH-E014	85	92	92	121	29	126	1.4	31	174
10	DH-E015	90	90	88	123	33	142	1.6	32	297
11	DH-E018	95	85	88	122	37	91	1.7	34	359
12	DH-E019	90	99	87	127	28	147	0.9	23	206
13	DH-E020	95	90	92	123	33	146	1.8	29	240
14	DH-E021	95	89	90	124	35	141	2.1	33	137
15	DH-E022	90	95	75	124	29	158	1.4	25	263
16	DH-E023	95	102	86	130	28	171	1.5	26	261
CHK1	Chk 1 (WH147)	90	95	120	124	29	178	1.9	36	167
CHK2	Chk 2 (PBW175)	95	95	112	124	29	140	2.2	44	230
CHK3	Chk 3 (NI5439)	80	98	111	127	29	99	1.9	51	225
17	DH-E024	95	86	75	122	36	186	1.5	32	315
18	DH-E025	95	86	80	123	37	164	1.6	33	196
19	DH-E026	95	104	90	131	27	194	1.6	30	219
20	DH-E029	95	102	85	129	27	196	1.6	29	168
21	DH-E032	90	96	82	125	29	210	1.5	32	210
22	DH-E033	95	88	89	122	34	178	1.8	36	169
23	DH-E034	95	88	84	122	34	195	0.8	16	201
24	DH-E036	95	89	85	123	34	128	1.3	34	236
25	DH-E038	95	104	88	130	26	166	1.5	34	253
26	DH-E039	95	95	92	122	27	178	1.5	34	152

27	DH-E040	95	102	90	128	26	162	1.3	22	220
28	DH-E041	90	96	85	125	29	184	2.1	44	191
29	DH-E042	95	103	92	127	24	122	1.4	28	140
30	DH-E043	95	94	87	123	29	176	1.8	32	102
31	DH-E044	95	102	85	126	24	189	1.7	29	205
32	DH-E046	85	102	95	126	24	159	1.4	31	184
CHK1	Chk 1 (WH147)	95	103	88	129	26	102	0.9	26	93
CHK2	Chk 2 (PBW175)	95	96	97	125	29	98	1.4	31	186
CHK3	Chk 3 (NI5439)	95	91	87	122	31	100	1.6	37	180
33	DH-E047	85	98	95	124	26	148	1.0	25	121
34	DH-E051	90	92	80	121	29	98	1.2	26	132
35	DH-E052	95	89	87	121	32	114	1.5	31	123
36	DH-E053	85	102	90	126	24	126	1.6	32	155
37	DH-E054	85	102	85	126	24	121	1.4	30	206
38	DH-E055	95	98	82	126	28	125	1.5	33	120
39	DH-E056	95	102	93	129	27	105	1.2	29	263
40	DH-E058	95	104	89	130	26	109	1.6	35	222
41	DH-E059	95	88	93	122	34	98	1.6	35	273
42	DH-E060	95	91	90	123	32	115	1.5	31	219
43	DH-E061	95	95	108	124	29	121	1.4	34	192
44	DH-E062	95	95	128	124	29	136	2.6	43	217
45	DH-E063	95	95	110	124	29	113	2.0	50	237
46	DH-E065	95	88	72	122	34	90	2.5	50	215
47	DH-E066	95	102	76	126	24	129	2.1	39	198
48	DH-E067	95	99	87	125	26	98	2.2	41	190
CHK1	Chk 1 (WH147)	95	96	108	122	26	153	1.5	33	253
CHK2	Chk 2 (PBW175)	95	96	120	122	26	136	2.2	42	174
CHK3	Chk 3 (NI5439)	95	96	100	122	26	198	1.5	45	133
49	DH-E068	95	88	90	122	34	95	1.6	32	182
50	DH-E069	95	91	94	122	31	115	1.5	31	220
51	DH-E070	95	100	78	127	27	118	0.9	23	267
52	DH-E071	95	89	80	123	34	141	1.3	31	159
53	DH-E072	95	89	90	122	33	92	1.4	26	213
54	DH-E073	95	98	87	125	27	88	1.5	31	167
55	DH-E074	95	102	93	126	24	95	1.5	29	226
56	DH-E075	95	94	89	121	27	155	1.8	30	216
57	DH-E076	90	87	77	123	36	142	1.4	29	99
58	DH-E077	95	86	82	121	35	124	1.8	38	293
59	DH-E078	95	92	83	121	29	198	1.7	28	200
60	DH-E080	95	94	89	122	28	181	1.4	28	165
61	DH-E081	95	102	93	127	25	175	1.3	24	210
62	DH-E082	95	95	95	122	27	145	1.3	30	222
63	DH-E083	95	100	93	127	27	125	1.3	30	179
64	DH-E084	95	102	86	128	26	195	1.3	25	120
CHK1	Chk 1 (WH147)	95	97	115	124	27	188	1.3	29	163
CHK2	Chk 2 (PBW175)	95	97	127	124	27	156	2.3	38	233
CHK3	Chk 3 (NI5439)	95	98	110	123	25	172	2.3	50	120
65	DH-E086	95	92	92	121	29	122	1.9	32	177
66	DH-E087	95	88	92	121	33	135	1.6	33	115
67	DH-E088	95	96	90	122	26	195	1.9	33	285

68	DH-E090	95	90	87	121	31	162	1.3	26	255
69	DH-E091	95	84	83	122	38	176	1.1	27	177
70	DH-E092	95	100	83	127	27	145	1.5	33	244
71	DH-E093	95	87	84	121	34	182	1.2	26	272
72	DH-E094	95	104	90	128	24	145	0.9	19	153
73	DH-E095	95	94	98	121	27	176	2.0	32	158
74	DH-E096	95	99	92	125	26	164	1.7	27	185
75	DH-E097	95	86	105	121	35	142	1.4	34	197
76	DH-E098	95	86	105	121	35	146	1.6	37	273
77	DH-E099	90	88	84	122	34	116	1.0	25	225
78	DH-E100	95	102	87	126	24	139	1.1	23	218
79	DH-E102	95	91	98	121	30	180	1.9	29	171
80	DH-E103	95	92	100	121	29	195	0.9	24	262
CHK1	Chk 1 (WH147)	95	94	126	122	28	121	1.6	36	295
CHK2	Chk 2 (PBW175)	95	94	132	122	28	115	2.4	45	255
CHK3	Chk 3 (NI5439)	85	96	110	123	27	101	1.8	52	192
81	DH-E104	95	98	93	125	27	148	1.8	34	236
82	DH-E105	95	89	88	121	32	184	1.9	33	197
83	DH-E108	90	94	98	122	28	115	1.3	34	291
84	DH-E109	95	93	76	121	28	95	1.4	28	153
85	DH-E110	95	98	92	125	27	86	1.6	29	202
86	DH-E111	90	90	88	121	31	110	1.0	27	92
87	DH-E113	95	98	86	124	26	110	1.3	33	215
88	DH-E114	85	102	87	126	24	85	1.1	24	126
89	DH-E115	95	102	90	126	24	116	1.2	27	184
90	DH-E117	95	100	84	125	25	85	1.4	29	147
91	DH-E118	90	100	90	125	25	108	1.6	34	178
92	DH-E119	95	86	86	121	35	82	1.7	37	162
93	DH-E120	95	102	82	127	25	102	1.2	35	214
94	DH-E121	95	98	84	125	27	88	1.1	27	215
95	DH-E122	90	97	87	125	28	110	1.5	33	351
96	DH-E123	90	98	90	125	27	110	1.5	32	285
CHK1	Chk 1 (WH147)	95	97	114	124	27	105	1.7	38	157
CHK2	Chk 2 (PBW175)	95	97	130	124	27	81	2.6	46	137
CHK3	Chk 3 (NI5439)	75	97	112	124	27	121	2.2	52	182
97	DH-E124	95	90	86	121	31	96	1.4	32	160
98	DH-E125	95	86	85	121	35	95	1.5	30	303
99	DH-E126	95	102	82	127	25	120	1.1	29	163
100	DH-E127	95	99	86	126	27	129	1.1	29	145
101	DH-E129	95	102	87	126	24	151	1.7	35	180
102	DH-E130	95	104	82	127	23	151	1.8	34	128
103	DH-E131	90	91	83	121	30	112	1.2	26	250
104	DH-E132	95	98	74	125	27	118	1.5	31	164
105	DH-E133	85	98	78	125	27	131	1.1	24	220
106	DH-E134	95	102	79	126	24	142	1.0	27	176
107	DH-E135	95	92	73	122	30	137	1.1	28	261
108	DH-E137	95	99	84	125	26	175	1.2	30	199
109	DH-E138	90	94	86	121	27	163	2.4	37	155
110	DH-E141	95	89	78	121	32	130	1.3	26	170
111	DH-E144	95	99	86	125	26	156	1.4	34	160

112	DH-E145	90	99	82	125	26	134	1.3	29	153
CHK1	Chk 1 (WH147)	95	97	115	122	25	158	1.4	31	260
CHK2	Chk 2 (PBW175)	95	96	133	123	27	160	2.4	45	204
CHK3	Chk 3 (NI5439)	95	96	110	123	27	175	1.5	45	172
113	DH-E146	95	98	80	127	29	130	1.7	25	145
114	DH-E147	90	92	80	122	30	120	1.5	33	255
115	DH-E148	95	98	90	124	26	164	1.8	34	354
116	DH-E149	95	96	78	123	27	170	1.4	32	255
117	DH-E150	95	95	82	122	27	140	1.3	27	334
118	DH-E152	95	92	82	121	29	145	1.5	35	294
119	DH-E153	95	100	88	121	21	170	1.3	22	149
120	DH-E154	95	95	92	122	27	135	1.7	32	184
121	DH-E155	95	92	85	121	29	190	1.4	33	211
122	DH-E156	90	98	78	124	26	178	1.3	33	161
123	DH-E158	95	92	86	121	29	140	1.9	29	226
124	DH-E159	90	85	72	121	36	175	1.3	36	239
125	DH-E161	95	85	84	121	36	144	1.5	30	204
126	DH-E162	95	110	100	131	21	176	2.1	37	183
127	DH-E164	95	96	75	122	26	188	1.1	21	220
128	DH-E165	95	94	77	122	28	142	0.9	22	196
CHK1	Chk 1 (WH147)	95	94	120	122	28	111	1.6	36	172
CHK2	Chk 2 (PBW175)	95	94	132	122	28	102	2.3	46	256
CHK3	Chk 3 (NI5439)	90	95	112	122	27	135	1.7	47	189
129	DH-E166	95	102	80	125	23	155	1.4	31	260
130	DH-E167	95	102	86	125	23	140	1.3	27	140
131	DH-E168	95	102	90	125	23	135	1.6	37	165
132	DH-E169	95	98	84	122	24	125	1.4	33	230
133	DH-E170	95	92	120	121	29	130	1.8	38	273
134	DH-E171	95	95	92	123	28	110	1.9	36	169
135	DH-E172	95	89	87	121	32	122	1.8	34	279
136	DH-E175	95	90	81	121	31	165	2.0	38	162
137	DH-E176	95	89	84	121	32	120	1.7	31	241
138	DH-E177	95	92	87	121	29	61	2.2	37	234
139	DH-E178	95	92	83	121	29	98	1.8	44	266
140	DH-E179	95	92	88	121	29	85	1.4	36	244
141	DH-E180	95	92	95	121	29	128	1.7	31	201
142	DH-E181	95	98	85	123	25	94	2.3	38	231
143	DH-E182	95	98	84	123	25	86	1.4	33	170
144	DH-E183	95	89	78	121	32	82	1.4	32	137
CHK1	Chk 1 (WH147)	95	98	105	124	26	126	1.9	53	281
CHK2	Chk 2 (PBW175)	95	101	88	126	25	96	1.2	25	358
CHK3	Chk 3 (NI5439)	95	91	88	121	30	85	1.5	29	227
145	DH-E184	95	94	83	122	28	98	1.4	34	99
146	DH-E185	95	92	88	121	29	105	1.9	39	172
147	DH-E186	95	99	93	124	25	115	1.9	33	154
148	DH-E187	95	93	82	121	28	112	1.2	25	145
149	DH-E188	95	99	82	125	26	96	1.2	29	241
150	DH-E189	95	98	82	124	26	120	1.4	31	217
151	DH-E190	95	98	88	124	26	131	1.6	33	293
152	DH-E191	95	88	75	121	33	90	1.1	23	360

153	DH-E192	95	92	88	121	29	121	1.7	29	333
154	DH-E193	95	90	84	121	31	91	1.2	26	360
155	DH-E194	95	92	76	121	29	110	1.7	34	516
156	DH-E195	95	98	114	124	26	122	1.3	32	244
157	DH-E196	95	98	120	124	26	112	2.9	45	260
158	DH-E197	75	89	80	121	32	82	1.4	31	296
159	DH-E198	95	95	88	122	27	105	1.5	28	302
160	DH-E199	95	95	83	122	27	72	1.6	30	302
CHK1	Chk 1 (WH147)	95	98	116	124	26	138	1.6	32	209
CHK2	Chk 2 (PBW175)	95	96	126	123	27	131	2.5	43	314
CHK3	Chk 3 (NI5439)	95	97	105	124	27	142	1.8	45	231
161	DH-E200	95	102	76	125	23	98	1.7	27	225
162	DH-E202	95	92	92	121	29	105	1.8	29	192
163	DH-E203	95	92	85	121	29	122	1.3	26	332
164	DH-E204	95	99	85	125	26	142	1.2	22	190
165	DH-E205	95	94	90	121	27	176	2.2	33	281
166	DH-E206	95	95	76	122	27	140	1.1	28	285
167	DH-E207	95	95	80	122	27	156	2.4	41	281
168	DH-E208	95	96	88	123	27	176	1.6	28	230
169	DH-E209	95	88	88	121	33	146	1.5	28	258
170	DH-E210	95	92	88	121	29	135	1.3	31	304
171	DH-E211	90	85	80	121	36	120	1.8	35	230
172	DH-E213	95	90	85	121	31	125	1.8	33	333
173	DH-E214	95	81	87	134	53	175	1.4	29	257
174	DH-E215	95	104	84	132	28	144	1.8	29	163
175	DH-E216	95	82	87	132	50	132	1.4	25	249
176	DH-E217	95	89	75	121	32	154	1.5	34	354
CHK1	Chk 1 (WH147)	95	98	128	124	26	190	1.7	37	150
CHK2	Chk 2 (PBW175)	95	98	100	124	26	196	2.0	42	118
CHK3	Chk 3 (NI5439)	95	98	100	124	26	168	1.6	40	210
177	DH-E218	85	99	88	125	26	175	1.5	29	156
178	DH-E219	95	102	90	126	24	98	1.5	31	344
179	DH-E220	95	100	90	127	27	110	2.0	32	227
180	DH-E221	95	99	88	128	29	112	1.5	37	217
181	DH-E222	95	99	95	128	29	108	2.3	33	303
182	DH-E223	95	95	75	122	27	111	1.4	34	225
183	DH-E224	95	96	75	123	27	132	1.3	33	147
184	DH-E226	95	94	142	122	28	107	2.3	31	188
185	DH-E227	95	94	92	122	28	147	1.1	27	184
186	DH-E228	95	95	90	123	28	135	1.8	36	134
187	DH-E229	80	93	90	124	31	170	1.5	32	195
188	DH-E230	95	89	90	121	32	140	1.5	32	160
189	DH-E231	85	89	77	121	32	133	1.6	38	160
190	DH-E232	95	98	87	124	26	144	1.8	40	239
191	DH-E233	95	89	92	121	32	155	2.0	39	265
192	DH-E233	95	98	86	124	26	130	1.7	41	140
**Location**	Karnal									
**Crop-season**	2010-11									
**Condition**	Irrigated									
S.No.	**DH-2 Line code**	**GP***	**DTA**	**PH***	**DTM**	**GFD**	**PTPM**	**GWPE**	**TGW***	**GYPP**

CHK1	Chk 1 (WH147)	95	95	113	126	31	132	1.2	24	315
CHK2	Chk 2 (PBW175)	95	95	109	127	32	128	2.6	47	427
CHK3	Chk 3 (NI5439)	90	96	108	127	31	140	2.0	45	636
1	DH-E001	95	92	84	124	32	130	1.2	26	422
2	DH-E003	95	102	99	130	28	132	1.9	35	437
3	DH-E005	95	92	75	124	32	144	0.9	19	314
4	DH-E006	95	95	83	124	29	178	1.3	24	327
5	DH-E007	90	103	87	128	25	138	1.6	28	322
6	DH-E008	90	88	84	123	35	126	1.8	40	327
7	DH-E009	90	104	92	128	24	111	1.9	39	439
8	DH-E010	90	103	97	128	25	139	1.0	26	383
9	DH-E014	90	96	92	124	28	133	1.4	31	428
10	DH-E015	90	89	88	127	38	138	1.6	32	466
11	DH-E018	90	87	88	125	38	113	1.7	34	349
12	DH-E019	90	92	87	129	37	136	0.9	23	392
13	DH-E020	90	90	92	124	34	125	1.8	29	427
14	DH-E021	80	89	90	125	36	132	2.1	33	526
15	DH-E022	80	95	75	126	31	122	1.4	25	330
16	DH-E023	90	104	86	130	26	135	1.5	26	392
CHK1	Chk 1 (WH147)	95	97	120	126	29	195	1.9	45	420
CHK2	Chk 2 (PBW175)	95	97	112	126	29	170	2.2	44	224
CHK3	Chk 3 (NI5439)	95	97	111	128	31	175	1.9	51	744
17	DH-E024	95	88	75	123	35	152	1.5	32	433
18	DH-E025	90	88	80	125	37	149	1.6	33	433
19	DH-E026	95	104	90	131	27	118	1.6	30	520
20	DH-E029	95	104	85	130	26	220	1.6	29	526
21	DH-E032	90	97	82	127	30	130	1.5	32	424
22	DH-E033	85	89	89	123	34	150	1.8	36	514
23	DH-E034	80	88	84	122	34	111	0.8	16	480
24	DH-E036	85	92	85	124	32	135	1.3	34	478
25	DH-E038	90	104	88	130	26	151	1.5	34	336
26	DH-E039	90	95	92	123	28	155	1.5	34	441
27	DH-E040	90	103	90	130	27	210	1.3	22	336
28	DH-E041	85	97	85	126	29	180	2.1	44	480
29	DH-E042	80	104	92	127	23	157	1.4	28	615
30	DH-E043	80	97	87	125	28	132	1.8	32	377
31	DH-E044	85	104	85	126	22	220	1.7	29	338
32	DH-E046	90	102	95	127	25	105	1.4	31	368
CHK1	Chk 1 (WH147)	90	96	88	129	33	140	0.9	26	456
CHK2	Chk 2 (PBW175)	95	96	97	126	30	145	1.4	31	301
CHK3	Chk 3 (NI5439)	85	97	87	123	26	175	1.6	37	430
33	DH-E047	95	102	95	125	23	175	1.0	25	410
34	DH-E051	90	96	80	122	26	160	1.2	26	361
35	DH-E052	95	92	87	123	31	165	1.5	31	368
36	DH-E053	95	102	90	127	25	140	1.6	32	313
37	DH-E054	85	102	85	127	25	165	1.4	30	281
38	DH-E055	80	98	82	127	29	150	1.5	33	352
39	DH-E056	90	103	93	129	26	165	1.2	29	332
40	DH-E058	90	98	89	131	33	149	1.6	35	474
41	DH-E059	95	104	93	123	19	184	1.6	35	364

42	DH-E060	90	92	90	124	32	200	1.5	31	383
43	DH-E061	95	90	108	125	35	180	1.4	34	329
44	DH-E062	85	88	128	125	37	154	2.6	43	432
45	DH-E063	80	92	110	124	32	147	2.0	50	432
46	DH-E065	80	89	72	123	34	140	2.2	41	243
47	DH-E066	90	102	76	127	25	151	2.1	39	433
48	DH-E067	85	98	87	126	28	147	2.2	41	452
CHK1	Chk 1 (WH147)	90	96	108	124	28	186	1.5	33	282
CHK2	Chk 2 (PBW175)	90	96	120	123	27	178	2.2	42	303
CHK3	Chk 3 (NI5439)	95	96	100	123	27	160	1.5	45	572
49	DH-E068	85	87	90	124	37	110	1.6	32	367
50	DH-E069	95	92	94	124	32	185	1.5	31	440
51	DH-E070	95	102	78	128	26	151	0.9	23	195
52	DH-E071	90	90	80	125	35	108	1.3	31	362
53	DH-E072	90	90	90	125	35	140	1.4	26	432
54	DH-E073	95	94	87	127	33	185	1.5	31	370
55	DH-E074	95	103	93	126	23	182	1.5	29	220
56	DH-E075	90	97	89	123	26	172	1.8	30	319
57	DH-E076	95	89	77	125	36	160	1.4	29	314
58	DH-E077	95	86	82	122	36	138	1.8	38	455
59	DH-E078	95	96	83	122	26	195	1.7	28	472
60	DH-E080	90	95	89	124	29	170	1.4	28	453
61	DH-E081	95	103	93	129	26	166	1.3	24	270
62	DH-E082	90	95	95	124	29	124	1.3	30	397
63	DH-E083	95	101	93	128	27	170	1.3	30	435
64	DH-E084	90	102	86	130	28	183	1.3	25	385
CHK1	Chk 1 (WH147)	95	96	115	126	30	191	1.3	29	442
CHK2	Chk 2 (PBW175)	95	96	127	126	30	148	2.3	38	270
CHK3	Chk 3 (NI5439)	90	97	110	125	28	111	2.3	50	474
65	DH-E086	90	91	92	123	32	132	1.9	32	401
66	DH-E087	95	87	92	122	35	145	1.6	33	423
67	DH-E088	95	97	90	123	26	120	1.9	33	385
68	DH-E090	90	92	87	123	31	147	1.3	26	573
69	DH-E091	95	86	83	124	38	112	1.1	27	521
70	DH-E092	95	103	83	128	25	161	1.5	33	300
71	DH-E093	90	88	84	122	34	155	1.2	26	375
72	DH-E094	85	103	90	128	25	165	0.9	19	403
73	DH-E095	90	94	98	123	29	130	2.0	32	617
74	DH-E096	80	102	92	126	24	185	1.7	27	336
75	DH-E097	85	86	105	122	36	150	1.4	34	333
76	DH-E098	85	86	105	123	37	120	1.6	37	384
77	DH-E099	95	88	84	124	36	166	1.0	25	519
78	DH-E100	90	102	87	127	25	170	1.1	23	358
79	DH-E102	95	95	98	123	28	124	1.9	29	381
80	DH-E103	90	95	100	122	27	145	0.9	24	417
CHK1	Chk 1 (WH147)	95	97	126	124	27	165	1.6	36	481
CHK2	Chk 2 (PBW175)	95	95	132	124	29	185	2.4	45	325
CHK3	Chk 3 (NI5439)	90	96	110	125	29	104	1.8	52	427
81	DH-E104	95	102	93	126	24	115	1.8	34	432
82	DH-E105	95	89	88	122	33	170	1.9	33	493

83	DH-E108	90	92	98	123	31	105	1.3	34	516
84	DH-E109	95	94	76	122	28	137	1.4	28	327
85	DH-E110	95	96	92	126	30	170	1.6	29	486
86	DH-E111	90	89	88	122	33	150	1.0	27	400
87	DH-E113	95	96	86	126	30	118	1.3	33	361
88	DH-E114	95	102	87	127	25	195	1.1	24	369
89	DH-E115	90	103	90	127	24	165	1.2	27	442
90	DH-E117	95	102	84	126	24	175	1.4	29	405
91	DH-E118	95	102	90	126	24	168	1.6	34	393
92	DH-E119	95	86	86	122	36	112	1.7	37	480
93	DH-E120	95	103	82	128	25	178	1.2	35	409
94	DH-E121	90	98	84	127	29	128	1.1	27	415
95	DH-E122	85	98	87	127	29	120	1.5	33	423
96	DH-E123	95	98	90	127	29	147	1.5	32	493
CHK1	Chk 1 (WH147)	95	95	114	125	30	187	1.7	38	284
CHK2	Chk 2 (PBW175)	95	95	130	125	30	154	2.6	46	296
CHK3	Chk 3 (NI5439)	95	96	112	125	29	182	2.2	52	526
97	DH-E124	85	87	86	122	35	110	1.4	32	478
98	DH-E125	90	86	85	122	36	185	1.5	30	405
99	DH-E126	80	104	82	128	24	125	1.1	29	309
100	DH-E127	95	102	86	127	25	175	1.1	29	446
101	DH-E129	75	102	87	127	25	59	1.7	35	426
102	DH-E130	90	104	82	128	24	158	1.8	34	403
103	DH-E131	80	90	83	122	32	147	1.2	26	221
104	DH-E132	90	97	74	126	29	139	1.5	31	352
105	DH-E133	95	98	78	126	28	140	1.1	24	371
106	DH-E134	95	102	79	127	25	151	1.0	27	367
107	DH-E135	90	92	73	123	31	125	1.2	29	346
108	DH-E137	90	95	84	125	30	147	1.2	30	344
109	DH-E138	85	94	86	121	27	146	2.4	37	407
110	DH-E141	90	90	78	122	32	145	1.3	26	452
111	DH-E144	90	102	86	126	24	148	1.4	34	433
112	DH-E145	95	102	82	126	24	136	1.3	29	293
CHK1	Chk 1 (WH147)	90	96	115	122	26	175	1.4	31	727
CHK2	Chk 2 (PBW175)	95	96	133	124	28	139	2.4	45	496
CHK3	Chk 3 (NI5439)	90	97	110	124	27	140	1.5	45	499
113	DH-E146	90	98	80	127	29	159	1.7	25	422
114	DH-E147	85	96	80	123	27	138	1.5	33	455
115	DH-E148	90	98	90	125	27	110	1.8	34	388
116	DH-E149	90	98	78	125	27	124	1.4	32	416
117	DH-E150	90	95	82	125	30	108	1.3	27	357
118	DH-E152	95	92	82	122	30	142	1.5	35	317
119	DH-E153	95	102	88	122	20	137	1.3	22	288
120	DH-E154	95	102	92	124	22	126	1.7	32	290
121	DH-E155	90	89	85	123	34	136	1.4	33	483
122	DH-E156	85	93	78	125	32	115	1.3	33	376
123	DH-E158	90	92	86	123	31	126	1.9	29	521
124	DH-E159	85	86	72	123	37	120	1.3	36	302
125	DH-E161	85	86	84	123	37	128	1.5	30	445
126	DH-E162	95	108	100	124	16	140	2.1	37	488

127	DH-E164	95	98	75	124	26	138	1.1	21	285
128	DH-E165	90	95	77	124	29	122	0.9	22	426
CHK1	Chk 1 (WH147)	95	94	120	125	31	168	1.6	36	467
CHK2	Chk 2 (PBW175)	95	95	132	125	30	136	2.3	46	349
CHK3	Chk 3 (NI5439)	90	96	112	125	29	141	1.7	47	553
129	DH-E166	95	102	80	125	23	135	1.4	31	449
130	DH-E167	95	101	86	125	24	190	1.3	27	384
131	DH-E168	90	102	90	126	24	110	1.6	37	346
132	DH-E169	90	102	84	123	21	101	1.4	33	531
133	DH-E170	95	94	120	122	28	200	1.8	38	607
134	DH-E171	90	94	92	124	30	155	1.9	36	363
135	DH-E172	95	89	87	123	34	150	1.8	34	474
136	DH-E175	90	91	81	123	32	126	2.0	38	485
137	DH-E176	90	90	84	123	33	125	1.7	31	478
138	DH-E177	95	102	87	122	20	27	2.2	37	425
139	DH-E178	95	92	83	122	30	132	1.8	44	434
140	DH-E179	90	89	88	122	33	112	1.4	36	413
141	DH-E180	95	89	95	122	33	147	1.7	31	554
142	DH-E181	95	95	85	124	29	155	2.3	38	387
143	DH-E182	95	94	84	124	30	170	1.4	33	361
144	DH-E183	85	89	78	123	34	156	1.4	32	412
CHK1	Chk 1 (WH147)	95	95	105	127	32	165	1.9	53	219
CHK2	Chk 2 (PBW175)	95	95	88	128	33	160	1.2	25	377
CHK3	Chk 3 (NI5439)	90	96	88	124	28	173	1.5	29	622
145	DH-E184	80	89	83	124	35	150	1.4	34	492
146	DH-E185	90	92	88	123	31	112	1.9	39	511
147	DH-E186	95	96	93	125	29	131	1.9	33	312
148	DH-E187	95	92	82	121	29	155	1.2	25	506
149	DH-E188	90	102	82	127	25	180	1.2	29	335
150	DH-E189	95	95	82	125	30	185	1.4	31	443
151	DH-E190	90	95	88	126	31	129	1.6	33	554
152	DH-E191	80	87	75	122	35	142	1.1	23	390
153	DH-E192	90	92	88	122	30	150	1.7	29	455
154	DH-E193	95	89	84	122	33	175	1.2	26	494
155	DH-E194	95	92	76	122	30	121	1.7	34	429
156	DH-E195	95	102	114	126	24	195	1.3	32	516
157	DH-E196	85	92	120	126	34	188	2.9	45	314
158	DH-E197	85	89	80	122	33	120	1.4	31	258
159	DH-E198	90	96	88	123	27	124	1.5	28	491
160	DH-E199	80	96	83	123	27	118	1.6	30	404
CHK1	Chk 1 (WH147)	95	96	116	125	29	236	1.6	32	361
CHK2	Chk 2 (PBW175)	95	96	126	126	30	242	2.5	43	261
CHK3	Chk 3 (NI5439)	70	96	105	124	28	130	1.8	45	592
161	DH-E200	90	102	76	124	22	98	1.7	27	363
162	DH-E202	90	92	92	123	31	138	1.8	29	444
163	DH-E203	90	92	85	123	31	124	1.3	26	428
164	DH-E204	90	101	85	126	25	137	1.2	22	216
165	DH-E205	90	96	90	123	27	142	2.2	33	384
166	DH-E206	90	92	76	124	32	135	1.1	28	297
167	DH-E207	85	92	80	125	33	135	2.4	41	642

168	DH-E208	90	95	88	124	29	173	1.6	28	454
169	DH-E209	85	86	88	122	36	142	1.5	28	490
170	DH-E210	90	91	88	122	31	150	1.3	31	371
171	DH-E211	85	85	80	122	37	175	1.8	35	423
172	DH-E213	90	89	85	122	33	185	1.8	33	535
173	DH-E214	80	108	87	134	26	167	1.4	29	407
174	DH-E215	90	103	84	132	29	240	1.8	29	401
175	DH-E216	90	108	87	132	24	210	1.4	25	413
176	DH-E217	80	89	75	122	33	145	1.5	34	288
CHK1	Chk 1 (WH147)	95	96	128	126	30	170	1.7	37	455
CHK2	Chk 2 (PBW175)	95	96	100	127	31	169	2.0	42	330
CHK3	Chk 3 (NI5439)	90	97	100	127	30	198	1.6	40	533
177	DH-E218	75	96	88	126	30	126	1.5	29	641
178	DH-E219	90	104	90	128	24	130	1.5	31	322
179	DH-E220	95	102	90	129	27	172	2.0	32	429
180	DH-E221	95	107	88	132	25	158	1.5	37	329
181	DH-E222	85	96	95	132	36	130	2.3	33	487
182	DH-E223	90	95	75	126	31	134	1.4	34	410
183	DH-E224	90	95	75	124	29	144	1.3	33	407
184	DH-E226	95	95	142	124	29	121	2.3	31	549
185	DH-E227	90	95	92	124	29	148	1.1	27	256
186	DH-E228	95	98	90	125	27	167	1.8	36	437
187	DH-E229	90	90	90	126	36	117	1.5	32	467
188	DH-E230	85	94	90	122	28	151	1.5	32	426
189	DH-E231	80	87	77	122	35	156	1.6	38	374
190	DH-E232	85	96	87	125	29	182	1.8	40	597
191	DH-E233	95	89	92	122	33	185	2.0	39	600
192	DH-E233	95	103	86	126	23	122	1.7	41	588
**Location**	Pune									
**Crop-season**	2010-11									
**Condition**	Rainfed									
S.No.	**DH-2 Line code**	**GP***	**DTA**	**PH***	**DTM**	**GFD**	**PTPM**	**GWPE**	**TGW***	**GYPP**
CHK1	Chk 1 (WH147)	90	63	76	96	33	100	1.5	34	324
CHK2	Chk 2 (PBW175)	90	63	95	96	33	107	1.5	41	453
CHK3	Chk 3 (NI5439)	90	75	77	100	25	75	1.0	38	315
1	DH-E001	80	57	54	89	32	87	1.1	30	176
2	DH-E003	90	62	62	92	30	72	1.2	30	253
3	DH-E005	85	57	58	86	29	97	1.2	32	242
4	DH-E006	90	63	54	97	34	86	1.3	30	222
5	DH-E007	90	74	54	98	24	78	0.8	32	228
6	DH-E008	90	50	52	83	33	95	1.0	41	215
7	DH-E009	90	63	58	96	33	96	1.2	30	199
8	DH-E010	90	61	53	92	31	48	0.9	34	189
9	DH-E014	90	57	60	91	34	96	1.6	37	304
10	DH-E015	90	50	62	83	33	65	1.7	37	263
11	DH-E018	70	50	56	82	32	46	1.4	36	172
12	DH-E019	90	61	63	91	30	70	1.3	29	212
13	DH-E020	90	55	67	83	28	69	1.7	40	274
14	DH-E021	90	57	64	97	40	122	1.1	33	234
15	DH-E022	90	61	64	102	41	76	1.2	28	109

16	DH-E023	85	61	48	110	49	59	1.2	28	132
CHK1	Chk 1 (WH147)	80	63	66	94	31	67	1.0	31	233
CHK2	Chk 2 (PBW175)	90	61	76	93	32	65	1.5	43	224
CHK3	Chk 3 (NI5439)	70	74	62	98	24	65	0.8	39	120
17	DH-E024	85	49	50	84	35	91	1.0	36	220
18	DH-E025	90	49	55	83	34	105	1.1	37	297
19	DH-E026	50	75	61	109	34	130	0.7	29	228
20	DH-E029	85	74	61	99	25	98	1.1	26	267
21	DH-E032	90	61	54	91	30	103	0.8	33	252
22	DH-E033	90	55	58	84	29	66	1.5	32	197
23	DH-E034	80	50	54	82	32	42	1.4	38	149
24	DH-E036	90	57	57	93	36	66	1.2	31	153
25	DH-E038	80	61	53	92	31	66	1.3	28	167
26	DH-E039	80	61	53	89	28	59	1.1	34	184
27	DH-E040	80	61	47	92	31	70	0.7	23	119
28	DH-E041	90	61	41	85	24	67	0.6	21	102
29	DH-E042	90	64	49	93	29	91	0.5	20	120
30	DH-E043	90	61	55	91	30	90	0.9	29	244
31	DH-E044	90	74	46	98	24	74	0.7	24	104
32	DH-E046	80	62	60	92	30	64	1.3	31	177
CHK1	Chk 1 (WH147)	90	63	70	98	35	75	1.3	37	275
CHK2	Chk 2 (PBW175)	90	63	94	97	34	84	1.3	42	291
CHK3	Chk 3 (NI5439)	55	74	74	97	23	74	1.0	42	205
33	DH-E047	90	61	58	92	31	66	1.2	30	195
34	DH-E051	80	61	56	93	32	76	1.1	38	239
35	DH-E052	85	61	56	93	32	123	1.5	36	298
36	DH-E053	80	63	60	98	35	106	1.5	31	255
37	DH-E054	90	74	51	103	29	62	1.3	32	232
38	DH-E055	85	60	55	98	38	62	1.0	30	151
39	DH-E056	90	61	62	94	33	83	1.7	34	265
40	DH-E058	90	62	64	98	36	55	1.3	34	176
41	DH-E059	80	74	57	98	24	64	0.9	32	147
42	DH-E060	90	59	59	91	32	55	1.3	40	175
43	DH-E061	90	63	62	99	36	76	1.1	32	179
44	DH-E062	90	56	60	89	33	63	1.8	40	217
45	DH-E063	90	60	63	92	32	86	1.6	38	205
46	DH-E065	85	56	53	89	33	62	0.5	34	160
47	DH-E066	80	61	60	92	31	65	1.1	38	177
48	DH-E067	70	60	59	93	33	68	1.2	39	175
CHK1	Chk 1 (WH147)	90	63	64	94	31	57	0.9	36	147
CHK2	Chk 2 (PBW175)	90	63	79	97	34	45	1.4	40	163
CHK3	Chk 3 (NI5439)	60	74	66	99	25	40	1.3	42	106
49	DH-E068	70	50	52	82	32	53	1.4	41	108
50	DH-E069	90	56	58	86	30	83	1.1	40	148
51	DH-E070	90	63	46	97	34	102	1.4	33	127
52	DH-E071	90	50	54	82	32	77	0.9	39	134
53	DH-E072	90	56	52	84	28	59	1.0	35	132
54	DH-E073	90	56	54	83	27	103	1.1	41	150
55	DH-E074	85	56	52	91	35	55	1.3	38	149
56	DH-E075	90	60	51	92	32	42	1.6	37	148

57	DH-E076	90	56	61	85	29	71	1.5	34	223
58	DH-E077	90	60	58	83	23	81	1.2	40	249
59	DH-E078	85	60	62	91	31	59	1.5	35	282
60	DH-E080	90	60	62	92	32	116	1.1	28	262
61	DH-E081	90	75	65	108	33	95	0.9	21	192
62	DH-E082	85	57	61	92	35	92	1.3	37	351
63	DH-E083	90	74	59	112	38	87	0.9	27	259
64	DH-E084	90	74	58	102	28	73	0.9	29	227
CHK1	Chk 1 (WH147)	90	63	76	95	32	88	1.1	34	416
CHK2	Chk 2 (PBW175)	75	63	91	95	32	72	1.4	42	480
CHK3	Chk 3 (NI5439)	50	75	70	98	23	102	1.3	38	254
65	DH-E086	90	59	63	89	30	68	1.4	30	258
66	DH-E087	50	51	63	90	39	84	1.6	34	220
67	DH-E088	70	61	67	99	38	93	1.0	29	279
68	DH-E090	80	54	59	86	32	75	1.3	32	298
69	DH-E091	90	52	58	83	31	79	1.7	38	302
70	DH-E092	90	74	57	96	22	84	0.9	34	275
71	DH-E093	70	54	55	86	32	76	1.3	32	238
72	DH-E094	80	75	64	108	33	91	1.0	26	184
73	DH-E095	60	75	60	97	22	67	1.2	28	282
74	DH-E096	80	61	65	92	31	78	1.8	32	272
75	DH-E097	90	55	65	83	28	59	1.5	42	257
76	DH-E098	90	51	65	84	33	69	1.6	44	226
77	DH-E099	85	55	56	91	36	72	1.3	37	217
78	DH-E100	90	63	53	96	33	55	1.0	28	164
79	DH-E102	80	60	62	92	32	80	1.8	32	243
80	DH-E103	85	60	71	91	31	76	1.4	39	240
CHK1	Chk 1 (WH147)	90	61	71	97	36	86	1.1	32	274
CHK2	Chk 2 (PBW175)	90	64	86	96	32	82	1.2	37	348
CHK3	Chk 3 (NI5439)	75	74	71	98	24	34	1.0	32	137
81	DH-E104	90	60	60	93	33	58	1.3	34	267
82	DH-E105	85	55	63	93	38	60	1.6	36	310
83	DH-E108	60	61	57	93	32	71	1.2	42	226
84	DH-E109	80	56	52	92	36	49	0.9	31	162
85	DH-E110	90	61	61	94	33	47	1.6	36	234
86	DH-E111	90	54	58	85	31	89	1.2	33	237
87	DH-E113	85	74	54	98	24	70	1.0	32	223
88	DH-E114	90	74	61	102	28	107	0.7	27	213
89	DH-E115	85	74	53	96	22	77	0.3	31	186
90	DH-E117	90	59	54	92	33	79	1.1	32	170
91	DH-E118	75	61	59	98	37	65	1.0	30	184
92	DH-E119	90	49	58	83	34	76	1.3	42	227
93	DH-E120	90	74	53	100	26	88	1.0	34	200
94	DH-E121	80	74	52	98	24	53	1.1	29	210
95	DH-E122	90	64	59	93	29	46	1.2	32	162
96	DH-E123	90	74	56	96	22	53	1.2	36	145
CHK1	Chk 1 (WH147)	90	63	75	94	31	62	1.6	38	336
CHK2	Chk 2 (PBW175)	90	63	94	96	33	56	1.5	43	309
CHK3	Chk 3 (NI5439)	75	75	75	103	28	75	1.2	43	269
97	DH-E124	90	51	56	82	31	49	1.5	36	193

98	DH-E125	90	50	60	84	34	54	1.5	39	253
99	DH-E126	70	61	58	96	35	46	1.6	39	178
100	DH-E127	90	74	57	100	26	73	1.0	32	253
101	DH-E129	70	74	56	108	34	66	1.0	29	224
102	DH-E130	85	74	56	109	35	95	0.9	26	214
103	DH-E131	90	56	53	92	36	55	0.8	34	238
104	DH-E132	90	61	59	94	33	77	1.1	31	290
105	DH-E133	90	61	58	98	37	64	1.2	33	228
106	DH-E134	90	74	57	100	26	82	1.1	30	230
107	DH-E135	80	60	60	93	33	70	1.4	35	256
108	DH-E137	90	74	55	114	40	64	0.9	31	200
109	DH-E138	90	60	60	96	36	60	1.2	32	200
110	DH-E141	90	51	56	93	42	63	1.2	33	274
111	DH-E144	90	60	57	98	38	60	1.1	36	288
112	DH-E145	50	74	61	99	25	73	0.9	14	207
CHK1	Chk 1 (WH147)	90	63	71	97	34	83	1.6	37	298
CHK2	Chk 2 (PBW175)	90	63	85	97	34	58	1.5	42	244
CHK3	Chk 3 (NI5439)	75	74	73	103	29	38	1.3	43	146
113	DH-E146	90	60	58	92	32	63	1.5	40	224
114	DH-E147	80	56	58	92	36	72	1.5	46	192
115	DH-E148	90	57	62	96	39	64	1.4	39	252
116	DH-E149	90	63	55	97	34	58	1.0	42	185
117	DH-E150	80	60	52	91	31	59	1.4	38	204
118	DH-E152	90	56	57	89	33	48	1.3	40	198
119	DH-E153	90	60	56	92	32	66	1.3	34	227
120	DH-E154	90	61	56	97	36	41	1.3	36	174
121	DH-E155	90	57	55	91	34	55	1.0	38	154
122	DH-E156	60	61	53	94	33	81	1.3	39	176
123	DH-E158	80	57	52	89	32	76	1.0	30	197
124	DH-E159	80	51	54	83	32	72	1.4	42	230
125	DH-E161	65	55	56	90	35	63	1.3	35	225
126	DH-E162	80	74	60	102	28	65	1.2	29	210
127	DH-E164	80	59	52	91	32	113	0.9	29	247
128	DH-E165	80	57	55	91	34	61	1.1	33	261
CHK1	Chk 1 (WH147)	90	63	92	99	36	67	1.5	36	486
CHK2	Chk 2 (PBW175)	90	63	102	99	36	45	1.8	43	463
CHK3	Chk 3 (NI5439)	80	74	83	102	28	91	1.3	42	292
129	DH-E166	90	61	57	93	32	49	1.0	30	238
130	DH-E167	90	59	62	92	33	83	1.2	31	296
131	DH-E168	80	75	55	99	24	61	1.1	41	152
132	DH-E169	90	61	58	93	32	75	1.1	33	260
133	DH-E170	90	63	60	94	31	100	1.2	32	329
134	DH-E171	90	59	59	93	34	84	1.1	30	263
135	DH-E172	30	55	59	89	34	55	1.1	36	213
136	DH-E175	90	59	64	91	32	116	1.4	36	359
137	DH-E176	75	54	64	91	37	62	1.4	35	298
138	DH-E177	90	61	59	103	42	94	1.2	26	226
139	DH-E178	90	56	58	92	36	69	1.1	38	272
140	DH-E179	85	55	57	89	34	82	1.1	38	305
141	DH-E180	90	55	63	86	31	55	1.5	35	321

142	DH-E181	90	64	56	97	33	92	0.8	36	284
143	DH-E182	85	63	57	97	34	106	0.7	35	267
144	DH-E183	80	55	60	93	38	78	1.6	36	232
CHK1	Chk 1 (WH147)	75	75	79	102	27	66	1.4	41	320
CHK2	Chk 2 (PBW175)	90	74	61	98	24	78	1.1	32	354
CHK3	Chk 3 (NI5439)	85	59	63	91	32	94	1.5	38	331
145	DH-E184	60	57	56	92	35	50	1.4	31	171
146	DH-E185	75	59	65	93	34	65	1.3	33	346
147	DH-E186	70	61	69	102	41	74	1.6	34	282
148	DH-E187	85	59	59	91	32	60	1.4	32	230
149	DH-E188	90	61	62	93	32	80	0.9	32	255
150	DH-E189	90	61	61	92	31	97	1.1	32	233
151	DH-E190	90	59	60	97	38	88	1.0	31	268
152	DH-E191	85	56	57	89	33	72	1.1	31	306
153	DH-E192	90	59	60	91	32	52	1.3	33	228
154	DH-E193	85	56	55	86	30	75	1.3	35	219
155	DH-E194	85	59	56	89	30	52	0.9	33	226
156	DH-E195	85	61	78	92	31	130	1.0	34	212
157	DH-E196	90	63	86	94	31	63	1.4	40	304
158	DH-E197	60	55	57	86	31	97	1.2	40	281
159	DH-E198	85	57	70	91	34	70	1.7	40	334
160	DH-E199	90	59	61	93	34	61	1.3	33	284
CHK1	Chk 1 (WH147)	90	62	77	99	37	51	1.5	38	335
CHK2	Chk 2 (PBW175)	90	63	97	99	36	80	1.9	45	358
CHK3	Chk 3 (NI5439)	60	75	77	102	27	59	1.3	46	182
161	DH-E200	85	61	58	98	37	50	0.8	28	132
162	DH-E202	80	59	59	97	38	66	1.4	36	364
163	DH-E203	70	59	61	93	34	56	1.5	36	237
164	DH-E204	80	74	56	98	24	67	1.5	28	204
165	DH-E205	90	59	62	91	32	70	1.3	35	260
166	DH-E206	90	59	59	91	32	53	1.6	39	268
167	DH-E207	90	58	58	92	34	82	2.4	39	328
168	DH-E208	90	59	58	91	32	76	1.3	31	284
169	DH-E209	90	54	58	91	37	35	1.3	34	154
170	DH-E210	90	59	61	92	33	59	1.2	40	267
171	DH-E211	90	49	58	82	33	68	1.2	39	305
172	DH-E213	90	59	63	93	34	75	1.6	38	365
173	DH-E214	75	78	60	109	31	115	0.7	16	188
174	DH-E215	90	74	61	102	28	72	1.0	28	175
175	DH-E216	80	74	64	103	29	70	0.9	28	186
176	DH-E217	90	59	61	91	32	57	1.4	40	208
CHK1	Chk 1 (WH147)	90	63	66	98	35	76	1.4	36	283
CHK2	Chk 2 (PBW175)	90	62	92	99	37	61	1.7	41	197
CHK3	Chk 3 (NI5439)	90	74	73	99	25	40	1.9	45	205
177	DH-E218	60	61	68	99	38	45	1.6	37	179
178	DH-E219	90	74	62	103	29	80	0.9	31	346
179	DH-E220	90	73	66	98	25	82	1.6	38	325
180	DH-E221	90	74	61	102	28	61	0.9	30	169
181	DH-E222	90	62	65	98	36	58	1.1	36	245
182	DH-E223	90	59	54	91	32	65	1.2	40	216

183	DH-E224	70	59	46	92	33	65	1.6	43	232
184	DH-E226	90	63	95	99	36	53	1.4	30	253
185	DH-E227	80	55	63	91	36	55	1.6	40	250
186	DH-E228	90	74	69	101	27	57	1.3	35	256
187	DH-E229	70	59	63	93	34	51	1.6	40	229
188	DH-E230	80	59	58	93	34	56	1.4	42	222
189	DH-E231	80	49	48	82	33	60	1.2	45	210
190	DH-E232	90	74	69	102	28	56	1.5	38	195
191	DH-E233	90	54	62	93	39	35	1.8	42	192
192	DH-E233	80	63	62	97	34	42	1.4	51	130
**Location**	Pune									
**Crop-season**	2010-11									
**Condition**	Irrigated									
S.No.	**DH-2 Line code**	**GP***	**DTA**	**PH***	**DTM**	**GFD**	**PTPM**	**GWPE**	**TGW***	**GYPP**
CHK1	Chk 1 (WH147)	90	67	107	104	37	153	2.0	32	508
CHK2	Chk 2 (PBW175)	70	67	113	104	37	132	2.0	37	591
CHK3	Chk 3 (NI5439)	90	75	94	110	35	212	1.6	44	623
1	DH-E001	90	64	76	102	38	169	1.3	27	518
2	DH-E003	90	68	91	108	40	156	1.8	28	569
3	DH-E005	90	61	74	104	43	175	1.8	30	521
4	DH-E006	75	65	74	115	50	139	1.1	26	406
5	DH-E007	90	80	79	116	36	132	1.1	29	496
6	DH-E008	80	55	70	100	45	146	2.1	42	556
7	DH-E009	90	67	87	104	37	165	1.7	32	590
8	DH-E010	90	77	79	110	33	222	1.2	28	453
9	DH-E014	90	61	88	106	45	204	1.7	32	645
10	DH-E015	90	58	87	101	43	158	1.5	32	567
11	DH-E018	90	55	74	100	45	90	1.9	38	507
12	DH-E019	80	57	81	99	42	119	1.5	36	529
13	DH-E020	70	57	80	100	43	120	1.4	35	528
14	DH-E021	85	61	82	101	40	151	1.5	33	469
15	DH-E022	90	64	80	104	40	119	1.5	30	371
16	DH-E023	90	65	84	105	40	166	1.6	27	401
CHK1	Chk 1 (WH147)	85	66	106	104	38	158	2.4	32	730
CHK2	Chk 2 (PBW175)	90	67	90	106	39	142	2.2	38	571
CHK3	Chk 3 (NI5439)	60	75	90	109	34	85	1.6	38	523
17	DH-E024	80	57	69	100	43	111	1.1	32	454
18	DH-E025	85	54	64	98	44	155	1.7	33	502
19	DH-E026	80	80	78	113	33	140	1.1	29	419
20	DH-E029	90	82	75	106	24	130	1.2	28	464
21	DH-E032	90	62	79	104	42	190	1.0	29	470
22	DH-E033	80	57	76	98	41	135	1.5	39	610
23	DH-E034	85	56	70	98	42	105	1.8	39	434
24	DH-E036	80	63	76	104	41	115	2.2	34	459
25	DH-E038	90	67	70	104	37	140	1.4	42	295
26	DH-E039	80	62	78	99	37	125	1.4	28	356
27	DH-E040	90	66	71	104	38	118	2.6	31	357
28	DH-E041	80	64	73	97	33	124	1.7	25	386
29	DH-E042	90	67	76	104	37	115	1.4	25	472
30	DH-E043	90	64	83	103	39	111	1.6	27	463

31	DH-E044	90	78	68	111	33	180	1.1	33	430
32	DH-E046	90	67	80	105	38	130	1.5	29	399
CHK1	Chk 1 (WH147)	90	65	95	100	35	103	1.6	27	509
CHK2	Chk 2 (PBW175)	90	66	111	99	33	109	2.0	35	448
CHK3	Chk 3 (NI5439)	90	67	94	106	39	118	1.2	41	546
33	DH-E047	90	66	82	103	37	132	1.8	29	481
34	DH-E051	90	64	77	99	35	125	1.8	32	470
35	DH-E052	90	63	75	99	36	112	1.9	33	449
36	DH-E053	90	67	75	107	40	140	1.5	31	502
37	DH-E054	90	69	73	109	40	134	1.3	27	175
38	DH-E055	85	64	75	107	43	100	1.4	28	537
39	DH-E056	85	64	88	101	37	99	1.3	25	378
40	DH-E058	80	64	84	100	36	114	2.2	29	352
41	DH-E059	90	78	66	109	31	113	1.1	26	289
42	DH-E060	90	62	69	97	35	117	1.2	34	376
43	DH-E061	90	64	72	110	46	105	1.4	29	307
44	DH-E062	90	58	76	95	37	103	1.8	36	494
45	DH-E063	85	63	74	95	32	109	1.7	34	447
46	DH-E065	80	61	67	95	34	116	1.7	34	380
47	DH-E066	90	62	83	99	37	133	1.3	36	485
48	DH-E067	80	61	81	104	43	128	1.4	35	546
CHK1	Chk 1 (WH147)	85	65	93	104	39	162	1.9	29	519
CHK2	Chk 2 (PBW175)	90	66	112	104	38	105	2.1	35	440
CHK3	Chk 3 (NI5439)	70	68	97	107	39	136	1.7	40	502
49	DH-E068	90	55	73	94	39	117	1.7	36	304
50	DH-E069	80	66	82	97	31	147	1.8	38	605
51	DH-E070	90	67	74	108	41	132	1.1	27	341
52	DH-E071	90	57	72	94	37	110	1.7	39	626
53	DH-E072	90	61	77	95	34	109	1.4	33	609
54	DH-E073	90	62	79	95	33	130	1.5	30	608
55	DH-E074	80	64	85	99	35	131	1.5	32	635
56	DH-E075	90	63	84	96	33	127	1.4	30	505
57	DH-E076	90	63	77	95	32	151	1.5	37	602
58	DH-E077	80	57	72	94	37	88	2.1	28	490
59	DH-E078	85	67	83	99	32	170	1.7	30	525
60	DH-E080	90	67	88	102	35	165	1.5	35	601
61	DH-E081	90	84	93	118	34	164	0.8	34	540
62	DH-E082	90	66	89	101	35	135	1.4	32	532
63	DH-E083	90	83	76	119	36	99	1.3	26	460
64	DH-E084	90	83	74	119	36	137	1.3	30	485
CHK1	Chk 1 (WH147)	90	67	102	110	43	156	1.7	34	630
CHK2	Chk 2 (PBW175)	90	66	118	107	41	155	2.1	42	724
CHK3	Chk 3 (NI5439)	80	75	101	112	37	148	1.8	42	703
65	DH-E086	90	66	83	96	30	127	1.6	30	510
66	DH-E087	70	57	85	102	45	219	1.7	32	490
67	DH-E088	90	67	89	110	43	168	1.3	34	530
68	DH-E090	80	58	71	100	42	164	1.3	33	484
69	DH-E091	85	57	79	94	37	149	1.5	39	659
70	DH-E092	90	78	77	112	34	211	1.3	34	568
71	DH-E093	90	58	78	96	38	191	1.6	33	580

72	DH-E094	90	79	87	119	40	209	0.8	25	391
73	DH-E095	70	66	87	102	36	128	2.3	36	880
74	DH-E096	80	64	86	104	40	117	1.8	34	461
75	DH-E097	90	56	83	94	38	140	1.8	40	511
76	DH-E098	90	56	83	93	37	119	1.3	40	560
77	DH-E099	90	59	75	95	36	122	1.8	40	528
78	DH-E100	80	58	71	104	46	139	1.3	30	352
79	DH-E102	80	61	84	94	33	101	1.6	32	409
80	DH-E103	90	63	93	96	33	92	1.8	38	551
CHK1	Chk 1 (WH147)	90	65	104	99	34	164	1.5	34	660
CHK2	Chk 2 (PBW175)	90	65	104	104	39	150	2.5	38	585
CHK3	Chk 3 (NI5439)	75	71	95	107	36	129	1.7	40	589
81	DH-E104	90	65	83	101	36	95	1.9	32	550
82	DH-E105	80	57	82	98	41	132	1.8	32	507
83	DH-E108	90	63	75	95	32	129	1.4	39	575
84	DH-E109	90	57	69	93	36	110	1.2	33	386
85	DH-E110	90	64	80	98	34	91	1.8	36	582
86	DH-E111	80	57	73	94	37	113	1.3	33	428
87	DH-E113	90	70	81	109	39	149	1.4	31	434
88	DH-E114	90	80	72	114	34	149	1.4	26	404
89	DH-E115	80	69	75	107	38	163	1.2	31	476
90	DH-E117	80	64	72	101	37	147	1.1	28	291
91	DH-E118	90	64	82	107	43	118	1.5	32	521
92	DH-E119	90	54	74	92	38	125	1.6	36	490
93	DH-E120	90	78	73	113	35	145	1.2	30	498
94	DH-E121	80	70	69	106	36	130	1.8	28	379
95	DH-E122	50	66	83	99	33	107	1.9	34	401
96	DH-E123	90	75	71	107	32	152	1.5	30	463
CHK1	Chk 1 (WH147)	85	66	104	99	33	100	1.4	31	551
CHK2	Chk 2 (PBW175)	90	66	105	101	35	135	2.2	36	478
CHK3	Chk 3 (NI5439)	70	68	95	107	39	115	1.7	40	462
97	DH-E124	85	57	71	93	36	127	1.7	30	359
98	DH-E125	90	55	68	92	37	108	1.7	34	420
99	DH-E126	70	64	73	109	45	93	1.6	33	394
100	DH-E127	90	76	67	110	34	150	1.4	28	396
101	DH-E129	90	76	70	110	34	149	1.2	30	443
102	DH-E130	85	67	69	113	46	153	1.4	26	416
103	DH-E131	75	57	69	94	37	144	1.5	30	332
104	DH-E132	90	62	73	97	35	121	1.2	26	346
105	DH-E133	80	64	65	98	34	165	1.9	26	371
106	DH-E134	90	76	66	109	33	127	1.1	28	356
107	DH-E135	90	62	69	102	40	95	1.6	28	389
108	DH-E137	90	76	72	107	31	131	1.4	28	370
109	DH-E138	80	59	81	102	43	128	1.5	34	459
110	DH-E141	90	57	75	94	37	140	1.6	30	440
111	DH-E144	80	63	76	101	38	99	1.4	30	434
112	DH-E145	85	70	70	104	34	119	1.3	28	348
CHK1	Chk 1 (WH147)	80	65	107	102	37	162	1.4	32	787
CHK2	Chk 2 (PBW175)	90	64	115	101	37	148	1.8	40	644
CHK3	Chk 3 (NI5439)	60	69	91	107	38	112	1.7	45	469

113	DH-E146	90	60	80	94	34	137	1.4	34	453
114	DH-E147	80	60	74	94	34	97	1.0	35	427
115	DH-E148	90	59	82	93	34	118	1.7	30	582
116	DH-E149	90	67	73	104	37	126	1.5	37	564
117	DH-E150	80	61	78	97	36	139	1.6	35	431
118	DH-E152	90	60	73	94	34	162	1.6	36	526
119	DH-E153	90	63	76	96	33	148	1.3	27	437
120	DH-E154	90	63	81	107	44	120	1.2	32	555
121	DH-E155	90	61	76	96	35	136	1.2	35	547
122	DH-E156	80	64	75	102	38	135	1.7	32	487
123	DH-E158	80	64	83	97	33	148	1.4	27	568
124	DH-E159	50	57	70	99	42	136	1.6	41	401
125	DH-E161	60	59	81	101	42	121	1.3	34	612
126	DH-E162	80	82	91	118	36	175	1.2	29	515
127	DH-E164	80	66	74	103	37	181	1.2	27	427
128	DH-E165	90	63	80	102	39	197	1.3	26	474
CHK1	Chk 1 (WH147)	90	67	104	110	43	180	1.0	30	331
CHK2	Chk 2 (PBW175)	90	69	114	110	41	150	1.9	39	422
CHK3	Chk 3 (NI5439)	80	76	96	111	35	133	1.5	42	554
129	DH-E166	90	70	74	110	40	152	1.0	27	490
130	DH-E167	80	70	77	110	40	131	1.5	29	389
131	DH-E168	80	83	76	120	37	85	1.4	32	409
132	DH-E169	90	69	80	107	38	190	1.2	28	356
133	DH-E170	90	67	100	110	43	180	2.1	31	538
134	DH-E171	90	68	85	109	41	144	1.3	30	410
135	DH-E172	90	57	84	94	37	146	1.4	28	368
136	DH-E175	90	62	85	97	35	152	1.8	30	456
137	DH-E176	80	57	81	96	39	155	1.6	30	544
138	DH-E177	80	56	78	112	56	176	1.2	25	361
139	DH-E178	75	61	81	97	36	146	1.6	34	406
140	DH-E179	80	57	78	95	38	138	1.2	32	529
141	DH-E180	80	57	80	94	37	130	1.7	32	211
142	DH-E181	90	72	78	107	35	182	1.3	33	278
143	DH-E182	90	70	72	109	39	145	1.5	33	440
144	DH-E183	55	57	75	102	45	180	1.8	36	413
CHK1	Chk 1 (WH147)	90	72	95	109	37	143	1.7	39	574
CHK2	Chk 2 (PBW175)	85	74	76	109	35	101	1.6	32	484
CHK3	Chk 3 (NI5439)	90	63	82	94	31	155	1.6	31	486
145	DH-E184	60	59	77	97	38	101	1.3	30	337
146	DH-E185	80	61	85	100	39	127	1.9	36	636
147	DH-E186	85	66	83	107	41	169	1.5	30	392
148	DH-E187	90	63	76	97	34	115	1.7	26	396
149	DH-E188	90	65	75	104	39	133	1.5	30	446
150	DH-E189	90	66	72	104	38	131	1.5	30	339
151	DH-E190	80	64	72	107	43	185	1.5	28	454
152	DH-E191	80	61	68	94	33	104	1.4	35	378
153	DH-E192	75	63	83	95	32	98	1.4	27	407
154	DH-E193	90	58	70	94	36	109	1.0	29	262
155	DH-E194	90	63	75	94	31	99	1.2	28	343
156	DH-E195	90	65	104	101	36	140	1.9	32	483

157	DH-E196	90	65	105	103	38	158	2.6	37	554
158	DH-E197	50	57	71	94	37	147	1.2	31	414
159	DH-E198	90	60	83	94	34	118	1.9	32	593
160	DH-E199	55	63	86	106	43	150	1.9	31	377
CHK1	Chk 1 (WH147)	90	65	97	101	36	154	1.8	32	587
CHK2	Chk 2 (PBW175)	90	66	111	104	38	118	2.4	38	562
CHK3	Chk 3 (NI5439)	50	72	101	107	35	140	1.6	43	494
161	DH-E200	90	75	71	110	35	100	1.2	27	312
162	DH-E202	90	63	79	104	41	125	1.6	34	436
163	DH-E203	90	60	81	96	36	122	1.5	30	427
164	DH-E204	90	76	79	110	34	158	1.3	22	318
165	DH-E205	90	63	80	98	35	116	1.5	32	461
166	DH-E206	90	62	83	102	40	156	1.7	38	542
167	DH-E207	90	61	81	104	43	147	1.9	34	645
168	DH-E208	90	62	80	102	40	196	1.3	28	422
169	DH-E209	80	57	85	102	45	125	1.4	32	502
170	DH-E210	90	63	84	100	37	120	1.4	35	582
171	DH-E211	90	56	72	94	38	141	1.1	30	437
172	DH-E213	75	60	78	102	42	164	1.6	33	546
173	DH-E214	60	83	76	119	36	131	0.8	22	234
174	DH-E215	90	80	71	116	36	154	1.4	22	354
175	DH-E216	90	84	76	119	35	118	1.2	25	303
176	DH-E217	90	59	72	93	34	122	1.6	32	544
CHK1	Chk 1 (WH147)	90	67	99	102	35	168	1.5	30	550
CHK2	Chk 2 (PBW175)	90	67	130	104	37	96	2.4	39	635
CHK3	Chk 3 (NI5439)	50	72	86	110	38	154	1.8	40	726
177	DH-E218	70	64	80	107	43	104	1.7	32	451
178	DH-E219	90	78	78	109	31	141	0.9	26	389
179	DH-E220	90	73	89	104	31	185	1.1	29	509
180	DH-E221	80	79	77	118	39	134	1.4	27	345
181	DH-E222	90	65	78	107	42	102	1.7	31	500
182	DH-E223	90	57	65	94	37	122	1.0	34	348
183	DH-E224	80	60	69	97	37	107	1.6	39	414
184	DH-E226	90	67	125	102	35	115	1.5	27	502
185	DH-E227	80	60	79	94	34	103	1.4	31	309
186	DH-E228	80	74	84	107	33	91	2.0	36	643
187	DH-E229	75	64	83	96	32	123	1.4	34	498
188	DH-E230	90	62	78	97	35	118	1.7	36	611
189	DH-E231	70	51	58	93	42	125	1.4	38	392
190	DH-E232	90	74	85	107	33	117	2.0	39	668
191	DH-E233	80	57	88	104	47	99	1.8	40	652
192	DH-E233	80	66	81	104	38	104	2.9	48	479
**Location**	Kanpur									
**Crop-season**	2011-12									
**Condition**	Rainfed									
**S.No.**	**DH-2 Line code**	**GP***	**DTA**	**PH**	**DTM**	**GFD**	**PTPM**	**GWPE**	**TGW***	**GYPP**
CHK1	Chk 1 (WH147)	78	75	NA	120	45	97	NA	43	NA
CHK2	Chk 2 (PBW175)	72	77	NA	121	44	118	NA	44	NA
CHK3	Chk 3 (NI5439)	78	74	NA	121	47	115	NA	44	NA
1	DH-E001	76	78	NA	118	40	77	NA	38	NA

2	DH-E003	79	75	NA	112	37	88	NA	39	NA
3	DH-E005	77	79	NA	118	39	79	NA	36	NA
4	DH-E006	73	76	NA	114	38	71	NA	37	NA
5	DH-E007	71	77	NA	115	38	68	NA	35	NA
6	DH-E008	72	78	NA	117	39	72	NA	36	NA
7	DH-E009	74	76	NA	116	40	90	NA	41	NA
8	DH-E010	73	78	NA	118	40	83	NA	40	NA
9	DH-E014	72	76	NA	116	40	84	NA	40	NA
10	DH-E015	75	76	NA	116	40	75	NA	37	NA
11	DH-E018	74	76	NA	116	40	95	NA	38	NA
12	DH-E019	70	76	NA	116	40	88	NA	36	NA
13	DH-E020	71	75	NA	115	40	85	NA	39	NA
14	DH-E021	72	73	NA	113	40	75	NA	37	NA
15	DH-E022	74	78	NA	118	40	100	NA	39	NA
16	DH-E023	73	77	NA	114	37	101	NA	41	NA
CHK1	Chk 1 (WH147)	78	78	NA	117	39	112	NA	42	NA
CHK2	Chk 2 (PBW175)	77	77	NA	119	42	115	NA	42	NA
CHK3	Chk 3 (NI5439)	77	78	NA	121	43	118	NA	43	NA
17	DH-E024	74	75	NA	114	39	99	NA	40	NA
18	DH-E025	72	72	NA	112	40	85	NA	40	NA
19	DH-E026	71	78	NA	118	40	74	NA	37	NA
20	DH-E029	76	70	NA	110	40	78	NA	39	NA
21	DH-E032	78	77	NA	117	40	78	NA	37	NA
22	DH-E033	78	70	NA	114	44	77	NA	36	NA
23	DH-E034	74	75	NA	115	40	70	NA	35	NA
24	DH-E036	75	77	NA	117	40	73	NA	35	NA
25	DH-E038	76	76	NA	116	40	78	NA	37	NA
26	DH-E039	75	82	NA	119	37	80	NA	37	NA
27	DH-E040	70	83	NA	120	37	78	NA	35	NA
28	DH-E041	71	82	NA	118	36	75	NA	36	NA
29	DH-E042	75	80	NA	117	37	72	NA	35	NA
30	DH-E043	70	78	NA	116	38	74	NA	36	NA
31	DH-E044	79	74	NA	114	40	80	NA	37	NA
32	DH-E046	78	81	NA	114	33	73	NA	36	NA
CHK1	Chk 1 (WH147)	74	76	NA	114	38	78	NA	37	NA
CHK2	Chk 2 (PBW175)	75	80	NA	116	36	72	NA	36	NA
CHK3	Chk 3 (NI5439)	70	82	NA	107	25	71	NA	36	NA
33	DH-E047	70	78	NA	113	35	72	NA	36	NA
34	DH-E051	75	86	NA	118	32	72	NA	37	NA
35	DH-E052	73	76	NA	114	38	63	NA	37	NA
36	DH-E053	72	79	NA	114	35	65	NA	37	NA
37	DH-E054	78	76	NA	113	37	70	NA	37	NA
38	DH-E055	70	78	NA	118	40	80	NA	38	NA
39	DH-E056	72	77	NA	117	40	75	NA	37	NA
40	DH-E058	78	78	NA	113	35	68	NA	37	NA
41	DH-E059	74	74	NA	114	40	72	NA	36	NA
42	DH-E060	73	78	NA	118	40	73	NA	36	NA
43	DH-E061	75	81	NA	119	38	69	NA	35	NA
44	DH-E062	72	80	NA	120	40	72	NA	36	NA
45	DH-E063	76	84	NA	118	34	68	NA	36	NA

46	DH-E065	78	78	NA	119	41	115	NA	42	NA
47	DH-E066	78	84	NA	121	37	117	NA	44	NA
48	DH-E067	73	85	NA	121	36	118	NA	42	NA
CHK1	Chk 1 (WH147)	76	81	NA	113	32	76	NA	38	NA
CHK2	Chk 2 (PBW175)	78	79	NA	118	39	107	NA	43	NA
CHK3	Chk 3 (NI5439)	77	86	NA	120	34	118	NA	44	NA
49	DH-E068	70	76	NA	118	42	65	NA	35	NA
50	DH-E069	78	74	NA	119	45	71	NA	36	NA
51	DH-E070	71	78	NA	117	39	79	NA	37	NA
52	DH-E071	74	79	NA	115	36	74	NA	35	NA
53	DH-E072	73	80	NA	114	34	75	NA	36	NA
54	DH-E073	78	81	NA	114	33	80	NA	37	NA
55	DH-E074	78	79	NA	112	33	98	NA	39	NA
56	DH-E075	75	86	NA	118	32	99	NA	40	NA
57	DH-E076	73	78	NA	112	34	71	NA	35	NA
58	DH-E077	70	76	NA	117	41	87	NA	40	NA
59	DH-E078	72	75	NA	114	39	88	NA	39	NA
60	DH-E080	78	73	NA	117	44	75	NA	38	NA
61	DH-E081	77	74	NA	114	40	74	NA	37	NA
62	DH-E082	79	75	NA	113	38	85	NA	39	NA
63	DH-E083	70	77	NA	115	38	83	NA	38	NA
64	DH-E084	74	78	NA	115	37	81	NA	38	NA
CHK1	Chk 1 (WH147)	72	70	NA	120	50	98	NA	39	NA
CHK2	Chk 2 (PBW175)	78	80	NA	121	41	109	NA	43	NA
CHK3	Chk 3 (NI5439)	77	84	NA	122	38	112	NA	44	NA
65	DH-E086	73	75	NA	115	40	82	NA	39	NA
66	DH-E087	75	74	NA	114	40	81	NA	38	NA
67	DH-E088	76	73	NA	113	40	80	NA	37	NA
68	DH-E090	74	75	NA	115	40	87	NA	40	NA
69	DH-E091	70	78	NA	118	40	82	NA	39	NA
70	DH-E092	72	68	NA	108	40	80	NA	36	NA
71	DH-E093	75	83	NA	118	35	96	NA	41	NA
72	DH-E094	70	75	NA	111	36	80	NA	40	NA
73	DH-E095	74	76	NA	113	37	78	NA	36	NA
74	DH-E096	73	78	NA	114	36	76	NA	35	NA
75	DH-E097	70	77	NA	112	35	100	NA	40	NA
76	DH-E098	78	76	NA	116	40	101	NA	41	NA
77	DH-E099	74	75	NA	115	40	76	NA	40	NA
78	DH-E100	73	76	NA	113	37	78	NA	37	NA
79	DH-E102	70	76	NA	116	40	76	NA	35	NA
80	DH-E103	74	74	NA	114	40	79	NA	36	NA
CHK1	Chk 1 (WH147)	70	77	NA	117	40	79	NA	36	NA
CHK2	Chk 2 (PBW175)	72	78	NA	120	42	107	NA	43	NA
CHK3	Chk 3 (NI5439)	77	79	NA	121	42	112	NA	44	NA
81	DH-E104	73	73	NA	113	40	84	NA	40	NA
82	DH-E105	73	74	NA	114	40	86	NA	41	NA
83	DH-E108	71	68	NA	109	41	87	NA	42	NA
84	DH-E109	72	72	NA	112	40	82	NA	39	NA
85	DH-E110	74	74	NA	114	40	83	NA	38	NA
86	DH-E111	78	77	NA	117	40	78	NA	37	NA

87	DH-E113	75	78	NA	120	42	86	NA	39	NA
88	DH-E114	70	77	NA	118	41	84	NA	38	NA
89	DH-E115	78	72	NA	112	40	78	NA	36	NA
90	DH-E117	70	74	NA	114	40	80	NA	37	NA
91	DH-E118	71	76	NA	116	40	72	NA	35	NA
92	DH-E119	74	74	NA	114	40	75	NA	36	NA
93	DH-E120	73	74	NA	115	41	74	NA	37	NA
94	DH-E121	72	76	NA	116	40	75	NA	35	NA
95	DH-E122	74	78	NA	118	40	78	NA	36	NA
96	DH-E123	78	80	NA	120	40	84	NA	39	NA
CHK1	Chk 1 (WH147)	75	74	NA	114	40	80	NA	38	NA
CHK2	Chk 2 (PBW175)	78	78	NA	118	40	117	NA	44	NA
CHK3	Chk 3 (NI5439)	75	73	NA	113	40	84	NA	39	NA
97	DH-E124	79	74	NA	114	40	74	NA	36	NA
98	DH-E125	74	76	NA	116	40	70	NA	35	NA
99	DH-E126	79	80	NA	120	40	72	NA	36	NA
100	DH-E127	78	78	NA	118	40	73	NA	37	NA
101	DH-E129	74	77	NA	117	40	75	NA	38	NA
102	DH-E130	75	76	NA	116	40	78	NA	37	NA
103	DH-E131	70	75	NA	115	40	74	NA	37	NA
104	DH-E132	70	72	NA	112	40	90	NA	41	NA
105	DH-E133	78	74	NA	114	40	86	NA	41	NA
106	DH-E134	79	76	NA	116	40	82	NA	40	NA
107	DH-E135	70	74	NA	114	40	84	NA	41	NA
108	DH-E137	73	70	NA	122	52	86	NA	40	NA
109	DH-E138	78	78	NA	118	40	96	NA	45	NA
110	DH-E141	73	76	NA	116	40	86	NA	41	NA
111	DH-E144	75	74	NA	114	40	84	NA	40	NA
112	DH-E145	70	73	NA	113	40	88	NA	41	NA
CHK1	Chk 1 (WH147)	74	76	NA	112	36	87	NA	41	NA
CHK2	Chk 2 (PBW175)	78	80	NA	118	38	117	NA	42	NA
CHK3	Chk 3 (NI5439)	77	79	NA	119	40	118	NA	43	NA
113	DH-E146	71	72	NA	112	40	82	NA	39	NA
114	DH-E147	74	70	NA	112	42	88	NA	41	NA
115	DH-E148	70	71	NA	111	40	84	NA	39	NA
116	DH-E149	75	78	NA	118	40	78	NA	37	NA
117	DH-E150	76	77	NA	117	40	78	NA	36	NA
118	DH-E152	78	76	NA	117	41	82	NA	39	NA
119	DH-E153	70	75	NA	115	40	81	NA	40	NA
120	DH-E154	71	74	NA	114	40	83	NA	39	NA
121	DH-E155	78	78	NA	120	42	114	NA	42	NA
122	DH-E156	79	76	NA	114	38	92	NA	40	NA
123	DH-E158	70	80	NA	118	38	89	NA	41	NA
124	DH-E159	78	78	NA	119	41	78	NA	36	NA
125	DH-E161	74	77	NA	117	40	70	NA	35	NA
126	DH-E162	72	76	NA	116	40	93	NA	42	NA
127	DH-E164	74	74	NA	114	40	94	NA	41	NA
128	DH-E165	79	72	NA	114	42	88	NA	40	NA
CHK1	Chk 1 (WH147)	70	79	NA	118	39	76	NA	36	NA
CHK2	Chk 2 (PBW175)	72	76	NA	118	42	74	NA	37	NA

CHK3	Chk 3 (NI5439)	74	78	NA	119	41	75	NA	36	NA
129	DH-E166	76	74	NA	115	41	72	NA	37	NA
130	DH-E167	77	76	NA	118	42	74	NA	35	NA
131	DH-E168	75	75	NA	116	41	75	NA	36	NA
132	DH-E169	70	73	NA	113	40	76	NA	35	NA
133	DH-E170	75	72	NA	114	42	72	NA	35	NA
134	DH-E171	77	70	NA	110	40	73	NA	36	NA
135	DH-E172	75	73	NA	114	41	75	NA	36	NA
136	DH-E175	74	76	NA	118	42	73	NA	36	NA
137	DH-E176	71	78	NA	118	40	74	NA	35	NA
138	DH-E177	76	77	NA	118	41	78	NA	37	NA
139	DH-E178	75	76	NA	118	42	75	NA	35	NA
140	DH-E179	73	77	NA	119	42	72	NA	35	NA
141	DH-E180	72	76	NA	115	39	73	NA	36	NA
142	DH-E181	71	75	NA	114	39	71	NA	35	NA
143	DH-E182	76	74	NA	114	40	70	NA	35	NA
144	DH-E183	75	73	NA	114	41	72	NA	36	NA
CHK1	Chk 1 (WH147)	75	81	NA	122	41	96	NA	42	NA
CHK2	Chk 2 (PBW175)	70	80	NA	118	38	80	NA	38	NA
CHK3	Chk 3 (NI5439)	78	76	NA	112	36	84	NA	39	NA
145	DH-E184	77	74	NA	113	39	74	NA	37	NA
146	DH-E185	71	78	NA	115	37	73	NA	35	NA
147	DH-E186	73	80	NA	119	39	68	NA	35	NA
148	DH-E187	71	80	NA	119	39	72	NA	37	NA
149	DH-E188	72	78	NA	117	39	74	NA	37	NA
150	DH-E189	74	77	NA	116	39	69	NA	35	NA
151	DH-E190	75	76	NA	117	41	68	NA	36	NA
152	DH-E191	76	74	NA	118	44	69	NA	36	NA
153	DH-E192	77	72	NA	114	42	72	NA	37	NA
154	DH-E193	75	70	NA	110	40	70	NA	36	NA
155	DH-E194	76	78	NA	117	39	80	NA	37	NA
156	DH-E195	78	80	NA	120	40	107	NA	43	NA
157	DH-E196	78	80	NA	120	40	114	NA	44	NA
158	DH-E197	79	75	NA	112	37	70	NA	36	NA
159	DH-E198	70	77	NA	113	36	72	NA	37	NA
160	DH-E199	71	78	NA	115	37	74	NA	37	NA
CHK1	Chk 1 (WH147)	70	76	NA	117	41	80	NA	38	NA
CHK2	Chk 2 (PBW175)	71	75	NA	114	39	80	NA	37	NA
CHK3	Chk 3 (NI5439)	78	78	NA	118	40	110	NA	44	NA
161	DH-E200	73	76	NA	114	38	78	NA	36	NA
162	DH-E202	74	75	NA	114	39	75	NA	35	NA
163	DH-E203	70	74	NA	113	39	76	NA	36	NA
164	DH-E204	71	73	NA	112	39	77	NA	37	NA
165	DH-E205	73	72	NA	111	39	74	NA	36	NA
166	DH-E206	72	70	NA	110	40	75	NA	35	NA
167	DH-E207	78	70	NA	115	45	78	NA	36	NA
168	DH-E208	75	69	NA	109	40	76	NA	37	NA
169	DH-E209	76	70	NA	111	41	76	NA	35	NA
170	DH-E210	77	72	NA	113	41	74	NA	35	NA
171	DH-E211	72	74	NA	114	40	72	NA	36	NA

172	DH-E213	74	76	NA	115	39	74	NA	35	NA
173	DH-E214	78	74	NA	114	40	73	NA	36	NA
174	DH-E215	77	74	NA	113	39	74	NA	37	NA
175	DH-E216	75	78	NA	118	40	78	NA	37	NA
176	DH-E217	78	79	NA	119	40	118	NA	44	NA
CHK1	Chk 1 (WH147)	73	76	NA	116	40	72	NA	37	NA
CHK2	Chk 2 (PBW175)	72	78	NA	118	40	74	NA	38	NA
CHK3	Chk 3 (NI5439)	75	76	NA	117	41	84	NA	39	NA
177	DH-E218	75	76	NA	115	39	103	NA	43	NA
178	DH-E219	76	70	NA	113	43	79	NA	37	NA
179	DH-E220	76	70	NA	112	42	80	NA	37	NA
180	DH-E221	72	64	NA	109	45	66	NA	35	NA
181	DH-E222	75	78	NA	112	34	82	NA	37	NA
182	DH-E223	78	69	NA	110	41	76	NA	36	NA
183	DH-E224	77	78	NA	115	37	80	NA	41	NA
184	DH-E226	78	78	NA	112	34	112	NA	44	NA
185	DH-E227	74	77	NA	110	33	92	NA	41	NA
186	DH-E228	75	78	NA	96	18	78	NA	37	NA
187	DH-E229	78	76	NA	118	42	117	NA	45	NA
188	DH-E230	70	74	NA	113	39	92	NA	41	NA
189	DH-E231	71	74	NA	114	40	80	NA	36	NA
190	DH-E232	78	78	NA	117	39	81	NA	37	NA
191	DH-E233	76	79	NA	118	39	84	NA	41	NA
192	DH-E233	74	78	NA	117	39	78	NA	38	NA
**Location**	Kanpur									
**Crop-season**	2011-12									
**Condition**	Irrigated									
**S.No.**	**DH-2 Line code**	**GP***	**DTA**	**PH**	**DTM**	**GFD**	**PTPM**	**GWPE**	**TGW***	**GYPP**
CHK1	Chk 1 (WH147)	79	77	NA	122	45	103	NA	44	NA
CHK2	Chk 2 (PBW175)	74	79	NA	123	44	122	NA	45	NA
CHK3	Chk 3 (NI5439)	79	76	NA	121	45	120	NA	46	NA
1	DH-E001	78	79	NA	121	42	89	NA	39	NA
2	DH-E003	80	77	NA	114	37	94	NA	40	NA
3	DH-E005	79	81	NA	120	39	84	NA	37	NA
4	DH-E006	76	80	NA	116	36	78	NA	38	NA
5	DH-E007	77	79	NA	118	39	76	NA	36	NA
6	DH-E008	74	80	NA	119	39	78	NA	37	NA
7	DH-E009	76	78	NA	119	41	96	NA	42	NA
8	DH-E010	77	79	NA	121	42	92	NA	41	NA
9	DH-E014	74	78	NA	119	41	92	NA	41	NA
10	DH-E015	77	77	NA	118	41	80	NA	38	NA
11	DH-E018	77	78	NA	119	41	99	NA	40	NA
12	DH-E019	73	77	NA	122	45	96	NA	37	NA
13	DH-E020	74	77	NA	119	42	92	NA	40	NA
14	DH-E021	74	75	NA	118	43	80	NA	38	NA
15	DH-E022	77	80	NA	119	39	107	NA	40	NA
16	DH-E023	76	79	NA	116	37	104	NA	42	NA
CHK1	Chk 1 (WH147)	80	80	NA	118	38	118	NA	42	NA
CHK2	Chk 2 (PBW175)	79	79	NA	121	42	121	NA	43	NA
CHK3	Chk 3 (NI5439)	78	80	NA	123	43	124	NA	44	NA

17	DH-E024	76	76	NA	116	40	101	NA	41	NA
18	DH-E025	74	74	NA	114	40	90	NA	42	NA
19	DH-E026	73	80	NA	119	39	80	NA	38	NA
20	DH-E029	78	73	NA	117	44	88	NA	41	NA
21	DH-E032	79	79	NA	122	43	84	NA	38	NA
22	DH-E033	80	74	NA	122	48	83	NA	37	NA
23	DH-E034	76	77	NA	120	43	79	NA	36	NA
24	DH-E036	77	79	NA	121	42	77	NA	37	NA
25	DH-E038	79	78	NA	119	41	86	NA	38	NA
26	DH-E039	77	83	NA	121	38	84	NA	38	NA
27	DH-E040	74	84	NA	122	38	80	NA	36	NA
28	DH-E041	75	84	NA	120	36	77	NA	37	NA
29	DH-E042	77	82	NA	119	37	74	NA	36	NA
30	DH-E043	74	80	NA	119	39	76	NA	37	NA
31	DH-E044	80	76	NA	116	40	84	NA	38	NA
32	DH-E046	80	83	NA	117	34	74	NA	37	NA
CHK1	Chk 1 (WH147)	76	78	NA	116	38	84	NA	39	NA
CHK2	Chk 2 (PBW175)	77	81	NA	118	37	78	NA	37	NA
CHK3	Chk 3 (NI5439)	72	84	NA	111	27	79	NA	38	NA
33	DH-E047	72	79	NA	114	35	74	NA	38	NA
34	DH-E051	87	88	NA	120	32	77	NA	39	NA
35	DH-E052	75	78	NA	118	40	70	NA	38	NA
36	DH-E053	74	81	NA	116	35	72	NA	39	NA
37	DH-E054	80	78	NA	115	37	76	NA	38	NA
38	DH-E055	74	80	NA	119	39	82	NA	39	NA
39	DH-E056	74	79	NA	119	40	78	NA	38	NA
40	DH-E058	80	79	NA	117	38	75	NA	39	NA
41	DH-E059	76	76	NA	116	40	74	NA	37	NA
42	DH-E060	75	80	NA	120	40	76	NA	38	NA
43	DH-E061	77	82	NA	121	39	72	NA	37	NA
44	DH-E062	74	83	NA	122	39	74	NA	37	NA
45	DH-E063	78	86	NA	120	34	74	NA	37	NA
46	DH-E065	80	80	NA	121	41	118	NA	43	NA
47	DH-E066	80	86	NA	123	37	120	NA	45	NA
48	DH-E067	75	88	NA	124	36	122	NA	43	NA
CHK1	Chk 1 (WH147)	78	84	NA	114	30	80	NA	39	NA
CHK2	Chk 2 (PBW175)	80	84	NA	120	36	111	NA	44	NA
CHK3	Chk 3 (NI5439)	79	89	NA	121	32	121	NA	45	NA
49	DH-E068	74	78	NA	120	42	78	NA	37	NA
50	DH-E069	80	76	NA	118	42	75	NA	37	NA
51	DH-E070	74	80	NA	120	40	84	NA	38	NA
52	DH-E071	76	81	NA	118	37	78	NA	36	NA
53	DH-E072	75	82	NA	117	35	78	NA	38	NA
54	DH-E073	80	84	NA	116	32	84	NA	38	NA
55	DH-E074	80	83	NA	114	31	104	NA	40	NA
56	DH-E075	77	89	NA	120	31	102	NA	41	NA
57	DH-E076	75	80	NA	114	34	75	NA	36	NA
58	DH-E077	74	78	NA	116	38	90	NA	41	NA
59	DH-E078	76	77	NA	118	41	89	NA	40	NA
60	DH-E080	80	75	NA	118	43	78	NA	39	NA

61	DH-E081	79	77	NA	116	39	76	NA	38	NA
62	DH-E082	77	77	NA	118	41	86	NA	40	NA
63	DH-E083	74	79	NA	117	38	86	NA	39	NA
64	DH-E084	76	80	NA	118	38	84	NA	39	NA
CHK1	Chk 1 (WH147)	74	74	NA	121	47	96	NA	40	NA
CHK2	Chk 2 (PBW175)	80	84	NA	122	38	112	NA	44	NA
CHK3	Chk 3 (NI5439)	79	86	NA	124	38	114	NA	45	NA
65	DH-E086	75	77	NA	116	39	83	NA	40	NA
66	DH-E087	77	74	NA	116	42	84	NA	39	NA
67	DH-E088	78	74	NA	117	43	85	NA	39	NA
68	DH-E090	76	77	NA	116	39	89	NA	41	NA
69	DH-E091	72	80	NA	119	39	86	NA	40	NA
70	DH-E092	74	72	NA	111	39	85	NA	38	NA
71	DH-E093	77	85	NA	120	35	100	NA	42	NA
72	DH-E094	77	78	NA	114	36	84	NA	41	NA
73	DH-E095	76	78	NA	114	36	82	NA	37	NA
74	DH-E096	75	80	NA	117	37	80	NA	37	NA
75	DH-E097	72	78	NA	118	40	107	NA	41	NA
76	DH-E098	80	79	NA	116	37	108	NA	42	NA
77	DH-E099	76	76	NA	116	40	79	NA	41	NA
78	DH-E100	75	79	NA	114	35	80	NA	38	NA
79	DH-E102	72	79	NA	118	39	80	NA	37	NA
80	DH-E103	76	76	NA	118	42	82	NA	38	NA
CHK1	Chk 1 (WH147)	72	79	NA	119	40	81	NA	38	NA
CHK2	Chk 2 (PBW175)	74	80	NA	122	42	111	NA	44	NA
CHK3	Chk 3 (NI5439)	79	81	NA	124	43	114	NA	45	NA
81	DH-E104	77	75	NA	117	42	86	NA	41	NA
82	DH-E105	75	77	NA	117	40	87	NA	42	NA
83	DH-E108	74	72	NA	113	41	89	NA	43	NA
84	DH-E109	76	74	NA	114	40	84	NA	40	NA
85	DH-E110	77	76	NA	117	41	86	NA	40	NA
86	DH-E111	81	79	NA	118	39	80	NA	39	NA
87	DH-E113	77	82	NA	122	40	89	NA	40	NA
88	DH-E114	74	80	NA	120	40	86	NA	40	NA
89	DH-E115	80	74	NA	114	40	84	NA	37	NA
90	DH-E117	74	76	NA	116	40	86	NA	39	NA
91	DH-E118	75	78	NA	117	39	76	NA	37	NA
92	DH-E119	76	76	NA	116	40	79	NA	38	NA
93	DH-E120	75	75	NA	118	43	78	NA	38	NA
94	DH-E121	74	78	NA	119	41	86	NA	37	NA
95	DH-E122	76	80	NA	120	40	86	NA	38	NA
96	DH-E123	80	84	NA	122	38	89	NA	40	NA
CHK1	Chk 1 (WH147)	77	76	NA	118	42	90	NA	39	NA
CHK2	Chk 2 (PBW175)	80	80	NA	120	40	118	NA	45	NA
CHK3	Chk 3 (NI5439)	77	75	NA	116	41	94	NA	40	NA
97	DH-E124	77	76	NA	116	40	84	NA	37	NA
98	DH-E125	76	77	NA	118	41	80	NA	37	NA
99	DH-E126	80	84	NA	122	38	82	NA	37	NA
100	DH-E127	80	80	NA	120	40	79	NA	38	NA
101	DH-E129	76	79	NA	119	40	81	NA	39	NA

102	DH-E130	77	79	NA	119	40	86	NA	39	NA
103	DH-E131	74	77	NA	118	41	86	NA	38	NA
104	DH-E132	72	74	NA	114	40	98	NA	42	NA
105	DH-E133	80	76	NA	116	40	96	NA	42	NA
106	DH-E134	77	78	NA	118	40	89	NA	41	NA
107	DH-E135	72	77	NA	116	39	90	NA	42	NA
108	DH-E137	75	72	NA	114	42	96	NA	41	NA
109	DH-E138	80	80	NA	120	40	107	NA	46	NA
110	DH-E141	75	77	NA	118	41	98	NA	42	NA
111	DH-E144	77	76	NA	116	40	96	NA	41	NA
112	DH-E145	74	75	NA	115	40	94	NA	42	NA
CHK1	Chk 1 (WH147)	75	77	NA	114	37	94	NA	42	NA
CHK2	Chk 2 (PBW175)	80	82	NA	120	38	120	NA	43	NA
CHK3	Chk 3 (NI5439)	79	81	NA	121	40	124	NA	44	NA
113	DH-E146	74	74	NA	114	40	90	NA	40	NA
114	DH-E147	76	74	NA	114	40	92	NA	41	NA
115	DH-E148	72	74	NA	114	40	90	NA	40	NA
116	DH-E149	77	80	NA	120	40	86	NA	39	NA
117	DH-E150	78	79	NA	121	42	84	NA	37	NA
118	DH-E152	80	78	NA	120	42	88	NA	40	NA
119	DH-E153	74	77	NA	119	42	86	NA	41	NA
120	DH-E154	74	76	NA	116	40	84	NA	40	NA
121	DH-E155	80	81	NA	122	41	120	NA	43	NA
122	DH-E156	77	79	NA	116	37	99	NA	41	NA
123	DH-E158	74	83	NA	120	37	93	NA	42	NA
124	DH-E159	80	81	NA	121	40	82	NA	37	NA
125	DH-E161	76	79	NA	119	40	78	NA	36	NA
126	DH-E162	74	78	NA	118	40	96	NA	43	NA
127	DH-E164	76	76	NA	118	42	99	NA	43	NA
128	DH-E165	77	74	NA	116	42	96	NA	41	NA
CHK1	Chk 1 (WH147)	74	81	NA	122	41	87	NA	38	NA
CHK2	Chk 2 (PBW175)	74	78	NA	120	42	89	NA	37	NA
CHK3	Chk 3 (NI5439)	76	80	NA	123	43	88	NA	37	NA
129	DH-E166	78	76	NA	117	41	84	NA	39	NA
130	DH-E167	79	78	NA	120	42	86	NA	47	NA
131	DH-E168	77	77	NA	118	41	86	NA	37	NA
132	DH-E169	74	75	NA	114	39	87	NA	36	NA
133	DH-E170	77	74	NA	117	43	84	NA	37	NA
134	DH-E171	79	73	NA	114	41	82	NA	37	NA
135	DH-E172	77	76	NA	117	41	84	NA	37	NA
136	DH-E175	76	78	NA	120	42	82	NA	37	NA
137	DH-E176	72	80	NA	121	41	86	NA	37	NA
138	DH-E177	78	79	NA	120	41	89	NA	38	NA
139	DH-E178	77	78	NA	121	43	87	NA	36	NA
140	DH-E179	75	79	NA	122	43	80	NA	37	NA
141	DH-E180	74	78	NA	119	41	82	NA	37	NA
142	DH-E181	73	76	NA	117	41	80	NA	36	NA
143	DH-E182	78	76	NA	116	40	81	NA	37	NA
144	DH-E183	77	75	NA	118	43	83	NA	37	NA
CHK1	Chk 1 (WH147)	77	83	NA	124	41	100	NA	43	NA

CHK2	Chk 2 (PBW175)	72	82	NA	120	38	89	NA	39	NA
CHK3	Chk 3 (NI5439)	80	79	NA	114	35	92	NA	40	NA
145	DH-E184	72	76	NA	117	41	84	NA	38	NA
146	DH-E185	73	80	NA	118	38	85	NA	37	NA
147	DH-E186	80	81	NA	121	40	75	NA	37	NA
148	DH-E187	74	84	NA	121	37	83	NA	39	NA
149	DH-E188	74	80	NA	120	40	86	NA	38	NA
150	DH-E189	76	79	NA	118	39	75	NA	37	NA
151	DH-E190	77	79	NA	119	40	74	NA	37	NA
152	DH-E191	78	75	NA	121	46	76	NA	38	NA
153	DH-E192	79	73	NA	118	45	77	NA	39	NA
154	DH-E193	77	74	NA	113	39	80	NA	38	NA
155	DH-E194	78	80	NA	118	38	89	NA	39	NA
156	DH-E195	80	83	NA	122	39	118	NA	44	NA
157	DH-E196	80	82	NA	123	41	119	NA	45	NA
158	DH-E197	80	77	NA	115	38	82	NA	37	NA
159	DH-E198	72	78	NA	117	39	84	NA	38	NA
160	DH-E199	74	80	NA	118	38	86	NA	38	NA
CHK1	Chk 1 (WH147)	72	80	NA	121	41	89	NA	39	NA
CHK2	Chk 2 (PBW175)	74	79	NA	118	39	92	NA	38	NA
CHK3	Chk 3 (NI5439)	80	80	NA	120	40	117	NA	45	NA
161	DH-E200	76	79	NA	116	37	90	NA	37	NA
162	DH-E202	77	76	NA	118	42	80	NA	36	NA
163	DH-E203	72	76	NA	117	41	84	NA	38	NA
164	DH-E204	74	78	NA	114	36	89	NA	38	NA
165	DH-E205	76	74	NA	114	40	85	NA	37	NA
166	DH-E206	74	72	NA	113	41	87	NA	38	NA
167	DH-E207	80	74	NA	118	44	89	NA	37	NA
168	DH-E208	77	74	NA	110	36	84	NA	38	NA
169	DH-E209	78	73	NA	114	41	84	NA	37	NA
170	DH-E210	79	74	NA	117	43	86	NA	36	NA
171	DH-E211	75	76	NA	118	42	84	NA	37	NA
172	DH-E213	76	78	NA	119	41	86	NA	39	NA
173	DH-E214	80	76	NA	116	40	84	NA	37	NA
174	DH-E215	79	76	NA	117	41	86	NA	38	NA
175	DH-E216	77	80	NA	122	42	87	NA	38	NA
176	DH-E217	81	81	NA	121	40	120	NA	45	NA
CHK1	Chk 1 (WH147)	76	79	NA	118	39	79	NA	38	NA
CHK2	Chk 2 (PBW175)	74	80	NA	120	40	82	NA	39	NA
CHK3	Chk 3 (NI5439)	77	79	NA	119	40	89	NA	40	NA
177	DH-E218	77	78	NA	118	40	110	NA	44	NA
178	DH-E219	78	74	NA	117	43	86	NA	38	NA
179	DH-E220	79	73	NA	114	41	89	NA	39	NA
180	DH-E221	74	66	NA	111	45	78	NA	38	NA
181	DH-E222	76	80	NA	114	34	84	NA	38	NA
182	DH-E223	80	72	NA	113	41	80	NA	37	NA
183	DH-E224	79	82	NA	117	35	86	NA	42	NA
184	DH-E226	80	80	NA	114	34	117	NA	45	NA
185	DH-E227	76	79	NA	117	38	98	NA	42	NA
186	DH-E228	77	80	NA	100	20	84	NA	38	NA

187	DH-E229	79	79	NA	120	41	120	NA	46	NA
188	DH-E230	72	76	NA	115	39	99	NA	42	NA
189	DH-E231	73	77	NA	117	40	89	NA	38	NA
190	DH-E232	80	80	NA	119	39	88	NA	39	NA
191	DH-E233	78	81	NA	120	39	92	NA	42	NA
192	DH-E233	76	80	NA	119	39	88	NA	39	NA
**Location**	Karnal									
**Crop-season**	2011-12									
**Condition**	Rainfed									
**S.No.**	**DH-2 Line code**	**GP***	**DTA**	**PH***	**DTM**	**GFD**	**PTPM**	**GWPE**	**TGW**	**GYPP**
CHK1	Chk 1 (WH147)	80	97	106	121	24	91	NA	NA	NA
CHK2	Chk 2 (PBW175)	75	97	157	118	21	109	NA	NA	NA
CHK3	Chk 3 (NI5439)	75	98	94	120	22	115	NA	NA	NA
1	DH-E001	85	96	89	120	24	157	NA	NA	NA
2	DH-E003	95	99	95	118	19	112	NA	NA	NA
3	DH-E005	90	97	81	120	23	123	NA	NA	NA
4	DH-E006	90	98	79	117	19	115	NA	NA	NA
5	DH-E007	85	97	75	120	23	88	NA	NA	NA
6	DH-E008	90	78	73	115	37	93	NA	NA	NA
7	DH-E009	95	98	91	117	19	110	NA	NA	NA
8	DH-E010	90	98	86	117	19	100	NA	NA	NA
9	DH-E014	80	86	84	115	29	71	NA	NA	NA
10	DH-E015	85	86	86	115	29	110	NA	NA	NA
11	DH-E018	80	87	83	121	34	58	NA	NA	NA
12	DH-E019	80	98	84	112	14	107	NA	NA	NA
13	DH-E020	75	88	83	115	27	61	NA	NA	NA
14	DH-E021	80	93	88	116	23	109	NA	NA	NA
15	DH-E022	80	97	74	120	23	11	NA	NA	NA
16	DH-E023	85	98	74	117	19	107	NA	NA	NA
CHK1	Chk 1 (WH147)	75	97	95	116	19	114	NA	NA	NA
CHK2	Chk 2 (PBW175)	80	96	113	116	20	117	NA	NA	NA
CHK3	Chk 3 (NI5439)	80	98	95	120	22	181	NA	NA	NA
17	DH-E024	90	91	69	110	19	87	NA	NA	NA
18	DH-E025	95	85	71	107	22	108	NA	NA	NA
19	DH-E026	90	103	84	122	19	101	NA	NA	NA
20	DH-E029	85	80	75	123	43	110	NA	NA	NA
21	DH-E032	80	97	82	118	21	119	NA	NA	NA
22	DH-E033	75	91	80	111	20	111	NA	NA	NA
23	DH-E034	90	91	73	112	21	100	NA	NA	NA
24	DH-E036	90	91	81	111	20	89	NA	NA	NA
25	DH-E038	80	98	82	120	22	97	NA	NA	NA
26	DH-E039	80	97	90	121	24	101	NA	NA	NA
27	DH-E040	85	98	83	123	25	111	NA	NA	NA
28	DH-E041	85	98	86	123	25	90	NA	NA	NA
29	DH-E042	90	98	83	123	25	118	NA	NA	NA
30	DH-E043	95	97	86	121	24	97	NA	NA	NA
31	DH-E044	95	102	80	123	21	106	NA	NA	NA
32	DH-E046	95	98	86	119	21	99	NA	NA	NA
CHK1	Chk 1 (WH147)	85	98	74	123	25	70	NA	NA	NA
CHK2	Chk 2 (PBW175)	90	97	75	121	24	91	NA	NA	NA

CHK3	Chk 3 (NI5439)	85	95	72	119	24	42	NA	NA	NA
33	DH-E047	90	97	85	120	23	143	NA	NA	NA
34	DH-E051	95	97	84	121	24	96	NA	NA	NA
35	DH-E052	90	95	80	119	24	101	NA	NA	NA
36	DH-E053	95	98	78	125	27	95	NA	NA	NA
37	DH-E054	90	98	72	124	26	90	NA	NA	NA
38	DH-E055	85	97	75	121	24	124	NA	NA	NA
39	DH-E056	85	98	88	124	26	96	NA	NA	NA
40	DH-E058	90	102	74	123	21	90	NA	NA	NA
41	DH-E059	90	95	83	118	23	71	NA	NA	NA
42	DH-E060	85	96	78	122	26	91	NA	NA	NA
43	DH-E061	90	97	105	122	25	75	NA	NA	NA
44	DH-E062	85	98	112	126	28	101	NA	NA	NA
45	DH-E063	85	98	93	119	21	104	NA	NA	NA
46	DH-E065	80	95	74	118	23	95	NA	NA	NA
47	DH-E066	85	98	83	119	21	96	NA	NA	NA
48	DH-E067	85	98	86	120	22	110	NA	NA	NA
CHK1	Chk 1 (WH147)	80	98	104	123	25	118	NA	NA	NA
CHK2	Chk 2 (PBW175)	80	98	112	123	25	112	NA	NA	NA
CHK3	Chk 3 (NI5439)	85	98	90	123	25	100	NA	NA	NA
49	DH-E068	90	88	70	112	24	55	NA	NA	NA
50	DH-E069	80	97	81	121	24	70	NA	NA	NA
51	DH-E070	85	98	71	123	25	102	NA	NA	NA
52	DH-E071	85	88	78	112	24	107	NA	NA	NA
53	DH-E072	75	88	81	112	24	96	NA	NA	NA
54	DH-E073	90	91	76	118	27	100	NA	NA	NA
55	DH-E074	95	97	82	121	24	97	NA	NA	NA
56	DH-E075	85	102	72	121	19	70	NA	NA	NA
57	DH-E076	90	97	62	121	24	50	NA	NA	NA
58	DH-E077	95	85	80	112	27	48	NA	NA	NA
59	DH-E078	90	86	76	112	26	51	NA	NA	NA
60	DH-E080	80	97	84	121	24	73	NA	NA	NA
61	DH-E081	90	97	82	121	24	65	NA	NA	NA
62	DH-E082	90	97	86	120	23	100	NA	NA	NA
63	DH-E083	95	97	88	120	23	40	NA	NA	NA
64	DH-E084	90	99	82	126	27	87	NA	NA	NA
CHK1	Chk 1 (WH147)	90	96	114	122	26	87	NA	NA	NA
CHK2	Chk 2 (PBW175)	85	70	117	121	51	110	NA	NA	NA
CHK3	Chk 3 (NI5439)	90	96	94	123	27	108	NA	NA	NA
65	DH-E086	95	91	83	118	27	94	NA	NA	NA
66	DH-E087	90	78	84	108	30	100	NA	NA	NA
67	DH-E088	90	83	85	107	24	76	NA	NA	NA
68	DH-E090	85	84	83	109	25	71	NA	NA	NA
69	DH-E091	90	82	85	106	24	58	NA	NA	NA
70	DH-E092	90	82	87	106	24	86	NA	NA	NA
71	DH-E093	90	84	74	104	20	115	NA	NA	NA
72	DH-E094	90	90	76	127	37	77	NA	NA	NA
73	DH-E095	85	96	85	124	28	78	NA	NA	NA
74	DH-E096	90	96	90	124	28	108	NA	NA	NA
75	DH-E097	85	82	93	108	26	70	NA	NA	NA

76	DH-E098	85	83	97	108	25	98	NA	NA	NA
77	DH-E099	80	92	85	115	23	120	NA	NA	NA
78	DH-E100	80	71	74	128	57	111	NA	NA	NA
79	DH-E102	90	95	90	122	27	155	NA	NA	NA
80	DH-E103	85	95	95	122	27	118	NA	NA	NA
CHK1	Chk 1 (WH147)	85	96	113	123	27	126	NA	NA	NA
CHK2	Chk 2 (PBW175)	80	96	123	123	27	124	NA	NA	NA
CHK3	Chk 3 (NI5439)	85	96	103	123	27	141	NA	NA	NA
81	DH-E104	80	93	95	124	31	100	NA	NA	NA
82	DH-E105	85	82	84	108	26	106	NA	NA	NA
83	DH-E108	85	95	91	121	26	75	NA	NA	NA
84	DH-E109	85	89	73	115	26	105	NA	NA	NA
85	DH-E110	85	96	87	123	27	130	NA	NA	NA
86	DH-E111	80	89	83	108	19	12	NA	NA	NA
87	DH-E113	85	100	80	124	24	133	NA	NA	NA
88	DH-E114	85	100	81	127	27	182	NA	NA	NA
89	DH-E115	90	100	89	127	27	146	NA	NA	NA
90	DH-E117	95	96	88	123	27	135	NA	NA	NA
91	DH-E118	85	96	92	123	27	144	NA	NA	NA
92	DH-E119	95	79	80	103	24	89	NA	NA	NA
93	DH-E120	90	100	81	127	27	102	NA	NA	NA
94	DH-E121	85	100	83	127	27	121	NA	NA	NA
95	DH-E122	80	99	85	124	25	97	NA	NA	NA
96	DH-E123	80	95	76	122	27	102	NA	NA	NA
CHK1	Chk 1 (WH147)	90	95	79	123	28	126	NA	NA	NA
CHK2	Chk 2 (PBW175)	90	97	77	123	26	150	NA	NA	NA
CHK3	Chk 3 (NI5439)	95	96	102	117	21	110	NA	NA	NA
97	DH-E124	75	89	87	108	19	98	NA	NA	NA
98	DH-E125	80	89	86	109	20	109	NA	NA	NA
99	DH-E126	85	95	82	122	27	92	NA	NA	NA
100	DH-E127	75	100	81	125	25	124	NA	NA	NA
101	DH-E129	80	100	82	125	25	116	NA	NA	NA
102	DH-E130	90	100	79	127	27	130	NA	NA	NA
103	DH-E131	75	89	73	114	25	94	NA	NA	NA
104	DH-E132	95	97	83	119	22	127	NA	NA	NA
105	DH-E133	90	97	80	123	26	135	NA	NA	NA
106	DH-E134	90	100	83	127	27	150	NA	NA	NA
107	DH-E135	95	93	81	118	25	80	NA	NA	NA
108	DH-E137	95	97	81	124	27	140	NA	NA	NA
109	DH-E138	95	95	89	123	28	54	NA	NA	NA
110	DH-E141	90	89	94	114	25	150	NA	NA	NA
111	DH-E144	95	87	92	113	26	126	NA	NA	NA
112	DH-E145	90	96	75	123	27	103	NA	NA	NA
CHK1	Chk 1 (WH147)	85	97	105	123	26	118	NA	NA	NA
CHK2	Chk 2 (PBW175)	80	97	123	118	21	122	NA	NA	NA
CHK3	Chk 3 (NI5439)	85	95	101	121	26	155	NA	NA	NA
113	DH-E146	85	95	97	121	26	118	NA	NA	NA
114	DH-E147	90	93	85	118	25	85	NA	NA	NA
115	DH-E148	95	95	95	121	26	168	NA	NA	NA
116	DH-E149	90	95	75	121	26	108	NA	NA	NA

117	DH-E150	90	95	81	120	25	121	NA	NA	NA
118	DH-E152	85	94	84	118	24	146	NA	NA	NA
119	DH-E153	85	97	81	121	24	154	NA	NA	NA
120	DH-E154	80	97	84	120	23	106	NA	NA	NA
121	DH-E155	80	95	88	121	26	128	NA	NA	NA
122	DH-E156	90	97	86	122	25	135	NA	NA	NA
123	DH-E158	80	84	85	120	36	63	NA	NA	NA
124	DH-E159	85	84	73	110	26	87	NA	NA	NA
125	DH-E161	90	84	80	110	26	68	NA	NA	NA
126	DH-E162	85	100	85	127	27	91	NA	NA	NA
127	DH-E164	90	95	74	121	26	99	NA	NA	NA
128	DH-E165	80	94	80	118	24	65	NA	NA	NA
CHK1	Chk 1 (WH147)	85	95	106	121	26	109	NA	NA	NA
CHK2	Chk 2 (PBW175)	85	97	111	126	29	51	NA	NA	NA
CHK3	Chk 3 (NI5439)	85	97	91	126	29	75	NA	NA	NA
129	DH-E166	85	96	82	122	26	55	NA	NA	NA
130	DH-E167	85	96	83	122	26	160	NA	NA	NA
131	DH-E168	80	97	84	126	29	90	NA	NA	NA
132	DH-E169	90	96	84	123	27	101	NA	NA	NA
133	DH-E170	90	95	104	122	27	109	NA	NA	NA
134	DH-E171	90	95	89	122	27	65	NA	NA	NA
135	DH-E172	85	96	81	113	17	65	NA	NA	NA
136	DH-E175	90	94	88	123	29	130	NA	NA	NA
137	DH-E176	90	89	81	114	25	105	NA	NA	NA
138	DH-E177	95	101	73	126	25	90	NA	NA	NA
139	DH-E178	90	84	71	110	26	68	NA	NA	NA
140	DH-E179	95	86	72	111	25	70	NA	NA	NA
141	DH-E180	95	91	81	109	18	110	NA	NA	NA
142	DH-E181	95	97	79	120	23	111	NA	NA	NA
143	DH-E182	90	97	73	122	25	100	NA	NA	NA
144	DH-E183	95	86	81	111	25	110	NA	NA	NA
CHK1	Chk 1 (WH147)	90	97	91	124	27	140	NA	NA	NA
CHK2	Chk 2 (PBW175)	90	97	89	124	27	136	NA	NA	NA
CHK3	Chk 3 (NI5439)	95	93	72	119	26	170	NA	NA	NA
145	DH-E184	90	86	82	110	24	139	NA	NA	NA
146	DH-E185	85	89	89	114	25	136	NA	NA	NA
147	DH-E186	90	94	89	124	30	11	NA	NA	NA
148	DH-E187	85	95	83	123	28	125	NA	NA	NA
149	DH-E188	90	97	92	123	26	140	NA	NA	NA
150	DH-E189	90	97	87	124	27	103	NA	NA	NA
151	DH-E190	90	98	91	126	28	135	NA	NA	NA
152	DH-E191	80	89	80	113	24	91	NA	NA	NA
153	DH-E192	85	93	91	118	25	104	NA	NA	NA
154	DH-E193	95	89	90	114	25	111	NA	NA	NA
155	DH-E194	95	95	87	124	29	135	NA	NA	NA
156	DH-E195	95	97	123	126	29	121	NA	NA	NA
157	DH-E196	80	97	106	126	29	122	NA	NA	NA
158	DH-E197	90	82	72	108	26	130	NA	NA	NA
159	DH-E198	80	94	91	118	24	156	NA	NA	NA
160	DH-E199	85	93	85	118	25	156	NA	NA	NA

CHK1	Chk 1 (WH147)	85	95	73	121	26	160	NA	NA	NA
CHK2	Chk 2 (PBW175)	85	95	105	121	26	171	NA	NA	NA
CHK3	Chk 3 (NI5439)	90	96	90	124	28	78	NA	NA	NA
161	DH-E200	85	95	64	122	27	111	NA	NA	NA
162	DH-E202	90	95	84	122	27	150	NA	NA	NA
163	DH-E203	95	83	87	112	29	183	NA	NA	NA
164	DH-E204	90	97	91	124	27	137	NA	NA	NA
165	DH-E205	85	95	89	122	27	117	NA	NA	NA
166	DH-E206	80	95	84	122	27	87	NA	NA	NA
167	DH-E207	90	89	84	112	23	121	NA	NA	NA
168	DH-E208	90	95	90	123	28	109	NA	NA	NA
169	DH-E209	95	83	87	110	27	155	NA	NA	NA
170	DH-E210	95	93	86	118	25	146	NA	NA	NA
171	DH-E211	90	78	80	108	30	113	NA	NA	NA
172	DH-E213	90	89	74	117	28	130	NA	NA	NA
173	DH-E214	80	101	73	126	25	113	NA	NA	NA
174	DH-E215	85	100	73	125	25	120	NA	NA	NA
175	DH-E216	85	100	70	127	27	101	NA	NA	NA
176	DH-E217	90	89	82	116	27	80	NA	NA	NA
CHK1	Chk 1 (WH147)	85	96	107	124	28	180	NA	NA	NA
CHK2	Chk 2 (PBW175)	80	96	110	124	28	70	NA	NA	NA
CHK3	Chk 3 (NI5439)	80	100	91	125	25	110	NA	NA	NA
177	DH-E218	85	95	92	121	26	102	NA	NA	NA
178	DH-E219	90	99	82	123	24	122	NA	NA	NA
179	DH-E220	90	99	82	123	24	143	NA	NA	NA
180	DH-E221	85	99	81	123	24	181	NA	NA	NA
181	DH-E222	80	95	82	122	27	163	NA	NA	NA
182	DH-E223	90	95	79	121	26	130	NA	NA	NA
183	DH-E224	90	95	81	121	26	160	NA	NA	NA
184	DH-E226	85	97	116	124	27	190	NA	NA	NA
185	DH-E227	85	95	89	121	26	95	NA	NA	NA
186	DH-E228	85	96	89	121	25	115	NA	NA	NA
187	DH-E229	90	95	86	121	26	50	NA	NA	NA
188	DH-E230	90	89	90	118	29	155	NA	NA	NA
189	DH-E231	80	90	78	127	37	193	NA	NA	NA
190	DH-E232	80	97	86	123	26	205	NA	NA	NA
191	DH-E233	85	83	102	110	27	115	NA	NA	NA
192	DH-E233	75	95	98	121	26	197	NA	NA	NA
**Location**	Karnal									
**Crop-season**	2011-12									
**Condition**	Irrigated									
**S.No.**	**DH-2 Line code**	**GP***	**DTA**	**PH***	**DTM**	**GFD**	**PTPM**	**GWPE**	**TGW**	**GYPP**
CHK1	Chk 1 (WH147)	80	101	107	127	26	119	NA	NA	NA
CHK2	Chk 2 (PBW175)	75	99	125	126	27	114	NA	NA	NA
CHK3	Chk 3 (NI5439)	80	101	108	128	27	95	NA	NA	NA
1	DH-E001	85	95	93	123	28	180	NA	NA	NA
2	DH-E003	80	96	103	124	28	153	NA	NA	NA
3	DH-E005	80	95	82	123	28	103	NA	NA	NA
4	DH-E006	80	99	94	126	27	126	NA	NA	NA
5	DH-E007	75	94	91	120	26	80	NA	NA	NA

6	DH-E008	85	86	97	116	30	12	NA	NA	NA
7	DH-E009	85	101	103	128	27	80	NA	NA	NA
8	DH-E010	85	101	106	128	27	115	NA	NA	NA
9	DH-E014	80	94	96	120	26	153	NA	NA	NA
10	DH-E015	75	90	100	118	28	151	NA	NA	NA
11	DH-E018	85	88	98	115	27	90	NA	NA	NA
12	DH-E019	80	101	100	128	27	130	NA	NA	NA
13	DH-E020	85	90	100	118	28	105	NA	NA	NA
14	DH-E021	85	94	100	120	26	125	NA	NA	NA
15	DH-E022	80	95	87	123	28	105	NA	NA	NA
16	DH-E023	80	101	89	128	27	85	NA	NA	NA
CHK1	Chk 1 (WH147)	75	99	103	126	27	170	NA	NA	NA
CHK2	Chk 2 (PBW175)	80	101	110	128	27	185	NA	NA	NA
CHK3	Chk 3 (NI5439)	75	101	100	128	27	175	NA	NA	NA
17	DH-E024	85	88	88	115	27	110	NA	NA	NA
18	DH-E025	85	88	85	115	27	120	NA	NA	NA
19	DH-E026	80	99	102	126	27	135	NA	NA	NA
20	DH-E029	80	100	92	126	26	243	NA	NA	NA
21	DH-E032	85	100	91	126	26	180	NA	NA	NA
22	DH-E033	85	89	100	118	29	150	NA	NA	NA
23	DH-E034	80	90	88	118	28	134	NA	NA	NA
24	DH-E036	85	88	102	116	28	150	NA	NA	NA
25	DH-E038	85	99	91	123	24	125	NA	NA	NA
26	DH-E039	85	94	100	120	26	120	NA	NA	NA
27	DH-E040	80	99	92	126	27	150	NA	NA	NA
28	DH-E041	80	99	94	123	24	130	NA	NA	NA
29	DH-E042	75	99	99	123	24	225	NA	NA	NA
30	DH-E043	75	94	99	120	26	155	NA	NA	NA
31	DH-E044	80	101	95	128	27	185	NA	NA	NA
32	DH-E046	85	100	105	126	26	185	NA	NA	NA
CHK1	Chk 1 (WH147)	85	100	88	127	27	115	NA	NA	NA
CHK2	Chk 2 (PBW175)	85	95	105	121	26	175	NA	NA	NA
CHK3	Chk 3 (NI5439)	85	95	97	122	27	135	NA	NA	NA
33	DH-E047	85	101	100	128	27	235	NA	NA	NA
34	DH-E051	85	95	100	122	27	155	NA	NA	NA
35	DH-E052	80	95	98	122	27	160	NA	NA	NA
36	DH-E053	80	99	100	125	26	130	NA	NA	NA
37	DH-E054	75	98	92	126	28	135	NA	NA	NA
38	DH-E055	80	99	94	123	24	136	NA	NA	NA
39	DH-E056	80	100	100	127	27	190	NA	NA	NA
40	DH-E058	85	96	99	124	28	230	NA	NA	NA
41	DH-E059	90	95	95	122	27	120	NA	NA	NA
42	DH-E060	90	93	96	121	28	160	NA	NA	NA
43	DH-E061	95	93	100	121	28	180	NA	NA	NA
44	DH-E062	95	96	115	124	28	130	NA	NA	NA
45	DH-E063	80	98	102	124	26	175	NA	NA	NA
46	DH-E065	80	94	86	120	26	146	NA	NA	NA
47	DH-E066	85	98	100	126	28	126	NA	NA	NA
48	DH-E067	95	98	100	124	26	129	NA	NA	NA
CHK1	Chk 1 (WH147)	95	101	107	124	23	224	NA	NA	NA

CHK2	Chk 2 (PBW175)	80	99	115	123	24	214	NA	NA	NA
CHK3	Chk 3 (NI5439)	95	100	108	126	26	234	NA	NA	NA
49	DH-E068	90	89	99	121	32	224	NA	NA	NA
50	DH-E069	80	94	99	120	26	119	NA	NA	NA
51	DH-E070	85	97	87	121	24	120	NA	NA	NA
52	DH-E071	85	89	90	121	32	165	NA	NA	NA
53	DH-E072	85	94	100	120	26	130	NA	NA	NA
54	DH-E073	95	97	94	123	26	145	NA	NA	NA
55	DH-E074	90	101	102	124	23	195	NA	NA	NA
56	DH-E075	95	101	90	127	26	120	NA	NA	NA
57	DH-E076	85	94	102	120	26	72	NA	NA	NA
58	DH-E077	90	89	93	121	32	130	NA	NA	NA
59	DH-E078	95	89	100	121	32	160	NA	NA	NA
60	DH-E080	90	95	97	121	26	100	NA	NA	NA
61	DH-E081	95	99	102	124	25	209	NA	NA	NA
62	DH-E082	90	99	104	124	25	182	NA	NA	NA
63	DH-E083	95	99	103	124	25	200	NA	NA	NA
64	DH-E084	95	95	97	123	28	215	NA	NA	NA
CHK1	Chk 1 (WH147)	85	98	105	124	26	200	NA	NA	NA
CHK2	Chk 2 (PBW175)	95	99	105	126	27	172	NA	NA	NA
CHK3	Chk 3 (NI5439)	95	100	107	127	27	210	NA	NA	NA
65	DH-E086	95	95	100	123	28	105	NA	NA	NA
66	DH-E087	95	88	102	121	33	115	NA	NA	NA
67	DH-E088	95	88	97	116	28	146	NA	NA	NA
68	DH-E090	95	89	99	118	29	135	NA	NA	NA
69	DH-E091	90	87	100	116	29	160	NA	NA	NA
70	DH-E092	95	87	96	116	29	135	NA	NA	NA
71	DH-E093	90	89	94	124	35	195	NA	NA	NA
72	DH-E094	90	94	102	123	29	248	NA	NA	NA
73	DH-E095	80	99	103	126	27	125	NA	NA	NA
74	DH-E096	85	99	100	126	27	110	NA	NA	NA
75	DH-E097	90	89	95	120	31	125	NA	NA	NA
76	DH-E098	95	88	102	120	32	125	NA	NA	NA
77	DH-E099	85	94	94	121	27	115	NA	NA	NA
78	DH-E100	85	99	96	126	27	140	NA	NA	NA
79	DH-E102	85	98	97	124	26	153	NA	NA	NA
80	DH-E103	95	99	99	124	25	135	NA	NA	NA
CHK1	Chk 1 (WH147)	80	100	103	127	27	170	NA	NA	NA
CHK2	Chk 2 (PBW175)	95	100	109	127	27	103	NA	NA	NA
CHK3	Chk 3 (NI5439)	95	101	104	128	27	195	NA	NA	NA
81	DH-E104	90	100	104	127	27	115	NA	NA	NA
82	DH-E105	90	88	100	121	33	199	NA	NA	NA
83	DH-E108	85	94	103	121	27	215	NA	NA	NA
84	DH-E109	80	94	89	121	27	183	NA	NA	NA
85	DH-E110	85	100	102	127	27	125	NA	NA	NA
86	DH-E111	90	94	105	121	27	183	NA	NA	NA
87	DH-E113	80	100	90	127	27	112	NA	NA	NA
88	DH-E114	85	99	91	126	27	215	NA	NA	NA
89	DH-E115	85	98	93	126	28	185	NA	NA	NA
90	DH-E117	80	99	91	125	26	195	NA	NA	NA

91	DH-E118	85	94	97	121	27	155	NA	NA	NA
92	DH-E119	85	86	94	113	27	115	NA	NA	NA
93	DH-E120	90	101	90	128	27	194	NA	NA	NA
94	DH-E121	90	98	92	125	27	135	NA	NA	NA
95	DH-E122	90	98	98	126	28	155	NA	NA	NA
96	DH-E123	85	98	97	126	28	147	NA	NA	NA
CHK1	Chk 1 (WH147)	90	98	110	127	29	130	NA	NA	NA
CHK2	Chk 2 (PBW175)	80	98	129	127	29	102	NA	NA	NA
CHK3	Chk 3 (NI5439)	95	101	106	128	27	188	NA	NA	NA
97	DH-E124	75	94	90	121	27	140	NA	NA	NA
98	DH-E125	85	86	91	113	27	150	NA	NA	NA
99	DH-E126	80	100	94	129	29	145	NA	NA	NA
100	DH-E127	80	101	96	128	27	140	NA	NA	NA
101	DH-E129	85	98	94	126	28	125	NA	NA	NA
102	DH-E130	80	98	95	127	29	150	NA	NA	NA
103	DH-E131	90	88	90	121	33	160	NA	NA	NA
104	DH-E132	85	100	92	126	26	135	NA	NA	NA
105	DH-E133	80	100	99	126	26	234	NA	NA	NA
106	DH-E134	90	98	100	126	28	117	NA	NA	NA
107	DH-E135	85	94	87	121	27	130	NA	NA	NA
108	DH-E137	90	101	97	128	27	103	NA	NA	NA
109	DH-E138	95	94	99	121	27	117	NA	NA	NA
110	DH-E141	85	88	91	121	33	96	NA	NA	NA
111	DH-E144	80	98	100	126	28	142	NA	NA	NA
112	DH-E145	85	101	99	128	27	150	NA	NA	NA
CHK1	Chk 1 (WH147)	80	99	104	126	27	145	NA	NA	NA
CHK2	Chk 2 (PBW175)	90	99	113	126	27	122	NA	NA	NA
CHK3	Chk 3 (NI5439)	90	101	97	128	27	135	NA	NA	NA
113	DH-E146	80	99	106	124	25	125	NA	NA	NA
114	DH-E147	80	94	103	120	26	175	NA	NA	NA
115	DH-E148	80	98	100	126	28	215	NA	NA	NA
116	DH-E149	85	98	93	126	28	190	NA	NA	NA
117	DH-E150	90	94	100	121	27	197	NA	NA	NA
118	DH-E152	90	94	96	121	27	205	NA	NA	NA
119	DH-E153	85	101	98	127	26	145	NA	NA	NA
120	DH-E154	80	101	103	127	26	196	NA	NA	NA
121	DH-E155	90	94	94	120	26	153	NA	NA	NA
122	DH-E156	95	99	100	126	27	165	NA	NA	NA
123	DH-E158	90	94	101	120	26	105	NA	NA	NA
124	DH-E159	85	85	90	115	30	145	NA	NA	NA
125	DH-E161	90	87	91	117	30	135	NA	NA	NA
126	DH-E162	90	99	104	126	27	147	NA	NA	NA
127	DH-E164	90	98	92	123	25	142	NA	NA	NA
128	DH-E165	85	94	94	120	26	121	NA	NA	NA
CHK1	Chk 1 (WH147)	90	98	110	124	26	145	NA	NA	NA
CHK2	Chk 2 (PBW175)	95	98	125	124	26	170	NA	NA	NA
CHK3	Chk 3 (NI5439)	95	99	110	126	27	135	NA	NA	NA
129	DH-E166	90	101	97	127	26	130	NA	NA	NA
130	DH-E167	95	101	94	127	26	160	NA	NA	NA
131	DH-E168	90	101	95	128	27	118	NA	NA	NA

132	DH-E169	85	99	90	126	27	130	NA	NA	NA
133	DH-E170	85	96	106	123	27	155	NA	NA	NA
134	DH-E171	80	98	99	123	25	156	NA	NA	NA
135	DH-E172	95	89	95	120	31	160	NA	NA	NA
136	DH-E175	80	94	101	120	26	135	NA	NA	NA
137	DH-E176	75	94	94	120	26	54	NA	NA	NA
138	DH-E177	90	101	98	128	27	175	NA	NA	NA
139	DH-E178	90	94	93	117	23	95	NA	NA	NA
140	DH-E179	90	93	100	117	24	53	NA	NA	NA
141	DH-E180	95	94	102	120	26	155	NA	NA	NA
142	DH-E181	90	98	95	123	25	190	NA	NA	NA
143	DH-E182	70	98	92	123	25	185	NA	NA	NA
144	DH-E183	80	93	97	117	24	146	NA	NA	NA
CHK1	Chk 1 (WH147)	80	101	103	128	27	145	NA	NA	NA
CHK2	Chk 2 (PBW175)	85	101	102	128	27	185	NA	NA	NA
CHK3	Chk 3 (NI5439)	80	94	94	120	26	95	NA	NA	NA
145	DH-E184	85	96	96	122	26	109	NA	NA	NA
146	DH-E185	85	94	106	120	26	127	NA	NA	NA
147	DH-E186	90	98	103	123	25	165	NA	NA	NA
148	DH-E187	90	94	99	120	26	146	NA	NA	NA
149	DH-E188	95	98	102	123	25	132	NA	NA	NA
150	DH-E189	90	98	97	123	25	172	NA	NA	NA
151	DH-E190	80	98	99	123	25	205	NA	NA	NA
152	DH-E191	85	94	97	120	26	165	NA	NA	NA
153	DH-E192	80	94	99	120	26	178	NA	NA	NA
154	DH-E193	80	94	95	120	26	241	NA	NA	NA
155	DH-E194	80	98	94	124	26	214	NA	NA	NA
156	DH-E195	75	98	125	124	26	115	NA	NA	NA
157	DH-E196	80	97	130	125	28	175	NA	NA	NA
158	DH-E197	85	88	91	117	29	150	NA	NA	NA
159	DH-E198	85	94	103	120	26	110	NA	NA	NA
160	DH-E199	90	94	93	120	26	113	NA	NA	NA
CHK1	Chk 1 (WH147)	75	94	107	120	26	145	NA	NA	NA
CHK2	Chk 2 (PBW175)	80	98	104	124	26	180	NA	NA	NA
CHK3	Chk 3 (NI5439)	80	98	102	124	26	232	NA	NA	NA
161	DH-E200	80	101	95	128	27	163	NA	NA	NA
162	DH-E202	80	94	95	120	26	113	NA	NA	NA
163	DH-E203	85	94	92	120	26	50	NA	NA	NA
164	DH-E204	90	98	82	123	25	130	NA	NA	NA
165	DH-E205	85	98	100	123	25	104	NA	NA	NA
166	DH-E206	90	98	91	124	26	90	NA	NA	NA
167	DH-E207	80	94	102	120	26	102	NA	NA	NA
168	DH-E208	80	94	103	120	26	110	NA	NA	NA
169	DH-E209	75	86	102	116	30	65	NA	NA	NA
170	DH-E210	80	94	94	120	26	132	NA	NA	NA
171	DH-E211	80	86	83	116	30	125	NA	NA	NA
172	DH-E213	85	94	90	120	26	80	NA	NA	NA
173	DH-E214	85	98	98	123	25	100	NA	NA	NA
174	DH-E215	85	99	100	126	27	140	NA	NA	NA
175	DH-E216	80	98	99	124	26	144	NA	NA	NA

176	DH-E217	85	94	90	120	26	96	NA	NA	NA
CHK1	Chk 1 (WH147)	80	101	100	128	27	212	NA	NA	NA
CHK2	Chk 2 (PBW175)	85	101	113	128	27	187	NA	NA	NA
CHK3	Chk 3 (NI5439)	85	101	107	128	27	253	NA	NA	NA
177	DH-E218	85	99	102	126	27	50	NA	NA	NA
178	DH-E219	95	99	100	126	27	112	NA	NA	NA
179	DH-E220	90	99	99	126	27	125	NA	NA	NA
180	DH-E221	90	99	98	126	27	130	NA	NA	NA
181	DH-E222	85	101	103	128	27	117	NA	NA	NA
182	DH-E223	90	94	90	125	31	125	NA	NA	NA
183	DH-E224	80	94	97	125	31	135	NA	NA	NA
184	DH-E226	85	98	143	125	27	113	NA	NA	NA
185	DH-E227	85	94	103	120	26	180	NA	NA	NA
186	DH-E228	75	97	98	126	29	205	NA	NA	NA
187	DH-E229	80	94	100	120	26	185	NA	NA	NA
188	DH-E230	90	94	101	120	26	145	NA	NA	NA
189	DH-E231	90	86	91	109	23	180	NA	NA	NA
190	DH-E232	85	96	94	125	29	133	NA	NA	NA
191	DH-E233	70	86	92	119	33	175	NA	NA	NA
192	DH-E233	80	101	106	128	27	154	NA	NA	NA
**Location**	Pune									
**Crop-season**	2011-12									
**Condition**	Rainfed									
**S.No.**	**DH-2 Line code**	**GP***	**DTA**	**PH***	**DTM**	**GFD**	**PTPM**	**GWPE**	**TGW***	**GYPP**
CHK1	Chk 1 (WH147)	85	62	68	92	30	97	1.1	30	302
CHK2	Chk 2 (PBW175)	90	62	98	94	32	60	1.4	35	272
CHK3	Chk 3 (NI5439)	75	63	76	97	34	50	1.1	34	223
1	DH-E001	75	58	65	98	40	66	1.4	36	322
2	DH-E003	90	61	74	96	35	69	1.5	30	311
3	DH-E005	90	55	57	96	41	109	1.5	34	272
4	DH-E006	90	70	69	101	31	62	1.4	30	342
5	DH-E007	85	71	72	103	32	77	1.4	31	356
6	DH-E008	85	48	50	82	34	80	1.0	45	236
7	DH-E009	90	62	65	96	34	140	1.2	35	432
8	DH-E010	90	62	74	97	35	93	1.2	32	312
9	DH-E014	90	56	65	87	31	60	1.2	30	227
10	DH-E015	80	50	56	82	32	42	1.4	30	160
11	DH-E018	90	50	56	80	30	87	1.2	36	255
12	DH-E019	90	60	56	88	28	41	0.8	25	196
13	DH-E020	80	53	55	86	33	44	1.3	35	197
14	DH-E021	90	54	60	89	35	71	1.1	32	258
15	DH-E022	90	54	62	86	32	72	1.0	31	227
16	DH-E023	90	68	54	104	36	71	0.9	38	237
CHK1	Chk 1 (WH147)	85	64	69	96	32	91	1.2	29	209
CHK2	Chk 2 (PBW175)	80	64	93	96	32	98	1.4	34	238
CHK3	Chk 3 (NI5439)	80	69	63	99	30	68	1.1	36	203
17	DH-E024	90	50	57	82	32	77	1.0	34	255
18	DH-E025	85	50	56	81	31	81	1.1	36	270
19	DH-E026	90	70	69	99	29	79	0.8	32	261
20	DH-E029	85	70	63	97	27	51	0.9	30	223

21	DH-E032	90	83	60	120	37	82	0.9	30	291
22	DH-E033	65	54	60	86	32	130	1.3	34	220
23	DH-E034	80	54	50	84	30	77	1.7	38	257
24	DH-E036	75	54	50	88	34	48	0.9	26	102
25	DH-E038	70	62	49	91	29	44	1.1	23	80
26	DH-E039	75	58	52	89	31	57	1.0	29	141
27	DH-E040	75	62	44	92	30	48	1.2	23	87
28	DH-E041	80	62	48	89	27	52	0.9	24	99
29	DH-E042	85	64	53	98	34	46	0.8	24	153
30	DH-E043	70	58	57	95	37	42	1.6	31	124
31	DH-E044	85	70	56	99	29	61	0.8	22	155
32	DH-E046	85	61	51	96	35	70	1.0	25	192
CHK1	Chk 1 (WH147)	85	78	61	105	27	79	1.1	30	183
CHK2	Chk 2 (PBW175)	85	60	66	99	39	108	1.1	30	229
CHK3	Chk 3 (NI5439)	85	55	54	86	31	85	1.6	31	233
33	DH-E047	80	62	63	96	34	85	1.7	29	210
34	DH-E051	85	56	65	91	35	78	0.9	39	245
35	DH-E052	85	55	66	93	38	120	1.7	34	294
36	DH-E053	85	64	68	100	36	119	1.2	32	327
37	DH-E054	80	71	63	102	31	56	1.5	32	293
38	DH-E055	70	60	69	101	41	78	1.4	32	216
39	DH-E056	80	64	70	98	34	86	1.4	30	255
40	DH-E058	85	71	52	97	26	94	1.0	24	135
41	DH-E059	90	56	56	86	30	68	1.3	34	298
42	DH-E060	90	56	63	86	30	62	1.1	31	246
43	DH-E061	85	69	57	100	31	78	0.9	36	225
44	DH-E062	90	64	97	98	34	73	1.4	38	226
45	DH-E063	90	68	86	99	31	108	1.1	41	245
46	DH-E065	75	54	52	88	34	75	0.9	34	142
47	DH-E066	90	58	67	91	33	93	1.1	37	220
48	DH-E067	90	60	65	92	32	102	1.0	34	314
CHK1	Chk 1 (WH147)	80	64	82	96	32	97	1.3	32	284
CHK2	Chk 2 (PBW175)	65	64	90	97	33	80	1.3	34	135
CHK3	Chk 3 (NI5439)	85	68	66	97	29	103	1.0	34	217
49	DH-E068	80	50	52	82	32	81	1.1	38	197
50	DH-E069	90	58	69	88	30	92	0.9	38	284
51	DH-E070	85	68	62	96	28	89	0.8	40	214
52	DH-E071	90	52	54	82	30	90	0.9	35	275
53	DH-E072	90	56	66	86	30	101	1.5	33	272
54	DH-E073	85	56	75	93	37	103	1.4	36	274
55	DH-E074	85	61	57	91	30	61	0.9	30	190
56	DH-E075	80	64	60	98	34	56	1.2	30	146
57	DH-E076	85	53	62	82	29	48	1.1	34	123
58	DH-E077	80	51	51	82	31	59	1.2	43	166
59	DH-E078	90	48	55	85	37	94	1.4	40	232
60	DH-E080	80	58	63	99	41	43	1.7	46	142
61	DH-E081	90	57	73	98	41	100	1.5	32	296
62	DH-E082	90	57	70	98	41	85	1.7	37	291
63	DH-E083	90	59	67	103	44	89	1.2	36	272
64	DH-E084	90	70	70	101	31	113	1.2	32	323

CHK1	Chk 1 (WH147)	90	62	80	99	37	98	1.1	33	339
CHK2	Chk 2 (PBW175)	80	64	96	101	37	100	1.6	40	331
CHK3	Chk 3 (NI5439)	90	68	91	101	33	83	1.4	42	415
65	DH-E086	85	55	67	102	47	73	1.4	36	219
66	DH-E087	75	55	70	92	37	87	1.9	41	266
67	DH-E088	80	50	58	98	48	96	1.3	36	223
68	DH-E090	80	53	55	82	29	54	1.3	45	211
69	DH-E091	80	50	55	81	31	68	1.2	44	135
70	DH-E092	70	51	51	82	31	56	1.3	43	83
71	DH-E093	70	51	57	85	34	90	1.1	38	231
72	DH-E094	75	71	81	103	32	121	1.8	32	351
73	DH-E095	90	64	75	98	34	81	2.2	34	373
74	DH-E096	90	57	77	92	35	108	1.8	33	372
75	DH-E097	85	49	66	81	32	85	1.1	44	264
76	DH-E098	90	48	74	80	32	96	1.2	46	252
77	DH-E099	90	54	59	89	35	103	1.1	41	270
78	DH-E100	90	62	59	94	32	122	0.8	25	178
79	DH-E102	85	55	63	84	29	54	1.0	30	203
80	DH-E103	80	55	61	84	29	66	1.2	33	180
CHK1	Chk 1 (WH147)	90	63	86	97	34	115	1.7	32	356
CHK2	Chk 2 (PBW175)	75	64	98	97	33	65	1.3	40	215
CHK3	Chk 3 (NI5439)	90	69	80	98	29	99	1.0	39	286
81	DH-E104	90	55	72	87	32	84	1.1	28	272
82	DH-E105	75	50	66	89	39	71	1.0	34	254
83	DH-E108	85	56	58	84	28	65	1.0	34	265
84	DH-E109	85	55	58	88	33	74	1.2	28	249
85	DH-E110	70	62	54	90	28	56	1.4	30	178
86	DH-E111	90	52	62	82	30	130	1.4	37	306
87	DH-E113	90	69	66	97	28	60	1.2	37	238
88	DH-E114	90	70	55	97	27	99	0.8	29	210
89	DH-E115	90	68	69	97	29	86	1.0	33	280
90	DH-E117	90	58	57	96	38	70	0.8	30	191
91	DH-E118	85	63	67	98	35	101	1.1	32	248
92	DH-E119	90	48	63	80	32	68	1.2	46	270
93	DH-E120	85	70	72	100	30	72	1.0	30	271
94	DH-E121	90	64	65	98	34	90	1.3	29	285
95	DH-E122	80	61	59	92	31	66	0.8	30	194
96	DH-E123	85	64	54	94	30	64	0.8	28	154
CHK1	Chk 1 (WH147)	90	63	92	98	35	127	1.3	32	485
CHK2	Chk 2 (PBW175)	70	64	96	101	37	106	1.5	37	342
CHK3	Chk 3 (NI5439)	90	68	84	100	32	108	1.2	38	379
97	DH-E124	60	53	57	85	32	51	1.6	33	138
98	DH-E125	80	50	57	81	31	77	1.4	36	292
99	DH-E126	90	62	70	98	36	105	1.4	32	304
100	DH-E127	90	70	74	101	31	112	1.1	27	319
101	DH-E129	90	70	72	101	31	78	1.1	26	267
102	DH-E130	85	69	70	101	32	120	0.8	25	338
103	DH-E131	70	54	62	87	33	74	1.4	36	224
104	DH-E132	80	62	60	97	35	98	1.1	27	277
105	DH-E133	80	61	58	97	36	90	1.1	26	221

106	DH-E134	80	71	64	101	30	51	0.8	28	145
107	DH-E135	85	56	68	98	42	76	1.1	30	248
108	DH-E137	90	69	64	98	29	88	1.1	32	277
109	DH-E138	80	54	68	98	44	76	1.3	32	203
110	DH-E141	80	52	54	91	39	48	1.1	32	139
111	DH-E144	80	61	64	98	37	65	1.1	33	222
112	DH-E145	80	70	61	99	29	89	1.2	32	214
CHK1	Chk 1 (WH147)	85	63	92	99	36	93	1.4	35	393
CHK2	Chk 2 (PBW175)	70	64	95	99	35	74	1.6	38	258
CHK3	Chk 3 (NI5439)	85	68	86	98	30	97	1.1	39	298
113	DH-E146	80	58	68	92	34	80	1.1	33	319
114	DH-E147	70	55	73	91	36	90	1.4	40	318
115	DH-E148	85	57	70	98	41	99	1.1	32	355
116	DH-E149	90	64	68	97	33	105	1.2	33	310
117	DH-E150	75	58	69	91	33	97	1.3	32	314
118	DH-E152	80	56	67	91	35	112	1.1	34	310
119	DH-E153	70	62	65	97	35	90	1.3	27	331
120	DH-E154	65	63	77	99	36	110	1.4	28	325
121	DH-E155	90	57	62	84	27	82	1.0	33	240
122	DH-E156	90	57	55	90	33	80	1.0	36	272
123	DH-E158	85	55	58	85	30	52	1.0	43	123
124	DH-E159	90	49	60	79	30	57	1.1	40	190
125	DH-E161	90	49	51	79	30	61	1.1	33	187
126	DH-E162	90	71	65	98	27	97	1.0	30	213
127	DH-E164	90	57	56	85	28	58	0.9	33	187
128	DH-E165	85	54	60	82	28	77	1.1	32	222
CHK1	Chk 1 (WH147)	85	61	64	96	35	96	1.4	29	272
CHK2	Chk 2 (PBW175)	85	64	92	95	31	77	1.1	35	215
CHK3	Chk 3 (NI5439)	90	69	70	96	27	62	1.0	34	182
129	DH-E166	90	59	58	87	28	65	0.9	28	197
130	DH-E167	80	60	59	87	27	60	0.8	29	171
131	DH-E168	90	70	71	98	28	74	1.4	38	245
132	DH-E169	90	61	66	92	31	97	1.1	32	257
133	DH-E170	90	63	81	98	35	101	1.3	36	313
134	DH-E171	75	61	75	95	34	96	1.1	30	239
135	DH-E172	60	50	59	80	30	55	0.9	34	187
136	DH-E175	85	56	59	80	24	95	1.1	33	221
137	DH-E176	85	50	57	79	29	62	1.2	33	186
138	DH-E177	75	71	54	97	26	56	1.0	41	115
139	DH-E178	85	52	53	80	28	64	1.1	37	167
140	DH-E179	90	48	60	79	31	68	0.9	37	183
141	DH-E180	85	49	52	79	30	51	0.8	35	145
142	DH-E181	90	60	62	94	34	90	0.8	32	193
143	DH-E182	90	63	65	95	32	86	0.8	32	204
144	DH-E183	85	48	64	87	39	79	0.9	41	237
CHK1	Chk 1 (WH147)	90	69	83	96	27	107	1.2	38	296
CHK2	Chk 2 (PBW175)	85	63	65	91	28	59	1.5	29	176
CHK3	Chk 3 (NI5439)	90	55	52	82	27	66	1.0	37	161
145	DH-E184	90	50	62	82	32	96	1.4	40	211
146	DH-E185	85	54	70	92	38	74	1.4	36	279

147	DH-E186	85	63	82	99	36	89	1.4	33	292
148	DH-E187	90	55	69	92	37	119	1.2	34	355
149	DH-E188	85	62	68	96	34	101	1.0	28	302
150	DH-E189	75	61	66	95	34	68	1.3	29	196
151	DH-E190	80	58	66	101	43	76	1.0	31	281
152	DH-E191	60	54	53	87	33	67	1.3	37	160
153	DH-E192	70	56	70	89	33	81	1.5	31	324
154	DH-E193	85	52	65	86	34	88	1.4	38	250
155	DH-E194	90	54	63	86	32	114	1.2	38	212
156	DH-E195	90	62	77	98	36	77	1.1	32	246
157	DH-E196	75	64	95	99	35	81	1.8	39	213
158	DH-E197	90	48	55	79	31	91	1.0	40	299
159	DH-E198	85	54	69	80	26	56	1.5	41	267
160	DH-E199	90	53	69	85	32	91	1.2	39	248
CHK1	Chk 1 (WH147)	90	63	84	98	35	99	1.2	34	335
CHK2	Chk 2 (PBW175)	75	63	98	98	35	58	1.3	37	210
CHK3	Chk 3 (NI5439)	80	69	80	98	29	77	1.0	38	224
161	DH-E200	85	64	61	95	31	64	1.2	26	172
162	DH-E202	90	53	70	96	43	97	1.3	35	275
163	DH-E203	90	50	76	86	36	73	1.2	35	285
164	DH-E204	85	64	60	98	34	76	1.1	28	226
165	DH-E205	85	55	71	86	31	80	1.2	35	244
166	DH-E206	70	56	56	88	32	87	1.0	34	218
167	DH-E207	85	56	75	89	33	80	1.2	37	224
168	DH-E208	90	57	65	88	31	77	1.0	33	232
169	DH-E209	85	49	63	87	38	79	1.1	39	266
170	DH-E210	80	56	68	86	30	87	1.1	39	250
171	DH-E211	80	48	65	80	32	83	1.1	37	251
172	DH-E213	80	55	63	79	24	78	1.2	35	242
173	DH-E214	75	75	64	103	28	54	1.0	28	147
174	DH-E215	90	70	65	101	31	69	1.1	30	255
175	DH-E216	85	72	57	99	27	62	0.7	25	132
176	DH-E217	90	55	55	84	29	56	1.0	34	173
CHK1	Chk 1 (WH147)	75	64	84	100	36	62	1.4	31	315
CHK2	Chk 2 (PBW175)	75	64	94	101	37	56	1.6	38	263
CHK3	Chk 3 (NI5439)	85	69	77	98	29	48	0.9	39	188
177	DH-E218	90	63	64	94	31	88	1.1	30	208
178	DH-E219	90	71	64	100	29	92	1.0	29	242
179	DH-E220	75	70	58	97	27	78	1.1	30	226
180	DH-E221	65	80	60	103	23	49	1.1	26	153
181	DH-E222	50	68	75	104	36	52	1.4	32	250
182	DH-E223	70	58	65	94	36	82	1.1	33	332
183	DH-E224	65	57	68	92	35	59	1.4	40	226
184	DH-E226	90	61	98	98	37	85	1.4	26	326
185	DH-E227	70	58	71	92	34	66	1.2	32	247
186	DH-E228	60	71	74	101	30	81	1.4	36	236
187	DH-E229	85	54	72	89	35	102	1.3	35	338
188	DH-E230	70	61	73	97	36	75	1.5	33	224
189	DH-E231	80	49	56	82	33	73	1.2	43	209
190	DH-E232	70	71	83	101	30	86	1.5	37	308

191	DH-E233	85	49	85	92	43	105	1.6	36	391
192	DH-E233	60	64	67	97	33	36	1.6	43	138
**Location**	Pune									
**Crop-season**	2011-12									
**Condition**	Irrigated									
**S.No.**	**DH-2 Line code**	**GP***	**DTA**	**PH***	**DTM**	**GFD**	**PTPM**	**GWPE**	**TGW***	**GYPP**
CHK1	Chk 1 (WH147)	85	67	110	102	35	153	1.8	35	508
CHK2	Chk 2 (PBW175)	85	67	125	107	40	119	2.1	40	531
CHK3	Chk 3 (NI5439)	70	75	100	109	34	94	1.3	40	431
1	DH-E001	30	61	79	101	40	62	1.9	37	200
2	DH-E003	70	68	85	102	34	83	1.5	32	362
3	DH-E005	70	57	73	100	43	110	1.2	34	317
4	DH-E006	65	59	80	106	47	77	1.4	34	269
5	DH-E007	70	60	76	107	47	94	1.4	39	479
6	DH-E008	65	52	71	93	41	72	1.2	44	249
7	DH-E009	75	69	88	107	38	84	1.4	38	378
8	DH-E010	85	63	88	100	37	88	1.5	37	446
9	DH-E014	60	60	81	103	43	81	1.9	35	384
10	DH-E015	75	54	82	99	45	87	1.4	35	473
11	DH-E018	90	49	77	94	45	77	1.6	43	425
12	DH-E019	80	63	77	102	39	104	1.6	31	484
13	DH-E020	30	57	82	102	45	75	1.7	40	349
14	DH-E021	90	64	82	108	44	113	1.2	35	581
15	DH-E022	85	56	83	97	41	115	1.4	34	436
16	DH-E023	90	92	85	114	22	97	0.8	34	428
CHK1	Chk 1 (WH147)	90	69	112	110	41	123	1.8	34	738
CHK2	Chk 2 (PBW175)	85	69	120	109	40	86	2.1	40	642
CHK3	Chk 3 (NI5439)	90	73	94	109	36	97	1.3	40	449
17	DH-E024	85	52	77	88	36	81	1.1	42	328
18	DH-E025	80	51	73	91	40	115	1.4	38	533
19	DH-E026	90	77	89	113	36	138	0.8	34	489
20	DH-E029	75	79	87	113	34	116	1.3	33	574
21	DH-E032	85	63	77	108	45	127	1.2	30	580
22	DH-E033	70	55	78	94	39	77	1.5	38	473
23	DH-E034	80	52	80	94	42	112	1.9	39	622
24	DH-E036	75	61	85	102	41	71	1.6	28	491
25	DH-E038	60	69	67	109	40	93	1.7	30	428
26	DH-E039	75	61	81	100	39	92	1.5	35	521
27	DH-E040	80	70	75	102	32	88	1.8	30	435
28	DH-E041	85	63	77	99	36	98	1.7	32	518
29	DH-E042	90	69	80	100	31	81	1.2	30	457
30	DH-E043	90	60	82	100	40	96	1.9	36	513
31	DH-E044	90	78	83	112	34	86	0.8	31	489
32	DH-E046	80	60	77	102	42	82	1.5	31	353
CHK1	Chk 1 (WH147)	90	92	91	119	27	84	1.4	28	411
CHK2	Chk 2 (PBW175)	90	70	91	115	45	123	1.9	31	609
CHK3	Chk 3 (NI5439)	85	63	83	97	34	94	1.5	32	692
33	DH-E047	85	63	88	101	38	81	1.1	31	401
34	DH-E051	90	61	77	101	40	78	1.6	38	566
35	DH-E052	90	59	77	98	39	122	1.7	31	610

36	DH-E053	90	69	80	113	44	86	1.3	32	635
37	DH-E054	80	74	81	114	40	76	1.7	36	493
38	DH-E055	70	67	87	112	45	122	1.7	28	602
39	DH-E056	75	69	90	113	44	97	1.6	29	687
40	DH-E058	80	94	87	117	23	110	1.3	23	510
41	DH-E059	90	57	84	97	40	104	1.6	34	678
42	DH-E060	70	60	86	99	39	84	1.5	32	637
43	DH-E061	90	67	112	100	33	113	1.4	37	742
44	DH-E062	75	69	123	101	32	88	1.8	40	468
45	DH-E063	90	71	104	102	31	124	1.2	40	610
46	DH-E065	70	58	75	96	38	89	1.5	32	458
47	DH-E066	85	65	91	98	33	108	1.5	34	613
48	DH-E067	90	63	82	98	35	93	1.5	36	711
CHK1	Chk 1 (WH147)	90	66	107	103	37	98	1.3	44	728
CHK2	Chk 2 (PBW175)	30	73	122	108	35	70	2.6	42	490
CHK3	Chk 3 (NI5439)	75	72	95	108	36	89	1.5	42	522
49	DH-E068	90	78	78	118	40	104	1.5	38	531
50	DH-E069	85	60	89	97	37	103	1.3	34	586
51	DH-E070	70	76	78	111	35	103	1.0	28	506
52	DH-E071	70	52	80	97	45	85	1.4	40	482
53	DH-E072	65	56	83	97	41	95	1.2	31	521
54	DH-E073	75	59	85	98	39	91	1.5	37	581
55	DH-E074	65	66	80	103	37	96	1.6	34	469
56	DH-E075	90	74	82	113	39	105	1.6	39	510
57	DH-E076	90	57	85	95	38	85	1.4	31	582
58	DH-E077	80	52	75	91	39	92	1.6	38	536
59	DH-E078	90	51	80	94	43	112	0.7	38	516
60	DH-E080	75	66	80	101	35	53	2.1	52	387
61	DH-E081	85	61	84	114	53	129	1.3	28	618
62	DH-E082	85	60	92	113	53	75	1.4	35	607
63	DH-E083	90	61	82	112	51	97	1.2	34	535
64	DH-E084	80	79	78	110	31	83	1.2	21	516
CHK1	Chk 1 (WH147)	80	69	114	107	38	40	1.3	37	294
CHK2	Chk 2 (PBW175)	75	68	117	111	43	65	2.2	40	339
CHK3	Chk 3 (NI5439)	65	75	98	112	37	56	1.5	46	333
65	DH-E086	85	60	80	97	37	69	2.1	35	496
66	DH-E087	90	55	77	97	42	112	1.8	37	563
67	DH-E088	85	53	81	97	44	91	1.6	40	422
68	DH-E090	65	57	75	91	34	42	1.2	36	273
69	DH-E091	75	52	72	89	37	68	1.5	43	320
70	DH-E092	65	53	65	90	37	58	1.0	40	199
71	DH-E093	60	57	75	95	38	43	1.2	33	186
72	DH-E094	65	79	100	115	36	65	1.3	31	351
73	DH-E095	80	70	97	100	30	96	2.1	36	648
74	DH-E096	55	65	82	114	49	57	1.3	35	309
75	DH-E097	65	55	86	95	40	71	1.5	42	402
76	DH-E098	70	52	92	94	42	58	1.4	42	427
77	DH-E099	80	56	80	95	39	60	0.7	36	413
78	DH-E100	90	70	81	102	32	73	1.3	30	411
79	DH-E102	80	61	86	96	35	65	1.5	34	470

80	DH-E103	75	61	86	97	36	61	1.5	38	452
CHK1	Chk 1 (WH147)	90	67	110	108	41	109	1.7	34	757
CHK2	Chk 2 (PBW175)	65	72	124	110	38	61	1.6	40	514
CHK3	Chk 3 (NI5439)	90	71	108	111	40	105	1.3	40	626
81	DH-E104	90	63	85	102	39	80	1.4	34	571
82	DH-E105	90	57	90	102	45	79	1.4	40	541
83	DH-E108	85	60	89	97	37	73	1.6	40	583
84	DH-E109	90	57	81	101	44	79	1.2	32	477
85	DH-E110	60	69	86	108	39	85	1.9	34	465
86	DH-E111	85	53	81	97	44	56	0.7	34	564
87	DH-E113	85	74	87	111	37	79	1.5	38	597
88	DH-E114	90	77	79	114	37	91	0.9	30	521
89	DH-E115	90	73	92	110	37	84	1.1	36	606
90	DH-E117	85	63	78	110	47	89	0.9	32	469
91	DH-E118	90	67	91	113	46	79	1.4	32	633
92	DH-E119	90	49	87	93	44	88	1.4	43	539
93	DH-E120	85	92	86	115	23	92	1.5	32	517
94	DH-E121	85	75	85	113	38	92	1.3	31	637
95	DH-E122	90	67	85	101	34	96	1.4	37	684
96	DH-E123	85	76	87	113	37	62	1.4	35	533
CHK1	Chk 1 (WH147)	90	65	111	110	45	122	1.9	37	642
CHK2	Chk 2 (PBW175)	80	66	116	110	44	108	1.4	41	544
CHK3	Chk 3 (NI5439)	70	75	109	109	34	84	1.4	40	458
97	DH-E124	75	54	81	96	42	59	1.5	34	502
98	DH-E125	75	51	82	97	46	65	1.3	36	541
99	DH-E126	90	65	83	114	49	59	1.0	35	458
100	DH-E127	90	79	80	113	34	105	1.4	34	512
101	DH-E129	90	80	84	115	35	78	1.3	31	561
102	DH-E130	90	93	82	117	24	72	1.4	27	473
103	DH-E131	90	53	85	95	42	105	1.0	32	515
104	DH-E132	90	68	78	110	42	145	1.2	23	490
105	DH-E133	80	67	79	110	43	115	1.4	26	432
106	DH-E134	90	77	87	113	36	88	0.8	31	456
107	DH-E135	90	57	82	100	43	107	1.2	34	401
108	DH-E137	90	73	78	101	28	98	1.4	33	573
109	DH-E138	80	61	88	100	39	81	1.3	28	535
110	DH-E141	90	52	91	98	46	69	1.4	36	441
111	DH-E144	90	66	88	108	42	117	1.6	32	598
112	DH-E145	85	76	83	110	34	99	1.5	30	551
CHK1	Chk 1 (WH147)	90	66	116	102	36	129	1.5	36	692
CHK2	Chk 2 (PBW175)	80	68	111	110	42	121	1.9	42	672
CHK3	Chk 3 (NI5439)	90	69	105	103	34	98	1.7	42	642
113	DH-E146	85	63	91	100	37	84	2.0	33	560
114	DH-E147	85	61	90	98	37	142	1.7	38	578
115	DH-E148	85	63	89	102	39	109	1.7	36	539
116	DH-E149	90	69	77	112	43	97	1.1	38	493
117	DH-E150	90	63	85	97	34	101	2.1	31	542
118	DH-E152	90	57	85	97	40	108	1.3	38	640
119	DH-E153	80	67	82	101	34	71	1.3	27	586
120	DH-E154	75	68	92	113	45	120	1.5	32	547

121	DH-E155	85	57	84	97	40	65	1.2	35	517
122	DH-E156	85	66	79	102	36	78	1.3	32	496
123	DH-E158	85	61	86	97	36	79	1.6	30	523
124	DH-E159	85	52	74	91	39	78	1.3	45	535
125	DH-E161	75	53	82	97	44	93	1.3	39	531
126	DH-E162	90	77	93	110	33	75	1.8	37	605
127	DH-E164	90	64	75	99	35	68	1.0	27	351
128	DH-E165	85	57	78	97	40	78	1.3	30	426
CHK1	Chk 1 (WH147)	90	66	109	102	36	127	2.0	36	726
CHK2	Chk 2 (PBW175)	85	67	125	108	41	95	1.7	42	473
CHK3	Chk 3 (NI5439)	90	68	114	108	40	92	1.3	41	540
129	DH-E166	90	63	82	101	38	87	1.6	30	434
130	DH-E167	85	67	75	100	33	98	1.7	29	529
131	DH-E168	90	77	83	111	34	105	1.7	33	563
132	DH-E169	75	67	80	102	35	88	1.4	31	477
133	DH-E170	85	72	110	103	31	94	1.8	37	651
134	DH-E171	90	65	90	102	37	84	1.4	32	451
135	DH-E172	70	56	80	99	43	73	1.6	36	431
136	DH-E175	90	56	84	95	39	82	1.1	34	567
137	DH-E176	90	60	81	94	34	94	1.3	36	527
138	DH-E177	75	79	85	110	31	87	1.5	29	407
139	DH-E178	70	63	80	97	34	84	1.3	36	317
140	DH-E179	90	57	82	95	38	92	1.1	38	517
141	DH-E180	90	52	76	94	42	88	1.6	34	581
142	DH-E181	90	69	80	100	31	96	1.2	35	562
143	DH-E182	90	60	81	98	38	105	1.5	41	630
144	DH-E183	90	55	77	97	42	86	1.3	37	507
CHK1	Chk 1 (WH147)	75	73	109	110	37	75	1.5	41	579
CHK2	Chk 2 (PBW175)	80	74	84	110	36	68	1.6	34	581
CHK3	Chk 3 (NI5439)	85	61	84	96	35	79	1.2	36	628
145	DH-E184	90	57	79	95	38	95	1.3	34	475
146	DH-E185	85	58	94	97	39	97	2.0	35	634
147	DH-E186	85	66	88	110	44	95	2.0	33	459
148	DH-E187	90	61	83	98	37	97	1.2	32	418
149	DH-E188	90	66	80	100	34	80	1.6	33	320
150	DH-E189	80	66	78	101	35	56	1.2	35	288
151	DH-E190	85	65	91	110	45	71	1.7	31	526
152	DH-E191	80	65	75	108	43	95	1.4	32	510
153	DH-E192	90	61	83	97	36	112	1.8	32	610
154	DH-E193	80	54	82	97	43	95	1.4	34	471
155	DH-E194	90	60	83	97	37	88	1.4	38	640
156	DH-E195	85	66	119	102	36	142	1.8	35	699
157	DH-E196	50	69	125	108	39	92	1.4	40	502
158	DH-E197	80	53	77	90	37	89	1.5	36	451
159	DH-E198	90	54	84	94	40	95	0.9	36	573
160	DH-E199	85	61	84	99	38	94	1.6	32	513
CHK1	Chk 1 (WH147)	90	65	109	100	35	115	1.6	36	791
CHK2	Chk 2 (PBW175)	85	67	128	110	43	89	1.5	42	567
CHK3	Chk 3 (NI5439)	90	68	106	110	42	110	1.4	42	637
161	DH-E200	80	61	81	99	38	102	1.6	36	477

162	DH-E202	85	61	86	110	49	107	1.7	35	607
163	DH-E203	90	54	81	97	43	84	1.5	35	572
164	DH-E204	80	74	83	110	36	73	2.2	27	492
165	DH-E205	90	58	90	98	40	79	2.0	34	599
166	DH-E206	90	55	75	98	43	98	1.3	38	623
167	DH-E207	90	57	83	98	41	97	2.1	37	629
168	DH-E208	90	60	89	98	38	82	1.1	31	512
169	DH-E209	90	51	86	98	47	78	1.3	36	626
170	DH-E210	90	60	83	98	38	101	1.4	34	608
171	DH-E211	90	51	84	89	38	96	1.2	37	547
172	DH-E213	90	57	87	95	38	122	1.4	34	608
173	DH-E214	85	92	95	118	26	97	1.3	22	390
174	DH-E215	90	79	85	112	33	94	1.4	28	537
175	DH-E216	90	78	88	111	33	78	1.3	30	472
176	DH-E217	90	56	83	95	39	92	1.3	35	512
CHK1	Chk 1 (WH147)	90	66	114	108	42	129	1.9	34	687
CHK2	Chk 2 (PBW175)	85	66	124	109	43	115	1.8	42	620
CHK3	Chk 3 (NI5439)	80	69	108	110	41	86	1.3	42	426
177	DH-E218	85	66	90	110	44	99	1.2	34	614
178	DH-E219	90	78	97	113	35	96	1.3	27	592
179	DH-E220	90	74	97	110	36	128	1.4	33	799
180	DH-E221	90	92	93	113	21	82	1.3	25	452
181	DH-E222	90	67	95	110	43	87	1.2	36	583
182	DH-E223	85	55	82	98	43	116	1.2	32	574
183	DH-E224	90	52	81	93	41	90	1.5	40	555
184	DH-E226	90	69	135	100	31	88	1.1	29	586
185	DH-E227	85	56	95	98	42	81	1.2	32	501
186	DH-E228	85	73	106	108	35	94	1.7	39	598
187	DH-E229	90	60	88	98	38	112	1.1	33	651
188	DH-E230	90	64	93	100	36	96	1.3	35	631
189	DH-E231	85	49	80	90	41	84	1.2	35	457
190	DH-E232	80	75	101	114	39	107	1.6	40	670
191	DH-E233	90	52	99	100	48	127	1.8	38	836
192	DH-E233	80	56	93	98	42	98	1.5	36	402
**Location**	Hisar									
**Crop-season**	2011-12									
**Condition**	Rainfed									
**S.No.**	**DH-2 Line code**	**GP***	**DTA**	**PH**	**DTM**	**GFD**	**PTPM**	**GWPE**	**TGW***	**GYPP**
CHK1	Chk 1 (WH147)	55	NA	NA	118	NA	NA	1.1	19	149
CHK2	Chk 2 (PBW175)	65	NA	NA	123	NA	NA	1.2	26	149
CHK3	Chk 3 (NI5439)	63	NA	NA	122	NA	NA	1.6	27	149
1	DH-E001	80	NA	NA	125	NA	NA	1.6	28	136
2	DH-E003	75	NA	NA	124	NA	NA	1.1	39	149
3	DH-E005	64	NA	NA	122	NA	NA	1.2	13	149
4	DH-E006	70	NA	NA	123	NA	NA	1.4	16	149
5	DH-E007	75	NA	NA	124	NA	NA	1.7	31	149
6	DH-E008	71	NA	NA	124	NA	NA	1.4	31	149
7	DH-E009	60	NA	NA	121	NA	NA	2.0	29	149
8	DH-E010	60	NA	NA	121	NA	NA	2.0	35	149
9	DH-E014	87	NA	NA	126	NA	NA	1.2	31	149

10	DH-E015	65	NA	NA	123	NA	NA	1.3	20	149
11	DH-E018	89	NA	NA	126	NA	NA	1.5	32	149
12	DH-E019	62	NA	NA	121	NA	NA	1.1	21	149
13	DH-E020	92	NA	NA	126	NA	NA	1.2	33	149
14	DH-E021	74	NA	NA	124	NA	NA	1.3	21	149
15	DH-E022	76	NA	NA	124	NA	NA	1.4	32	149
16	DH-E023	60	NA	NA	119	NA	NA	2.1	28	149
CHK1	Chk 1 (WH147)	60	NA	NA	120	NA	NA	1.0	22	170
CHK2	Chk 2 (PBW175)	85	NA	NA	125	NA	NA	1.6	35	170
CHK3	Chk 3 (NI5439)	87	NA	NA	126	NA	NA	1.1	33	170
17	DH-E024	70	NA	NA	124	NA	NA	0.9	29	153
18	DH-E025	55	NA	NA	119	NA	NA	1.9	31	153
19	DH-E026	65	NA	NA	123	NA	NA	1.1	25	153
20	DH-E029	65	NA	NA	122	NA	NA	1.3	25	157
21	DH-E032	55	NA	NA	117	NA	NA	1.0	16	162
22	DH-E033	60	NA	NA	121	NA	NA	1.5	25	162
23	DH-E034	87	NA	NA	126	NA	NA	1.4	26	163
24	DH-E036	70	NA	NA	124	NA	NA	1.3	24	166
25	DH-E038	60	NA	NA	121	NA	NA	1.7	23	170
26	DH-E039	65	NA	NA	123	NA	NA	1.7	23	170
27	DH-E040	70	NA	NA	124	NA	NA	1.3	16	170
28	DH-E041	55	NA	NA	116	NA	NA	1.0	19	161
29	DH-E042	63	NA	NA	122	NA	NA	1.2	19	170
30	DH-E043	65	NA	NA	122	NA	NA	1.4	32	170
31	DH-E044	78	NA	NA	125	NA	NA	1.6	24	170
32	DH-E046	63	NA	NA	122	NA	NA	1.3	30	170
CHK1	Chk 1 (WH147)	77	NA	NA	124	NA	NA	1.4	26	179
CHK2	Chk 2 (PBW175)	65	NA	NA	123	NA	NA	1.4	25	179
CHK3	Chk 3 (NI5439)	80	NA	NA	125	NA	NA	1.1	24	179
33	DH-E047	65	NA	NA	123	NA	NA	1.3	28	170
34	DH-E051	93	NA	NA	126	NA	NA	1.3	31	170
35	DH-E052	65	NA	NA	122	NA	NA	1.3	16	177
36	DH-E053	55	NA	NA	119	NA	NA	1.8	27	174
37	DH-E054	68	NA	NA	123	NA	NA	1.5	18	179
38	DH-E055	75	NA	NA	124	NA	NA	1.7	22	179
39	DH-E056	63	NA	NA	121	NA	NA	1.3	17	179
40	DH-E058	94	NA	NA	126	NA	NA	1.7	29	183
41	DH-E059	65	NA	NA	122	NA	NA	1.6	28	183
42	DH-E060	86	NA	NA	125	NA	NA	1.2	29	183
43	DH-E061	60	NA	NA	120	NA	NA	1.5	27	187
44	DH-E062	93	NA	NA	126	NA	NA	1.7	28	187
45	DH-E063	80	NA	NA	125	NA	NA	1.3	31	187
46	DH-E065	55	NA	NA	119	NA	NA	1.1	27	187
47	DH-E066	92	NA	NA	126	NA	NA	1.0	19	191
48	DH-E067	75	NA	NA	124	NA	NA	1.6	33	191
CHK1	Chk 1 (WH147)	55	NA	NA	120	NA	NA	1.3	15	174
CHK2	Chk 2 (PBW175)	54	NA	NA	116	NA	NA	1.3	21	152
CHK3	Chk 3 (NI5439)	65	NA	NA	123	NA	NA	1.6	26	191
49	DH-E068	60	NA	NA	119	NA	NA	1.1	22	191
50	DH-E069	76	NA	NA	124	NA	NA	1.2	29	191

51	DH-E070	65	NA	NA	123	NA	NA	1.0	27	191
52	DH-E071	63	NA	NA	121	NA	NA	1.1	18	191
53	DH-E072	77	NA	NA	124	NA	NA	1.0	24	191
54	DH-E073	54	NA	NA	116	NA	NA	0.8	24	126
55	DH-E074	67	NA	NA	123	NA	NA	0.8	23	191
56	DH-E075	79	NA	NA	125	NA	NA	1.3	23	191
57	DH-E076	60	NA	NA	121	NA	NA	1.7	26	191
58	DH-E077	72	NA	NA	124	NA	NA	1.0	22	191
59	DH-E078	65	NA	NA	123	NA	NA	1.5	35	191
60	DH-E080	75	NA	NA	124	NA	NA	1.5	34	191
61	DH-E081	67	NA	NA	123	NA	NA	1.2	20	191
62	DH-E082	65	NA	NA	123	NA	NA	1.5	24	191
63	DH-E083	74	NA	NA	124	NA	NA	1.2	22	191
64	DH-E084	70	NA	NA	124	NA	NA	1.2	23	191
CHK1	Chk 1 (WH147)	72	NA	NA	124	NA	NA	1.3	25	191
CHK2	Chk 2 (PBW175)	85	NA	NA	125	NA	NA	1.6	25	191
CHK3	Chk 3 (NI5439)	55	NA	NA	119	NA	NA	2.0	29	174
65	DH-E086	65	NA	NA	123	NA	NA	1.4	27	191
66	DH-E087	55	NA	NA	120	NA	NA	1.8	28	191
67	DH-E088	86	NA	NA	125	NA	NA	1.2	29	191
68	DH-E090	85	NA	NA	125	NA	NA	1.7	31	191
69	DH-E091	55	NA	NA	120	NA	NA	1.3	20	174
70	DH-E092	65	NA	NA	123	NA	NA	1.0	16	191
71	DH-E093	63	NA	NA	122	NA	NA	1.3	26	191
72	DH-E094	65	NA	NA	123	NA	NA	1.3	29	191
73	DH-E095	82	NA	NA	125	NA	NA	1.4	33	191
74	DH-E096	95	NA	NA	127	NA	NA	1.4	24	191
75	DH-E097	60	NA	NA	121	NA	NA	1.4	29	191
76	DH-E098	55	NA	NA	116	NA	NA	1.0	20	152
77	DH-E099	72	NA	NA	124	NA	NA	1.3	29	195
78	DH-E100	65	NA	NA	123	NA	NA	1.5	18	200
79	DH-E102	55	NA	NA	119	NA	NA	1.2	20	174
80	DH-E103	70	NA	NA	124	NA	NA	1.6	25	204
CHK1	Chk 1 (WH147)	55	NA	NA	118	NA	NA	1.9	31	213
CHK2	Chk 2 (PBW175)	69	NA	NA	124	NA	NA	1.6	20	213
CHK3	Chk 3 (NI5439)	60	NA	NA	121	NA	NA	1.3	26	213
81	DH-E104	94	NA	NA	126	NA	NA	1.6	32	204
82	DH-E105	56	NA	NA	120	NA	NA	1.6	31	187
83	DH-E108	90	NA	NA	126	NA	NA	1.0	28	204
84	DH-E109	55	NA	NA	118	NA	NA	1.2	21	204
85	DH-E110	65	NA	NA	122	NA	NA	1.6	20	208
86	DH-E111	85	NA	NA	125	NA	NA	1.8	27	208
87	DH-E113	84	NA	NA	125	NA	NA	1.4	30	213
88	DH-E114	73	NA	NA	124	NA	NA	1.4	25	213
89	DH-E115	62	NA	NA	122	NA	NA	1.4	25	213
90	DH-E117	65	NA	NA	123	NA	NA	1.5	28	213
91	DH-E118	75	NA	NA	124	NA	NA	2.0	31	213
92	DH-E119	75	NA	NA	124	NA	NA	1.7	25	217
93	DH-E120	55	NA	NA	120	NA	NA	1.4	17	174
94	DH-E121	65	NA	NA	122	NA	NA	1.3	20	217

95	DH-E122	65	NA	NA	123	NA	NA	0.9	15	221
96	DH-E123	67	NA	NA	122	NA	NA	1.4	23	221
CHK1	Chk 1 (WH147)	60	NA	NA	121	NA	NA	1.2	20	195
CHK2	Chk 2 (PBW175)	85	NA	NA	125	NA	NA	1.6	25	230
CHK3	Chk 3 (NI5439)	65	NA	NA	123	NA	NA	1.6	25	230
97	DH-E124	72	NA	NA	124	NA	NA	2.1	29	221
98	DH-E125	75	NA	NA	124	NA	NA	1.8	29	223
99	DH-E126	96	NA	NA	127	NA	NA	1.6	22	223
100	DH-E127	75	NA	NA	124	NA	NA	1.6	26	223
101	DH-E129	65	NA	NA	123	NA	NA	1.7	27	230
102	DH-E130	65	NA	NA	122	NA	NA	1.3	22	230
103	DH-E131	98	NA	NA	127	NA	NA	1.8	31	230
104	DH-E132	60	NA	NA	121	NA	NA	2.0	31	206
105	DH-E133	60	NA	NA	121	NA	NA	1.0	15	195
106	DH-E134	65	NA	NA	122	NA	NA	1.5	24	231
107	DH-E135	55	NA	NA	120	NA	NA	1.6	24	174
108	DH-E137	63	NA	NA	122	NA	NA	1.9	24	234
109	DH-E138	65	NA	NA	123	NA	NA	1.4	22	234
110	DH-E141	63	NA	NA	122	NA	NA	1.2	19	224
111	DH-E144	75	NA	NA	124	NA	NA	1.8	26	234
112	DH-E145	80	NA	NA	125	NA	NA	1.1	30	234
CHK1	Chk 1 (WH147)	67	NA	NA	120	NA	NA	1.6	21	187
CHK2	Chk 2 (PBW175)	74	NA	NA	124	NA	NA	1.4	36	234
CHK3	Chk 3 (NI5439)	65	NA	NA	123	NA	NA	1.1	34	234
113	DH-E146	90	NA	NA	126	NA	NA	1.5	33	234
114	DH-E147	87	NA	NA	125	NA	NA	1.4	30	234
115	DH-E148	96	NA	NA	127	NA	NA	1.5	24	234
116	DH-E149	65	NA	NA	121	NA	NA	1.7	29	217
117	DH-E150	75	NA	NA	124	NA	NA	1.5	21	234
118	DH-E152	85	NA	NA	125	NA	NA	1.2	22	234
119	DH-E153	85	NA	NA	125	NA	NA	1.4	28	234
120	DH-E154	70	NA	NA	124	NA	NA	1.4	36	234
121	DH-E155	70	NA	NA	124	NA	NA	1.2	21	234
122	DH-E156	55	NA	NA	119	NA	NA	2.4	36	174
123	DH-E158	58	NA	NA	120	NA	NA	1.7	26	187
124	DH-E159	75	NA	NA	124	NA	NA	1.5	25	234
125	DH-E161	67	NA	NA	121	NA	NA	1.1	24	217
126	DH-E162	97	NA	NA	127	NA	NA	1.7	28	238
127	DH-E164	65	NA	NA	123	NA	NA	1.3	33	238
128	DH-E165	80	NA	NA	125	NA	NA	1.4	22	238
CHK1	Chk 1 (WH147)	75	NA	NA	124	NA	NA	1.8	23	247
CHK2	Chk 2 (PBW175)	78	NA	NA	125	NA	NA	1.3	32	247
CHK3	Chk 3 (NI5439)	64	NA	NA	122	NA	NA	1.3	21	217
129	DH-E166	75	NA	NA	124	NA	NA	1.3	21	238
130	DH-E167	63	NA	NA	122	NA	NA	1.7	22	236
131	DH-E168	70	NA	NA	124	NA	NA	1.4	29	239
132	DH-E169	63	NA	NA	122	NA	NA	1.8	26	228
133	DH-E170	65	NA	NA	123	NA	NA	1.2	28	240
134	DH-E171	60	NA	NA	121	NA	NA	1.5	18	195
135	DH-E172	65	NA	NA	123	NA	NA	0.9	20	239

136	DH-E175	76	NA	NA	124	NA	NA	1.1	34	247
137	DH-E176	63	NA	NA	122	NA	NA	1.5	26	236
138	DH-E177	77	NA	NA	124	NA	NA	1.4	20	252
139	DH-E178	60	NA	NA	119	NA	NA	1.9	31	174
140	DH-E179	65	NA	NA	123	NA	NA	1.6	27	254
141	DH-E180	80	NA	NA	125	NA	NA	0.8	22	255
142	DH-E181	65	NA	NA	123	NA	NA	1.2	40	255
143	DH-E182	55	NA	NA	116	NA	NA	1.9	30	156
144	DH-E183	65	NA	NA	123	NA	NA	1.1	22	240
CHK1	Chk 1 (WH147)	67	NA	NA	120	NA	NA	1.1	11	183
CHK2	Chk 2 (PBW175)	80	NA	NA	125	NA	NA	1.0	35	264
CHK3	Chk 3 (NI5439)	70	NA	NA	124	NA	NA	1.0	18	261
145	DH-E184	68	NA	NA	123	NA	NA	2.0	20	255
146	DH-E185	67	NA	NA	123	NA	NA	1.5	23	234
147	DH-E186	80	NA	NA	125	NA	NA	1.5	29	256
148	DH-E187	92	NA	NA	126	NA	NA	1.6	29	256
149	DH-E188	67	NA	NA	123	NA	NA	1.3	21	239
150	DH-E189	78	NA	NA	125	NA	NA	1.3	19	256
151	DH-E190	89	NA	NA	126	NA	NA	1.3	19	259
152	DH-E191	65	NA	NA	122	NA	NA	1.2	31	239
153	DH-E192	60	NA	NA	121	NA	NA	1.1	16	217
154	DH-E193	78	NA	NA	124	NA	NA	1.2	16	259
155	DH-E194	85	NA	NA	125	NA	NA	1.7	29	260
156	DH-E195	55	NA	NA	117	NA	NA	1.1	28	174
157	DH-E196	60	NA	NA	121	NA	NA	1.8	25	214
158	DH-E197	55	NA	NA	116	NA	NA	1.9	28	152
159	DH-E198	65	NA	NA	123	NA	NA	1.2	16	240
160	DH-E199	65	NA	NA	123	NA	NA	1.5	25	240
CHK1	Chk 1 (WH147)	55	NA	NA	119	NA	NA	2.0	31	174
CHK2	Chk 2 (PBW175)	60	NA	NA	122	NA	NA	1.1	12	217
CHK3	Chk 3 (NI5439)	65	NA	NA	123	NA	NA	1.0	30	256
161	DH-E200	87	NA	NA	125	NA	NA	1.4	30	264
162	DH-E202	62	NA	NA	122	NA	NA	1.4	21	217
163	DH-E203	85	NA	NA	125	NA	NA	1.5	32	264
164	DH-E204	84	NA	NA	125	NA	NA	1.7	31	264
165	DH-E205	65	NA	NA	123	NA	NA	1.2	25	254
166	DH-E206	70	NA	NA	124	NA	NA	1.3	26	261
167	DH-E207	65	NA	NA	123	NA	NA	1.3	24	256
168	DH-E208	60	NA	NA	121	NA	NA	1.5	28	195
169	DH-E209	75	NA	NA	124	NA	NA	2.2	27	272
170	DH-E210	60	NA	NA	121	NA	NA	1.3	25	195
171	DH-E211	55	NA	NA	118	NA	NA	1.9	22	174
172	DH-E213	75	NA	NA	124	NA	NA	1.4	27	272
173	DH-E214	68	NA	NA	123	NA	NA	1.2	21	260
174	DH-E215	77	NA	NA	124	NA	NA	1.2	20	273
175	DH-E216	70	NA	NA	124	NA	NA	1.3	23	261
176	DH-E217	60	NA	NA	121	NA	NA	1.1	18	217
CHK1	Chk 1 (WH147)	55	NA	NA	119	NA	NA	1.5	24	174
CHK2	Chk 2 (PBW175)	55	NA	NA	119	NA	NA	1.1	19	174
CHK3	Chk 3 (NI5439)	60	NA	NA	121	NA	NA	0.8	17	195

177	DH-E218	55	NA	NA	119	NA	NA	1.9	31	174
178	DH-E219	65	NA	NA	123	NA	NA	1.4	17	239
179	DH-E220	55	NA	NA	117	NA	NA	1.5	28	169
180	DH-E221	60	NA	NA	121	NA	NA	1.5	20	217
181	DH-E222	70	NA	NA	124	NA	NA	1.7	18	261
182	DH-E223	59	NA	NA	120	NA	NA	1.4	20	191
183	DH-E224	84	NA	NA	125	NA	NA	1.5	25	276
184	DH-E226	60	NA	NA	121	NA	NA	1.6	24	191
185	DH-E227	78	NA	NA	124	NA	NA	1.5	22	276
186	DH-E228	80	NA	NA	125	NA	NA	1.3	33	276
187	DH-E229	55	NA	NA	116	NA	NA	1.6	31	161
188	DH-E230	60	NA	NA	122	NA	NA	1.5	23	217
189	DH-E231	78	NA	NA	124	NA	NA	1.2	27	281
190	DH-E232	68	NA	NA	124	NA	NA	1.7	25	260
191	DH-E233	75	NA	NA	124	NA	NA	1.1	18	274
192	DH-E233	64	NA	NA	122	NA	NA	1.3	19	236
**Location**	Hisar									
**Crop-season**	2011-12									
**Condition**	Irrigated									
**S.No.**	**DH-2 Line code**	**GP***	**DTA**	**PH**	**DTM**	**GFD**	**PTPM**	**GWPE**	**TGW***	**GYPP**
CHK1	Chk 1 (WH147)	65	NA	NA	123	NA	NA	2.0	33	204
CHK2	Chk 2 (PBW175)	75	NA	NA	127	NA	NA	1.4	34	282
CHK3	Chk 3 (NI5439)	75	NA	NA	126	NA	NA	1.9	32	276
1	DH-E001	85	NA	NA	130	NA	NA	1.7	37	343
2	DH-E003	83	NA	NA	130	NA	NA	1.6	45	328
3	DH-E005	75	NA	NA	126	NA	NA	1.2	24	255
4	DH-E006	80	NA	NA	129	NA	NA	1.6	20	302
5	DH-E007	80	NA	NA	129	NA	NA	2.4	42	328
6	DH-E008	80	NA	NA	129	NA	NA	2.0	35	307
7	DH-E009	70	NA	NA	125	NA	NA	2.8	45	230
8	DH-E010	72	NA	NA	126	NA	NA	2.3	40	241
9	DH-E014	90	NA	NA	131	NA	NA	1.5	37	357
10	DH-E015	77	NA	NA	128	NA	NA	1.7	37	299
11	DH-E018	90	NA	NA	132	NA	NA	1.7	38	357
12	DH-E019	73	NA	NA	126	NA	NA	1.7	25	252
13	DH-E020	91	NA	NA	133	NA	NA	1.5	40	373
14	DH-E021	80	NA	NA	129	NA	NA	2.8	43	307
15	DH-E022	85	NA	NA	130	NA	NA	1.6	44	333
16	DH-E023	68	NA	NA	125	NA	NA	2.3	46	204
CHK1	Chk 1 (WH147)	70	NA	NA	125	NA	NA	1.9	32	204
CHK2	Chk 2 (PBW175)	90	NA	NA	130	NA	NA	1.7	41	350
CHK3	Chk 3 (NI5439)	90	NA	NA	131	NA	NA	1.6	43	357
17	DH-E024	80	NA	NA	129	NA	NA	1.6	39	307
18	DH-E025	65	NA	NA	124	NA	NA	2.9	43	204
19	DH-E026	76	NA	NA	128	NA	NA	1.4	43	297
20	DH-E029	75	NA	NA	127	NA	NA	1.9	31	281
21	DH-E032	65	NA	NA	123	NA	NA	1.9	28	204
22	DH-E033	70	NA	NA	125	NA	NA	1.8	32	230
23	DH-E034	90	NA	NA	131	NA	NA	1.7	37	357
24	DH-E036	80	NA	NA	129	NA	NA	1.6	28	307

25	DH-E038	72	NA	NA	125	NA	NA	2.7	30	230
26	DH-E039	76	NA	NA	128	NA	NA	1.9	35	282
27	DH-E040	80	NA	NA	129	NA	NA	1.3	23	306
28	DH-E041	65	NA	NA	123	NA	NA	2.1	34	200
29	DH-E042	75	NA	NA	126	NA	NA	1.3	25	271
30	DH-E043	75	NA	NA	127	NA	NA	1.7	36	281
31	DH-E044	85	NA	NA	130	NA	NA	1.7	42	339
32	DH-E046	75	NA	NA	126	NA	NA	1.8	35	278
CHK1	Chk 1 (WH147)	85	NA	NA	130	NA	NA	1.8	33	333
CHK2	Chk 2 (PBW175)	77	NA	NA	128	NA	NA	1.8	45	297
CHK3	Chk 3 (NI5439)	87	NA	NA	130	NA	NA	1.4	38	349
33	DH-E047	75	NA	NA	127	NA	NA	1.7	42	281
34	DH-E051	92	NA	NA	135	NA	NA	2.1	39	374
35	DH-E052	75	NA	NA	126	NA	NA	1.5	23	255
36	DH-E053	65	NA	NA	124	NA	NA	2.0	35	210
37	DH-E054	80	NA	NA	129	NA	NA	1.6	25	306
38	DH-E055	84	NA	NA	130	NA	NA	1.9	37	328
39	DH-E056	75	NA	NA	126	NA	NA	1.4	18	255
40	DH-E058	93	NA	NA	135	NA	NA	1.9	40	376
41	DH-E059	75	NA	NA	127	NA	NA	1.8	42	281
42	DH-E060	90	NA	NA	131	NA	NA	2.2	32	355
43	DH-E061	70	NA	NA	125	NA	NA	2.5	37	220
44	DH-E062	92	NA	NA	134	NA	NA	2.0	33	374
45	DH-E063	86	NA	NA	130	NA	NA	1.7	42	348
46	DH-E065	65	NA	NA	124	NA	NA	1.4	47	204
47	DH-E066	91	NA	NA	132	NA	NA	1.5	22	371
48	DH-E067	80	NA	NA	129	NA	NA	2.3	45	323
CHK1	Chk 1 (WH147)	70	NA	NA	125	NA	NA	1.9	27	225
CHK2	Chk 2 (PBW175)	60	NA	NA	122	NA	NA	2.2	34	225
CHK3	Chk 3 (NI5439)	75	NA	NA	127	NA	NA	2.2	34	282
49	DH-E068	68	NA	NA	124	NA	NA	2.1	31	204
50	DH-E069	85	NA	NA	130	NA	NA	1.4	41	333
51	DH-E070	76	NA	NA	128	NA	NA	1.6	34	296
52	DH-E071	73	NA	NA	126	NA	NA	2.4	32	252
53	DH-E072	85	NA	NA	130	NA	NA	1.7	42	333
54	DH-E073	60	NA	NA	122	NA	NA	1.6	27	225
55	DH-E074	78	NA	NA	129	NA	NA	1.1	28	301
56	DH-E075	85	NA	NA	130	NA	NA	1.6	39	343
57	DH-E076	72	NA	NA	126	NA	NA	2.6	34	230
58	DH-E077	80	NA	NA	129	NA	NA	2.6	38	307
59	DH-E078	76	NA	NA	128	NA	NA	1.7	44	282
60	DH-E080	80	NA	NA	129	NA	NA	1.8	39	323
61	DH-E081	79	NA	NA	129	NA	NA	1.6	26	302
62	DH-E082	75	NA	NA	128	NA	NA	1.6	30	282
63	DH-E083	80	NA	NA	129	NA	NA	2.6	44	313
64	DH-E084	80	NA	NA	129	NA	NA	1.7	31	307
CHK1	Chk 1 (WH147)	80	NA	NA	129	NA	NA	1.5	42	307
CHK2	Chk 2 (PBW175)	90	NA	NA	130	NA	NA	1.9	33	355
CHK3	Chk 3 (NI5439)	65	NA	NA	123	NA	NA	2.7	33	225
65	DH-E086	75	NA	NA	128	NA	NA	1.7	31	282

66	DH-E087	70	NA	NA	125	NA	NA	2.1	33	220
67	DH-E088	90	NA	NA	131	NA	NA	1.5	35	355
68	DH-E090	90	NA	NA	130	NA	NA	2.0	43	350
69	DH-E091	70	NA	NA	125	NA	NA	1.5	23	225
70	DH-E092	75	NA	NA	128	NA	NA	1.5	18	282
71	DH-E093	75	NA	NA	126	NA	NA	1.8	30	271
72	DH-E094	75	NA	NA	128	NA	NA	1.5	43	282
73	DH-E095	87	NA	NA	130	NA	NA	1.9	38	350
74	DH-E096	95	NA	NA	135	NA	NA	1.7	37	378
75	DH-E097	70	NA	NA	125	NA	NA	2.0	34	230
76	DH-E098	65	NA	NA	122	NA	NA	1.3	29	225
77	DH-E099	80	NA	NA	129	NA	NA	1.9	32	307
78	DH-E100	75	NA	NA	128	NA	NA	1.5	31	282
79	DH-E102	65	NA	NA	124	NA	NA	1.8	28	239
80	DH-E103	80	NA	NA	129	NA	NA	1.9	35	307
CHK1	Chk 1 (WH147)	65	NA	NA	123	NA	NA	2.0	38	250
CHK2	Chk 2 (PBW175)	80	NA	NA	129	NA	NA	1.6	32	306
CHK3	Chk 3 (NI5439)	73	NA	NA	126	NA	NA	2.0	35	250
81	DH-E104	95	NA	NA	135	NA	NA	1.7	36	376
82	DH-E105	70	NA	NA	125	NA	NA	2.1	37	240
83	DH-E108	90	NA	NA	132	NA	NA	1.5	44	357
84	DH-E109	65	NA	NA	123	NA	NA	1.2	29	240
85	DH-E110	75	NA	NA	127	NA	NA	1.7	30	281
86	DH-E111	89	NA	NA	130	NA	NA	1.9	39	350
87	DH-E113	87	NA	NA	130	NA	NA	1.5	31	350
88	DH-E114	80	NA	NA	129	NA	NA	1.6	40	307
89	DH-E115	75	NA	NA	126	NA	NA	1.6	28	255
90	DH-E117	77	NA	NA	129	NA	NA	1.5	44	301
91	DH-E118	80	NA	NA	129	NA	NA	2.8	46	328
92	DH-E119	80	NA	NA	129	NA	NA	1.8	34	328
93	DH-E120	70	NA	NA	125	NA	NA	2.3	37	260
94	DH-E121	75	NA	NA	126	NA	NA	1.6	23	260
95	DH-E122	75	NA	NA	127	NA	NA	1.9	37	281
96	DH-E123	75	NA	NA	126	NA	NA	1.6	27	260
CHK1	Chk 1 (WH147)	71	NA	NA	125	NA	NA	2.5	33	270
CHK2	Chk 2 (PBW175)	90	NA	NA	130	NA	NA	1.8	34	355
CHK3	Chk 3 (NI5439)	77	NA	NA	128	NA	NA	1.7	35	299
97	DH-E124	80	NA	NA	129	NA	NA	2.2	46	307
98	DH-E125	81	NA	NA	129	NA	NA	2.2	41	328
99	DH-E126	95	NA	NA	135	NA	NA	1.6	39	378
100	DH-E127	84	NA	NA	130	NA	NA	2.0	33	333
101	DH-E129	75	NA	NA	127	NA	NA	1.8	30	281
102	DH-E130	75	NA	NA	127	NA	NA	1.5	29	281
103	DH-E131	98	NA	NA	135	NA	NA	2.1	41	383
104	DH-E132	72	NA	NA	126	NA	NA	2.2	41	270
105	DH-E133	70	NA	NA	125	NA	NA	1.9	21	272
106	DH-E134	75	NA	NA	127	NA	NA	1.9	32	281
107	DH-E135	70	NA	NA	125	NA	NA	2.4	41	275
108	DH-E137	75	NA	NA	127	NA	NA	2.0	42	278
109	DH-E138	75	NA	NA	128	NA	NA	1.7	36	282

110	DH-E141	75	NA	NA	126	NA	NA	1.9	23	275
111	DH-E144	81	NA	NA	130	NA	NA	1.9	40	328
112	DH-E145	85	NA	NA	130	NA	NA	1.7	35	348
CHK1	Chk 1 (WH147)	70	NA	NA	125	NA	NA	1.7	33	275
CHK2	Chk 2 (PBW175)	80	NA	NA	129	NA	NA	2.6	41	323
CHK3	Chk 3 (NI5439)	77	NA	NA	128	NA	NA	1.5	40	299
113	DH-E146	90	NA	NA	132	NA	NA	1.7	45	357
114	DH-E147	90	NA	NA	131	NA	NA	1.6	40	355
115	DH-E148	95	NA	NA	135	NA	NA	1.7	35	378
116	DH-E149	75	NA	NA	126	NA	NA	1.9	34	275
117	DH-E150	85	NA	NA	130	NA	NA	1.6	39	333
118	DH-E152	90	NA	NA	130	NA	NA	1.6	31	355
119	DH-E153	90	NA	NA	130	NA	NA	1.8	38	350
120	DH-E154	80	NA	NA	129	NA	NA	1.8	41	307
121	DH-E155	80	NA	NA	129	NA	NA	1.4	27	307
122	DH-E156	65	NA	NA	124	NA	NA	2.5	37	275
123	DH-E158	70	NA	NA	125	NA	NA	2.4	34	275
124	DH-E159	80	NA	NA	129	NA	NA	2.4	38	328
125	DH-E161	75	NA	NA	126	NA	NA	2.4	38	275
126	DH-E162	97	NA	NA	135	NA	NA	2.1	32	382
127	DH-E164	76	NA	NA	128	NA	NA	1.7	45	282
128	DH-E165	86	NA	NA	130	NA	NA	1.4	39	348
CHK1	Chk 1 (WH147)	80	NA	NA	129	NA	NA	2.1	40	328
CHK2	Chk 2 (PBW175)	85	NA	NA	130	NA	NA	1.4	36	333
CHK3	Chk 3 (NI5439)	75	NA	NA	126	NA	NA	1.4	37	295
129	DH-E166	82	NA	NA	130	NA	NA	1.7	27	328
130	DH-E167	75	NA	NA	126	NA	NA	1.8	35	280
131	DH-E168	80	NA	NA	129	NA	NA	2.3	42	307
132	DH-E169	75	NA	NA	126	NA	NA	2.0	38	282
133	DH-E170	76	NA	NA	128	NA	NA	1.7	35	283
134	DH-E171	70	NA	NA	125	NA	NA	1.8	29	283
135	DH-E172	75	NA	NA	127	NA	NA	1.3	24	290
136	DH-E175	85	NA	NA	130	NA	NA	1.3	41	333
137	DH-E176	75	NA	NA	126	NA	NA	1.8	33	290
138	DH-E177	85	NA	NA	130	NA	NA	1.4	38	333
139	DH-E178	70	NA	NA	125	NA	NA	2.5	41	300
140	DH-E179	77	NA	NA	128	NA	NA	1.9	39	300
141	DH-E180	85	NA	NA	130	NA	NA	1.5	25	347
142	DH-E181	78	NA	NA	129	NA	NA	1.6	49	301
143	DH-E182	65	NA	NA	122	NA	NA	2.2	34	300
144	DH-E183	75	NA	NA	128	NA	NA	1.8	39	300
CHK1	Chk 1 (WH147)	70	NA	NA	125	NA	NA	2.4	28	310
CHK2	Chk 2 (PBW175)	85	NA	NA	130	NA	NA	1.8	43	348
CHK3	Chk 3 (NI5439)	80	NA	NA	129	NA	NA	1.5	29	310
145	DH-E184	80	NA	NA	129	NA	NA	2.1	29	306
146	DH-E185	78	NA	NA	129	NA	NA	1.8	37	301
147	DH-E186	85	NA	NA	130	NA	NA	2.0	47	343
148	DH-E187	91	NA	NA	134	NA	NA	1.6	42	374
149	DH-E188	80	NA	NA	129	NA	NA	1.5	23	302
150	DH-E189	85	NA	NA	130	NA	NA	1.4	32	333

151	DH-E190	90	NA	NA	132	NA	NA	1.4	27	357
152	DH-E191	75	NA	NA	127	NA	NA	1.8	47	305
153	DH-E192	74	NA	NA	126	NA	NA	1.6	22	305
154	DH-E193	85	NA	NA	130	NA	NA	1.5	32	333
155	DH-E194	89	NA	NA	130	NA	NA	1.7	35	350
156	DH-E195	65	NA	NA	123	NA	NA	1.9	34	310
157	DH-E196	73	NA	NA	126	NA	NA	2.5	34	310
158	DH-E197	65	NA	NA	122	NA	NA	2.4	40	310
159	DH-E198	75	NA	NA	128	NA	NA	1.9	29	310
160	DH-E199	75	NA	NA	127	NA	NA	1.5	36	310
CHK1	Chk 1 (WH147)	70	NA	NA	125	NA	NA	2.2	44	325
CHK2	Chk 2 (PBW175)	75	NA	NA	126	NA	NA	1.1	18	325
CHK3	Chk 3 (NI5439)	78	NA	NA	129	NA	NA	1.4	38	325
161	DH-E200	90	NA	NA	131	NA	NA	1.7	41	355
162	DH-E202	75	NA	NA	126	NA	NA	2.0	28	310
163	DH-E203	90	NA	NA	130	NA	NA	1.9	39	350
164	DH-E204	88	NA	NA	130	NA	NA	2.0	37	350
165	DH-E205	77	NA	NA	128	NA	NA	1.4	46	311
166	DH-E206	80	NA	NA	129	NA	NA	1.8	40	314
167	DH-E207	78	NA	NA	129	NA	NA	1.5	38	320
168	DH-E208	72	NA	NA	126	NA	NA	2.1	39	320
169	DH-E209	80	NA	NA	129	NA	NA	2.5	50	323
170	DH-E210	70	NA	NA	125	NA	NA	2.0	33	320
171	DH-E211	65	NA	NA	123	NA	NA	2.1	30	320
172	DH-E213	84	NA	NA	130	NA	NA	1.7	37	332
173	DH-E214	80	NA	NA	129	NA	NA	1.4	28	321
174	DH-E215	85	NA	NA	130	NA	NA	1.5	33	333
175	DH-E216	80	NA	NA	129	NA	NA	2.2	40	325
176	DH-E217	73	NA	NA	126	NA	NA	1.9	26	325
CHK1	Chk 1 (WH147)	65	NA	NA	124	NA	NA	1.9	32	340
CHK2	Chk 2 (PBW175)	68	NA	NA	124	NA	NA	1.4	28	340
CHK3	Chk 3 (NI5439)	70	NA	NA	125	NA	NA	1.2	23	351
177	DH-E218	65	NA	NA	123	NA	NA	2.1	35	325
178	DH-E219	75	NA	NA	127	NA	NA	1.6	30	325
179	DH-E220	65	NA	NA	123	NA	NA	2.6	45	325
180	DH-E221	75	NA	NA	126	NA	NA	2.1	26	325
181	DH-E222	80	NA	NA	129	NA	NA	1.9	27	325
182	DH-E223	70	NA	NA	125	NA	NA	1.7	26	325
183	DH-E224	87	NA	NA	130	NA	NA	1.6	39	350
184	DH-E226	70	NA	NA	125	NA	NA	2.2	28	325
185	DH-E227	85	NA	NA	130	NA	NA	1.6	31	333
186	DH-E228	86	NA	NA	130	NA	NA	1.8	38	349
187	DH-E229	65	NA	NA	122	NA	NA	2.0	41	325
188	DH-E230	75	NA	NA	126	NA	NA	1.6	40	325
189	DH-E231	85	NA	NA	130	NA	NA	1.7	34	333
190	DH-E232	80	NA	NA	129	NA	NA	1.8	30	330
191	DH-E233	80	NA	NA	129	NA	NA	2.5	39	330
192	DH-E233	75	NA	NA	127	NA	NA	1.5	22	335
**Location**	Kanpur									
**Crop-season**	2012-13									

Condition	Rainfed									
**S.No.**	**DH-2 Line code**	**GP***	**DTA**	**PH***	**DTM**	**GFD**	**PTPM**	**GWPE**	**TGW***	**GYPP**
CHK1	Chk 1 (WH147)	77	78	87	127	49	378	NA	38	355
CHK2	Chk 2 (PBW175)	82	80	95	118	38	345	NA	33	321
CHK3	Chk 3 (NI5439)	77	80	100	128	48	387	NA	38	364
1	DH-E001	80	76	63	123	47	360	NA	33	335
2	DH-E003	80	75	83	129	54	330	NA	36	310
3	DH-E005	75	80	58	125	45	345	NA	36	322
4	DH-E006	80	79	66	121	42	330	NA	37	306
5	DH-E007	81	74	56	122	48	328	NA	33	305
6	DH-E008	75	73	48	120	47	336	NA	34	313
7	DH-E009	77	76	68	123	47	321	NA	31	300
8	DH-E010	79	75	62	119	44	350	NA	34	326
9	DH-E014	74	80	52	129	49	346	NA	34	323
10	DH-E015	71	79	58	123	44	337	NA	32	314
11	DH-E018	80	74	67	123	49	340	NA	32	318
12	DH-E019	81	79	75	123	44	350	NA	34	327
13	DH-E020	80	78	68	122	44	345	NA	33	322
14	DH-E021	82	76	75	120	44	330	NA	31	308
15	DH-E022	79	81	61	125	44	360	NA	34	343
16	DH-E023	77	80	73	121	41	355	NA	33	336
CHK1	Chk 1 (WH147)	75	81	84	124	43	375	NA	35	353
CHK2	Chk 2 (PBW175)	76	80	87	122	42	380	NA	39	360
CHK3	Chk 3 (NI5439)	80	81	83	123	42	390	NA	41	367
17	DH-E024	80	78	45	121	43	365	NA	32	334
18	DH-E025	81	75	44	119	44	336	NA	31	316
19	DH-E026	80	81	71	125	44	340	NA	33	317
20	DH-E029	79	73	62	117	44	328	NA	36	305
21	DH-E032	81	80	56	120	40	342	NA	33	319
22	DH-E033	80	73	60	121	48	370	NA	36	348
23	DH-E034	76	78	59	118	40	330	NA	32	307
24	DH-E036	79	80	55	125	45	342	NA	33	314
25	DH-E038	82	79	58	120	41	345	NA	36	317
26	DH-E039	70	76	61	122	46	368	NA	34	340
27	DH-E040	67	70	65	118	48	335	NA	33	318
28	DH-E041	68	75	67	121	46	326	NA	35	300
29	DH-E042	70	71	73	120	49	364	NA	35	335
30	DH-E043	67	76	66	118	42	345	NA	32	321
31	DH-E044	73	75	70	124	49	312	NA	33	285
32	DH-E046	73	76	65	124	48	350	NA	32	323
CHK1	Chk 1 (WH147)	69	77	70	128	51	385	NA	39	358
CHK2	Chk 2 (PBW175)	70	74	60	120	46	376	NA	40	345
CHK3	Chk 3 (NI5439)	65	75	68	122	47	360	NA	40	350
33	DH-E047	65	78	64	120	42	354	NA	31	327
34	DH-E051	80	76	58	115	39	347	NA	33	320
35	DH-E052	68	75	62	120	45	306	NA	36	279
36	DH-E053	67	76	73	118	42	314	NA	33	287
37	DH-E054	73	77	67	117	40	333	NA	32	306
38	DH-E055	67	74	80	120	46	326	NA	31	284
39	DH-E056	67	79	79	125	46	341	NA	34	314

40	DH-E058	73	76	45	119	43	362	NA	35	335
41	DH-E059	69	74	50	118	44	360	NA	37	350
42	DH-E060	68	77	49	119	42	360	NA	36	328
43	DH-E061	70	76	69	120	44	323	NA	36	292
44	DH-E062	67	77	90	123	46	310	NA	33	280
45	DH-E063	71	76	87	119	43	322	NA	32	295
46	DH-E065	73	78	49	122	44	338	NA	35	315
47	DH-E066	73	78	63	120	42	357	NA	35	330
48	DH-E067	68	80	74	121	41	336	NA	35	310
CHK1	Chk 1 (WH147)	71	78	58	122	44	338	NA	38	311
CHK2	Chk 2 (PBW175)	73	79	77	118	39	298	NA	38	275
CHK3	Chk 3 (NI5439)	72	74	84	121	47	310	NA	39	285
49	DH-E068	67	73	57	118	45	312	NA	36	285
50	DH-E069	73	71	65	121	50	306	NA	33	282
51	DH-E070	67	76	60	121	45	337	NA	32	310
52	DH-E071	69	76	58	118	42	330	NA	31	303
53	DH-E072	68	77	54	118	41	346	NA	31	319
54	DH-E073	73	78	60	120	42	355	NA	32	328
55	DH-E074	73	74	57	123	49	361	NA	33	335
56	DH-E075	70	75	72	120	45	325	NA	33	300
57	DH-E076	75	79	52	120	41	380	NA	35	350
58	DH-E077	72	75	59	123	48	370	NA	33	345
59	DH-E078	69	75	59	122	47	365	NA	32	340
60	DH-E080	72	73	63	117	44	368	NA	36	346
61	DH-E081	72	75	84	119	44	390	NA	37	368
62	DH-E082	75	75	68	121	46	381	NA	35	355
63	DH-E083	68	75	72	119	44	375	NA	38	350
64	DH-E084	71	76	71	123	47	361	NA	38	340
CHK1	Chk 1 (WH147)	75	77	65	117	40	390	NA	39	370
CHK2	Chk 2 (PBW175)	70	92	80	136	44	378	NA	38	356
CHK3	Chk 3 (NI5439)	74	93	76	137	44	351	NA	37	330
65	DH-E086	70	72	63	120	48	395	NA	39	373
66	DH-E087	72	73	55	117	44	291	NA	37	269
67	DH-E088	73	72	58	110	38	278	NA	37	256
68	DH-E090	71	90	60	131	41	395	NA	35	375
69	DH-E091	70	76	57	120	44	310	NA	34	288
70	DH-E092	70	74	42	118	44	318	NA	33	296
71	DH-E093	72	76	59	120	44	311	NA	39	290
72	DH-E094	69	73	77	117	44	348	NA	38	325
73	DH-E095	71	75	56	119	44	297	NA	38	275
74	DH-E096	70	74	88	118	44	318	NA	39	296
75	DH-E097	68	76	64	120	44	310	NA	38	285
76	DH-E098	75	76	72	120	44	315	NA	37	298
77	DH-E099	71	74	58	118	44	321	NA	36	300
78	DH-E100	70	73	64	117	44	315	NA	36	295
79	DH-E102	70	75	66	119	44	297	NA	36	285
80	DH-E103	71	74	57	118	44	310	NA	39	290
CHK1	Chk 1 (WH147)	67	76	56	120	44	291	NA	38	257
CHK2	Chk 2 (PBW175)	72	89	67	130	41	295	NA	37	275
CHK3	Chk 3 (NI5439)	70	90	72	134	44	310	NA	36	295

81	DH-E104	70	74	61	118	44	278	NA	36	256
82	DH-E105	70	71	60	115	44	298	NA	38	276
83	DH-E108	68	70	54	114	44	310	NA	38	290
84	DH-E109	70	76	60	115	39	280	NA	36	260
85	DH-E110	71	74	52	118	44	300	NA	35	278
86	DH-E111	73	75	62	119	44	305	NA	39	280
87	DH-E113	72	84	81	128	44	318	NA	38	300
88	DH-E114	70	79	75	123	44	323	NA	37	305
89	DH-E115	68	77	80	120	43	383	NA	33	345
90	DH-E117	72	80	81	128	48	390	NA	38	372
91	DH-E118	74	71	78	114	43	365	NA	35	336
92	DH-E119	69	78	65	121	43	379	NA	35	355
93	DH-E120	74	78	60	127	49	376	NA	35	350
94	DH-E121	71	71	75	114	43	372	NA	36	346
95	DH-E122	70	71	65	114	43	377	NA	30	347
96	DH-E123	71	73	63	116	43	389	NA	38	370
CHK1	Chk 1 (WH147)	73	73	67	116	43	381	NA	37	360
CHK2	Chk 2 (PBW175)	72	75	75	118	43	310	NA	36	290
CHK3	Chk 3 (NI5439)	73	75	62	118	43	391	NA	38	370
97	DH-E124	70	80	68	128	48	376	NA	32	352
98	DH-E125	75	77	52	120	43	365	NA	31	345
99	DH-E126	70	73	68	116	43	367	NA	33	323
100	DH-E127	68	75	74	118	43	370	NA	34	352
101	DH-E129	72	76	79	119	43	376	NA	36	350
102	DH-E130	70	78	68	121	43	384	NA	37	360
103	DH-E131	74	78	52	128	50	372	NA	36	354
104	DH-E132	74	63	60	106	43	393	NA	37	378
105	DH-E133	75	76	58	119	43	396	NA	37	378
106	DH-E134	74	73	64	116	43	387	NA	33	355
107	DH-E135	71	78	56	128	50	391	NA	32	363
108	DH-E137	70	71	67	114	43	391	NA	35	373
109	DH-E138	71	77	66	120	43	325	NA	32	308
110	DH-E141	74	80	59	128	48	402	NA	39	384
111	DH-E144	70	81	61	124	43	348	NA	33	330
112	DH-E145	66	71	62	114	43	395	NA	36	375
CHK1	Chk 1 (WH147)	72	70	60	122	52	395	NA	35	370
CHK2	Chk 2 (PBW175)	74	76	59	119	43	358	NA	30	340
CHK3	Chk 3 (NI5439)	73	75	70	118	43	362	NA	32	345
113	DH-E146	67	78	60	127	49	387	NA	32	365
114	DH-E147	68	75	60	118	43	355	NA	33	337
115	DH-E148	70	78	70	121	43	396	NA	37	378
116	DH-E149	71	81	62	129	48	387	NA	34	369
117	DH-E150	72	79	58	128	49	376	NA	32	360
118	DH-E152	72	70	55	113	43	384	NA	31	365
119	DH-E153	70	73	62	116	43	386	NA	33	355
120	DH-E154	71	64	63	109	45	379	NA	37	361
121	DH-E155	72	68	57	111	43	375	NA	33	358
122	DH-E156	73	75	55	118	43	385	NA	35	368
123	DH-E158	70	76	56	119	43	380	NA	35	350
124	DH-E159	68	77	50	120	43	374	NA	36	345

125	DH-E161	71	80	48	128	48	406	NA	38	370
126	DH-E162	72	78	73	121	43	390	NA	35	361
127	DH-E164	71	78	49	121	43	388	NA	36	355
128	DH-E165	70	66	56	109	43	330	NA	31	303
CHK1	Chk 1 (WH147)	74	74	60	117	43	368	NA	36	341
CHK2	Chk 2 (PBW175)	68	73	60	110	37	388	NA	37	355
CHK3	Chk 3 (NI5439)	70	79	71	122	43	395	NA	38	370
129	DH-E166	72	71	63	114	43	355	NA	36	328
130	DH-E167	75	78	64	126	48	390	NA	37	360
131	DH-E168	70	75	73	120	45	375	NA	35	345
132	DH-E169	68	74	62	117	43	370	NA	36	335
133	DH-E170	72	80	56	129	49	384	NA	36	350
134	DH-E171	71	76	62	119	43	378	NA	37	351
135	DH-E172	70	78	55	125	47	390	NA	37	363
136	DH-E175	70	72	57	115	43	360	NA	36	330
137	DH-E176	72	71	50	114	43	353	NA	37	325
138	DH-E177	70	73	66	116	43	363	NA	37	336
139	DH-E178	72	75	45	120	45	375	NA	35	348
140	DH-E179	71	75	42	118	43	373	NA	36	350
141	DH-E180	72	78	51	121	43	390	NA	36	362
142	DH-E181	73	80	61	127	47	366	NA	37	340
143	DH-E182	74	76	65	124	48	376	NA	37	347
144	DH-E183	72	72	54	115	43	358	NA	37	330
CHK1	Chk 1 (WH147)	73	78	73	126	48	355	NA	35	330
CHK2	Chk 2 (PBW175)	70	80	67	127	47	400	NA	38	375
CHK3	Chk 3 (NI5439)	75	76	50	119	43	384	NA	36	355
145	DH-E184	73	80	56	128	48	400	NA	38	375
146	DH-E185	75	70	58	113	43	348	NA	37	320
147	DH-E186	70	70	56	118	48	350	NA	38	325
148	DH-E187	70	78	60	121	43	398	NA	38	371
149	DH-E188	65	74	61	117	43	378	NA	35	351
150	DH-E189	73	76	66	120	44	355	NA	34	325
151	DH-E190	72	75	68	118	43	373	NA	35	346
152	DH-E191	70	75	55	118	43	387	NA	37	360
153	DH-E192	72	74	60	120	46	345	NA	35	321
154	DH-E193	70	76	53	119	43	366	NA	36	340
155	DH-E194	75	75	58	118	43	381	NA	36	354
156	DH-E195	72	80	75	128	48	358	NA	32	328
157	DH-E196	75	78	66	125	47	356	NA	34	335
158	DH-E197	74	76	54	120	44	370	NA	40	340
159	DH-E198	74	78	54	125	47	378	NA	38	350
160	DH-E199	75	75	43	120	45	376	NA	42	352
CHK1	Chk 1 (WH147)	71	77	60	125	48	327	NA	29	295
CHK2	Chk 2 (PBW175)	73	72	75	116	44	380	NA	35	356
CHK3	Chk 3 (NI5439)	74	80	80	128	48	380	NA	49	350
161	DH-E200	76	74	47	118	44	383	NA	35	360
162	DH-E202	70	73	43	117	44	376	NA	38	350
163	DH-E203	74	76	42	120	44	328	NA	30	304
164	DH-E204	75	73	41	117	44	345	NA	45	321
165	DH-E205	72	75	46	120	45	377	NA	38	350

166	DH-E206	72	72	47	115	43	362	NA	30	330
167	DH-E207	68	65	74	109	44	330	NA	37	290
168	DH-E208	72	70	61	121	51	378	NA	37	350
169	DH-E209	75	67	54	113	46	365	NA	38	340
170	DH-E210	71	71	51	115	44	355	NA	35	330
171	DH-E211	76	73	61	118	45	370	NA	30	345
172	DH-E213	74	68	57	113	45	345	NA	38	321
173	DH-E214	75	73	69	117	44	378	NA	35	354
174	DH-E215	74	74	64	122	48	340	NA	30	310
175	DH-E216	70	77	62	121	44	352	NA	35	325
176	DH-E217	72	77	54	121	44	394	NA	48	375
CHK1	Chk 1 (WH147)	75	78	92	136	58	389	NA	45	365
CHK2	Chk 2 (PBW175)	73	75	90	125	50	350	NA	37	325
CHK3	Chk 3 (NI5439)	74	76	92	120	44	385	NA	46	360
177	DH-E218	70	76	60	120	44	374	NA	45	345
178	DH-E219	71	71	56	115	44	380	NA	43	350
179	DH-E220	74	68	52	114	46	374	NA	48	342
180	DH-E221	72	62	56	106	44	343	NA	35	315
181	DH-E222	75	73	64	117	44	387	NA	45	360
182	DH-E223	73	67	45	110	43	372	NA	49	340
183	DH-E224	74	80	47	128	48	396	NA	44	375
184	DH-E226	72	78	50	124	46	390	NA	39	360
185	DH-E227	71	77	45	121	44	400	NA	40	370
186	DH-E228	73	76	47	120	44	384	NA	56	360
187	DH-E229	74	80	53	127	47	385	NA	48	361
188	DH-E230	75	71	49	115	44	406	NA	50	380
189	DH-E231	71	75	56	119	44	386	NA	46	362
190	DH-E232	72	80	71	127	47	394	NA	38	370
191	DH-E233	73	75	75	124	49	348	NA	30	325
192	DH-E233	70	77	100	121	44	375	NA	39	350
**Location**	Kanpur									
**Crop-season**	2012-13									
**Condition**	Irrigated									
**S.No.**	**DH-2 Line code**	**GP***	**DTA**	**PH***	**DTM**	**GFD**	**PTPM**	**GWPE**	**TGW***	**GYPP**
CHK1	Chk 1 (WH147)	76	86	91	131	45	447	NA	42	355
CHK2	Chk 2 (PBW175)	80	89	99	138	49	421	NA	38	321
CHK3	Chk 3 (NI5439)	80	89	105	128	39	419	NA	42	364
1	DH-E001	75	83	71	135	52	410	NA	38	335
2	DH-E003	79	84	96	127	43	377	NA	38	310
3	DH-E005	73	73	65	136	63	383	NA	37	322
4	DH-E006	79	80	68	138	58	377	NA	38	306
5	DH-E007	75	85	66	134	49	375	NA	39	305
6	DH-E008	75	77	66	137	60	377	NA	40	313
7	DH-E009	78	82	88	139	57	395	NA	35	300
8	DH-E010	74	85	72	135	50	391	NA	40	326
9	DH-E014	78	81	66	139	58	391	NA	36	323
10	DH-E015	80	88	70	143	55	379	NA	35	314
11	DH-E018	74	89	75	130	41	398	NA	38	318
12	DH-E019	79	95	85	136	41	395	NA	37	327
13	DH-E020	82	89	75	130	41	391	NA	38	322

14	DH-E021	78	91	85	137	46	379	NA	39	308
15	DH-E022	74	87	73	139	52	406	NA	40	343
16	DH-E023	80	84	75	139	55	403	NA	38	336
CHK1	Chk 1 (WH147)	76	93	94	137	44	417	NA	38	353
CHK2	Chk 2 (PBW175)	80	92	95	135	43	447	NA	43	360
CHK3	Chk 3 (NI5439)	77	92	98	129	37	444	NA	44	367
17	DH-E024	75	89	60	133	44	400	NA	36	334
18	DH-E025	80	90	68	128	38	389	NA	35	316
19	DH-E026	78	90	93	132	42	379	NA	38	317
20	DH-E029	75	83	75	124	41	387	NA	41	305
21	DH-E032	76	79	72	133	54	383	NA	37	319
22	DH-E033	79	73	79	130	57	437	NA	42	348
23	DH-E034	78	88	82	128	40	378	NA	41	307
24	DH-E036	73	83	80	134	51	376	NA	37	314
25	DH-E038	80	91	66	127	36	407	NA	38	317
26	DH-E039	77	82	84	130	48	454	NA	38	419
27	DH-E040	74	75	75	124	49	536	NA	41	500
28	DH-E041	78	84	92	129	45	411	NA	37	374
29	DH-E042	76	79	75	125	46	474	NA	38	450
30	DH-E043	78	82	80	130	48	439	NA	38	413
31	DH-E044	76	81	82	129	48	449	NA	38	425
32	DH-E046	80	81	74	130	49	449	NA	38	415
CHK1	Chk 1 (WH147)	80	80	80	131	51	483	NA	41	461
CHK2	Chk 2 (PBW175)	79	76	76	128	52	468	NA	39	440
CHK3	Chk 3 (NI5439)	73	78	74	129	51	462	NA	37	444
33	DH-E047	78	80	82	132	52	444	NA	40	414
34	DH-E051	77	78	89	130	52	424	NA	41	381
35	DH-E052	80	81	83	129	48	480	NA	40	447
36	DH-E053	79	80	83	130	50	412	NA	42	390
37	DH-E054	77	80	76	125	45	432	NA	40	400
38	DH-E055	77	79	86	128	49	445	NA	41	410
39	DH-E056	76	84	82	133	49	458	NA	38	415
40	DH-E058	80	75	84	130	55	431	NA	38	380
41	DH-E059	76	80	75	128	48	443	NA	39	406
42	DH-E060	76	82	70	131	49	447	NA	40	414
43	DH-E061	79	78	83	130	52	417	NA	37	391
44	DH-E062	80	80	95	131	51	413	NA	39	395
45	DH-E063	75	82	95	130	48	422	NA	38	395
46	DH-E065	78	80	89	132	52	406	NA	41	354
47	DH-E066	74	81	82	132	51	446	NA	38	415
48	DH-E067	76	82	82	128	46	436	NA	38	428
CHK1	Chk 1 (WH147)	75	80	80	132	52	424	NA	38	390
CHK2	Chk 2 (PBW175)	78	86	89	127	41	394	NA	37	370
CHK3	Chk 3 (NI5439)	75	78	96	128	50	406	NA	38	382
49	DH-E068	80	77	83	127	50	394	NA	39	356
50	DH-E069	79	76	83	125	49	396	NA	38	366
51	DH-E070	80	80	75	130	50	430	NA	40	403
52	DH-E071	77	81	82	130	49	414	NA	38	378
53	DH-E072	75	82	70	125	43	438	NA	37	410
54	DH-E073	78	82	80	132	50	443	NA	37	411

55	DH-E074	80	76	83	128	52	443	NA	36	405
56	DH-E075	79	79	78	125	46	427	NA	38	415
57	DH-E076	75	86	73	136	50	428	NA	38	386
58	DH-E077	75	82	77	125	43	401	NA	40	384
59	DH-E078	76	82	81	132	50	370	NA	44	352
60	DH-E080	80	81	80	126	45	375	NA	41	347
61	DH-E081	82	82	80	132	50	435	NA	42	370
62	DH-E082	79	82	78	125	43	365	NA	41	350
63	DH-E083	80	82	80	132	50	356	NA	38	340
64	DH-E084	75	83	82	130	47	413	NA	38	378
CHK1	Chk 1 (WH147)	80	85	75	134	49	350	NA	42	328
CHK2	Chk 2 (PBW175)	70	88	94	142	54	440	NA	42	406
CHK3	Chk 3 (NI5439)	78	86	82	145	59	434	NA	41	408
65	DH-E086	80	80	75	128	48	429	NA	37	405
66	DH-E087	76	79	66	130	51	431	NA	37	378
67	DH-E088	75	79	77	125	46	355	NA	39	235
68	DH-E090	78	94	74	141	47	365	NA	41	410
69	DH-E091	80	83	70	135	52	355	NA	40	340
70	DH-E092	78	83	68	131	48	425	NA	42	335
71	DH-E093	80	84	70	134	50	380	NA	42	332
72	DH-E094	78	81	84	120	39	384	NA	41	347
73	DH-E095	80	82	65	132	50	330	NA	37	302
74	DH-E096	80	82	95	131	49	325	NA	37	302
75	DH-E097	76	83	75	132	49	328	NA	40	312
76	DH-E098	80	84	84	135	51	450	NA	39	402
77	DH-E099	76	81	77	131	50	422	NA	38	387
78	DH-E100	75	82	71	120	38	406	NA	42	417
79	DH-E102	76	83	78	124	41	390	NA	39	361
80	DH-E103	86	81	72	123	42	398	NA	39	307
CHK1	Chk 1 (WH147)	81	84	70	133	49	375	NA	38	352
CHK2	Chk 2 (PBW175)	75	93	75	143	50	435	NA	38	415
CHK3	Chk 3 (NI5439)	76	98	78	145	47	430	NA	40	410
81	DH-E104	80	81	70	124	43	428	NA	40	300
82	DH-E105	75	80	69	120	40	431	NA	38	408
83	DH-E108	80	78	69	120	42	428	NA	40	405
84	DH-E109	75	79	70	123	44	401	NA	38	375
85	DH-E110	76	81	67	124	43	327	NA	37	300
86	DH-E111	76	83	69	132	49	320	NA	38	295
87	DH-E113	78	92	89	138	46	356	NA	39	335
88	DH-E114	80	88	82	136	48	415	NA	41	390
89	DH-E115	76	84	88	127	43	463	NA	40	425
90	DH-E117	80	86	90	134	48	469	NA	41	432
91	DH-E118	82	76	89	119	43	442	NA	38	397
92	DH-E119	80	79	79	122	43	453	NA	40	425
93	DH-E120	75	78	78	127	49	446	NA	42	419
94	DH-E121	80	86	86	129	43	449	NA	37	407
95	DH-E122	81	86	86	129	43	444	NA	38	402
96	DH-E123	82	89	89	132	43	451	NA	40	408
CHK1	Chk 1 (WH147)	78	78	78	121	43	458	NA	41	426
CHK2	Chk 2 (PBW175)	82	78	78	121	43	395	NA	40	365

CHK3	Chk 3 (NI5439)	80	82	82	125	43	459	NA	41	425
97	DH-E124	76	84	84	132	48	448	NA	39	417
98	DH-E125	81	80	80	123	43	425	NA	37	395
99	DH-E126	80	82	82	125	43	441	NA	40	405
100	DH-E127	82	79	85	122	43	447	NA	40	410
101	DH-E129	76	81	87	124	43	456	NA	41	418
102	DH-E130	79	86	86	129	43	454	NA	39	419
103	DH-E131	82	86	86	136	50	426	NA	40	391
104	DH-E132	77	68	74	111	43	467	NA	37	429
105	DH-E133	75	82	82	125	43	460	NA	42	427
106	DH-E134	78	78	78	121	43	461	NA	41	429
107	DH-E135	82	85	85	135	50	450	NA	41	416
108	DH-E137	77	77	77	120	43	469	NA	40	436
109	DH-E138	76	84	84	127	43	395	NA	41	361
110	DH-E141	82	86	86	134	48	463	NA	42	430
111	DH-E144	77	83	83	126	43	414	NA	41	385
112	DH-E145	82	77	77	120	43	468	NA	42	435
CHK1	Chk 1 (WH147)	86	79	79	131	52	464	NA	42	428
CHK2	Chk 2 (PBW175)	80	84	84	127	43	421	NA	38	386
CHK3	Chk 3 (NI5439)	78	81	81	124	43	439	NA	41	406
113	DH-E146	77	86	86	135	49	449	NA	46	414
114	DH-E147	82	84	84	127	43	419	NA	42	383
115	DH-E148	83	83	83	126	43	471	NA	40	439
116	DH-E149	80	86	86	134	48	451	NA	41	419
117	DH-E150	79	84	84	133	49	438	NA	40	406
118	DH-E152	82	78	78	121	43	449	NA	40	414
119	DH-E153	77	79	79	122	43	457	NA	43	424
120	DH-E154	72	70	78	115	45	452	NA	44	411
121	DH-E155	82	75	75	118	43	445	NA	42	411
122	DH-E156	82	81	81	124	43	447	NA	41	414
123	DH-E158	75	82	74	125	43	445	NA	40	412
124	DH-E159	78	85	77	128	43	442	NA	39	407
125	DH-E161	79	82	88	125	43	474	NA	44	445
126	DH-E162	80	85	83	128	43	445	NA	40	411
127	DH-E164	73	82	84	125	43	450	NA	39	419
128	DH-E165	80	73	70	116	43	394	NA	38	355
CHK1	Chk 1 (WH147)	83	82	63	125	43	449	NA	41	414
CHK2	Chk 2 (PBW175)	78	78	96	115	37	468	NA	43	436
CHK3	Chk 3 (NI5439)	76	86	82	129	43	466	NA	42	432
129	DH-E166	82	78	76	121	43	419	NA	38	379
130	DH-E167	78	85	81	133	48	448	NA	39	414
131	DH-E168	80	81	82	123	42	442	NA	37	409
132	DH-E169	82	78	76	121	43	438	NA	38	407
133	DH-E170	76	85	84	134	49	466	NA	42	434
134	DH-E171	78	81	83	124	43	454	NA	41	422
135	DH-E172	80	86	85	133	47	458	NA	39	423
136	DH-E175	84	79	71	122	43	436	NA	37	402
137	DH-E176	80	74	66	118	44	436	NA	37	406
138	DH-E177	74	81	78	124	43	451	NA	39	411
139	DH-E178	75	84	76	129	45	466	NA	43	430

140	DH-E179	76	82	74	125	43	451	NA	41	417
141	DH-E180	78	85	83	128	43	472	NA	37	438
142	DH-E181	80	84	71	131	47	434	NA	39	403
143	DH-E182	72	85	77	133	48	446	NA	41	410
144	DH-E183	78	77	73	120	43	426	NA	37	394
CHK1	Chk 1 (WH147)	75	85	95	130	45	462	NA	42	428
CHK2	Chk 2 (PBW175)	75	86	84	133	47	507	NA	45	474
CHK3	Chk 3 (NI5439)	78	84	81	127	43	492	NA	43	457
145	DH-E184	80	84	88	132	48	469	NA	42	438
146	DH-E185	76	73	72	116	43	419	NA	37	382
147	DH-E186	75	76	68	120	44	427	NA	37	394
148	DH-E187	80	82	84	125	43	459	NA	39	428
149	DH-E188	82	80	77	123	43	448	NA	38	415
150	DH-E189	76	83	80	127	44	423	NA	38	384
151	DH-E190	80	83	81	124	41	443	NA	37	402
152	DH-E191	75	80	84	123	43	458	NA	38	426
153	DH-E192	82	80	83	126	46	413	NA	45	380
154	DH-E193	81	85	77	128	43	432	NA	43	396
155	DH-E194	80	81	77	124	43	466	NA	43	433
156	DH-E195	81	86	97	134	48	443	NA	42	410
157	DH-E196	77	85	95	132	47	467	NA	37	433
158	DH-E197	82	79	61	126	47	466	NA	42	421
159	DH-E198	80	82	62	132	50	474	NA	41	428
160	DH-E199	76	82	54	130	48	459	NA	41	412
CHK1	Chk 1 (WH147)	78	81	67	132	51	435	NA	39	383
CHK2	Chk 2 (PBW175)	79	78	84	125	47	425	NA	43	400
CHK3	Chk 3 (NI5439)	82	86	89	137	51	431	NA	44	414
161	DH-E200	75	82	59	129	47	473	NA	42	422
162	DH-E202	80	77	51	124	47	457	NA	40	415
163	DH-E203	75	81	51	128	47	410	NA	36	365
164	DH-E204	76	79	51	126	47	427	NA	38	380
165	DH-E205	78	82	57	130	48	464	NA	41	416
166	DH-E206	80	77	56	123	46	453	NA	38	404
167	DH-E207	75	70	82	117	47	450	NA	37	399
168	DH-E208	78	78	72	132	54	478	NA	38	431
169	DH-E209	80	73	63	122	49	460	NA	41	413
170	DH-E210	75	75	58	122	47	450	NA	40	402
171	DH-E211	78	79	70	127	48	472	NA	42	425
172	DH-E213	77	73	65	121	48	446	NA	39	397
173	DH-E214	76	78	77	125	47	489	NA	43	442
174	DH-E215	75	79	72	130	51	448	NA	38	399
175	DH-E216	80	82	70	129	47	460	NA	38	409
176	DH-E217	78	86	66	133	47	422	NA	43	409
CHK1	Chk 1 (WH147)	79	82	99	143	61	470	NA	45	407
CHK2	Chk 2 (PBW175)	80	81	100	134	53	457	NA	39	434
CHK3	Chk 3 (NI5439)	76	83	102	130	47	422	NA	43	400
177	DH-E218	80	82	69	129	47	450	NA	40	428
178	DH-E219	80	79	67	126	47	477	NA	41	428
179	DH-E220	78	74	61	123	49	469	NA	42	420
180	DH-E221	77	69	66	116	47	434	NA	38	392

181	DH-E222	75	78	72	125	47	493	NA	44	446
182	DH-E223	77	75	62	121	46	453	NA	38	409
183	DH-E224	76	89	65	140	51	481	NA	41	434
184	DH-E226	75	86	68	135	49	418	NA	42	432
185	DH-E227	79	84	60	131	47	447	NA	39	438
186	DH-E228	80	84	62	131	47	460	NA	41	423
187	DH-E229	76	85	61	135	50	460	NA	42	433
188	DH-E230	77	76	57	123	47	445	NA	42	450
189	DH-E231	75	80	64	127	47	488	NA	40	437
190	DH-E232	80	86	80	136	50	460	NA	32	440
191	DH-E233	77	81	84	133	52	451	NA	39	417
192	DH-E233	76	85	108	132	47	440	NA	44	425
**Location**	Karnal									
**Crop-season**	2012-13									
**Condition**	Rainfed									
**S.No.**	**DH-2 Line code**	**GP***	**DTA**	**PH**	**DTM**	**GFD**	**PTPM**	**GWPE**	**TGW***	**GYPP**
CHK1	Chk 1 (WH147)	85	94	NA	118	24	186	NA	32	219
CHK2	Chk 2 (PBW175)	80	94	NA	118	24	170	NA	38	269
CHK3	Chk 3 (NI5439)	90	92	NA	112	20	139	NA	44	390
1	DH-E001	90	92	NA	112	20	198	NA	24	157
2	DH-E003	90	99	NA	125	26	165	NA	28	356
3	DH-E005	85	92	NA	112	20	173	NA	31	180
4	DH-E006	95	99	NA	125	26	125	NA	26	188
5	DH-E007	80	92	NA	112	20	145	NA	30	270
6	DH-E008	85	92	NA	112	20	130	NA	28	165
7	DH-E009	85	99	NA	126	27	129	NA	28	158
8	DH-E010	80	99	NA	126	27	140	NA	29	249
9	DH-E014	95	94	NA	118	24	156	NA	29	200
10	DH-E015	90	87	NA	110	23	172	NA	24	182
11	DH-E018	85	87	NA	110	23	190	NA	36	270
12	DH-E019	80	99	NA	125	26	156	NA	30	199
13	DH-E020	90	87	NA	110	23	168	NA	30	284
14	DH-E021	90	87	NA	110	23	246	NA	30	300
15	DH-E022	85	94	NA	117	23	156	NA	27	193
16	DH-E023	80	96	NA	119	23	145	NA	20	120
CHK1	Chk 1 (WH147)	80	94	NA	115	21	162	NA	31	294
CHK2	Chk 2 (PBW175)	75	94	NA	115	21	178	NA	40	320
CHK3	Chk 3 (NI5439)	85	94	NA	117	23	208	NA	46	437
17	DH-E024	80	94	NA	116	22	180	NA	31	284
18	DH-E025	85	94	NA	116	22	220	NA	32	284
19	DH-E026	90	96	NA	119	23	209	NA	29	309
20	DH-E029	85	96	NA	118	22	165	NA	25	206
21	DH-E032	90	94	NA	117	23	125	NA	31	280
22	DH-E033	80	87	NA	111	24	156	NA	32	258
23	DH-E034	85	87	NA	111	24	125	NA	34	297
24	DH-E036	85	87	NA	111	24	139	NA	30	270
25	DH-E038	85	98	NA	120	22	220	NA	27	183
26	DH-E039	85	85	NA	108	23	184	NA	32	377
27	DH-E040	80	98	NA	117	19	256	NA	22	99
28	DH-E041	95	94	NA	116	22	180	NA	27	222

29	DH-E042	95	102	NA	126	24	215	NA	20	109
30	DH-E043	90	96	NA	118	22	183	NA	32	203
31	DH-E044	75	99	NA	125	26	165	NA	24	136
32	DH-E046	95	98	NA	120	22	173	NA	25	200
CHK1	Chk 1 (WH147)	90	83	NA	106	23	142	NA	22	139
CHK2	Chk 2 (PBW175)	90	94	NA	116	22	230	NA	28	318
CHK3	Chk 3 (NI5439)	95	90	NA	112	22	144	NA	30	309
33	DH-E047	85	98	NA	120	22	154	NA	26	152
34	DH-E051	75	88	NA	111	23	190	NA	34	226
35	DH-E052	90	90	NA	112	22	215	NA	29	234
36	DH-E053	80	102	NA	126	24	185	NA	28	212
37	DH-E054	75	83	NA	106	23	178	NA	25	176
38	DH-E055	80	94	NA	116	22	148	NA	26	169
39	DH-E056	85	102	NA	126	24	235	NA	26	240
40	DH-E058	80	83	NA	106	23	156	NA	23	207
41	DH-E059	85	90	NA	112	22	214	NA	29	220
42	DH-E060	85	88	NA	111	23	148	NA	30	188
43	DH-E061	85	88	NA	111	23	245	NA	29	233
44	DH-E062	90	97	NA	119	22	135	NA	39	278
45	DH-E063	85	96	NA	119	23	183	NA	49	489
46	DH-E065	85	87	NA	110	23	234	NA	24	190
47	DH-E066	80	96	NA	119	23	163	NA	30	198
48	DH-E067	80	96	NA	119	23	225	NA	29	218
CHK1	Chk 1 (WH147)	85	94	NA	117	23	135	NA	30	285
CHK2	Chk 2 (PBW175)	80	96	NA	119	23	138	NA	37	334
CHK3	Chk 3 (NI5439)	80	96	NA	119	23	154	NA	46	386
49	DH-E068	85	90	NA	112	22	154	NA	28	190
50	DH-E069	90	90	NA	112	22	198	NA	27	277
51	DH-E070	90	102	NA	126	24	140	NA	21	131
52	DH-E071	95	88	NA	111	23	182	NA	24	224
53	DH-E072	95	85	NA	107	22	165	NA	24	214
54	DH-E073	85	90	NA	112	22	164	NA	28	197
55	DH-E074	85	97	NA	119	22	142	NA	28	166
56	DH-E075	75	90	NA	112	22	105	NA	26	91
57	DH-E076	85	94	NA	117	23	142	NA	28	191
58	DH-E077	80	90	NA	112	22	118	NA	31	226
59	DH-E078	95	88	NA	111	23	96	NA	32	266
60	DH-E080	90	96	NA	119	23	144	NA	35	357
61	DH-E081	95	98	NA	120	22	122	NA	25	265
62	DH-E082	85	96	NA	119	23	160	NA	27	210
63	DH-E083	85	96	NA	119	23	220	NA	31	270
64	DH-E084	80	102	NA	126	24	145	NA	28	281
CHK1	Chk 1 (WH147)	95	94	NA	117	23	122	NA	32	371
CHK2	Chk 2 (PBW175)	80	96	NA	119	23	150	NA	42	619
CHK3	Chk 3 (NI5439)	80	93	NA	116	23	256	NA	45	584
65	DH-E086	75	96	NA	119	23	128	NA	31	233
66	DH-E087	95	96	NA	119	23	102	NA	31	275
67	DH-E088	90	94	NA	117	23	114	NA	31	262
68	DH-E090	90	90	NA	112	22	106	NA	30	224
69	DH-E091	90	90	NA	112	22	122	NA	30	237

70	DH-E092	95	90	NA	112	22	105	NA	30	196
71	DH-E093	80	89	NA	111	22	224	NA	25	223
72	DH-E094	85	87	NA	110	23	186	NA	22	114
73	DH-E095	85	96	NA	118	22	166	NA	35	468
74	DH-E096	80	96	NA	118	22	155	NA	26	162
75	DH-E097	90	84	NA	107	23	248	NA	35	330
76	DH-E098	95	84	NA	107	23	184	NA	32	235
77	DH-E099	80	86	NA	108	22	147	NA	30	283
78	DH-E100	85	93	NA	116	23	189	NA	26	115
79	DH-E102	90	86	NA	108	22	245	NA	30	243
80	DH-E103	80	85	NA	106	21	235	NA	33	293
CHK1	Chk 1 (WH147)	95	96	NA	118	22	178	NA	20	32
CHK2	Chk 2 (PBW175)	95	96	NA	118	22	147	NA	32	268
CHK3	Chk 3 (NI5439)	90	96	NA	118	22	193	NA	40	387
81	DH-E104	95	96	NA	118	22	238	NA	32	298
82	DH-E105	85	89	NA	111	22	187	NA	30	263
83	DH-E108	85	97	NA	118	21	225	NA	40	474
84	DH-E109	85	84	NA	107	23	144	NA	29	300
85	DH-E110	80	96	NA	118	22	198	NA	25	223
86	DH-E111	80	89	NA	111	22	210	NA	27	245
87	DH-E113	95	96	NA	118	22	225	NA	31	221
88	DH-E114	90	98	NA	124	26	136	NA	28	239
89	DH-E115	95	102	NA	127	25	186	NA	31	354
90	DH-E117	85	102	NA	127	25	270	NA	32	312
91	DH-E118	85	98	NA	124	26	135	NA	28	282
92	DH-E119	80	84	NA	107	23	120	NA	26	209
93	DH-E120	85	89	NA	111	22	236	NA	28	174
94	DH-E121	85	99	NA	124	25	158	NA	25	153
95	DH-E122	80	96	NA	118	22	145	NA	26	176
96	DH-E123	95	99	NA	124	25	153	NA	32	221
CHK1	Chk 1 (WH147)	85	96	NA	118	22	194	NA	26	90
CHK2	Chk 2 (PBW175)	80	93	NA	116	23	224	NA	34	280
CHK3	Chk 3 (NI5439)	85	97	NA	118	21	265	NA	49	509
97	DH-E124	90	89	NA	111	22	230	NA	26	322
98	DH-E125	95	89	NA	111	22	248	NA	28	165
99	DH-E126	85	96	NA	118	22	156	NA	32	278
100	DH-E127	90	93	NA	116	23	113	NA	32	238
101	DH-E129	95	93	NA	116	23	137	NA	28	222
102	DH-E130	75	95	NA	118	23	260	NA	23	154
103	DH-E131	85	86	NA	108	22	237	NA	28	226
104	DH-E132	85	97	NA	118	21	210	NA	24	165
105	DH-E133	80	99	NA	124	25	176	NA	22	159
106	DH-E134	80	95	NA	118	23	184	NA	25	147
107	DH-E135	95	93	NA	116	23	225	NA	28	286
108	DH-E137	95	102	NA	125	23	253	NA	26	243
109	DH-E138	90	93	NA	116	23	153	NA	32	204
110	DH-E141	95	93	NA	116	23	177	NA	29	282
111	DH-E144	80	95	NA	118	23	256	NA	33	160
112	DH-E145	85	99	NA	124	25	164	NA	23	125
CHK1	Chk 1 (WH147)	95	93	NA	116	23	173	NA	27	71

CHK2	Chk 2 (PBW175)	90	93	NA	116	23	125	NA	38	240
CHK3	Chk 3 (NI5439)	85	89	NA	111	22	180	NA	48	399
113	DH-E146	85	102	NA	125	23	142	NA	32	229
114	DH-E147	85	96	NA	118	22	222	NA	38	355
115	DH-E148	80	93	NA	116	23	192	NA	30	244
116	DH-E149	95	96	NA	118	22	156	NA	36	234
117	DH-E150	90	93	NA	116	23	172	NA	27	194
118	DH-E152	85	89	NA	111	22	174	NA	34	341
119	DH-E153	80	96	NA	118	22	184	NA	20	97
120	DH-E154	80	88	NA	109	21	176	NA	26	263
121	DH-E155	90	86	NA	109	23	135	NA	37	335
122	DH-E156	90	95	NA	118	23	175	NA	30	338
123	DH-E158	95	95	NA	118	23	215	NA	28	402
124	DH-E159	80	91	NA	111	20	263	NA	24	133
125	DH-E161	85	91	NA	111	20	184	NA	24	160
126	DH-E162	85	98	NA	124	26	156	NA	24	169
127	DH-E164	80	93	NA	116	23	188	NA	21	116
128	DH-E165	80	89	NA	111	22	157	NA	23	153
CHK1	Chk 1 (WH147)	80	95	NA	118	23	175	NA	24	124
CHK2	Chk 2 (PBW175)	85	93	NA	116	23	194	NA	40	343
CHK3	Chk 3 (NI5439)	85	95	NA	116	21	183	NA	47	511
129	DH-E166	85	98	NA	124	26	175	NA	23	147
130	DH-E167	85	95	NA	118	23	170	NA	23	116
131	DH-E168	85	102	NA	127	25	165	NA	22	66
132	DH-E169	90	91	NA	111	20	242	NA	31	410
133	DH-E170	90	91	NA	111	20	175	NA	21	37
134	DH-E171	95	86	NA	110	24	180	NA	31	167
135	DH-E172	90	86	NA	110	24	130	NA	24	168
136	DH-E175	80	95	NA	118	23	173	NA	32	308
137	DH-E176	85	93	NA	111	18	217	NA	26	230
138	DH-E177	90	102	NA	127	25	234	NA	24	110
139	DH-E178	90	86	NA	109	23	115	NA	28	147
140	DH-E179	85	84	NA	107	23	180	NA	27	270
141	DH-E180	80	84	NA	107	23	178	NA	46	290
142	DH-E181	90	96	NA	118	22	185	NA	31	193
143	DH-E182	95	96	NA	118	22	232	NA	29	204
144	DH-E183	85	89	NA	111	22	211	NA	26	156
CHK1	Chk 1 (WH147)	85	85	NA	107	22	210	NA	43	436
CHK2	Chk 2 (PBW175)	85	100	NA	125	25	185	NA	28	184
CHK3	Chk 3 (NI5439)	80	86	NA	109	23	164	NA	30	208
145	DH-E184	85	89	NA	111	22	137	NA	26	169
146	DH-E185	80	89	NA	111	22	175	NA	34	382
147	DH-E186	90	86	NA	109	23	175	NA	27	212
148	DH-E187	85	86	NA	109	23	182	NA	25	205
149	DH-E188	85	100	NA	125	25	242	NA	30	269
150	DH-E189	80	100	NA	125	25	265	NA	27	189
151	DH-E190	95	100	NA	125	25	198	NA	30	353
152	DH-E191	95	86	NA	109	23	164	NA	27	283
153	DH-E192	90	84	NA	107	23	186	NA	25	213
154	DH-E193	95	86	NA	109	23	224	NA	24	181

155	DH-E194	90	86	NA	109	23	168	NA	30	192
156	DH-E195	80	86	NA	109	23	180	NA	28	50
157	DH-E196	80	84	NA	107	23	245	NA	38	224
158	DH-E197	90	89	NA	111	22	215	NA	29	200
159	DH-E198	95	89	NA	111	22	156	NA	26	240
160	DH-E199	95	89	NA	111	22	168	NA	27	270
CHK1	Chk 1 (WH147)	85	93	NA	116	23	260	NA	28	255
CHK2	Chk 2 (PBW175)	85	99	NA	124	25	263	NA	41	192
CHK3	Chk 3 (NI5439)	80	101	NA	125	24	184	NA	47	262
161	DH-E200	85	102	NA	127	25	172	NA	20	72
162	DH-E202	80	86	NA	109	23	173	NA	26	221
163	DH-E203	85	84	NA	107	23	241	NA	43	256
164	DH-E204	85	102	NA	127	25	168	NA	18	76
165	DH-E205	85	84	NA	107	23	125	NA	28	166
166	DH-E206	75	86	NA	109	23	168	NA	36	231
167	DH-E207	80	86	NA	109	23	184	NA	34	373
168	DH-E208	80	93	NA	116	23	214	NA	27	215
169	DH-E209	85	89	NA	111	22	208	NA	27	241
170	DH-E210	95	89	NA	111	22	241	NA	27	230
171	DH-E211	90	86	NA	109	23	164	NA	26	182
172	DH-E213	80	86	NA	109	23	194	NA	29	277
173	DH-E214	95	86	NA	109	23	173	NA	22	82
174	DH-E215	95	89	NA	111	22	235	NA	24	140
175	DH-E216	90	101	NA	125	24	246	NA	22	142
176	DH-E217	80	89	NA	111	22	97	NA	30	216
CHK1	Chk 1 (WH147)	80	96	NA	118	22	165	NA	26	57
CHK2	Chk 2 (PBW175)	90	97	NA	118	21	196	NA	33	204
CHK3	Chk 3 (NI5439)	95	99	NA	124	25	190	NA	49	429
177	DH-E218	85	91	NA	111	20	160	NA	26	233
178	DH-E219	80	89	NA	111	22	175	NA	40	129
179	DH-E220	85	102	NA	127	25	192	NA	29	297
180	DH-E221	95	89	NA	111	22	180	NA	23	132
181	DH-E222	90	95	NA	118	23	185	NA	27	266
182	DH-E223	95	86	NA	108	22	188	NA	27	230
183	DH-E224	90	88	NA	111	23	176	NA	31	213
184	DH-E226	85	102	NA	127	25	178	NA	30	386
185	DH-E227	80	89	NA	111	22	263	NA	36	333
186	DH-E228	85	102	NA	127	25	256	NA	33	345
187	DH-E229	90	89	NA	111	22	153	NA	35	291
188	DH-E230	95	86	NA	108	22	136	NA	32	320
189	DH-E231	85	86	NA	108	22	148	NA	32	245
190	DH-E232	80	101	NA	125	24	195	NA	34	438
191	DH-E233	85	86	NA	108	22	190	NA	36	500
192	DH-E233	85	96	NA	118	22	146	NA	34	159
**Location**	Karnal									
**Crop-season**	2012-13									
**Condition**	Irrigated									
**S.No.**	**DH-2 Line code**	**GP***	**DTA**	**PH**	**DTM**	**GFD**	**PTPM**	**GWPE**	**TGW***	**GYPP**
CHK1	Chk 1 (WH147)	95	97	NA	118	21	146	NA	31	289
CHK2	Chk 2 (PBW175)	95	97	NA	118	21	241	NA	36	28

CHK3	Chk 3 (NI5439)	90	96	NA	118	22	239	NA	48	559
1	DH-E001	90	90	NA	110	20	168	NA	26	251
2	DH-E003	95	102	NA	126	24	158	NA	28	422
3	DH-E005	95	102	NA	126	24	220	NA	36	271
4	DH-E006	80	96	NA	118	22	242	NA	28	252
5	DH-E007	80	94	NA	117	23	165	NA	34	402
6	DH-E008	90	90	NA	110	20	130	NA	26	207
7	DH-E009	95	97	NA	118	21	173	NA	32	392
8	DH-E010	85	102	NA	126	24	158	NA	32	312
9	DH-E014	85	94	NA	116	22	148	NA	32	41
10	DH-E015	95	92	NA	116	24	127	NA	24	151
11	DH-E018	90	90	NA	110	20	100	NA	35	205
12	DH-E019	85	96	NA	118	22	156	NA	32	295
13	DH-E020	90	87	NA	110	23	152	NA	31	32
14	DH-E021	95	87	NA	110	23	138	NA	29	391
15	DH-E022	90	97	NA	118	21	213	NA	30	330
16	DH-E023	90	96	NA	118	22	186	NA	27	392
CHK1	Chk 1 (WH147)	95	94	NA	115	21	132	NA	29	214
CHK2	Chk 2 (PBW175)	95	94	NA	115	21	160	NA	36	186
CHK3	Chk 3 (NI5439)	80	95	NA	115	20	122	NA	49	468
17	DH-E024	95	87	NA	106	19	154	NA	27	433
18	DH-E025	85	87	NA	106	19	143	NA	30	433
19	DH-E026	85	101	NA	117	16	184	NA	30	520
20	DH-E029	90	90	NA	112	22	224	NA	25	274
21	DH-E032	80	93	NA	115	22	138	NA	30	381
22	DH-E033	85	87	NA	106	19	122	NA	28	217
23	DH-E034	85	87	NA	106	19	109	NA	35	295
24	DH-E036	80	87	NA	106	19	188	NA	36	447
25	DH-E038	90	100	NA	125	25	176	NA	32	266
26	DH-E039	95	90	NA	112	22	184	NA	36	434
27	DH-E040	85	100	NA	125	25	224	NA	32	158
28	DH-E041	80	98	NA	119	21	275	NA	28	301
29	DH-E042	85	98	NA	119	21	245	NA	20	208
30	DH-E043	80	97	NA	118	21	230	NA	33	271
31	DH-E044	90	100	NA	125	25	172	NA	24	264
32	DH-E046	95	102	NA	126	24	147	NA	24	231
CHK1	Chk 1 (WH147)	90	90	NA	112	22	172	NA	28	224
CHK2	Chk 2 (PBW175)	95	100	NA	125	25	164	NA	30	321
CHK3	Chk 3 (NI5439)	95	98	NA	119	21	165	NA	32	386
33	DH-E047	90	97	NA	118	21	186	NA	24	284
34	DH-E051	85	96	NA	118	22	147	NA	38	319
35	DH-E052	80	96	NA	118	22	167	NA	35	398
36	DH-E053	90	100	NA	125	25	172	NA	28	279
37	DH-E054	75	95	NA	116	21	182	NA	26	267
38	DH-E055	80	102	NA	126	24	180	NA	28	264
39	DH-E056	85	90	NA	112	22	210	NA	25	334
40	DH-E058	90	102	NA	126	24	160	NA	28	422
41	DH-E059	85	93	NA	113	20	158	NA	34	323
42	DH-E060	85	96	NA	118	22	156	NA	38	385
43	DH-E061	90	100	NA	125	25	222	NA	30	294

44	DH-E062	95	100	NA	125	25	164	NA	38	202
45	DH-E063	90	100	NA	125	25	230	NA	49	62
46	DH-E065	90	92	NA	113	21	170	NA	31	365
47	DH-E066	95	101	NA	125	24	169	NA	36	379
48	DH-E067	95	101	NA	125	24	150	NA	36	41
CHK1	Chk 1 (WH147)	80	98	NA	119	21	125	NA	27	183
CHK2	Chk 2 (PBW175)	80	102	NA	126	24	138	NA	36	242
CHK3	Chk 3 (NI5439)	85	98	NA	119	21	184	NA	40	489
49	DH-E068	90	90	NA	112	22	171	NA	32	311
50	DH-E069	90	96	NA	118	22	114	NA	31	39
51	DH-E070	95	90	NA	112	22	145	NA	24	271
52	DH-E071	95	90	NA	112	22	172	NA	28	318
53	DH-E072	90	87	NA	110	23	146	NA	26	276
54	DH-E073	80	94	NA	116	22	124	NA	30	248
55	DH-E074	85	102	NA	126	24	103	NA	29	332
56	DH-E075	90	98	NA	119	21	169	NA	20	208
57	DH-E076	85	95	NA	116	21	145	NA	24	271
58	DH-E077	90	87	NA	106	19	127	NA	28	218
59	DH-E078	95	87	NA	106	19	171	NA	28	306
60	DH-E080	85	102	NA	126	24	163	NA	33	406
61	DH-E081	90	100	NA	125	25	146	NA	26	249
62	DH-E082	90	98	NA	119	21	172	NA	25	76
63	DH-E083	95	98	NA	119	21	147	NA	30	311
64	DH-E084	85	102	NA	126	24	168	NA	44	325
CHK1	Chk 1 (WH147)	95	102	NA	126	24	158	NA	24	99
CHK2	Chk 2 (PBW175)	90	97	NA	118	21	148	NA	32	377
CHK3	Chk 3 (NI5439)	85	100	NA	125	25	137	NA	47	463
65	DH-E086	80	96	NA	118	22	146	NA	29	347
66	DH-E087	85	96	NA	118	22	172	NA	28	268
67	DH-E088	85	87	NA	106	19	168	NA	27	269
68	DH-E090	85	87	NA	106	19	142	NA	26	248
69	DH-E091	90	87	NA	106	19	139	NA	27	219
70	DH-E092	95	87	NA	106	19	156	NA	27	278
71	DH-E093	85	87	NA	106	19	130	NA	24	182
72	DH-E094	85	90	NA	112	22	140	NA	23	214
73	DH-E095	85	99	NA	123	24	147	NA	35	52
74	DH-E096	80	102	NA	126	24	185	NA	25	236
75	DH-E097	95	87	NA	106	19	156	NA	34	384
76	DH-E098	90	87	NA	106	19	120	NA	35	347
77	DH-E099	90	87	NA	106	19	165	NA	36	404
78	DH-E100	80	101	NA	125	24	170	NA	22	272
79	DH-E102	85	100	NA	125	25	220	NA	29	328
80	DH-E103	90	100	NA	125	25	184	NA	31	342
CHK1	Chk 1 (WH147)	80	98	NA	119	21	169	NA	25	10
CHK2	Chk 2 (PBW175)	80	98	NA	119	21	170	NA	37	235
CHK3	Chk 3 (NI5439)	80	100	NA	125	25	134	NA	49	509
81	DH-E104	95	102	NA	126	24	212	NA	31	339
82	DH-E105	95	90	NA	112	22	172	NA	28	271
83	DH-E108	95	88	NA	110	22	146	NA	36	331
84	DH-E109	90	90	NA	112	22	173	NA	27	29

85	DH-E110	95	98	NA	119	21	164	NA	26	336
86	DH-E111	90	87	NA	106	19	173	NA	27	275
87	DH-E113	85	100	NA	125	25	120	NA	36	342
88	DH-E114	85	102	NA	126	24	156	NA	28	406
89	DH-E115	85	98	NA	119	21	170	NA	28	34
90	DH-E117	80	98	NA	119	21	145	NA	28	366
91	DH-E118	85	98	NA	119	21	140	NA	28	433
92	DH-E119	95	102	NA	126	24	85	NA	29	291
93	DH-E120	90	102	NA	126	24	148	NA	28	376
94	DH-E121	90	102	NA	126	24	163	NA	23	216
95	DH-E122	95	100	NA	125	25	144	NA	26	239
96	DH-E123	95	100	NA	125	25	138	NA	28	328
CHK1	Chk 1 (WH147)	85	98	NA	119	21	168	NA	30	355
CHK2	Chk 2 (PBW175)	80	98	NA	119	21	170	NA	38	342
CHK3	Chk 3 (NI5439)	85	100	NA	125	25	156	NA	50	511
97	DH-E124	90	86	NA	106	20	140	NA	28	30
98	DH-E125	80	86	NA	106	20	164	NA	30	323
99	DH-E126	85	100	NA	125	25	120	NA	31	40
100	DH-E127	85	103	NA	128	25	166	NA	31	359
101	DH-E129	80	103	NA	128	25	171	NA	30	358
102	DH-E130	80	100	NA	125	25	164	NA	26	36
103	DH-E131	90	88	NA	110	22	173	NA	28	355
104	DH-E132	85	101	NA	125	24	166	NA	25	245
105	DH-E133	95	101	NA	125	24	144	NA	24	25
106	DH-E134	95	95	NA	118	23	120	NA	26	282
107	DH-E135	90	96	NA	118	22	142	NA	28	343
108	DH-E137	90	102	NA	126	24	138	NA	30	332
109	DH-E138	85	96	NA	118	22	154	NA	36	267
110	DH-E141	80	90	NA	110	20	145	NA	28	362
111	DH-E144	85	102	NA	127	25	178	NA	32	458
112	DH-E145	80	103	NA	128	25	173	NA	27	288
CHK1	Chk 1 (WH147)	85	102	NA	127	25	112	NA	25	141
CHK2	Chk 2 (PBW175)	85	102	NA	127	25	127	NA	37	295
CHK3	Chk 3 (NI5439)	85	102	NA	127	25	173	NA	48	511
113	DH-E146	90	100	NA	125	25	184	NA	31	336
114	DH-E147	95	100	NA	125	25	166	NA	38	359
115	DH-E148	90	102	NA	126	24	148	NA	33	34
116	DH-E149	90	100	NA	125	25	164	NA	40	403
117	DH-E150	95	102	NA	127	25	170	NA	30	345
118	DH-E152	95	96	NA	118	22	138	NA	37	256
119	DH-E153	85	103	NA	128	25	188	NA	22	278
120	DH-E154	80	103	NA	128	25	142	NA	26	271
121	DH-E155	85	96	NA	117	21	120	NA	36	288
122	DH-E156	80	98	NA	119	21	154	NA	28	388
123	DH-E158	80	96	NA	117	21	148	NA	28	413
124	DH-E159	95	84	NA	106	22	137	NA	25	145
125	DH-E161	95	84	NA	106	22	120	NA	25	166
126	DH-E162	90	93	NA	113	20	184	NA	30	328
127	DH-E164	90	102	NA	128	26	178	NA	24	184
128	DH-E165	95	93	NA	113	20	156	NA	26	268

CHK1	Chk 1 (WH147)	90	100	NA	125	25	260	NA	30	198
CHK2	Chk 2 (PBW175)	95	97	NA	118	21	153	NA	33	85
CHK3	Chk 3 (NI5439)	95	97	NA	118	21	210	NA	49	432
129	DH-E166	95	102	NA	128	26	144	NA	25	341
130	DH-E167	90	102	NA	128	26	181	NA	24	318
131	DH-E168	85	93	NA	113	20	165	NA	27	173
132	DH-E169	85	102	NA	126	24	180	NA	34	407
133	DH-E170	80	96	NA	117	21	225	NA	27	218
134	DH-E171	85	96	NA	128	32	170	NA	32	237
135	DH-E172	85	85	NA	110	25	175	NA	27	305
136	DH-E175	80	97	NA	118	21	165	NA	37	378
137	DH-E176	85	88	NA	107	19	178	NA	34	278
138	DH-E177	90	103	NA	128	25	146	NA	24	192
139	DH-E178	85	100	NA	125	25	135	NA	32	233
140	DH-E179	85	88	NA	110	22	126	NA	30	296
141	DH-E180	80	88	NA	110	22	140	NA	31	359
142	DH-E181	85	100	NA	125	25	188	NA	33	317
143	DH-E182	85	100	NA	125	25	180	NA	33	298
144	DH-E183	80	87	NA	110	23	182	NA	27	278
CHK1	Chk 1 (WH147)	90	97	NA	118	21	150	NA	48	468
CHK2	Chk 2 (PBW175)	95	103	NA	128	25	135	NA	29	311
CHK3	Chk 3 (NI5439)	95	94	NA	117	23	147	NA	30	417
145	DH-E184	80	87	NA	110	23	208	NA	35	326
146	DH-E185	80	96	NA	117	21	153	NA	34	459
147	DH-E186	95	100	NA	125	25	155	NA	30	35
148	DH-E187	95	97	NA	118	21	165	NA	28	301
149	DH-E188	90	98	NA	119	21	233	NA	29	336
150	DH-E189	90	98	NA	119	21	191	NA	28	308
151	DH-E190	90	102	NA	126	24	220	NA	34	50
152	DH-E191	95	88	NA	110	22	147	NA	33	296
153	DH-E192	90	96	NA	117	21	194	NA	26	358
154	DH-E193	95	88	NA	110	22	188	NA	28	298
155	DH-E194	90	96	NA	117	21	170	NA	30	336
156	DH-E195	95	105	NA	119	14	139	NA	25	144
157	DH-E196	95	97	NA	118	21	128	NA	38	115
158	DH-E197	90	94	NA	117	23	173	NA	28	243
159	DH-E198	90	94	NA	117	23	138	NA	30	359
160	DH-E199	95	96	NA	118	22	126	NA	33	399
CHK1	Chk 1 (WH147)	80	97	NA	118	21	184	NA	28	128
CHK2	Chk 2 (PBW175)	85	102	NA	126	24	120	NA	40	224
CHK3	Chk 3 (NI5439)	85	98	NA	119	21	158	NA	44	534
161	DH-E200	85	90	NA	112	22	153	NA	20	249
162	DH-E202	85	94	NA	117	23	140	NA	28	398
163	DH-E203	80	94	NA	117	23	210	NA	29	329
164	DH-E204	90	102	NA	126	24	215	NA	18	278
165	DH-E205	95	97	NA	118	21	164	NA	31	245
166	DH-E206	95	96	NA	118	22	152	NA	34	288
167	DH-E207	90	96	NA	118	22	130	NA	36	572
168	DH-E208	90	102	NA	126	24	165	NA	27	333
169	DH-E209	95	90	NA	112	22	180	NA	28	316

170	DH-E210	85	101	NA	118	17	155	NA	30	353
171	DH-E211	85	87	NA	110	23	150	NA	25	251
172	DH-E213	85	87	NA	110	23	139	NA	31	387
173	DH-E214	80	90	NA	112	22	176	NA	24	288
174	DH-E215	80	97	NA	118	21	180	NA	26	419
175	DH-E216	75	90	NA	112	22	176	NA	24	284
176	DH-E217	80	86	NA	110	24	143	NA	29	266
CHK1	Chk 1 (WH147)	80	101	NA	125	24	242	NA	25	184
CHK2	Chk 2 (PBW175)	95	101	NA	125	24	156	NA	35	123
CHK3	Chk 3 (NI5439)	95	101	NA	125	24	212	NA	49	534
177	DH-E218	90	101	NA	125	24	193	NA	28	332
178	DH-E219	90	90	NA	112	22	214	NA	27	362
179	DH-E220	80	94	NA	117	23	233	NA	29	402
180	DH-E221	95	94	NA	117	23	245	NA	25	285
181	DH-E222	90	100	NA	125	25	168	NA	27	381
182	DH-E223	90	98	NA	119	21	130	NA	30	244
183	DH-E224	85	97	NA	118	21	145	NA	34	206
184	DH-E226	85	94	NA	117	23	110	NA	28	316
185	DH-E227	80	94	NA	117	23	142	NA	28	208
186	DH-E228	85	101	NA	125	24	149	NA	38	431
187	DH-E229	85	96	NA	118	22	232	NA	30	375
188	DH-E230	80	96	NA	118	22	26	NA	32	397
189	DH-E231	85	90	NA	112	22	173	NA	30	191
190	DH-E232	85	102	NA	127	25	214	NA	39	433
191	DH-E233	95	94	NA	117	23	173	NA	35	385
192	DH-E233	95	102	NA	128	26	178	NA	40	594
**Location**	Pune									
**Crop-season**	2012-13									
**Condition**	Rainfed									
**S.No.**	**DH-2 Line code**	**GP***	**DTA**	**PH***	**DTM**	**GFD**	**PTPM**	**GWPE**	**TGW***	**GYPP**
CHK1	Chk 1 (WH147)	75	61	52	84	23	78	0.5	26	75
CHK2	Chk 2 (PBW175)	40	66	57	86	20	80	1.0	30	34
CHK3	Chk 3 (NI5439)	40	64	49	88	24	86	0.6	34	39
1	DH-E001	75	47	37	74	27	83	0.9	25	68
2	DH-E003	75	57	52	80	23	71	0.8	26	76
3	DH-E005	75	53	45	77	24	151	0.7	22	105
4	DH-E006	75	52	42	90	38	104	0.8	26	45
5	DH-E007	70	49	50	78	29	93	1.2	31	172
6	DH-E008	70	41	54	71	30	69	1.0	33	114
7	DH-E009	80	56	50	82	26	97	0.7	31	148
8	DH-E010	70	57	46	82	25	67	0.7	30	88
9	DH-E014	75	44	50	78	34	92	0.5	25	136
10	DH-E015	70	42	52	72	30	120	0.7	26	116
11	DH-E018	75	42	49	71	29	82	0.9	28	123
12	DH-E019	80	52	42	78	26	94	0.6	27	107
13	DH-E020	80	43	42	72	29	89	0.6	20	99
14	DH-E021	80	47	42	76	29	48	0.7	32	144
15	DH-E022	75	45	41	74	29	113	0.7	20	83
16	DH-E023	75	61	36	110	49	59	0.5	27	132
CHK1	Chk 1 (WH147)	70	63	46	86	23	78	1.1	28	61

CHK2	Chk 2 (PBW175)	75	62	58	89	27	72	1.0	34	68
CHK3	Chk 3 (NI5439)	75	61	45	90	29	98	0.5	36	19
17	DH-E024	75	42	40	71	29	99	0.5	24	144
18	DH-E025	70	41	43	71	30	81	0.6	24	68
19	DH-E026	70	75	60	109	34	130	1.2	28	228
20	DH-E029	80	67	40	93	26	138	1.3	30	112
21	DH-E032	80	54	39	80	26	90	0.5	25	68
22	DH-E033	75	46	47	68	22	70	0.8	24	88
23	DH-E034	70	43	42	71	28	60	0.9	26	173
24	DH-E036	65	47	47	80	33	64	0.8	27	73
25	DH-E038	75	53	41	77	24	85	0.6	23	140
26	DH-E039	80	51	42	74	23	75	0.7	26	153
27	DH-E040	75	52	34	78	26	96	0.4	26	135
28	DH-E041	80	57	40	77	20	100	0.5	22	145
29	DH-E042	80	59	42	83	24	102	0.6	26	151
30	DH-E043	80	49	44	76	27	89	1.1	25	165
31	DH-E044	80	67	44	94	27	55	0.6	19	127
32	DH-E046	75	57	44	82	25	95	0.9	26	145
CHK1	Chk 1 (WH147)	70	63	70	98	35	75	1.3	36	275
CHK2	Chk 2 (PBW175)	80	56	45	82	26	95	0.9	24	52
CHK3	Chk 3 (NI5439)	80	52	38	76	24	84	0.6	24	18
33	DH-E047	70	53	42	80	27	74	0.8	24	144
34	DH-E051	75	53	41	77	24	104	0.6	24	72
35	DH-E052	80	51	44	78	27	81	0.9	27	131
36	DH-E053	75	59	44	86	27	70	0.9	26	146
37	DH-E054	75	68	51	103	35	62	1.3	32	232
38	DH-E055	75	53	37	76	23	74	0.7	22	139
39	DH-E056	80	55	38	80	25	87	0.4	24	148
40	DH-E058	85	62	38	98	36	55	1.3	34	176
41	DH-E059	85	53	40	76	23	76	0.9	23	40
42	DH-E060	80	52	45	77	25	74	0.6	23	32
43	DH-E061	80	61	46	86	25	83	0.6	26	29
44	DH-E062	75	63	50	91	28	68	0.9	30	116
45	DH-E063	80	62	47	92	30	98	0.6	35	121
46	DH-E065	80	47	40	76	29	125	0.8	22	145
47	DH-E066	80	50	49	77	27	94	0.7	25	114
48	DH-E067	80	52	51	80	28	107	0.9	28	163
CHK1	Chk 1 (WH147)	75	61	56	85	24	82	1.1	26	111
CHK2	Chk 2 (PBW175)	75	60	68	86	26	77	1.2	29	85
CHK3	Chk 3 (NI5439)	75	61	56	93	32	89	0.9	36	59
49	DH-E068	80	41	50	71	30	88	0.9	26	156
50	DH-E069	80	49	51	77	28	120	0.7	29	129
51	DH-E070	85	61	42	86	25	79	0.7	26	155
52	DH-E071	85	43	45	72	29	105	0.8	22	120
53	DH-E072	80	49	52	77	28	88	0.8	26	109
54	DH-E073	80	50	48	77	27	92	0.7	31	110
55	DH-E074	75	52	49	78	26	75	0.9	27	91
56	DH-E075	80	57	57	90	33	81	1.1	34	134
57	DH-E076	55	49	54	79	30	60	1.0	27	145
58	DH-E077	75	40	53	73	33	145	1.0	27	143

59	DH-E078	75	43	51	74	31	93	0.7	28	107
60	DH-E080	65	52	54	79	27	102	0.6	29	105
61	DH-E081	65	51	52	79	28	93	0.8	23	180
62	DH-E082	75	52	49	78	26	92	0.7	28	77
63	DH-E083	70	51	51	80	29	105	0.7	29	171
64	DH-E084	70	64	45	91	27	99	0.4	25	144
CHK1	Chk 1 (WH147)	50	63	44	85	22	59	0.6	24	32
CHK2	Chk 2 (PBW175)	50	65	50	88	23	61	0.5	31	16
CHK3	Chk 3 (NI5439)	70	67	55	93	26	104	0.5	33	8
65	DH-E086	70	53	50	76	23	67	0.8	26	96
66	DH-E087	70	51	47	74	23	97	0.6	26	108
67	DH-E088	80	39	51	74	35	95	0.6	26	71
68	DH-E090	70	42	45	96	54	74	0.6	25	76
69	DH-E091	70	41	40	65	24	65	0.9	24	149
70	DH-E092	70	38	40	66	28	64	0.7	26	75
71	DH-E093	75	41	43	67	26	80	0.7	21	145
72	DH-E094	70	69	40	94	25	66	1.3	22	160
73	DH-E095	70	62	42	86	24	103	0.7	28	90
74	DH-E096	70	60	48	76	16	77	0.6	23	154
75	DH-E097	70	41	45	67	26	80	0.6	32	78
76	DH-E098	55	40	53	67	27	68	0.9	32	156
77	DH-E099	70	54	42	76	22	61	0.6	29	155
78	DH-E100	70	62	42	89	27	63	0.8	27	131
79	DH-E102	70	52	49	79	27	89	0.6	27	115
80	DH-E103	65	52	55	77	25	84	0.8	33	198
CHK1	Chk 1 (WH147)	70	62	42	86	24	93	0.4	26	23
CHK2	Chk 2 (PBW175)	70	63	54	90	27	147	0.7	32	28
CHK3	Chk 3 (NI5439)	65	67	44	95	28	67	0.2	33	8
81	DH-E104	70	52	46	80	28	95	0.7	29	77
82	DH-E105	70	43	46	78	35	94	0.6	26	167
83	DH-E108	70	52	52	76	24	138	0.6	29	103
84	DH-E109	75	43	44	74	31	91	0.5	21	99
85	DH-E110	75	53	45	78	25	76	0.8	29	165
86	DH-E111	70	45	44	68	23	94	0.4	25	165
87	DH-E113	70	65	38	94	29	58	0.3	27	110
88	DH-E114	65	65	39	93	28	63	0.6	21	138
89	DH-E115	70	64	42	94	30	112	0.8	30	129
90	DH-E117	70	54	44	78	24	72	0.3	24	151
91	DH-E118	70	56	44	86	30	76	0.7	26	184
92	DH-E119	60	51	21	81	30	95	0.5	41	227
93	DH-E120	65	74	53	100	26	88	0.4	33	158
94	DH-E121	70	74	52	98	24	53	0.6	28	210
95	DH-E122	70	64	30	93	29	46	0.5	32	162
96	DH-E123	75	68	40	96	28	53	0.6	36	145
CHK1	Chk 1 (WH147)	65	67	52	94	27	62	0.5	38	336
CHK2	Chk 2 (PBW175)	65	68	52	96	28	56	0.6	42	309
CHK3	Chk 3 (NI5439)	70	66	40	94	28	62	0.3	42	269
97	DH-E124	70	52	56	83	31	49	1.2	36	193
98	DH-E125	70	50	60	84	34	54	0.9	39	253
99	DH-E126	70	56	28	96	40	46	0.7	38	178

100	DH-E127	70	74	58	100	26	73	0.6	32	253
101	DH-E129	65	69	56	108	39	66	1.6	29	224
102	DH-E130	65	74	56	109	35	95	1.1	26	214
103	DH-E131	75	74	56	92	18	55	0.7	34	238
104	DH-E132	70	52	33	79	27	77	0.8	30	256
105	DH-E133	75	51	37	78	27	114	1.1	22	156
106	DH-E134	75	68	37	95	27	131	0.5	26	128
107	DH-E135	80	53	48	80	27	122	0.7	25	96
108	DH-E137	80	68	40	94	26	132	1.5	28	135
109	DH-E138	70	52	47	90	38	114	1.0	30	137
110	DH-E141	75	49	44	78	29	93	0.8	26	145
111	DH-E144	80	53	52	80	27	80	0.8	28	121
112	DH-E145	75	67	40	93	26	77	0.7	27	135
CHK1	Chk 1 (WH147)	75	59	55	85	26	147	0.8	25	212
CHK2	Chk 2 (PBW175)	75	62	73	86	24	140	1.0	32	154
CHK3	Chk 3 (NI5439)	75	62	54	94	32	120	0.8	34	77
113	DH-E146	80	51	52	76	25	125	0.7	24	156
114	DH-E147	75	52	52	76	24	139	0.7	28	143
115	DH-E148	85	52	49	78	26	151	0.6	24	120
116	DH-E149	80	57	45	86	29	80	0.6	28	176
117	DH-E150	80	52	43	79	27	111	0.8	24	154
118	DH-E152	75	52	50	78	26	110	0.8	26	159
119	DH-E153	85	55	52	79	24	178	0.4	22	137
120	DH-E154	80	56	51	83	27	117	0.5	22	130
121	DH-E155	80	52	54	78	26	159	0.7	25	150
122	DH-E156	80	52	46	76	24	110	0.5	22	100
123	DH-E158	85	51	46	76	25	104	0.7	20	119
124	DH-E159	80	40	53	70	30	112	0.8	31	186
125	DH-E161	80	43	54	70	27	120	0.9	23	194
126	DH-E162	85	70	54	98	28	55	0.6	25	58
127	DH-E164	85	50	49	77	27	128	0.7	22	144
128	DH-E165	80	46	48	76	30	112	0.7	21	151
CHK1	Chk 1 (WH147)	75	60	52	86	26	45	0.9	26	84
CHK2	Chk 2 (PBW175)	75	62	55	93	31	26	0.7	31	56
CHK3	Chk 3 (NI5439)	80	64	49	94	30	19	0.5	36	23
129	DH-E166	80	52	48	77	25	107	0.5	20	93
130	DH-E167	85	53	46	76	23	116	0.5	21	117
131	DH-E168	80	62	45	93	31	27	0.4	33	99
132	DH-E169	80	54	47	78	24	62	0.5	20	93
133	DH-E170	80	61	59	85	24	47	0.7	26	76
134	DH-E171	80	54	51	85	31	84	0.8	26	188
135	DH-E172	80	43	54	70	27	88	0.7	22	133
136	DH-E175	80	51	45	78	27	48	0.7	27	87
137	DH-E176	70	42	44	74	32	69	1.1	26	103
138	DH-E177	75	67	44	93	26	34	1.6	24	126
139	DH-E178	75	49	43	77	28	42	1.1	31	80
140	DH-E179	75	43	47	74	31	81	0.9	26	115
141	DH-E180	80	43	47	68	25	100	0.8	23	124
142	DH-E181	75	60	47	86	26	22	0.8	30	120
143	DH-E182	70	60	36	86	26	15	0.7	28	145

144	DH-E183	75	41	42	74	33	83	0.6	25	66
CHK1	Chk 1 (WH147)	75	63	51	94	31	28	0.4	36	25
CHK2	Chk 2 (PBW175)	75	62	40	92	30	25	0.4	29	25
CHK3	Chk 3 (NI5439)	75	49	45	76	27	86	0.4	28	87
145	DH-E184	75	45	45	74	29	98	0.4	26	69
146	DH-E185	75	49	47	76	27	108	0.7	27	95
147	DH-E186	80	56	46	83	27	45	0.9	27	61
148	DH-E187	75	52	37	78	26	32	0.5	26	34
149	DH-E188	75	55	42	80	25	26	0.6	26	30
150	DH-E189	75	51	42	80	29	50	1.1	25	30
151	DH-E190	75	55	41	86	31	88	0.6	26	156
152	DH-E191	70	51	35	77	26	35	0.4	25	140
153	DH-E192	75	51	47	76	25	51	0.5	25	155
154	DH-E193	75	43	43	74	31	41	0.4	25	166
155	DH-E194	75	50	49	76	26	20	0.8	27	49
156	DH-E195	75	61	47	93	32	130	0.9	27	137
157	DH-E196	70	64	52	93	29	63	0.5	30	80
158	DH-E197	80	41	43	93	52	87	0.7	26	140
159	DH-E198	75	44	48	74	30	52	0.9	26	89
160	DH-E199	70	50	45	80	30	30	0.7	27	49
CHK1	Chk 1 (WH147)	75	63	42	86	23	38	0.8	28	55
CHK2	Chk 2 (PBW175)	75	65	48	86	21	32	0.6	30	24
CHK3	Chk 3 (NI5439)	75	62	49	90	28	35	0.5	36	9
161	DH-E200	70	65	34	98	33	50	0.6	28	132
162	DH-E202	70	50	34	78	28	37	0.5	26	125
163	DH-E203	65	52	43	78	26	33	0.6	26	150
164	DH-E204	60	67	56	98	31	67	1.4	27	204
165	DH-E205	65	51	38	76	25	64	1.2	26	149
166	DH-E206	75	50	43	76	26	74	1.4	26	90
167	DH-E207	75	53	48	78	25	62	1.7	29	75
168	DH-E208	80	53	43	81	28	65	1.3	25	70
169	DH-E209	75	51	50	76	25	74	1.8	28	125
170	DH-E210	80	52	47	77	25	59	0.9	30	90
171	DH-E211	75	51	49	74	23	96	1.8	22	114
172	DH-E213	75	52	47	77	25	79	1.7	26	124
173	DH-E214	75	78	46	109	31	115	1.5	16	188
174	DH-E215	80	67	41	102	35	72	0.7	28	175
175	DH-E216	70	67	43	95	28	70	0.8	28	156
176	DH-E217	70	49	38	76	27	53	0.4	25	172
CHK1	Chk 1 (WH147)	75	57	62	84	27	70	0.6	25	124
CHK2	Chk 2 (PBW175)	75	60	58	84	24	88	1.1	30	108
CHK3	Chk 3 (NI5439)	80	61	55	92	31	108	1.1	36	93
177	DH-E218	70	55	45	83	28	33	0.6	25	142
178	DH-E219	80	66	43	94	28	22	0.5	30	246
179	DH-E220	80	65	42	93	28	29	0.6	24	134
180	DH-E221	80	66	43	102	36	61	1.1	29	169
181	DH-E222	75	57	45	83	26	79	0.8	23	78
182	DH-E223	80	47	44	76	29	107	0.5	24	142
183	DH-E224	75	47	44	74	27	96	0.8	25	66
184	DH-E226	75	61	61	86	25	30	0.8	21	87

185	DH-E227	75	49	50	76	27	76	1.2	26	72
186	DH-E228	75	62	50	86	24	55	0.6	34	41
187	DH-E229	75	52	46	78	26	90	1.0	27	97
188	DH-E230	75	51	53	76	25	93	0.8	24	90
189	DH-E231	80	39	44	67	28	86	0.8	29	151
190	DH-E232	75	64	49	89	25	53	1.0	33	50
191	DH-E233	75	41	59	78	37	100	1.0	28	135
192	DH-E233	75	61	47	90	29	53	0.8	32	84
**Location**	Pune									
**Crop-season**	2012-13									
**Condition**	Irrigated									
**S.No.**	**DH-2 Line code**	**GP***	**DTA**	**PH***	**DTM**	**GFD**	**PTPM**	**GWPE**	**TGW***	**GYPP**
CHK1	Chk 1 (WH147)	80	59	71	92	33	93	1.3	38	271
CHK2	Chk 2 (PBW175)	80	64	72	95	31	107	0.9	40	198
CHK3	Chk 3 (NI5439)	85	67	72	99	32	126	0.5	39	192
1	DH-E001	90	49	60	85	36	114	1.0	33	316
2	DH-E003	90	56	65	90	34	97	1.5	30	374
3	DH-E005	80	49	56	86	37	121	1.2	34	311
4	DH-E006	90	67	73	95	28	127	0.8	29	343
5	DH-E007	80	47	60	89	42	88	1.4	39	330
6	DH-E008	70	43	52	86	43	97	1.1	47	204
7	DH-E009	80	53	66	89	36	112	1.2	37	395
8	DH-E010	75	55	64	91	36	132	1.4	27	367
9	DH-E014	80	45	65	86	41	107	0.4	31	286
10	DH-E015	75	43	63	82	39	101	1.2	42	270
11	DH-E018	85	43	53	81	38	90	1.1	29	217
12	DH-E019	90	50	55	84	34	78	0.9	28	231
13	DH-E020	85	44	61	81	37	134	0.9	41	210
14	DH-E021	85	46	60	86	40	131	0.9	37	197
15	DH-E022	90	47	61	85	38	72	1.1	33	218
16	DH-E023	80	72	52	106	34	77	1.0	24	158
CHK1	Chk 1 (WH147)	80	64	62	97	33	136	1.0	30	165
CHK2	Chk 2 (PBW175)	70	70	71	98	28	85	1.0	30	134
CHK3	Chk 3 (NI5439)	80	67	69	103	36	115	0.4	34	110
17	DH-E024	80	43	53	86	43	120	1.1	38	241
18	DH-E025	80	43	59	81	38	102	1.3	38	241
19	DH-E026	80	72	62	105	33	161	0.2	26	243
20	DH-E029	80	67	61	97	30	137	0.5	25	160
21	DH-E032	75	52	54	90	38	125	0.8	33	246
22	DH-E033	65	46	63	83	37	92	1.1	41	236
23	DH-E034	80	44	57	82	38	59	1.4	46	221
24	DH-E036	85	47	63	88	41	120	1.4	34	290
25	DH-E038	75	52	51	88	36	141	1.2	28	179
26	DH-E039	90	49	61	85	36	131	1.3	31	218
27	DH-E040	85	53	50	91	38	98	1.1	29	195
28	DH-E041	75	53	57	85	32	149	1.2	29	227
29	DH-E042	75	56	54	89	33	154	1.0	30	180
30	DH-E043	80	49	61	84	35	81	0.4	31	198
31	DH-E044	75	71	58	101	30	98	0.8	26	139
32	DH-E046	75	53	58	89	36	76	0.9	28	149

CHK1	Chk 1 (WH147)	75	90	58	104	14	77	0.0	22	72
CHK2	Chk 2 (PBW175)	80	54	71	103	49	109	1.5	30	246
CHK3	Chk 3 (NI5439)	85	50	67	81	31	148	1.2	42	357
33	DH-E047	80	54	56	88	34	126	1.1	29	241
34	DH-E051	75	50	59	85	35	117	1.3	39	320
35	DH-E052	80	52	62	86	34	107	1.2	36	267
36	DH-E053	95	55	58	102	47	149	1.1	29	337
37	DH-E054	95	68	65	101	33	122	0.5	27	275
38	DH-E055	80	48	62	91	43	135	1.2	34	282
39	DH-E056	85	53	68	89	36	141	1.6	30	277
40	DH-E058	90	73	67	102	29	184	0.0	26	344
41	DH-E059	90	50	67	83	33	121	1.1	34	313
42	DH-E060	90	52	61	84	32	124	0.9	29	327
43	DH-E061	80	59	72	92	33	102	1.1	35	357
44	DH-E062	80	63	74	96	33	94	1.2	39	246
45	DH-E063	80	63	72	99	36	107	0.9	46	242
46	DH-E065	75	49	46	85	36	98	0.7	32	148
47	DH-E066	80	50	56	83	33	120	0.9	32	202
48	DH-E067	80	52	57	83	31	136	1.0	36	291
CHK1	Chk 1 (WH147)	85	60	62	92	32	173	1.4	34	305
CHK2	Chk 2 (PBW175)	80	64	76	96	32	106	1.5	35	251
CHK3	Chk 3 (NI5439)	80	65	73	97	32	84	0.8	42	191
49	DH-E068	75	42	54	107	65	93	1.1	42	143
50	DH-E069	80	50	66	81	31	114	1.2	36	141
51	DH-E070	80	60	50	92	32	126	1.0	28	123
52	DH-E071	90	43	42	82	39	120	1.0	33	108
53	DH-E072	85	49	61	85	36	106	1.6	32	152
54	DH-E073	80	50	59	83	33	112	1.0	34	131
55	DH-E074	80	50	58	84	34	94	1.4	33	222
56	DH-E075	85	61	55	94	33	161	1.0	42	194
57	DH-E076	80	51	60	86	35	133	1.1	32	344
58	DH-E077	80	42	55	84	42	107	0.9	39	246
59	DH-E078	75	42	56	84	42	95	1.1	37	219
60	DH-E080	80	53	56	86	33	122	1.0	32	256
61	DH-E081	75	54	59	88	34	95	1.2	30	293
62	DH-E082	80	52	66	88	36	133	1.2	33	420
63	DH-E083	80	52	65	98	46	97	1.1	34	318
64	DH-E084	85	68	59	102	34	135	0.9	32	218
CHK1	Chk 1 (WH147)	65	60	68	95	35	114	1.5	38	398
CHK2	Chk 2 (PBW175)	70	64	77	97	33	87	1.4	39	372
CHK3	Chk 3 (NI5439)	80	62	83	99	37	138	1.3	48	410
65	DH-E086	80	52	56	89	37	126	1.3	33	238
66	DH-E087	80	49	57	83	34	101	1.5	33	272
67	DH-E088	80	43	62	88	45	97	1.2	35	201
68	DH-E090	85	43	55	109	66	117	1.4	42	256
69	DH-E091	80	41	53	109	68	107	1.2	44	297
70	DH-E092	75	43	54	109	66	109	0.9	42	219
71	DH-E093	75	43	56	83	40	91	1.3	37	347
72	DH-E094	75	70	73	105	35	122	0.7	28	401
73	DH-E095	80	64	79	95	31	108	1.9	35	475

74	DH-E096	80	52	66	86	34	99	1.3	39	518
75	DH-E097	80	43	70	84	41	83	1.2	42	343
76	DH-E098	75	44	66	84	40	102	1.4	43	329
77	DH-E099	80	52	57	86	34	126	1.5	36	413
78	DH-E100	80	64	56	96	32	123	1.1	29	273
79	DH-E102	85	53	63	86	33	117	1.3	32	420
80	DH-E103	80	53	68	87	34	80	1.4	37	353
CHK1	Chk 1 (WH147)	75	64	66	95	31	157	1.0	34	343
CHK2	Chk 2 (PBW175)	75	67	70	97	30	105	1.1	34	203
CHK3	Chk 3 (NI5439)	80	68	73	105	37	91	1.0	38	163
81	DH-E104	80	54	67	88	34	103	1.5	32	404
82	DH-E105	85	45	58	86	41	96	1.5	33	360
83	DH-E108	80	52	58	88	36	113	1.4	37	393
84	DH-E109	75	46	52	88	42	119	1.0	30	293
85	DH-E110	80	57	59	89	32	108	1.2	37	383
86	DH-E111	75	45	69	85	40	97	1.1	40	348
87	DH-E113	80	65	66	96	31	117	0.7	44	241
88	DH-E114	85	71	63	103	32	134	0.4	22	224
89	DH-E115	75	68	68	99	31	96	0.6	33	248
90	DH-E117	80	52	60	88	36	123	1.0	37	257
91	DH-E118	80	55	63	96	41	119	1.0	32	248
92	DH-E119	80	44	57	83	39	83	1.2	41	300
93	DH-E120	75	71	66	104	33	99	0.5	26	238
94	DH-E121	80	67	66	95	28	129	0.5	30	325
95	DH-E122	75	57	61	91	34	123	1.6	41	378
96	DH-E123	80	68	70	98	30	109	0.8	30	397
CHK1	Chk 1 (WH147)	75	62	71	92	30	88	1.1	31	316
CHK2	Chk 2 (PBW175)	70	64	76	94	30	91	1.3	35	251
CHK3	Chk 3 (NI5439)	70	65	81	96	31	119	1.3	40	214
97	DH-E124	70	45	62	84	39	85	1.5	38	369
98	DH-E125	80	43	62	83	40	125	1.5	37	457
99	DH-E126	80	57	58	95	38	108	1.3	36	293
100	DH-E127	80	75	70	101	26	133	0.3	25	348
101	DH-E129	75	72	69	101	29	123	0.2	22	312
102	DH-E130	90	62	56	104	42	141	0.1	25	242
103	DH-E131	80	44	60	82	38	136	1.2	33	312
104	DH-E132	70	53	54	85	32	124	1.4	26	263
105	DH-E133	75	52	52	86	34	118	1.0	30	265
106	DH-E134	75	68	60	96	28	120	0.1	26	184
107	DH-E135	70	53	57	95	42	93	1.0	30	272
108	DH-E137	80	68	67	97	29	87	1.1	32	292
109	DH-E138	70	50	66	94	44	110	1.6	36	305
110	DH-E141	80	44	57	86	42	102	1.1	33	340
111	DH-E144	75	53	65	91	38	154	1.5	30	338
112	DH-E145	70	68	68	95	27	123	0.7	30	421
CHK1	Chk 1 (WH147)	75	60	80	95	35	195	1.7	33	556
CHK2	Chk 2 (PBW175)	75	63	92	94	31	147	1.0	34	391
CHK3	Chk 3 (NI5439)	70	62	90	102	40	117	1.1	40	362
113	DH-E146	75	52	69	84	32	124	1.4	31	502
114	DH-E147	80	54	73	86	32	109	1.5	34	460

115	DH-E148	80	54	66	88	34	107	1.8	32	388
116	DH-E149	80	64	70	97	33	128	1.2	39	446
117	DH-E150	75	54	66	86	32	119	1.6	32	486
118	DH-E152	80	53	69	86	33	106	1.2	34	455
119	DH-E153	80	53	65	86	33	118	0.9	30	482
120	DH-E154	75	55	65	93	38	164	1.7	29	375
121	DH-E155	80	54	65	90	36	144	1.4	34	353
122	DH-E156	75	53	56	90	37	111	1.1	33	284
123	DH-E158	80	52	62	89	37	98	1.6	30	372
124	DH-E159	80	43	53	82	39	124	1.2	44	273
125	DH-E161	75	45	64	83	38	118	1.5	36	403
126	DH-E162	80	72	78	103	31	129	0.7	26	329
127	DH-E164	85	51	63	86	35	124	1.3	31	408
128	DH-E165	85	49	65	86	37	151	1.5	33	428
CHK1	Chk 1 (WH147)	75	62	80	94	32	178	1.7	36	455
CHK2	Chk 2 (PBW175)	65	65	97	96	31	118	1.8	38	386
CHK3	Chk 3 (NI5439)	50	65	93	98	33	189	1.2	40	232
129	DH-E166	80	53	62	91	38	154	1.4	31	288
130	DH-E167	80	54	57	91	37	96	1.1	28	329
131	DH-E168	80	64	66	94	30	119	0.9	33	192
132	DH-E169	75	54	60	91	37	153	1.3	30	298
133	DH-E170	75	62	81	94	32	149	1.7	34	473
134	DH-E171	75	52	79	98	46	147	1.0	28	379
135	DH-E172	80	43	66	86	43	105	1.4	38	376
136	DH-E175	75	53	64	88	35	122	1.6	35	357
137	DH-E176	70	44	63	85	41	67	1.3	36	219
138	DH-E177	80	72	71	94	22	126	0.6	22	167
139	DH-E178	75	51	61	85	34	106	1.4	35	279
140	DH-E179	75	45	62	83	38	92	1.4	37	291
141	DH-E180	75	44	62	83	39	90	1.3	37	309
142	DH-E181	80	61	57	92	31	111	0.9	31	208
143	DH-E182	80	61	60	94	33	102	0.9	36	201
144	DH-E183	70	44	62	90	46	119	1.3	42	213
CHK1	Chk 1 (WH147)	70	66	84	98	32	134	0.6	40	222
CHK2	Chk 2 (PBW175)	80	69	74	96	27	134	0.2	33	228
CHK3	Chk 3 (NI5439)	75	52	63	85	33	123	1.0	35	303
145	DH-E184	70	47	56	85	38	75	1.2	36	233
146	DH-E185	75	50	69	89	39	108	1.9	34	248
147	DH-E186	75	54	65	90	36	122	0.6	27	153
148	DH-E187	75	53	61	87	34	129	1.1	30	263
149	DH-E188	75	53	61	92	39	157	1.0	30	268
150	DH-E189	80	54	65	90	36	163	0.8	29	279
151	DH-E190	75	59	61	94	35	158	0.9	28	273
152	DH-E191	75	43	56	86	43	129	1.2	35	159
153	DH-E192	75	52	60	86	34	125	1.2	28	215
154	DH-E193	65	44	53	83	39	114	1.4	38	202
155	DH-E194	70	50	58	87	37	103	1.5	33	190
156	DH-E195	70	60	76	95	35	135	1.1	32	272
157	DH-E196	70	65	72	94	29	118	0.4	34	186
158	DH-E197	80	44	60	83	39	129	1.3	36	323

159	DH-E198	80	47	71	83	36	127	1.9	37	303
160	DH-E199	80	50	63	83	33	138	1.6	32	280
CHK1	Chk 1 (WH147)	80	61	75	93	32	138	1.5	36	429
CHK2	Chk 2 (PBW175)	75	63	94	94	31	122	1.4	38	294
CHK3	Chk 3 (NI5439)	80	62	76	96	34	139	1.3	44	317
161	DH-E200	80	68	64	96	28	128	0.4	26	168
162	DH-E202	75	49	63	90	41	132	1.6	34	241
163	DH-E203	80	48	66	84	36	137	1.1	34	247
164	DH-E204	75	68	62	95	27	118	1.1	27	179
165	DH-E205	70	51	63	84	33	139	1.2	34	213
166	DH-E206	75	50	59	84	34	142	0.9	38	240
167	DH-E207	70	51	66	87	36	140	1.5	34	229
168	DH-E208	75	54	59	87	33	86	0.7	27	183
169	DH-E209	80	44	59	87	43	107	1.0	35	175
170	DH-E210	85	53	63	88	35	133	1.7	36	257
171	DH-E211	80	42	60	83	41	105	1.3	38	229
172	DH-E213	70	52	64	86	34	129	1.4	35	251
173	DH-E214	80	75	66	88	13	142	0.3	25	152
174	DH-E215	80	73	74	99	26	136	0.5	23	212
175	DH-E216	80	74	71	105	31	122	1.0	23	188
176	DH-E217	80	50	68	94	44	121	1.2	33	234
CHK1	Chk 1 (WH147)	80	60	67	95	35	134	1.1	34	298
CHK2	Chk 2 (PBW175)	80	62	78	95	33	98	1.9	39	250
CHK3	Chk 3 (NI5439)	80	65	80	97	32	86	1.0	45	233
177	DH-E218	75	53	73	95	42	113	0.9	30	278
178	DH-E219	80	70	76	105	35	141	0.9	24	340
179	DH-E220	75	65	67	105	40	142	1.4	30	345
180	DH-E221	75	74	73	88	14	151	0.5	26	285
181	DH-E222	70	56	75	95	39	132	0.9	30	254
182	DH-E223	80	47	58	84	37	96	0.3	35	245
183	DH-E224	75	49	59	84	35	113	1.4	39	252
184	DH-E226	85	62	75	94	32	137	1.5	27	189
185	DH-E227	70	51	60	84	33	106	1.1	35	215
186	DH-E228	70	65	61	93	28	83	1.3	38	144
187	DH-E229	75	52	61	85	33	87	1.3	33	216
188	DH-E230	75	53	56	86	33	92	1.3	34	227
189	DH-E231	75	38	46	80	42	90	0.8	41	166
190	DH-E232	80	65	63	94	29	101	0.9	40	268
191	DH-E233	70	50	64	86	36	97	1.5	35	245
192	DH-E233	75	65	62	95	30	123	1.6	40	220
**Location**	Hisar									
**Crop-season**	2012-13									
**Condition**	Rainfed									
**S.No.**	**DH-2 Line code**	**GP**	**DTA**	**PH***	**DTM**	**GFD**	**PTPM**	**GWPE**	**TGW***	**GYPP**
CHK1	Chk 1 (WH147)	NA	NA	100	NA	NA	72	2.1	25	161
CHK2	Chk 2 (PBW175)	NA	NA	105	NA	NA	96	2.0	29	170
CHK3	Chk 3 (NI5439)	NA	NA	85	NA	NA	82	3.1	19	279
1	DH-E001	NA	NA	89	NA	NA	87	1.9	44	302
2	DH-E003	NA	NA	95	NA	NA	91	1.2	23	291
3	DH-E005	NA	NA	81	NA	NA	86	1.0	25	254

4	DH-E006	NA	NA	87	NA	NA	91	0.9	24	193
5	DH-E007	NA	NA	91	NA	NA	89	1.8	27	272
6	DH-E008	NA	NA	89	NA	NA	72	1.7	29	179
7	DH-E009	NA	NA	80	NA	NA	86	2.1	18	242
8	DH-E010	NA	NA	97	NA	NA	91	2.0	29	210
9	DH-E014	NA	NA	94	NA	NA	67	1.8	29	223
10	DH-E015	NA	NA	98	NA	NA	82	0.5	19	151
11	DH-E018	NA	NA	97	NA	NA	76	1.0	33	243
12	DH-E019	NA	NA	82	NA	NA	85	1.7	17	155
13	DH-E020	NA	NA	91	NA	NA	91	1.1	34	168
14	DH-E021	NA	NA	95	NA	NA	76	2.2	47	195
15	DH-E022	NA	NA	74	NA	NA	68	2.3	21	140
16	DH-E023	NA	NA	99	NA	NA	97	1.4	14	160
CHK1	Chk 1 (WH147)	NA	NA	101	NA	NA	71	2.4	17	161
CHK2	Chk 2 (PBW175)	NA	NA	112	NA	NA	42	3.8	22	325
CHK3	Chk 3 (NI5439)	NA	NA	111	NA	NA	82	1.3	48	349
17	DH-E024	NA	NA	81	NA	NA	100	1.0	32	198
18	DH-E025	NA	NA	76	NA	NA	75	0.9	35	208
19	DH-E026	NA	NA	84	NA	NA	52	1.2	35	299
20	DH-E029	NA	NA	81	NA	NA	76	2.3	32	176
21	DH-E032	NA	NA	82	NA	NA	58	2.5	18	222
22	DH-E033	NA	NA	95	NA	NA	67	1.1	37	217
23	DH-E034	NA	NA	90	NA	NA	67	1.2	35	209
24	DH-E036	NA	NA	95	NA	NA	54	1.5	32	214
25	DH-E038	NA	NA	86	NA	NA	75	1.5	37	145
26	DH-E039	NA	NA	82	NA	NA	91	1.7	30	266
27	DH-E040	NA	NA	75	NA	NA	59	1.8	27	205
28	DH-E041	NA	NA	85	NA	NA	78	1.5	18	233
29	DH-E042	NA	NA	72	NA	NA	85	0.8	24	209
30	DH-E043	NA	NA	76	NA	NA	61	1.5	33	160
31	DH-E044	NA	NA	82	NA	NA	96	1.1	19	142
32	DH-E046	NA	NA	98	NA	NA	87	1.2	17	160
CHK1	Chk 1 (WH147)	NA	NA	76	NA	NA	74	2.1	27	172
CHK2	Chk 2 (PBW175)	NA	NA	92	NA	NA	69	1.7	28	191
CHK3	Chk 3 (NI5439)	NA	NA	79	NA	NA	58	2.4	18	232
33	DH-E047	NA	NA	75	NA	NA	101	1.8	19	182
34	DH-E051	NA	NA	76	NA	NA	58	1.2	26	185
35	DH-E052	NA	NA	75	NA	NA	55	2.2	24	227
36	DH-E053	NA	NA	79	NA	NA	76	1.9	37	172
37	DH-E054	NA	NA	84	NA	NA	63	2.1	13	179
38	DH-E055	NA	NA	81	NA	NA	59	1.7	42	166
39	DH-E056	NA	NA	84	NA	NA	67	2.1	35	172
40	DH-E058	NA	NA	71	NA	NA	99	1.8	35	221
41	DH-E059	NA	NA	81	NA	NA	91	1.6	28	158
42	DH-E060	NA	NA	99	NA	NA	89	1.5	27	209
43	DH-E061	NA	NA	100	NA	NA	75	1.0	25	180
44	DH-E062	NA	NA	95	NA	NA	69	1.8	37	308
45	DH-E063	NA	NA	101	NA	NA	89	2.1	36	353
46	DH-E065	NA	NA	78	NA	NA	69	1.1	24	186
47	DH-E066	NA	NA	82	NA	NA	75	2.1	36	171

48	DH-E067	NA	NA	96	NA	NA	84	1.3	35	160
CHK1	Chk 1 (WH147)	NA	NA	105	NA	NA	91	1.8	28	151
CHK2	Chk 2 (PBW175)	NA	NA	129	NA	NA	79	1.9	30	208
CHK3	Chk 3 (NI5439)	NA	NA	110	NA	NA	99	2.4	29	290
49	DH-E068	NA	NA	89	NA	NA	95	1.9	18	204
50	DH-E069	NA	NA	91	NA	NA	98	1.2	17	187
51	DH-E070	NA	NA	72	NA	NA	101	0.8	19	242
52	DH-E071	NA	NA	85	NA	NA	78	1.4	36	170
53	DH-E072	NA	NA	92	NA	NA	93	1.2	20	167
54	DH-E073	NA	NA	75	NA	NA	82	1.2	21	136
55	DH-E074	NA	NA	85	NA	NA	78	1.4	25	142
56	DH-E075	NA	NA	70	NA	NA	103	1.6	12	170
57	DH-E076	NA	NA	84	NA	NA	99	1.0	18	191
58	DH-E077	NA	NA	85	NA	NA	87	1.5	36	213
59	DH-E078	NA	NA	86	NA	NA	81	1.3	42	157
60	DH-E080	NA	NA	89	NA	NA	97	2.2	38	181
61	DH-E081	NA	NA	92	NA	NA	76	2.0	32	193
62	DH-E082	NA	NA	93	NA	NA	87	1.3	29	128
63	DH-E083	NA	NA	76	NA	NA	82	1.3	23	154
64	DH-E084	NA	NA	80	NA	NA	96	1.1	22	260
CHK1	Chk 1 (WH147)	NA	NA	110	NA	NA	87	1.0	22	227
CHK2	Chk 2 (PBW175)	NA	NA	121	NA	NA	68	1.2	21	231
CHK3	Chk 3 (NI5439)	NA	NA	112	NA	NA	96	1.2	31	257
65	DH-E086	NA	NA	91	NA	NA	91	1.3	26	162
66	DH-E087	NA	NA	85	NA	NA	79	1.4	20	179
67	DH-E088	NA	NA	86	NA	NA	87	1.7	25	207
68	DH-E090	NA	NA	86	NA	NA	59	2.1	38	207
69	DH-E091	NA	NA	91	NA	NA	49	1.0	26	225
70	DH-E092	NA	NA	91	NA	NA	85	1.1	27	181
71	DH-E093	NA	NA	87	NA	NA	79	1.1	36	145
72	DH-E094	NA	NA	81	NA	NA	58	0.6	27	194
73	DH-E095	NA	NA	85	NA	NA	107	1.2	34	301
74	DH-E096	NA	NA	95	NA	NA	89	0.5	38	247
75	DH-E097	NA	NA	94	NA	NA	78	0.9	21	171
76	DH-E098	NA	NA	84	NA	NA	67	1.1	35	196
77	DH-E099	NA	NA	81	NA	NA	76	0.6	21	204
78	DH-E100	NA	NA	67	NA	NA	110	1.7	32	170
79	DH-E102	NA	NA	80	NA	NA	86	1.6	21	186
80	DH-E103	NA	NA	90	NA	NA	101	1.5	16	200
CHK1	Chk 1 (WH147)	NA	NA	101	NA	NA	54	0.6	20	161
CHK2	Chk 2 (PBW175)	NA	NA	121	NA	NA	67	1.2	38	237
CHK3	Chk 3 (NI5439)	NA	NA	94	NA	NA	47	1.1	40	340
81	DH-E104	NA	NA	89	NA	NA	93	1.8	35	176
82	DH-E105	NA	NA	86	NA	NA	86	1.9	21	157
83	DH-E108	NA	NA	85	NA	NA	68	1.1	34	261
84	DH-E109	NA	NA	75	NA	NA	56	1.3	37	238
85	DH-E110	NA	NA	80	NA	NA	72	1.5	26	153
86	DH-E111	NA	NA	82	NA	NA	64	1.4	25	156
87	DH-E113	NA	NA	87	NA	NA	59	1.0	35	191
88	DH-E114	NA	NA	80	NA	NA	68	0.8	19	214

89	DH-E115	NA	NA	78	NA	NA	53	1.7	31	239
90	DH-E117	NA	NA	79	NA	NA	42	1.4	31	163
91	DH-E118	NA	NA	76	NA	NA	84	1.5	42	189
92	DH-E119	NA	NA	84	NA	NA	97	1.4	11	168
93	DH-E120	NA	NA	80	NA	NA	81	1.4	24	211
94	DH-E121	NA	NA	76	NA	NA	75	0.7	27	214
95	DH-E122	NA	NA	80	NA	NA	79	1.5	14	156
96	DH-E123	NA	NA	92	NA	NA	79	1.5	14	240
CHK1	Chk 1 (WH147)	NA	NA	80	NA	NA	79	0.8	24	223
CHK2	Chk 2 (PBW175)	NA	NA	108	NA	NA	38	1.3	34	312
CHK3	Chk 3 (NI5439)	NA	NA	115	NA	NA	62	1.5	37	351
97	DH-E124	NA	NA	90	NA	NA	64	1.5	12	181
98	DH-E125	NA	NA	84	NA	NA	78	1.3	37	247
99	DH-E126	NA	NA	78	NA	NA	96	1.7	25	154
100	DH-E127	NA	NA	82	NA	NA	87	0.6	26	246
101	DH-E129	NA	NA	80	NA	NA	79	0.6	32	207
102	DH-E130	NA	NA	61	NA	NA	107	1.8	27	166
103	DH-E131	NA	NA	75	NA	NA	65	1.6	26	222
104	DH-E132	NA	NA	76	NA	NA	85	1.4	20	164
105	DH-E133	NA	NA	85	NA	NA	78	0.5	17	232
106	DH-E134	NA	NA	75	NA	NA	56	1.5	27	150
107	DH-E135	NA	NA	80	NA	NA	87	2.0	22	171
108	DH-E137	NA	NA	79	NA	NA	71	0.8	28	198
109	DH-E138	NA	NA	85	NA	NA	49	1.5	28	191
110	DH-E141	NA	NA	84	NA	NA	85	1.3	30	210
111	DH-E144	NA	NA	85	NA	NA	71	0.6	19	211
112	DH-E145	NA	NA	82	NA	NA	90	0.7	25	166
CHK1	Chk 1 (WH147)	NA	NA	105	NA	NA	61	0.6	33	221
CHK2	Chk 2 (PBW175)	NA	NA	122	NA	NA	54	1.8	33	246
CHK3	Chk 3 (NI5439)	NA	NA	104	NA	NA	49	2.0	49	335
113	DH-E146	NA	NA	84	NA	NA	65	1.4	26	231
114	DH-E147	NA	NA	90	NA	NA	54	0.5	12	221
115	DH-E148	NA	NA	80	NA	NA	82	1.2	26	227
116	DH-E149	NA	NA	79	NA	NA	64	0.6	19	174
117	DH-E150	NA	NA	81	NA	NA	72	1.6	35	153
118	DH-E152	NA	NA	82	NA	NA	69	1.1	23	208
119	DH-E153	NA	NA	74	NA	NA	81	0.9	23	230
120	DH-E154	NA	NA	80	NA	NA	67	1.2	16	157
121	DH-E155	NA	NA	89	NA	NA	85	1.0	30	181
122	DH-E156	NA	NA	80	NA	NA	91	1.1	28	209
123	DH-E158	NA	NA	86	NA	NA	91	0.8	31	225
124	DH-E159	NA	NA	78	NA	NA	76	1.3	26	204
125	DH-E161	NA	NA	94	NA	NA	69	0.8	29	234
126	DH-E162	NA	NA	89	NA	NA	82	0.6	16	200
127	DH-E164	NA	NA	80	NA	NA	99	1.0	20	224
128	DH-E165	NA	NA	75	NA	NA	81	1.1	23	191
CHK1	Chk 1 (WH147)	NA	NA	115	NA	NA	48	1.0	21	177
CHK2	Chk 2 (PBW175)	NA	NA	132	NA	NA	52	1.0	38	299
CHK3	Chk 3 (NI5439)	NA	NA	115	NA	NA	61	1.1	23	301
129	DH-E166	NA	NA	82	NA	NA	72	0.4	13	225

130	DH-E167	NA	NA	83	NA	NA	61	1.1	30	163
131	DH-E168	NA	NA	84	NA	NA	59	0.6	17	173
132	DH-E169	NA	NA	95	NA	NA	82	0.5	13	188
133	DH-E170	NA	NA	82	NA	NA	68	1.3	27	159
134	DH-E171	NA	NA	112	NA	NA	75	0.4	9	200
135	DH-E172	NA	NA	102	NA	NA	59	0.9	16	181
136	DH-E175	NA	NA	78	NA	NA	72	0.9	15	219
137	DH-E176	NA	NA	81	NA	NA	56	0.8	24	193
138	DH-E177	NA	NA	80	NA	NA	75	0.6	16	147
139	DH-E178	NA	NA	85	NA	NA	69	0.6	18	225
140	DH-E179	NA	NA	93	NA	NA	76	1.3	25	214
141	DH-E180	NA	NA	90	NA	NA	69	1.3	31	244
142	DH-E181	NA	NA	75	NA	NA	89	0.9	25	225
143	DH-E182	NA	NA	83	NA	NA	68	0.9	30	194
144	DH-E183	NA	NA	80	NA	NA	51	0.8	35	177
CHK1	Chk 1 (WH147)	NA	NA	81	NA	NA	85	1.6	35	335
CHK2	Chk 2 (PBW175)	NA	NA	82	NA	NA	94	0.6	14	177
CHK3	Chk 3 (NI5439)	NA	NA	80	NA	NA	102	0.9	18	197
145	DH-E184	NA	NA	85	NA	NA	43	1.5	32	230
146	DH-E185	NA	NA	92	NA	NA	48	2.2	29	380
147	DH-E186	NA	NA	89	NA	NA	56	1.1	25	230
148	DH-E187	NA	NA	85	NA	NA	76	0.3	8	167
149	DH-E188	NA	NA	80	NA	NA	81	1.8	19	150
150	DH-E189	NA	NA	85	NA	NA	65	0.4	10	135
151	DH-E190	NA	NA	90	NA	NA	79	1.7	27	276
152	DH-E191	NA	NA	80	NA	NA	78	0.6	18	251
153	DH-E192	NA	NA	90	NA	NA	84	1.3	25	240
154	DH-E193	NA	NA	92	NA	NA	61	0.6	13	154
155	DH-E194	NA	NA	116	NA	NA	82	0.6	23	160
156	DH-E195	NA	NA	101	NA	NA	85	0.9	22	155
157	DH-E196	NA	NA	138	NA	NA	76	2.3	23	170
158	DH-E197	NA	NA	80	NA	NA	89	1.0	23	148
159	DH-E198	NA	NA	82	NA	NA	56	1.0	29	137
160	DH-E199	NA	NA	83	NA	NA	93	1.1	28	131
CHK1	Chk 1 (WH147)	NA	NA	96	NA	NA	59	1.0	29	182
CHK2	Chk 2 (PBW175)	NA	NA	115	NA	NA	92	1.9	25	288
CHK3	Chk 3 (NI5439)	NA	NA	95	NA	NA	74	1.2	25	275
161	DH-E200	NA	NA	75	NA	NA	72	1.5	23	119
162	DH-E202	NA	NA	70	NA	NA	87	0.9	26	150
163	DH-E203	NA	NA	80	NA	NA	69	1.5	30	167
164	DH-E204	NA	NA	65	NA	NA	75	0.4	14	198
165	DH-E205	NA	NA	73	NA	NA	65	0.8	19	185
166	DH-E206	NA	NA	80	NA	NA	58	1.2	32	170
167	DH-E207	NA	NA	80	NA	NA	98	1.6	32	267
168	DH-E208	NA	NA	65	NA	NA	96	1.3	28	144
169	DH-E209	NA	NA	75	NA	NA	71	1.1	35	181
170	DH-E210	NA	NA	75	NA	NA	79	1.3	24	161
171	DH-E211	NA	NA	80	NA	NA	57	0.7	24	128
172	DH-E213	NA	NA	80	NA	NA	73	1.1	28	145
173	DH-E214	NA	NA	72	NA	NA	92	1.5	24	137

174	DH-E215	NA	NA	60	NA	NA	88	1.8	19	199
175	DH-E216	NA	NA	72	NA	NA	69	0.7	33	167
176	DH-E217	NA	NA	70	NA	NA	86	1.4	23	143
CHK1	Chk 1 (WH147)	NA	NA	105	NA	NA	80	0.8	23	327
CHK2	Chk 2 (PBW175)	NA	NA	100	NA	NA	90	0.9	24	293
CHK3	Chk 3 (NI5439)	NA	NA	104	NA	NA	83	1.7	19	349
177	DH-E218	NA	NA	75	NA	NA	80	1.7	14	190
178	DH-E219	NA	NA	70	NA	NA	85	1.5	16	192
179	DH-E220	NA	NA	75	NA	NA	83	1.5	23	308
180	DH-E221	NA	NA	65	NA	NA	76	1.6	17	202
181	DH-E222	NA	NA	80	NA	NA	91	1.5	25	222
182	DH-E223	NA	NA	65	NA	NA	78	0.8	18	138
183	DH-E224	NA	NA	73	NA	NA	83	0.8	22	131
184	DH-E226	NA	NA	110	NA	NA	75	1.6	16	241
185	DH-E227	NA	NA	80	NA	NA	61	1.2	29	161
186	DH-E228	NA	NA	85	NA	NA	49	1.6	30	182
187	DH-E229	NA	NA	70	NA	NA	54	1.5	25	157
188	DH-E230	NA	NA	75	NA	NA	92	1.2	30	168
189	DH-E231	NA	NA	73	NA	NA	78	1.2	28	116
190	DH-E232	NA	NA	80	NA	NA	34	1.3	30	161
191	DH-E233	NA	NA	76	NA	NA	68	1.7	36	229
192	DH-E233	NA	NA	80	NA	NA	78	1.1	33	257
**Location**	Hisar									
**Crop-season**	2012-13									
**Condition**	Irrigated									
**S.No.**	**DH-2 Line code**	**GP**	**DTA**	**PH***	**DTM**	**GFD**	**PTPM**	**GWPE**	**TGW***	**GYPP**
CHK1	Chk 1 (WH147)	NA	NA	135	NA	NA	83	1.2	38	192
CHK2	Chk 2 (PBW175)	NA	NA	116	NA	NA	98	1.5	34	315
CHK3	Chk 3 (NI5439)	NA	NA	105	NA	NA	98	1.4	43	316
1	DH-E001	NA	NA	99	NA	NA	101	1.9	52	369
2	DH-E003	NA	NA	96	NA	NA	103	1.1	30	377
3	DH-E005	NA	NA	104	NA	NA	89	0.9	27	306
4	DH-E006	NA	NA	115	NA	NA	104	0.7	28	226
5	DH-E007	NA	NA	127	NA	NA	99	1.4	32	365
6	DH-E008	NA	NA	117	NA	NA	78	1.5	36	232
7	DH-E009	NA	NA	111	NA	NA	109	0.8	23	269
8	DH-E010	NA	NA	106	NA	NA	96	1.3	33	230
9	DH-E014	NA	NA	95	NA	NA	84	1.3	42	272
10	DH-E015	NA	NA	109	NA	NA	87	0.5	20	184
11	DH-E018	NA	NA	102	NA	NA	101	1.0	33	283
12	DH-E019	NA	NA	114	NA	NA	106	0.6	28	170
13	DH-E020	NA	NA	105	NA	NA	97	0.8	41	201
14	DH-E021	NA	NA	122	NA	NA	89	1.7	53	222
15	DH-E022	NA	NA	101	NA	NA	110	1.4	44	161
16	DH-E023	NA	NA	115	NA	NA	99	0.5	19	221
CHK1	Chk 1 (WH147)	NA	NA	109	NA	NA	96	0.8	24	261
CHK2	Chk 2 (PBW175)	NA	NA	135	NA	NA	61	1.6	38	352
CHK3	Chk 3 (NI5439)	NA	NA	121	NA	NA	101	1.3	54	365
17	DH-E024	NA	NA	95	NA	NA	105	1.0	35	304
18	DH-E025	NA	NA	91	NA	NA	101	0.9	35	241

19	DH-E026	NA	NA	86	NA	NA	92	1.1	47	331
20	DH-E029	NA	NA	84	NA	NA	87	2.1	38	202
21	DH-E032	NA	NA	85	NA	NA	107	0.8	35	247
22	DH-E033	NA	NA	97	NA	NA	75	1.0	38	299
23	DH-E034	NA	NA	118	NA	NA	71	1.1	42	253
24	DH-E036	NA	NA	96	NA	NA	84	1.0	33	278
25	DH-E038	NA	NA	96	NA	NA	107	1.9	59	238
26	DH-E039	NA	NA	97	NA	NA	98	1.4	39	284
27	DH-E040	NA	NA	102	NA	NA	103	1.2	34	226
28	DH-E041	NA	NA	105	NA	NA	111	1.0	27	314
29	DH-E042	NA	NA	95	NA	NA	101	0.7	26	268
30	DH-E043	NA	NA	120	NA	NA	132	1.2	42	177
31	DH-E044	NA	NA	105	NA	NA	121	0.8	34	175
32	DH-E046	NA	NA	125	NA	NA	105	0.6	22	199
CHK1	Chk 1 (WH147)	NA	NA	92	NA	NA	78	1.3	33	192
CHK2	Chk 2 (PBW175)	NA	NA	97	NA	NA	91	1.4	39	206
CHK3	Chk 3 (NI5439)	NA	NA	105	NA	NA	89	0.9	28	248
33	DH-E047	NA	NA	122	NA	NA	107	0.6	21	221
34	DH-E051	NA	NA	96	NA	NA	96	0.7	34	209
35	DH-E052	NA	NA	82	NA	NA	57	1.2	47	247
36	DH-E053	NA	NA	105	NA	NA	91	1.5	43	204
37	DH-E054	NA	NA	125	NA	NA	85	0.8	20	251
38	DH-E055	NA	NA	107	NA	NA	91	1.3	44	197
39	DH-E056	NA	NA	85	NA	NA	85	1.7	45	200
40	DH-E058	NA	NA	98	NA	NA	101	1.4	39	238
41	DH-E059	NA	NA	112	NA	NA	99	1.2	33	197
42	DH-E060	NA	NA	122	NA	NA	100	0.9	31	249
43	DH-E061	NA	NA	101	NA	NA	81	0.9	38	238
44	DH-E062	NA	NA	125	NA	NA	76	1.5	43	342
45	DH-E063	NA	NA	109	NA	NA	99	1.2	39	492
46	DH-E065	NA	NA	90	NA	NA	91	0.8	31	227
47	DH-E066	NA	NA	95	NA	NA	103	1.7	49	185
48	DH-E067	NA	NA	131	NA	NA	87	0.8	38	221
CHK1	Chk 1 (WH147)	NA	NA	111	NA	NA	99	1.1	33	181
CHK2	Chk 2 (PBW175)	NA	NA	131	NA	NA	96	1.5	37	337
CHK3	Chk 3 (NI5439)	NA	NA	116	NA	NA	127	1.6	45	413
49	DH-E068	NA	NA	109	NA	NA	97	0.9	27	236
50	DH-E069	NA	NA	103	NA	NA	106	0.7	19	214
51	DH-E070	NA	NA	102	NA	NA	109	0.6	26	263
52	DH-E071	NA	NA	110	NA	NA	99	1.3	42	249
53	DH-E072	NA	NA	96	NA	NA	96	0.8	22	214
54	DH-E073	NA	NA	90	NA	NA	89	0.7	23	165
55	DH-E074	NA	NA	95	NA	NA	102	0.9	30	185
56	DH-E075	NA	NA	77	NA	NA	117	0.5	16	226
57	DH-E076	NA	NA	90	NA	NA	104	0.7	21	227
58	DH-E077	NA	NA	111	NA	NA	91	1.3	48	251
59	DH-E078	NA	NA	91	NA	NA	102	1.3	48	180
60	DH-E080	NA	NA	93	NA	NA	101	1.7	49	444
61	DH-E081	NA	NA	94	NA	NA	79	1.4	42	248
62	DH-E082	NA	NA	95	NA	NA	121	0.9	30	195

63	DH-E083	NA	NA	96	NA	NA	91	0.7	34	225
64	DH-E084	NA	NA	81	NA	NA	100	0.9	32	293
CHK1	Chk 1 (WH147)	NA	NA	115	NA	NA	102	0.8	36	262
CHK2	Chk 2 (PBW175)	NA	NA	134	NA	NA	91	1.2	36	310
CHK3	Chk 3 (NI5439)	NA	NA	120	NA	NA	105	1.2	53	375
65	DH-E086	NA	NA	99	NA	NA	101	0.9	29	220
66	DH-E087	NA	NA	91	NA	NA	97	0.7	24	228
67	DH-E088	NA	NA	100	NA	NA	89	1.1	38	263
68	DH-E090	NA	NA	93	NA	NA	74	1.5	38	248
69	DH-E091	NA	NA	95	NA	NA	98	1.0	43	267
70	DH-E092	NA	NA	93	NA	NA	101	0.9	31	219
71	DH-E093	NA	NA	91	NA	NA	101	1.0	40	231
72	DH-E094	NA	NA	85	NA	NA	99	1.1	35	213
73	DH-E095	NA	NA	93	NA	NA	114	1.3	39	345
74	DH-E096	NA	NA	132	NA	NA	97	1.4	39	263
75	DH-E097	NA	NA	106	NA	NA	89	0.8	40	253
76	DH-E098	NA	NA	102	NA	NA	101	1.1	37	214
77	DH-E099	NA	NA	88	NA	NA	91	0.6	23	233
78	DH-E100	NA	NA	96	NA	NA	121	1.1	38	255
79	DH-E102	NA	NA	94	NA	NA	87	0.8	24	257
80	DH-E103	NA	NA	96	NA	NA	104	0.6	22	245
CHK1	Chk 1 (WH147)	NA	NA	110	NA	NA	71	0.4	33	207
CHK2	Chk 2 (PBW175)	NA	NA	129	NA	NA	72	1.5	N/A	290
CHK3	Chk 3 (NI5439)	NA	NA	107	NA	NA	63	1.1	44	418
81	DH-E104	NA	NA	95	NA	NA	99	1.3	39	284
82	DH-E105	NA	NA	94	NA	NA	109	0.7	21	213
83	DH-E108	NA	NA	96	NA	NA	98	1.0	38	325
84	DH-E109	NA	NA	91	NA	NA	102	1.3	43	254
85	DH-E110	NA	NA	92	NA	NA	95	1.0	28	193
86	DH-E111	NA	NA	96	NA	NA	87	0.8	40	217
87	DH-E113	NA	NA	108	NA	NA	62	0.8	46	263
88	DH-E114	NA	NA	85	NA	NA	75	0.6	21	275
89	DH-E115	NA	NA	86	NA	NA	79	0.9	37	256
90	DH-E117	NA	NA	85	NA	NA	85	0.9	32	193
91	DH-E118	NA	NA	87	NA	NA	92	1.4	48	212
92	DH-E119	NA	NA	87	NA	NA	97	0.4	12	240
93	DH-E120	NA	NA	85	NA	NA	103	1.1	29	251
94	DH-E121	NA	NA	87	NA	NA	110	0.1	N/A	249
95	DH-E122	NA	NA	82	NA	NA	85	0.5	15	216
96	DH-E123	NA	NA	95	NA	NA	121	0.3	14	271
CHK1	Chk 1 (WH147)	NA	NA	117	NA	NA	97	0.8	24	267
CHK2	Chk 2 (PBW175)	NA	NA	132	NA	NA	82	1.3	37	347
CHK3	Chk 3 (NI5439)	NA	NA	132	NA	NA	99	1.5	55	507
97	DH-E124	NA	NA	95	NA	NA	67	0.5	16	197
98	DH-E125	NA	NA	85	NA	NA	91	1.6	41	270
99	DH-E126	NA	NA	92	NA	NA	104	1.1	38	247
100	DH-E127	NA	NA	86	NA	NA	101	1.0	29	269
101	DH-E129	NA	NA	94	NA	NA	104	1.0	34	235
102	DH-E130	NA	NA	84	NA	NA	108	1.0	29	212
103	DH-E131	NA	NA	86	NA	NA	102	0.9	34	248

104	DH-E132	NA	NA	108	NA	NA	91	0.7	22	186
105	DH-E133	NA	NA	95	NA	NA	85	0.7	24	256
106	DH-E134	NA	NA	96	NA	NA	79	1.1	29	185
107	DH-E135	NA	NA	84	NA	NA	91	0.8	24	214
108	DH-E137	NA	NA	85	NA	NA	73	0.8	32	248
109	DH-E138	NA	NA	87	NA	NA	78	1.3	30	246
110	DH-E141	NA	NA	95	NA	NA	100	1.4	35	251
111	DH-E144	NA	NA	91	NA	NA	98	0.4	22	230
112	DH-E145	NA	NA	90	NA	NA	93	0.8	26	199
CHK1	Chk 1 (WH147)	NA	NA	116	NA	NA	79	1.0	43	253
CHK2	Chk 2 (PBW175)	NA	NA	129	NA	NA	58	1.8	38	337
CHK3	Chk 3 (NI5439)	NA	NA	109	NA	NA	67	2.0	53	381
113	DH-E146	NA	NA	92	NA	NA	101	1.2	37	308
114	DH-E147	NA	NA	92	NA	NA	92	0.4	12	282
115	DH-E148	NA	NA	86	NA	NA	86	1.2	34	268
116	DH-E149	NA	NA	89	NA	NA	95	0.6	34	381
117	DH-E150	NA	NA	85	NA	NA	90	1.1	38	177
118	DH-E152	NA	NA	84	NA	NA	78	0.8	38	264
119	DH-E153	NA	NA	76	NA	NA	85	0.8	28	256
120	DH-E154	NA	NA	91	NA	NA	75	0.8	23	207
121	DH-E155	NA	NA	94	NA	NA	92	0.8	30	292
122	DH-E156	NA	NA	87	NA	NA	101	1.1	36	257
123	DH-E158	NA	NA	92	NA	NA	105	1.2	38	293
124	DH-E159	NA	NA	86	NA	NA	90	1.0	33	266
125	DH-E161	NA	NA	96	NA	NA	93	1.1	30	248
126	DH-E162	NA	NA	91	NA	NA	102	0.8	37	316
127	DH-E164	NA	NA	82	NA	NA	98	0.8	21	253
128	DH-E165	NA	NA	79	NA	NA	103	0.9	24	231
CHK1	Chk 1 (WH147)	NA	NA	117	NA	NA	100	0.6	27	253
CHK2	Chk 2 (PBW175)	NA	NA	135	NA	NA	91	0.9	38	344
CHK3	Chk 3 (NI5439)	NA	NA	118	NA	NA	89	0.7	37	392
129	DH-E166	NA	NA	85	NA	NA	79	0.4	20	266
130	DH-E167	NA	NA	95	NA	NA	68	1.1	36	245
131	DH-E168	NA	NA	95	NA	NA	75	0.6	21	266
132	DH-E169	NA	NA	97	NA	NA	91	0.5	16	315
133	DH-E170	NA	NA	86	NA	NA	72	1.0	31	261
134	DH-E171	NA	NA	115	NA	NA	81	0.3	11	308
135	DH-E172	NA	NA	105	NA	NA	99	0.7	24	244
136	DH-E175	NA	NA	81	NA	NA	83	0.7	21	331
137	DH-E176	NA	NA	85	NA	NA	95	0.7	25	233
138	DH-E177	NA	NA	85	NA	NA	78	0.6	25	198
139	DH-E178	NA	NA	90	NA	NA	83	0.6	27	269
140	DH-E179	NA	NA	96	NA	NA	89	1.3	45	274
141	DH-E180	NA	NA	93	NA	NA	96	1.2	33	330
142	DH-E181	NA	NA	81	NA	NA	101	0.9	30	258
143	DH-E182	NA	NA	86	NA	NA	95	0.9	31	282
144	DH-E183	NA	NA	82	NA	NA	91	1.1	39	208
CHK1	Chk 1 (WH147)	NA	NA	98	NA	NA	87	1.6	45	408
CHK2	Chk 2 (PBW175)	NA	NA	85	NA	NA	102	0.6	21	192
CHK3	Chk 3 (NI5439)	NA	NA	83	NA	NA	138	0.9	22	253

145	DH-E184	NA	NA	91	NA	NA	83	1.1	43	243
146	DH-E185	NA	NA	95	NA	NA	79	1.6	35	402
147	DH-E186	NA	NA	90	NA	NA	85	1.1	30	266
148	DH-E187	NA	NA	105	NA	NA	78	0.3	14	287
149	DH-E188	NA	NA	84	NA	NA	91	0.7	27	187
150	DH-E189	NA	NA	87	NA	NA	85	0.4	14	179
151	DH-E190	NA	NA	92	NA	NA	92	1.4	31	361
152	DH-E191	NA	NA	85	NA	NA	114	0.6	21	270
153	DH-E192	NA	NA	95	NA	NA	87	1.0	28	275
154	DH-E193	NA	NA	93	NA	NA	87	0.5	19	183
155	DH-E194	NA	NA	120	NA	NA	90	0.6	32	192
156	DH-E195	NA	NA	105	NA	NA	126	0.7	23	169
157	DH-E196	NA	NA	140	NA	NA	98	1.3	41	355
158	DH-E197	NA	NA	84	NA	NA	93	0.8	27	165
159	DH-E198	NA	NA	85	NA	NA	85	0.9	34	173
160	DH-E199	NA	NA	87	NA	NA	96	1.2	35	148
CHK1	Chk 1 (WH147)	NA	NA	100	NA	NA	100	1.0	32	213
CHK2	Chk 2 (PBW175)	NA	NA	127	NA	NA	95	1.2	38	306
CHK3	Chk 3 (NI5439)	NA	NA	110	NA	NA	91	1.3	47	329
161	DH-E200	NA	NA	77	NA	NA	79	0.9	24	142
162	DH-E202	NA	NA	72	NA	NA	87	0.9	30	211
163	DH-E203	NA	NA	88	NA	NA	94	1.1	35	180
164	DH-E204	NA	NA	72	NA	NA	77	0.4	16	213
165	DH-E205	NA	NA	95	NA	NA	84	0.8	22	204
166	DH-E206	NA	NA	95	NA	NA	91	1.2	42	188
167	DH-E207	NA	NA	91	NA	NA	100	1.3	37	334
168	DH-E208	NA	NA	80	NA	NA	104	1.0	50	209
169	DH-E209	NA	NA	92	NA	NA	87	1.0	39	196
170	DH-E210	NA	NA	82	NA	NA	95	1.1	32	175
171	DH-E211	NA	NA	84	NA	NA	83	0.7	31	188
172	DH-E213	NA	NA	84	NA	NA	87	1.1	36	178
173	DH-E214	NA	NA	73	NA	NA	109	1.0	33	166
174	DH-E215	NA	NA	83	NA	NA	98	0.8	21	313
175	DH-E216	NA	NA	76	NA	NA	103	1.2	35	196
176	DH-E217	NA	NA	80	NA	NA	87	0.8	29	162
CHK1	Chk 1 (WH147)	NA	NA	128	NA	NA	81	0.8	29	449
CHK2	Chk 2 (PBW175)	NA	NA	125	NA	NA	96	0.9	29	449
CHK3	Chk 3 (NI5439)	NA	NA	115	NA	NA	95	0.9	26	454
177	DH-E218	NA	NA	82	NA	NA	102	0.4	20	175
178	DH-E219	NA	NA	77	NA	NA	89	0.5	20	273
179	DH-E220	NA	NA	83	NA	NA	93	0.8	23	344
180	DH-E221	NA	NA	75	NA	NA	97	0.8	29	239
181	DH-E222	NA	NA	88	NA	NA	104	1.1	33	249
182	DH-E223	NA	NA	76	NA	NA	97	0.6	22	220
183	DH-E224	NA	NA	90	NA	NA	94	0.8	27	172
184	DH-E226	NA	NA	118	NA	NA	92	0.5	21	322
185	DH-E227	NA	NA	90	NA	NA	87	1.2	34	217
186	DH-E228	NA	NA	85	NA	NA	96	1.2	48	274
187	DH-E229	NA	NA	85	NA	NA	101	0.9	30	192
188	DH-E230	NA	NA	88	NA	NA	94	1.1	33	212

189	DH-E231	NA	NA	75	NA	NA	81	0.9	32	254
190	DH-E232	NA	NA	93	NA	NA	78	1.0	40	265
191	DH-E233	NA	NA	87	NA	NA	85	1.7	40	307
192	DH-E233	NA	NA	103	NA	NA	113	1.1	43	285
