# Supplementary material for: QTL mapping for nine drought-responsive agronomic traits in bread wheat under irrigated and rain-fed environments
Source: PLoS One. 2017 Aug 9;12(8):e0182857. doi: 10.1371/journal.pone.0182857 (PMC5550002; doi:10.1371/journal.pone.0182857)
Supplement: S3 Table — (PDF) [file pone.0182857.s004.pdf]

S3 Table. Descriptive statistics (mean, SE, range, CV % and heritability; H^2) for nine (9) important agronomic traits measured on the Kukri/Excalibur DH mapping population in 22 different environments.

Trait/Env. ID	Mean±SE (Range)	CV (%)	H^{2*}	Trait/Env. ID	Mean±SE (Range)	CV (%)	H^2
**1. GP**				**4. GFD (cont.)**			
E01	55.32±0.33 (48.0-66.0)	08.38	-	E04	28.81±0.31 (21.0-53.0)	14.70	0.91
E02	47.50±0.25 (40.0-65.0)	07.24	0.58	E05	37.58±0.31 (24.0-56.0)	11.33	0.61
E03	90.03±0.35 (75.0-95.0)	05.41	0.23	E06	32.30±0.30 (22.0-49.0)	12.96	0.53
E04	93.78±0.22 (75.0-95.0)	03.26	0.25	E07	39.56±0.22 (20.0-48.0)	07.56	-
E05	84.64±0.60 (50.0-90.0)	09.85	0.15	E08	39.23±0.21 (18.0-52.0)	07.45	0.17
E06	84.17±0.68 (30.0-90.0)	11.25	0.28	E09	27.10±0.15 (23.0-35.0)	07.42	0.87
E07	76.49±0.18 (72.0-87.0)	03.29	-	E10	25.47±0.30 (14.0-57.0)	16.48	0.16
E08	74.23±0.20 (70.0-79.0)	03.77	0.16	E13	38.63±0.39 (21.0-53.0)	13.82	0.54
E09	85.34±0.41 (70.0-95.0)	06.62	0.12	E14	32.83±0.31 (23.0-48.0)	13.00	0.64
E10	87.21±0.38 (75.0-95.0)	06.09	0.78	E15	46.73±0.30 (36.0-63.0)	08.98	-
E11	82.45±0.73 (30.0-90.0)	12.30	0.29	E16	44.58±0.18 (38.0-54.0)	05.51	0.26
E12	82.86±0.59 (50.0-90.0)	09.79	0.45	E17	22.43±0.16 (14.0-32.0)	09.77	0.28
E13	78.47±0.57 (60.0-98.0)	10.04	0.18	E18	22.83±0.10 (18.0-27.0)	06.17	0.23
E14	69.97±0.80 (54.0-98.0)	15.78	0.44	E21	36.05±0.48 (13.0-68.0)	18.37	0.23
E15	78.01±0.19 (72.0-86.0)	03.30	0.23	E22	28.01±0.34 (16.0-54.0)	16.79	0.57
E16	72.26±0.26 (65.0-82.0)	04.92	0.34	**5. PH**			
E17	87.84±0.39 (75.0-95.0)	06.10	0.32	E01	71.21±0.46(60.0-107.0)	09.01	0.10
E18	86.38±0.41 (75.0-95.0)	06.52	0.31	E02	64.3±0.44(41.0-91.0)	09.41	0.18
E21	78.54±0.38 (65.0-95.0)	06.73	0.17	E03	87.3±0.66(72.0-142.0)	10.44	0.35
E22	74.82±0.40 (55.0-85.0)	07.38	0.30	E04	85.74±0.54(70.0-146.0)	08.80	0.69
**2. DTA**				E05	78.06±0.56(58.3-124.6)	09.91	0.53
E01	80.92±0.15 (76.0-86.0)	02.50	0.71	E06	58.58±0.43(40.6-95.0)	10.20	0.67
E02	76.67±0.26 (68.0-85.0)	04.65	0.81	E09	97.39±0.50(82.0-143.0)	07.09	0.66
E03	95.53±0.42 (85.0-108.0)	06.15	0.99	E10	83.61±0.60(62.3-122.6)	09.90	-
E04	94.81±0.40 (81.0-110.0)	05.85	0.79	E11	84.71±0.66(65.0-135.0)	10.84	0.72
E05	64.63±0.52 (51.0-84.0)	11.25	0.83	E12	63.87±0.64(44.0-98.0)	13.82	0.57
E06	61.02±0.52 (49.0-78.0)	11.72	0.63	E15	76.63±0.69(51.0-108.0)	12.56	0.51
E07	78.10±0.24 (66.0-89.0)	04.25	0.37	E16	61.27±0.73(41.0-100.0)	16.58	0.57
E08	75.90±0.24 (64.0-86.0)	04.45	0.19	E19	94.52±0.90(72.0-140.0)	13.24	0.61
E09	95.46±0.31 (85.0-101.0)	04.51	0.82	E20	83.45±0.70(60.0-138.0)	11.62	0.64
E10	93.45±0.42 (71.0-103.0)	06.23	0.28	E21	62.15±0.47(42.0-81.0)	10.55	0.53
E11	63.64±0.68 (49.0-94.0)	14.83	0.61	E22	45.84±0.45(21.0-61.0)	13.49	0.18
E14	59.02±0.52 (48.0-83.0)	12.26	0.54	**6. PTPM**			
E15	81.50±0.32 (68.0-95.0)	05.41	0.51	E01	170.28±2.31 (101.0-231.0)	18.82	0.78
E16	75.24±0.27 (62.0-90.0)	4.96	-	E02	158.76±2.08 (92.0-220.0)	18.11	0.71
E17	95.49±0.40 (84.0-105.0)	05.79	0.79	E03	146.4±2.03 (27.0-240.0)	19.21	0.10
E18	92.41±0.39 (83.0-102.0)	05.92	0.63	E04	135.15±2.28 (61.0-210.0)	23.41	0.46
E21	53.95±0.64 (38.0-75.0)	16.51	0.36	E05	135.02±2.00 (85.0-222.0)	20.50	-
E22	53.66±0.63 (38.0-78.0)	16.17	0.86	E06	71.9±1.33 (35.0-130.0)	25.67	-
**3. DTM**				E07	86.95±0.76 (70.0-122.0)	12.04	0.38
E01	115.3±0.16 (111.0-120.0)	01.93	0.71	E08	80.46±0.78 (63.0-118.0)	13.52	0.21
E02	108.61±0.18 (103.0-116.0)	02.35	0.81	E09	144.4±2.89 (12.0-248.0)	27.76	0.38
E03	124.92±0.19 (121.0-134.0)	02.07	0.73	E10	107.25±2.38 (11.0-205.0)	30.73	-
E04	123.62±0.20 (121.0-134.0)	02.22	0.69	E11	90.4±1.32 (42.0-145.0)	20.3	-
E05	102.21±0.49 (92.0-120.0)	06.70	0.72	E12	78.9±1.45 (36.0-140.0)	25.54	0.47
E06	93.31±0.46 (82.0-114.0)	06.83	0.83	E15	429.8±2.6 (320.0-536.0)	08.37	0.15
E07	117.70±0.21 (100.0-124.0)	02.47	0.10	E16	356.4±2.09 (278.0-406.0)	08.14	0.16
E08	115.14±0.22 (96.0-122.0)	02.66	0.19	E17	163.85±2.34 (26.0-275.0)	19.76	-
E09	122.57±0.27 (109.0-129.0)	03.01	0.71	E18	179.96±2.89 (96.0-270.0)	22.22	0.14
E10	118.92±0.41 (103.0-128.0)	04.80	0.85	E19	93.92±0.85 (57.0-132.0)	12.59	-
E11	102.27±0.53 (88.0-118.0)	07.13	0.54	E20	76.84±1.03 (34.0-110.0)	18.66	0.16
E12	91.85±0.53 (79.0-120.0)	08.05	0.75	E21	116.61±1.54 (59.0-184.0)	18.24	0.28
E13	128.05±0.21 (122.0-135.0)	02.22	0.38	E22	81.51±2.10 (15.0-178.0)	35.76	0.39
E14	122.77±0.17 (116.0-127.0)	01.96	0.21	**7. GWPE**			
E15	128.23±0.41 (111.0-143.0)	04.38	0.11	E03	1.53±0.03 (0.7-2.9)	22.79	0.36
E16	119.82±0.33 (106.0-131.0)	03.79	0.24	E04	1.53±0.03 (0.7-2.9)	23.01	0.37
E17	117.93±0.50 (106.0-128.0)	05.86	0.67	E05	1.5±0.02 (0.7-2.8)	21.18	0.49
E18	115.24±0.44 (106.0-127.0)	05.34	0.60	E06	1.22±0.02 (0.3-2.4)	23.73	0.45
E21	90.01±0.49 (80.0-109.0)	07.50	0.43	E11	1.42±0.02 (0.7-2.2)	20.16	0.14
E22	81.67±0.68 (65.0-110.0)	11.56	0.62	E12	1.18±0.02 (0.7-2.2)	21.28	0.24
**4. GFD**				E13	1.84±0.03 (1.0-2.8)	19.23	0.11
E01	34.38±0.10 (31.0-40.0)	04.08	0.45	E14	1.42±0.02 (0.7-2.3)	20.24	0.12
E02	31.95±0.21 (21.0-41.0)	09.32	0.75	E19	0.95±0.02 (0.1-2.1)	35.66	0.20
E03	29.39±0.32 (16.0-38.0)	15.21	0.90	E20	1.27±0.03 (0.29-2.45)	36.40	0.19

**7. GWPE (cont.)**				**8. TGW (contd)**			
E21	1.11±0.03 (0.0-1.9)	33.04	-	E22	26.66±0.28 (16.0-41.0)	14.36	-
E22	0.77±0.28 (0.3-1.8)	36.40	0.65	**9. GYPP**			
**8. TGW**				E01	249.64±3.43 (132.0-345.0)	19.04	0.65
E03	31.17±0.37 (16.3-49.7)	16.60	0.28	E02	166.31±2.15 (101.0-260.0)	17.95	0.76
E04	31.21±0.38 (16.3-49.7)	17.00	0.28	E03	411.4±6.06 (195.0-642.0)	20.41	0.22
E05	31.65±0.31 (22.0-47.6)	13.50	0.11	E04	212.96±4.67 (92.0-516.0)	30.41	0.37
E06	34.02±0.38 (13.6-50.8)	15.61	0.25	E05	461.17±7.24 (175.0-880.0)	21.76	0.33
E07	39.05±0.17 (36.0-47.0)	05.93	0.28	E06	225.27±4.03 (102.0-365.0)	24.77	0.44
E08	37.71±0.17 (35.0-45.0)	06.34	0.28	E11	512.02±7.63 (186.0-836.0)	20.64	-
E11	34.24±0.31 (20.8-51.6)	12.67	0.70	E12	237.49±4.67 (80.0-432.0)	27.27	0.53
E12	33.55±0.36 (22.4-46.0)	15.01	0.73	E13	305.54±3.06 (200.0-382.9)	13.87	0.15
E13	35.07±0.49 (18.2-49.6)	19.33	0.11	E14	205.26±2.66 (125.9-280.5)	17.96	0.21
E14	25.25±0.38 (12.8-39.8)	20.75	0.25	E15	390.16±3.13 (235.0-500.0)	11.11	0.43
E15	39.46±0.16 (32.0-46.0)	05.46	0.15	E16	331.61±2.07 (256.0-384.0)	08.65	0.18
E16	35.79±0.28 (30.0-56.0)	10.92	0.17	E17	289.68±7.70 (25.0-594.0)	36.80	0.37
E17	29.64±0.33 (17.6-48.8)	15.32	0.12	E18	228.32±5.94 (37.0-500.0)	36.03	0.12
E18	28.69±0.34 (17.6-48.8)	16.35	0.21	E19	245.38±3.98 (142.0-492.0)	22.45	0.12
E19	31.86±0.66 (11.3-58.8)	28.79	0.16	E20	194.83±3.13 (116.0-380.0)	22.29	-
E20	25.68±0.55 (08.2-47.0)	29.67	0.16	E21	277.86±6.16 (108.0-518.0)	30.72	-
E21	33.16±0.37 (21.6-46.8)	15.56	0.69	E22	127.8±3.35 (29.0-256.0)	36.30	0.51

For environments codes, refer Table 1; GP, germination percentage; DTA, days to anthesis; DTM, days to maturity; GFD, grain filling duration; PH, plant height; GWPE, grain weight/ear; PTPM, productive tillers/m^2; TGW, 1000 grain weight; GYPP, grain yield /plot. *Heritability could not be estimated due to very low genetic variance in some environments for some traits and indicated by "-"
