# Supplementary material for: QTL mapping for nine drought-responsive agronomic traits in bread wheat under irrigated and rain-fed environments
Source: PLoS One. 2017 Aug 9;12(8):e0182857. doi: 10.1371/journal.pone.0182857 (PMC5550002; doi:10.1371/journal.pone.0182857)
Supplement: S4 Table — (PDF) [file pone.0182857.s005.pdf]

S4 Table. Analysis of variance (ANOVA) for nine agronomic traits measured on the Kukri/Excalibur DH mapping population in 22 different environments.

Trait	S-Var	d.f.	E01	E02	E03	E04	E05	E06	E07	E08	E09	E10	E11
GP	Block	11	23.7	20.5**	15.9	20.3	68.9	82.0	7.7	10.1	95.2**	66.8***	82.3
	T	194	26.2	15.8**	23.3	10.2	78.9	98.4	6.6	8.1	27.2	29.9***	64.4
	Error	22	27.4	6.7	18.0	15.5	96.0	70.5	6.8	6.8	23.8	6.6	45.9
DTA	Block	11	2.4*	34.6***	1.0***	3.7	8.2	2.1	14.5*	10.7	5.9	22.9	-
	T	194	3.9***	8.8***	32.8***	29.2***	53.7***	56.8***	10.3*	10.0	19.8***	28.8	-
	Error	22	1.2	1.7	.2	6.1	9.1	20.8	6.5	8.1	3.6	20.6	-
DTM	Block	11	6.2***	5.9***	4.4**	3.5	21.5	6.9	13.7	17.7*	9.2*	11.7**	7.7
	T	194	4.8***	5.9***	5.9***	6.7***	46.4***	40.1***	8.7	10.1	14.5***	31.1***	8.2*
	Error	22	1.4	1.1	1.6	2.1	13.2	6.7	7.9	8.2	4.2	4.6	5.1
GFD	Block	11	4.1***	20.0***	5.2**	3.5**	9.1	9.5	34.1***	33.0**	2.5***	36.8	-
	T	194	1.8**	5.9***	19.1***	16.9***	16.3***	18.6**	8.4	8.5	3.5***	16.3	-
	Error	22	1.0	1.5	2.0	1.5	6.4	8.7	8.6	10.7	0.5	20.2	-
PH	Block	11	202.3	176.6	72.5	315.2***	109.7	153.7***	-	-	98.5***	535.9***	-
	T	194	115.4	85.4	135.2	193.2***	144.0**	101.3***	-	-	66.4***	136.9	-
	Error	22	129.0	110.3	87.9	59.3	68.1	33.9	-	-	22.6	136.4	-
PTPM	Block	11	740.6**	809.0**	1095.3	2643.8***	744.4	509.3	475.9***	610.1***	3735.2***	1821.6*	-
	T	194	1235.5***	1073.1***	785.9	653.4**	655.8	315.7	157.8*	167.7	1561.3*	878.1	-
	Error	22	271.7	312.7	708.7	355.2	641.8	287.4	98.1	131.7	960.3	917.5	-
GWPE	Block	11	-	-	0.2	0.1	0.1	0.1*	-	-	-	-	0.1
	T	194	-	-	0.1	0.1*	0.1**	0.1**	-	-	-	-	0.1
	Error	22	-	-	0.1	0.1	0.1	0.0	-	-	-	-	0.1
TGW	Block	11	-	-	53.0	44.7	8.9	18.4**	15.8***	16.7**	-	-	33.4
	T	194	-	-	42.1	42.5	22.4**	29.3***	6.2	6.4	-	-	44.9
	Error	22	-	-	50.8	50.3	9.8	7.3	4.5	4.7	-	-	40.1
GYPP	Block	11	1093.7	975.4***	9035.6	5389.9**	17090.8**	20030.5***	-	-	-	-	2839.4*
	T	194	2799.2***	1203.8***	8177.9	3679.7*	10010.3	3962.0*	-	-	-	-	1632.3
	Error	22	974.9	291.4	11269.6	2327.1	6706.9	2222.2	-	-	-	-	1393.5

Trait	S-Var	d.f.	E12	E13	E14	E15	E16	E17	E18	E19	E20	E21	E22
GP	Block	11	147.9*	154.7	37.6	8.8	24.9***	77.0***	52.2**	-	-	84.1**	209.6***
	T	194	122.8*	85.2	58.3*	6.6	7.7	29.5	33.3	-	-	24.8	30.6
	Error	22	67.7	109.7	32.9	9.5	5.1	20.0	23.0	-	-	31.3	54.3
DTA	Block	11	-	15.7	3.9	64.3***	53.8***	13.2*	20.9*	-	-	8.9	25.5**
	T	194	-	89.0***	51.8**	19.8**	16.6	29.9***	28.5***	-	-	87.4	76.1***
	Error	22	-	34.5	23.7	9.7	15.1	6.1	10.5	-	-	55.8	11.0
DTM	Block	11	6.9	12.5	25.1*	99.5***	51.2	29.2*	23.4	-	-	10.7	19.2
	T	194	6.0	52.6**	53.7***	28.9	23.4	45.4***	36.5***	-	-	48.6*	84.4***
	Error	22	4.8	24.0	13.6	25.7	34.4	14.8	14.5	-	-	27.7	31.9
GFD	Block	11	-	14.1	13.8*	59.6***	23.1*	4.0	1.2	-	-	6.9	14.4
	T	194	-	28.1**	17.8**	15.1	7.1	4.3	1.9	-	-	41.7	21.9*
	Error	22	-	12.9	6.3	15.9	10.9	3.1	1.5	-	-	32.2	9.4
PH	Block	11	-	235.6***	290.4***	222.4***	363.7***	-	-	383.5***	440.8***	173.9***	84.9
	T	194	-	177.3***	132.6**	97.3**	131.1**	-	-	207.9***	165.6***	71.5**	40.3
	Error	22	-	49.2	56.9	48.1	56.6	-	-	81.4	58.8	33.5	51.7
PTPM	Block	11	-	1071.5***	643.3**	1826.6**	2163.8***	2145.6*	2069.9	482.3***	634.3***	1087.2	2600.7***
	T	194	-	319.9	410.5**	800.0	605.8	1051.5	1460.6	150.3	200.5	465.8	649.6*
	Error	22	-	297.2	216.2	680.4	509.9	1017.1	1256.7	146.9	169.3	752.8	397.3
GWPE	Block	11	0.1	0.0	0.0	-	-	-	-	0.1	0.9***	0.2	0.1***
	T	194	0.1	0.1	0.1	-	-	-	-	0.1	0.2	0.1	0.1***
	Error	22	0.1	0.1	0.0	-	-	-	-	0.1	0.3	0.1	0.0
TGW	Block	11	31.9	27.8***	17.3**	8.4*	17.2	18.2	43.9	69.7	68.7	28.6**	29.9
	T	194	27.3	21.6***	22.2***	4.6	9.8	34.9	38.3	84.5	57.7	28.5***	17.7
	Error	22	41.3	6.4	6.0	3.9	12.4	40.6	30.2	71.0	71.2	10.3	16.2
GYPP	Block	11	2449.9***	25509.7**	11872.3***	3105.8***	2173.5***	7329.8	20610.1*	9802.6**	4058.8	21669.6***	19205.5***
	T	194	530.8	8695.6	4433.0**	1005.8*	622.7	17142.9	9238.3	4336.9	2689.3	4960.7	3646.1**
	Error	22	418.6	9388.2	2083.3	572.0	510.7	10798.4	10689.9	3823.5	2734.3	5233.0	1794.4

Foot note: For environments codes, refer Table 1; *, **, *** represent significance 0.1, 0.05, 0.01 levels respectively; "-" represents trait data unavailable; S-Var, Source of variation; T, Treatment.
