# Supplementary material for: QTL mapping for nine drought-responsive agronomic traits in bread wheat under irrigated and rain-fed environments
Source: PLoS One. 2017 Aug 9;12(8):e0182857. doi: 10.1371/journal.pone.0182857 (PMC5550002; doi:10.1371/journal.pone.0182857)
Supplement: S5 Table — (PDF) [file pone.0182857.s006.pdf]

S5 Table. Descriptive statistics (mean, SE, and range) for DSI of nine (9) important agronomic traits measured on the Kukri/Excalibur DH mapping population in 12 different environments.

Trait/Env. ID	Mean±SE	Range	Trait/Env. ID	Mean±SE	Range
**1. GP**			**5. PH (contd)**		
LY1	0.989±0.034	-0.54 to 2.45	LY4	0.991±0.018	0.45 to 1.78
LY2	1.036±0.112	-2.88 to 5.97	LY6	0.685±0.054	-1.35 to 4.24
LY4	0.272±1.802	-113.51 to 140.12	LY8	1.001±0.029	-0.05 to 2.01
LY5	1.001±0.048	-2.28 to 4.74	LY9	1.02±0.037	-0.45 to 2.42
LY6	1.101±0.320	-9.73 to 16.74	LY10	0.337±1.277	-144.73 to 30.95
LY7	1.029±0.043	-0.27 to 2.20	LY11	0.953±0.039	-0.39 to 2.23
LY8	2.513±1.889	-60.68 to 172.47	LY12	0.956±0.063	-1.45 to 3.25
LY9	0.991±0.055	-1.23 to 3.18	**6. PTPM**		
LY10	2.959±0.273	0.00 to 9.00	LY1	0.862±0.149	-13.15 to 7.28
LY11	0.943±0.210	-8.45 to 7.49	LY2	0.475±0.306	-20.51 to 7.42
**2.DTA**			LY4	0.974±0.022	-0.21 to 1.68
LY1	0.999±0.052	-1.25 to 3.13	LY5	1.037±0.036	-0.14 to 2.78
LY2	0.874±0.372	-10.84 to 34.08	LY6	1.051±0.887	-47.29 to 153.11
LY4	0.989±0.065	-2.47 to 3.77	LY8	0.596±0.214	-12.78 to 5.74
LY5	0.996±0.032	-0.45 to 2.23	LY9	0.992±0.033	-0.52 to 2.44
LY6	0.986±0.152	-2.77 to 12.2	LY10	1.012±0.709	-116.69 to 26.46
LY8	0.908±0.078	-5.36 to 3.8	LY11	0.779±0.116	-10.55 to 3.17
LY9	0.986±0.047	-1.21 to 2.59	LY12	0.965±0.071	-1.36 to 4.01
LY10	1.160±0.099	0.00 to 5.00	**7. GWPE**		
LY11	2.520±0.199	1.00 to 9.00	LY4	0.894±0.082	-2.49 to 3.98
**2. DTM**			LY7	0.939±0.055	-1.10 to 2.95
LY1	0.999±0.020	0.15 to 1.92	LY8	0.797±0.112	-10.05 to 2.96
LY2	1.000±0.043	0.00 to 2.92	LY11	-3.327±2.369	-386.09 to 2.74
LY4	0.989±0.037	-0.75 to 2.06	LY12	1.355±0.321	-35.63 to 19.69
LY5	1.001±0.048	-3.54 to 3.28	**8. TGW**		
LY6	0.992±0.159	-4.9 to 25.04	LY4	1.069±0.137	-6.88 to 5.72
LY7	0.999±0.013	0.69 to 1.58	LY5	0.998±0.045	0.06 to 6.90
LY8	0.992±0.040	-1.24 to 3.04	LY7	0.971±0.034	-0.49 to 2.20
LY9	0.989±0.033	-0.55 to 2.14	LY8	0.487±0.586	-47.33 to 13.39
LY10	0.890±0.205	-8.09 to 7.23	LY9	0.987±0.034	-0.07 to 3.86
LY11	0.999±0.058	-2.46 to 4.29	LY10	0.823±0.374	-16.94 to 13.82
**4. GFD**			LY11	0.904±0.067	-2.20 to 2.99
LY1	1.078±0.093	-2.97 to 5.43	LY12	0.786±0.162	-20.58 to 7.21
LY2	0.675±0.595	-48.5 to 13.99	**9. GYPP**		
LY4	1.043±0.060	-2.03 to 2.94	LY1	0.977±0.02	-0.62 to 1.83
LY5	0.954±0.480	-36.1 to 23.11	LY2	0.952±0.032	-0.77 to 1.66
LY6	0.836±0.357	-24.6 to 14.26	LY4	0.958±0.024	-1.02 to 1.54
LY8	0.901±0.072	-3.24 to 3.30	LY7	0.958±0.029	-0.47 to 1.82
LY9	0.808±0.165	-9.81 to 7.10	LY8	0.965±0.027	-0.42 to 1.85
LY10	-0.773±1.596	-118.16 to 75.27	LY9	0.976±0.044	-0.85 to 3.02
LY11	0.88±0.081	-7.45 to 2.60	LY10	1.580±0.121	0.00 to 5.00
**5. PH**			LY11	0.645±0.065	0.00 to 8.00
LY1	0.997±0.028	-0.41 to 4.02	LY12	0.859±0.077	-9.33 to 3.07
LY2	1.113±0.340	-12.67 to 28.42			

GP, germination percentage; DTA, days to anthesis; DTM, days to maturity; GFD, grain filling duration; PH, plant height; GWPE, grain weight/ear; PTPM, productive tillers/m^2; TGW, 1000 grain weight; GYPP, grain yield /plot; Location codes; LY1, Kanpur in year 2010-11; LY2, Karnal in year 2010-11; LY3, Hisar in year 2010-11; LY4, Pune in year 2010-11; LY5, Kanpur in year 2011-12; LY6, Karnal in year 2011-12; LY7, Hisar in year 2011-12; LY8, Pune in year 2011-12; LY9, Kanpur in year 2012-13; LY10, Karnal in year 2012-13; LY11, Hisar in year 2012-13; LY12, Pune in year 2012-13.
