# Supplementary material for: QTL mapping for nine drought-responsive agronomic traits in bread wheat under irrigated and rain-fed environments
Source: PLoS One. 2017 Aug 9;12(8):e0182857. doi: 10.1371/journal.pone.0182857 (PMC5550002; doi:10.1371/journal.pone.0182857)
Supplement: S6 Table — (PDF) [file pone.0182857.s007.pdf]

S6 Table. Number of markers per chromosome and the length of individual linkage groups of Kukri × Excalibur DH mapping population.

**Homeologous group**	**Total markers (length in cM)**			
	Sub-genome A	Sub-genome B	Sub-genome D	Total
**1**	28 (91.0)	18 (67.4)	13 (70.8)	59 (229.2)
**2**	15 (79.8)	29 (85.5)	11 (52.6)	55 (217.9)
**3**	17 (95.5)	24 (83.1)	07 (71.6)	48 (250.2)
**4**	28 (74.2)	11 (41.7)	04 (20.2)	43 (136.1)
**5**	17 (84.6)	22 (68.8)	09 (76.8)	48 (230.2)
**6**	25 (62.8)	25 (90.0)	13 (96.8)	63 (249.7)
**7**	38 (105.2)	25 (72.4)	13 (107.9)	76 (285.5)
**Total**	**168 (593.1)**	**154 (508.9)**	**70 (496.7)**	**392 (1598.7)**
