# Supplementary material for: QTL mapping for nine drought-responsive agronomic traits in bread wheat under irrigated and rain-fed environments
Source: PLoS One. 2017 Aug 9;12(8):e0182857. doi: 10.1371/journal.pone.0182857 (PMC5550002; doi:10.1371/journal.pone.0182857)
Supplement: S7 Table — (PDF) [file pone.0182857.s008.pdf]

S7 Table. List of major QTL (PVE> 20%) for seven drought-responsive traits identified in earlier studies along with their linked markers, PVE, mapping population type, and parental genotypes.

S.No.	Trait/QTL	Linked marker	PVE (R^2)[a]	Mapping population; parental genotypes	Favourable allele	Reference
**1. Grain yield**						
a)	*qGYWD.3B.2*	*Xgpw7774*	19.6	RIL; WL711(DS)/C306 (DT)	C306	[1]
b)	*4A*	*Xwmc420*	20.0	RIL; Sitta(DS)/Dharwar Dry (DT)	Dharwar Dry	[2]
c)	*4A-a*	*Xgwm397*	23.9	RIL; SeriM82/Babax	Babax	[3]
d)	*Qyld.csdh.7AL*	*Xgwm322*	20.0*	DH; Chinese Spring (DS)/SQ1(DT)	SQ1	[4]
**2. 1000 grain weight**						
a)	*3B*	*Xbarc101*	45.2	$F_{2:3}$ families; Massara-1 (DS)/ Oste-Gata (DT)	Oste-Gata	[5]
b)	*QTgw-7D-b*	*XC29-P13*	21.9	RIL; SeriM82/Babax	Babax	[6]
**3. Days to Heading**						
a)	*QDh-7D.b*	*XC29-P13*	22.7	RIL; SeriM82/Babax	Babax	[6]
b)	*QHd.idw-2A.2*	*Xwmc177*	32.2	RIL; Kofa/Svevo	Kofa	[7]
**4. Days to Maturity**						
a)	*QDm-7D.b*	*X7D-acc/cat-10*	22.7	RIL; SeriM82/Babax	Babax	[6]
**5. Stem Reserve Mobilization**						
a)	*QSrm.ipk-2D*	*Xgwm249a*	42.2	RIL; W7984/Opata 85	W7984	[8]
b)	*QSrm.ipk-5D*	*Xfbb238b*	37.5	RIL; W7984/Opata 85	W7984	[8]
c)	*QSrm.ipk-7D*	*Xfbb189b*	21.0	RIL; W7984/Opata 85	W7984	[8]
**6. Water soluble carbohydrate**						
a)	*QWsc-c.aww-3A*	*Xwmc0388A*	19.0	DH; Kukri (DS)/RAC875 (DT)	RAC875	[9]
**7. SPAD/chlorophyll content**						
a)	*Qchl.ksu-3B*	*Xbarc68*	59.1	RIL; HUW206 (DS)/C306 (DT)	C306	[10]

[a] Highest PVE (R^2) values under drought/water stress; * with >20% higher yield per ear, DS, drought sensitive; DT, drought tolerant.

1. Shukla S, Singh K, Patil R V, Kadam S, Bharti S, Prasad P, et al. Genomic regions associated with grain yield under drought stress in wheat (*Triticum aestivum* L.). Euphytica. 2015;203: 449–467. doi:10.1007/s10681-014-1314-y
2. Kirigwi FM, Van Ginkel M, Brown-Guedira G, Gill BS, Paulsen GM, Fritz AK. Markers associated with a QTL for grain yield in wheat under drought. Mol Breed. 2007;20: 401–413. doi:10.1007/s11032-007-9100-3
3. Pinto RS, Reynolds MP, Mathews KL, McIntyre CL, Olivares-Villegas J-J, Chapman SC. Heat and drought adaptive QTL in a wheat population designed to minimize confounding agronomic effects. Theor Appl Genet. 2010;121: 1001–1021. doi:10.1007/s00122-010-1351-4
4. Quarrie SA, Pekic Quarrie S, Radosevic R, Rancic D, Kaminska A, Barnes JD, et al. Dissecting a wheat QTL for yield present in a range of environments: From the QTL to candidate genes. J Exp Bot. 2006;57: 2627–2637. doi:10.1093/jxb/erl026
5. Golabadi M, Arzani A, Mirmohammadi Maibody SAM, Sayed Tabatabaei BE, Mohammadi SA. Identification of microsatellite markers linked with yield components under drought stress at terminal growth stages in durum wheat. Euphytica. 2011;177: 207–221. doi:10.1007/s10681-010-0242-8
6. Lopes MS, Reynolds MP, McIntyre CL, Mathews KL, Jalal Kamali MR, Mossad M, et al. QTL for yield and associated traits

in the Seri/Babax population grown across several environments in Mexico, in the West Asia, North Africa, and South Asia regions. Theor Appl Genet. 2013;126: 971–984. doi:10.1007/s00122-012-2030-4

7. Maccaferri M, Sanguineti MC, Corneti S, Ortega JLA, Salem M Ben, Bort J, et al. Quantitative trait loci for grain yield and adaptation of durum wheat (*Triticum durum* Desf.) across a wide range of water availability. Genetics. 2008;178: 489–511. doi:10.1534/genetics.107.077297

8. Salem KFM, Roder MS, Borner A. Identification and Mapping Quantitative Trait Loci for Stem Reserve Mobilisation in Wheat (*Triticum aestivum* L.). Cereal Res Commun. 2007;35: 1367–1374.

9. Bennett D, Izanloo A, Reynolds M, Kuchel H, Langridge P, Schnurbusch T. Genetic dissection of grain yield and physical grain quality in bread wheat (*Triticum aestivum* L.) under water-limited environments. Theor Appl Genet. 2012;125: 255–271. doi:10.1007/s00122-012-1831-9

10. Kumar S, Sehgal SK, Kumar U, Prasad PVV, Joshi AK, Gill BS. Genomic characterization of drought tolerance-related traits in spring wheat. Euphytica. 2012;186: 265–276. doi:10.1007/s10681-012-0675-3
